# Univariate and multivariate QTL analyses provide insights into genetic architecture of variation of key volatile aroma compounds in cucumber (*Cucumis sativus* L.)

**DOI:** 10.1007/s00122-026-05393-5

**Published:** 2026-10-01

**Authors:** Thi Nhien Hao Nguyen, Cassidy Duan, Uijeong An, Oyindamola Akinpelu, Yassine Yahia, Xiaofen Du, Yiqun Weng

**Affiliations:** 1 Department of Plant and Agroecosystem Sciences, University of Wisconsin-Madison, Madison, WI 53706 USA; 2 Department of Nutrition and Food Sciences, Texas Woman’s University, Denton, TX 76209 USA; 3 USDA-ARS Vegetable Crops Research Unit, Madison, WI 53706 USA

## Abstract

*****Key message***:**

**Univariate and multivariate (PC-based) QTL analyses in multiple segregating populations and environments identify 284 QTL and 52 PC-QTL for 24 volatile aroma compounds in cucumber, including two environmentally stable QTL clusters, each harboring 26 and 25 QTL for major C**_6_/C_9_ **alcohols and aldehydes, respectively.**

**Abstract:**

Consumers enjoy cucumber for its refreshing sensation, which is primarily associated with volatile organic compounds (VOCs) produced by the lipoxygenase biosynthesis pathway. However, our knowledge of cucumber flavor genetics remains limited. Here, we report QTL mapping of VOCs in multiple segregating populations grown under greenhouse and open-field conditions. VOC abundance exhibited highly quantitative inheritance with strong environmental effects and low broad‐sense heritability. Transgressive segregation and underdominance were observed for most VOCs. Univariate QTL analysis identified 284 QTL for 24 VOCs, averaging 17 QTL for the six most abundant and aromatically significant VOCs. Both additive and dominance effects contributed significantly to VOC variation. Consistent with the strong correlation among many VOCs, 154 of the 284 QTL were co-localized into 15 distinct clusters. Multivariate principal component (PC)-based QTL analysis identified 52 PC-QTL that captured the maximum covariances among VOCs. Results from both methods were highly congruent, including the identification of two population-independent and environmentally stable QTL clusters, each harboring 26 and 25 QTL for major C_6_/C_9_ alcohols and aldehydes, respectively. The *cos*^*2*^ and loading patterns of PCs mapped to these QTL clusters suggested closely linked loci rather than pleiotropic effects. Analysis of sequence variation and expression of annotated genes inside the two clusters identified several transcription factor genes as potential candidates. This study reveals a complex genetic architecture of VOC variation in cucumber. We discuss the results in the context of allele effects, segregation distortion, the association between QTL clustering and the LOX pathway, and implications for cucumber breeding for flavor enhancement.

**Supplementary Information:**

The online version contains supplementary material available at https://doi.org/10.1007/s00122-026-05393-5.

## Introduction

Aroma is an important quality attribute of fruits and vegetables, directly impacting consumer acceptance. It is primarily determined by volatile organic compounds (VOCs), which are secondary metabolites, including terpenoids, derivatives of amino acids, carotenoids, and fatty acids (Dudareva et al. 2013). The quantities, dominant classes, and combinations of VOCs are characteristic features of different crops and varieties. For example, climacteric fruits such as tomatoes and melons contain over 400 VOCs, predominantly fatty acid-derived aldehydes, alcohols, and esters, with profiles shifting dramatically during ripening (Klee and Giovannoni 2011; Mayobre et al. 2021). In contrast, immature commercial-stage cucumber fruit typically contains ~150 VOCs, with fatty acid-derived aldehydes and alcohols being the most prevalent (Du et al. 2022a; Wei et al. 2016). Beyond their sensory roles, VOCs also serve critical ecological functions, including attracting pollinators, activating plant defenses against pathogens and herbivores, protecting against abiotic stress, and facilitating plant-to-plant signaling (Dicke and Baldwin 2010; Dudareva et al. 2013; Engelberth 2020). These distinct VOC profiles likely reflect different selection histories during adaptation, domestication, or breeding driven by human preferences.

Cucumber (*Cucumis sativus* L.) is consumed for its immature fruit (typically 7–12 days after anthesis) and is appreciated for its unique refreshing flavor and crisp texture (Du et al. 2022a, b; Ramirez et al. 2022; Williams et al. 2022). The number and relative abundance of VOCs vary widely among genotypes and are influenced by various factors such as growing conditions, developmental stages, organ or tissue types, post-harvest handling practices and processing methods (Chen et al. 2015; Du et al. 2022a; Li et al. 2019; Min et al. 2023; Wei et al. 2016; Zhang et al. 2022). Nevertheless, in most of these studies, six- (C_6_) and nine-carbon (C_9_) aldehydes dominate the VOC profiles of cucumber varieties, which typically account for 74–82% of total volatiles with (E,Z)-2,6-nonadienal, (E)-2-nonenal, and hexanal being the most abundant. Among the many VOCs, each imparts a unique aroma or odor (details in Table S1), and their impact on human sensory perception is often quantified by the aroma value (content relative to perception threshold). The two C_9_ aldehydes, (E,Z)-2,6-nonadienal and (E)-2-nonenal, exhibit both high abundance and exceptionally low odor thresholds, making them the primary contributors to the characteristic cucumber-like aroma and refreshing perception (Chen et al. 2015; Du et al. 2022a, b; Schieberle et al. 1990; Williams et al. 2022; Zhang et al. 2022).

Cucumber VOCs are produced upon tissue disruption through enzymatic degradation of linoleic and linolenic acids via the lipoxygenase-hydroperoxide lyase (LOX-HPL) pathway that involves 9-/13-LOX, 9-/13-HPL, alcohol dehydrogenases (ADH), and isomerases (ISO) (illustrated in Fig. S1) (Galliard et al. 1976; Phillips and Galliard 1978; Vincenti et al. 2019). Genes encoding these enzymes often exist as gene families in the cucumber genome, and their expression is influenced by genotypes, fruit development stages, and environmental conditions (Chen et al. 2015; Matsui et al. 2000; Spyropoulou et al. 2017; Yang et al. 2012, 2020). However, the genetic basis underlying natural variation in cucumber VOCs remains poorly understood. An early study suggested that major cucumber VOCs exhibit quantitative inheritance, involving both additive and dominance effects (Xu et al. 2012). More recently, Sun et al. (2022) identified 34 QTL for 19 VOCs in a recombinant inbred line (RIL) population, including a major QTL *qol8-2.1* for (E,Z)-2,6-nonadienol on chromosome 2 co-localized with a LOX gene cluster, and one of which (*CsLOX8*) was proposed as a candidate for* qol8-2.1.*

The objective of this study is to identify the genetic basis underlying VOC variation in cucumber using complementary univariate and multivariate (principal component or PC-based) QTL mapping approaches. We analyzed 24 LOX-derived VOCs in multiple segregating populations grown under greenhouse and open-field environments. Early-generation populations (F_2_ and F_2:3_) enabled the capture of initial recombination events and detection of dominance effects, while the RIL population provided enhanced resolution and statistical power for QTL detection. By integrating results from multiple populations and environments, and employing both univariate and multivariate QTL analyses, this study achieves a comprehensive and robust identification of stable, environmentally independent QTL for pivotal aroma compounds in cucumber.

## Materials and methods

### Mapping populations, sample collection, and VOC analysis

Segregating F_2_, F_2:3,_ and RIL populations (population ID: WI7799) were developed from the cross between cucumber lines 9930 and WI7633, which display different sensory attributes and VOC profiles (Du et al. 2022a,b; Williams et al. 2022). A total of 135 F_2_ plants, 135 F_2:3_ families, and 135 F_6_/F_7_ RILs were grown at the University of Wisconsin Walnut Street Greenhouses (WSGH) or open fields at the Hancock Agriculture Research Station (HARS). The details of all trials are presented in Table S2. Briefly, individual F_2_ plants were grown in WSGH with a completely randomized design (CRD; Population ID: F2). F_2:3_ families were evaluated in WSGH (CRD with no replication; F3GH) and in HARS field with a randomized complete block design (RCBD) with two biological replications (F3HR1, F3HR2). All RILs were grown with RCBD in either the greenhouse (RILGHR1 and RILGHR2) or the HARS field (RILHR1 and RILHR2) with two biological replications.

Fruits were harvested at 10–14 days post-anthesis for VOC quantification using solid-phase microextraction (SPME) on a Shimadzu GC-2010 gas chromatograph with a QP2020 mass spectrometer (GC–MS) and an AOC-600 autosampler, following established protocols (Du et al. 2022b). VOC abundance was quantified as the integrated peak area under the chromatographic curve. Although more than 140 VOCs were detected, our subsequent analysis focused on 24 C_6_/C_9_ aldehydes and alcohols derived from the LOX pathway (Table S1). Four of the 24 VOCs were detected in only one environment with trace amounts, which were thus excluded from summary statistics and heritability estimates. To allow comparison of VOC quantities across populations and environments, missing values were first imputed using the *missForest* package in R. The imputed peak area of each VOC in each sample was then normalized to the total peak area of all 20 VOCs in that sample.

VOC names were abbreviated using a format based on their structural characteristics: isomer configuration (E/trans or Z/cis), double bond position (2, 3, 4 or 6), carbon chain length ("h" for C_6_; "n" for C_9_), number of double bonds ("e" for one or “de” for two), and structure group (“ol” for alcohol; “al” for aldehyde). For example, “hal” and “EZ26ndeal” denote hexanal and (E,Z)-2,6-nonadienal, respectively (Table S1).

### Linkage map construction

Linkage maps were constructed for WI7799F_2_ and WI7799RIL populations. For F_2_, genotyping-by-sequencing (GBS) was performed at the University of Wisconsin Genomics Core Facility using the *Ape*KI enzyme and 151-bp paired-end sequencing on the NovaSeq 6000 platform. Raw reads were processed using the GB-eaSy pipeline and aligned to the 9930v3.0 reference genome (Wickland et al. 2017). High-quality, biallelic SNP markers were retained using filtering criteria of minor allele frequency (MAF) < 0.02, read depth < 10, and missing data > 25%. Marker ordering by minimizing the sum of adjacent recombination frequencies and grouping of binned markers were performed using *MapRtools* R package. The Hidden Markov Model (*HMM* R package) inferred founder haplotype dosage based on transition and emission probabilities from observed genotypes.

A Diversity Arrays Technology (DArTag) genotyping platform was used to genotype the RIL population using a 3000-SNP panel (Lin et al. 2026). Map construction followed the same procedures as the F_2_ population.

### Univariate and multivariate QTL analyses

QTL analysis was performed on all 24 VOCs using R*/qtl*. For univariate mapping, peak areas of individual VOCs were analyzed by population, environment, and replication using composite interval mapping (CIM), followed by two-dimensional genome scans (*scantwo*). LOD thresholds were determined by 1,000 permutations at a significance level of α = 0.05. Final multi-QTL models were refined using *stepwiseqtl* and fitted with *fitqtl*. Additive effects (*A*) were estimated for all populations, while dominance effects (*D*) were estimated for F_2_ and F_2:3_ populations only. Positive *A* values indicate the 9930 allele increases VOC abundance. The percentage of phenotypic variance explained (PVE) by each QTL was quantified. QTL physical positions were defined by 1.5-LOD support intervals on the 9930v3.0 genome assembly.

For PC-based multivariate QTL mapping, the phenotype matrix of raw VOC peak areas (no transformation of data) was first standardized by centering and scaling each compound to unit variance (R/*base* package), rendering the analysis equivalent to a correlation-based PCA so that each VOC contributes equally, regardless of its absolute abundance. Missing values in the standardized matrix were then imputed using the Expectation–Maximization algorithm, with the optimal number of components estimated using the *missMDA* R package. PCA was performed on the standardized, imputed matrix with the *prcomp* function. Estimated parameters include variance percentages, eigenvalues, variable loadings, squared cosine values (*cos*^*2*^), and PC scores. QTL mapping was performed on the top five PCs and on TotalPC, a weighted composite of the top five principal components in which each PC score is weighted by its normalized proportion of explained variance among the components.

### QTL naming conventions, consensus QTL, and QTL clusters

QTL were named following established conventions in cucumber, consisting of the abbreviated VOC name, chromosome number, and sequential identifier (e.g., *EZ26ndeal1.1*) (Wang et al. 2020). A consensus QTL was defined as a genomic region identified by integrating multiple overlapping QTL detected for the same VOC across multiple populations or environments with concordant effect directions. QTL clusters (prefixed ‘voc’) were defined as chromosomal regions harboring QTL for at least two VOCs within overlapping confidence intervals, potentially reflecting pleiotropy or tight linkage. Clusters containing QTL with opposite directions of additive effects were distinguished by suffixes ‘a’ and ‘b’ (e.g., voc2.1a versus voc2.1b).

### Broad-sense heritability estimation and statistical analyses

For F_2_ and F_2:3_ populations, the broad-sense heritability (*H*^*2*^) was estimated utilizing the parent–offspring regression method (Fernandez and Miller, 1985). *H*^*2*^ for RIL populations was estimated using a linear mixed model implemented in R*/lme4* (Bates et al. 2015), treating environment as a fixed effect, and family, family-by-environment interaction, and replication nested within environment as random effects.

Linear mixed-effects model: Y_ijk_=μ+G_i_+E_j_+(GE)_ij_+R_k(j)_+ε_ijk_

$$i = \, \left\{ {{1}, \, \ldots \, ,{135}} \right\}{\text{ families}}$$

$$j = \, \left\{ {{1},{ 2}} \right\}{\text{ environments}}$$

$$k = \,\,\left\{ {{1},{ 2}} \right\}{\text{ replicates}}$$

where: Y_ijk_ = the observed trait value for the *i*^th^ family in the *j*^th^ environment and *k*^th^ replication

$$\mu \, = {\text{ the overall mean}}$$

$${\mathrm{G}}_{i} = {\text{ the random effect of the}}\,i^{{{\mathrm{th}}}} \,{\mathrm{family}},{\text{ G}}_{i} \sim {\mathrm{N}}\,\left( {0,\sigma_{G}^{2} } \right)$$

$${\mathrm{E}}_{{\mathrm{j}}} = {\text{ the fixed effect of the}}\,j^{{{\mathrm{th}}}} \,{\mathrm{environment}}$$

$$\begin{aligned} \left( {{\mathrm{GE}}} \right)_{{{\mathrm{ij}}}} = & {\text{ the random effect of family}}\\&{\text{-by-environment interaction}},{\text{ }}\left( {{\mathrm{GE}}} \right)_{{{\mathrm{ij}}}} \sim {\mathrm{N}}\,\,\left( {0,\sigma _{{GE}} ^{2} } \right) \\ \end{aligned}$$

$$\begin{aligned} {\mathrm{R}}_{{{\mathrm{k}}({\mathrm{j}})}} = & {\text{ the random effect of replication }} \\&{\text{nested within environment}},{\text{ R}}_{{{\mathrm{k}}({\mathrm{j}})}} \sim {\mathrm{N}}\,\,\left( {0,{\text{ }}\sigma _{{\mathrm{R}}} ^{{\mathrm{2}}} } \right) \\ \end{aligned}$$

$$\varepsilon_{{{\mathrm{ijk}}}} = {\text{ the residual error}}, \, \varepsilon_{{{\mathrm{ijk}}}} \sim {\mathrm{N}}\,\,\left( {0, \, \sigma_{\varepsilon }^{{2}} } \right)$$

The family mean-based *H*^*2*^ was calculated using the following formula (Holland et al. 2003):

$${H}^{2} = \frac{{\upsigma}_{\mathrm{G}}^{2}}{{\upsigma}_{\mathrm{G}}^{2}+ \frac{{\upsigma}_{\mathrm{G}\mathrm{E}}^{2}}{\mathrm{e}}+\frac{{\upsigma}_{\upvarepsilon }^{2}}{\mathrm{r}\mathrm{e}}}$$

where ${\upsigma}_{\mathrm{G}}^{2}$ is the genetic variance among families, ${\upsigma}_{\mathrm{G}\mathrm{E}}^{2}$ is the family-by-environment interaction variance, ${\upsigma}_{\upvarepsilon }^{2}$ is the residual error variance, *e* = {1, 2} environments, and *r* = {1, 2} replications per environment.

Peak areas were used for QTL mapping, while normalized peak areas (imputed; percentage of total LOX C_6_/C_9_ VOCs) were used for visualizing phenotypic variation and enabling comparison across populations and environments. Spearman correlation coefficient (*r*_*s*_) was calculated from peak areas to measure correlations among VOCs (α = 0.01). Skewness was assessed from normalized data using D’Agostino’s test, with |skewness| > 1 and *p* < 0.05 indicating significant deviation from symmetry. Coefficients of variation were calculated as (SD/mean) × 100% to measure relative variation. Best linear unbiased estimates (BLUEs) across environments were obtained from raw peak areas using R*/lme4* to compare VOC abundance among the parental lines (9930, WI7633) and their F_1_ (WI7799F1), which were included in all phenotyping trials.

### Inference of candidate genes in voc2.1 and voc3.1 QTL clusters

Genomic sequences from targeted regions were extracted from HiFi genome assemblies of two parental lines 9930 and WI7633 (Zhao et al. 2026). Sequence alignment and variant calling were performed with *minimap2.* Structural variants were identified using *SVIM-asm* in *svimenv* conda environment. Annotated genes in the target region from both the 9930v3.0 and Gy14v2.1 reference genomes (https://www.cucurbitgenomics.org/) were compared and analyzed for potential candidate genes in the voc2.1 and voc3.1 QTL clusters.

Transcriptional levels of candidate genes in fruit tissues at different developmental stages were examined using publicly available RNA-seq datasets from the NCBI database to provide further evidence of their potential functional relevance to fruit aroma. Precomputed gene expression values, expressed as fragments per kilobase of transcript per million mapped reads (FPKM), were extracted from two earlier studies: SRA071224 (Wei et al. 2016) and GSE116581 (Lü et al. 2018). Data from the following samples were analyzed: flesh of 1-week-old (SRA_7), 2-week-old (SRA_14), and 3-week-old (SRA_21) fruit from SRA071224; and fruit at 10 (GSE_10), 20 (GSE_20), 30 (GSE_30), and 40 (GSE_40) days post-anthesis from GSE116581. For each candidate gene underlying a QTL, FPKM values across developmental stages were compiled and visualized as bar graphs to compare expression trends among genes carrying polymorphic variants between the two parental lines (9930 and WI7633).

## Results

### Complex quantitative inheritance of VOCs with strong environmental influences

**Fig. 1:**
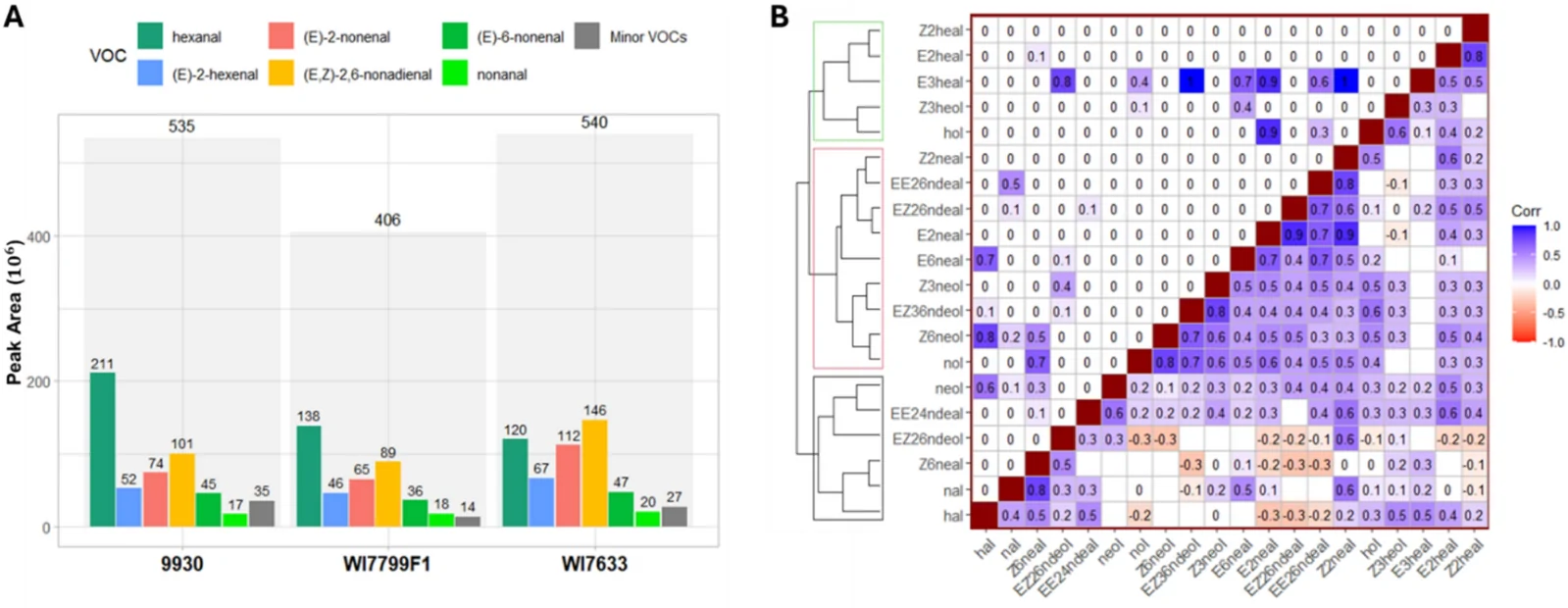
Phenotypic profiles of parental lines and correlations of C_6_ and C_9_ VOCs in WI7799 segregating populations. (**A**) Bar graphs showing the abundance of selected major VOCs in two parent lines (9930, WI7633) and their F_1_ (WI7799F1). The Y-axis represents the peak areas under the chromatographic curves, which are BLUEs (Best Linear Unbiased Estimates) from all environments. (**B**) Correlation plot showing Spearman correlation coefficients (lower triangle) and *p*-values (upper triangle) among VOCs, which were calculated from peak-area values of all individual samples pooled across all mapping populations and environments. The dendrogram for all VOCs to the left is based on hierarchical clustering of the correlation matrix. Blank cells indicate non-significant correlation at *p* > 0.01

SPME–GC–MS identified 140–165 VOCs across parental lines and WI7799 segregating populations, including 24 LOX-derived C_6_/C_9_ aldehydes and alcohols that together accounted for ~50% of total VOCs. Using BLUEs across trials (Fig. 1A and Table S3), >90% of LOX-derived VOCs in 9930, WI7633, and WI7799F_1_ were C_6_ and C_9_ aldehydes (Fig. S2). The six most abundant VOCs were hexanal, (E)-2-hexenal, (Z)-6-nonenal, nonanal, (E)-2-nonenal, and (E,Z)-2,6-nonadienal. While total VOC amounts were comparable between 9930 and WI7633, the relative abundance of individual VOCs differed significantly. WI7633 had higher C_9_ aldehydes (60.4%) but lower C_6_ aldehydes (34.1%) and total alcohols (5.6%) than 9930, with (E,Z)-2,6-nonadienal, (E)-2-nonenal, and (E)-2-hexenal being the predominant contributors. In contrast, hexanal accumulated 1.76-fold higher in 9930 than in WI7633 (Fig. 1A). Notably, total VOC content in F_1_ was ~75% of parental levels, with 18 of 20 VOCs in F_1_ being lower than the mid-parental values, indicating underdominance or negative heterosis in the F_1_ hybrid.

VOC variation in the segregating populations is highly quantitative and environmentally sensitive. The means and range of variation of 20 VOCs in the RILs are presented in Table S3. Field-grown RILs had significantly higher levels of total alcohols (9.9%) and lower levels of total C_9_ aldehydes (42.2%) compared to those grown in greenhouses (3.8 and 47.2%, respectively), while total C_6_ aldehydes remained similar (Fig. S2), highlighting strong environmental effects on VOC accumulation.

Across mapping populations and environments, most C_9_ VOCs were positively correlated (Fig. 1B**)**. The strongest positive correlation was between (E,Z)-2,6-nonadienal and (E)-2-nonenal (*r*_*s*_ = 0.88, *p* < 0.0001). Hexanal and (Z)-6-nonenal showed significant negative correlations with major C_9_ aldehydes, with *r*_*s*_ values ranging from −0.2 to −0.3. The significant correlations among VOCs suggest a coordinated regulation of their accumulation, probably due to pleiotropic QTL or linked genomic regions.

**Fig. 2:**
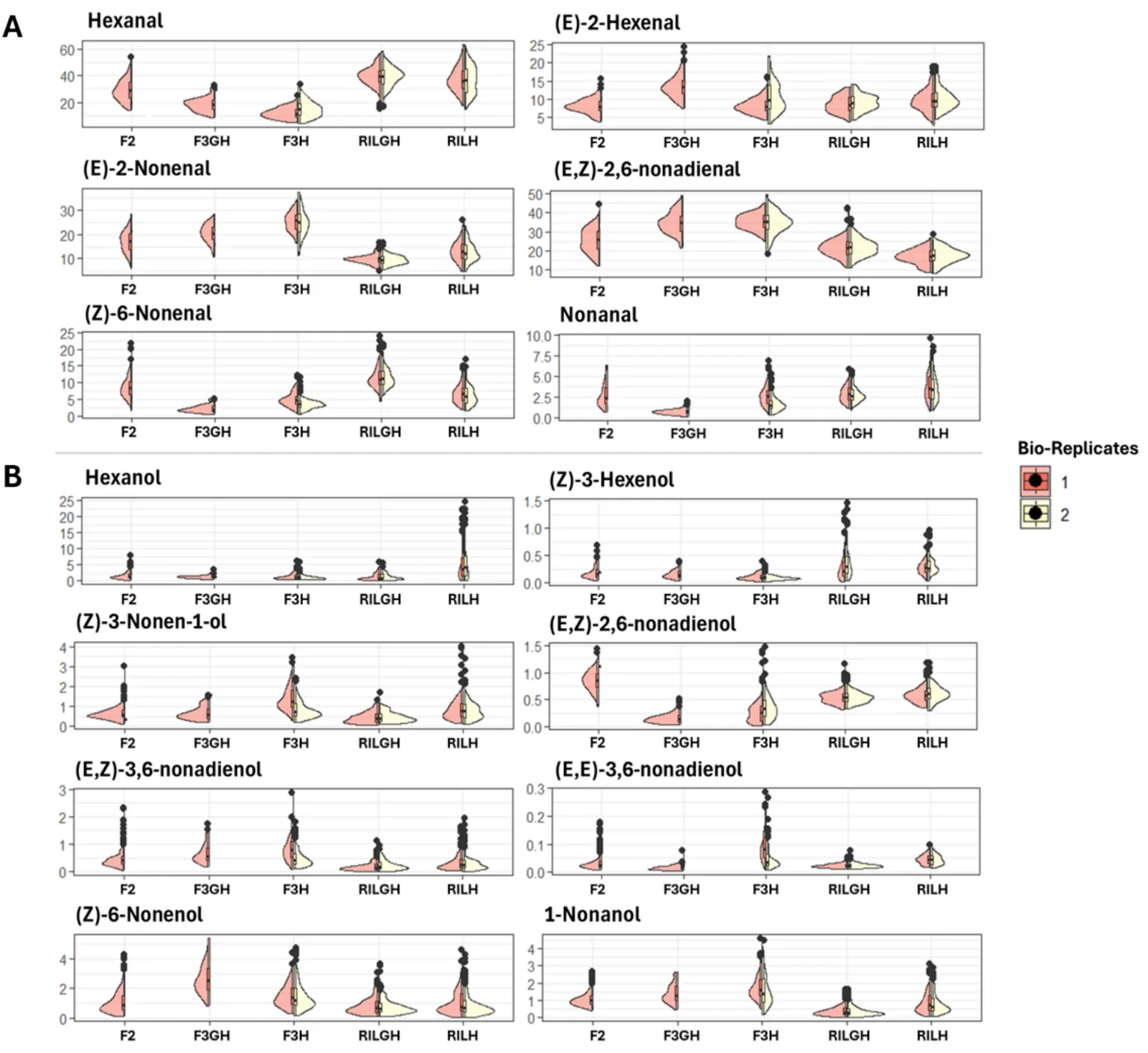
Split violin plots showing phenotypic variation of 14 LOX-pathway associated volatile organic compounds in different WI7799 segregating populations and environments. (**A**) Major C_6_ and C_9_ aldehydes. (**B**) Major C_6_ and C_9_ alcohols across populations. The left and right halves represent biological replicates 1 and 2 (where applicable), respectively, with overlaid boxplots indicating medians and interquartile ranges. Values for each VOC are normalized against the total peak areas (in percentages) of all C_6_ and C_9_ VOCs*.* GH = greenhouse; H = open-field

Split violin plots of six major aldehydes and eight alcohols by biological replications and environments (Fig. 2) showed congruent distributions between replications but a clear environmental influence. Compared to greenhouse conditions, both F_2:3_ and RIL populations exhibited broader phenotypic ranges under field conditions, suggesting greater expression of genetic variation. Notably, the analysis also revealed that most VOCs (~ 70%) exhibited highly right-skewed distributions (values cluster at lower abundance), particularly alcohols, whereas several major aldehydes approximated normal distributions (Fig. 2 and Table S4). The coefficient of variation ranged widely from 11.5 to 99.8%, with C_6_ alcohols being the most variable and C_9_ aldehydes comparatively stable. Together, these data suggest a complex quantitative and environmentally responsive nature of the variation of C_6_ and C_9_ VOCs in cucumber.

In agreement with this, the overall mean broad-sense heritability (*H*^*2*^) for all C_6_ and C_9_ VOCs was low (0.217), but it varied depending on populations, environments, and individual VOCs (Table 1). Mean *H*^*2*^ from F_2:3_ greenhouse trials (0.227) exceeded field trials (0.172), and RIL estimates (0.297) exceeded F_2:3_ family estimates (0.199). Also, VOCs with high abundance seem to have higher heritability, with (E,Z)-2,6-nonadienal exhibiting the highest *H*^*2*^ (0.539 from RIL data).

**Table 1:** Estimated broad-sense heritability (*H*^*2*^) of C_6_ and C_9_ VOCs in WI7799 F_2:3_ and RIL populations

#	VOCs	Abbr.	F3H	F3GH	RILs	*H*^*2*^_Mean
1	Hexanal	hal	0.172	0.161	0.232	**0.188**
2	1-Hexanol	hol	0.292	0.096	0.277	**0.222**
3	(E)-2-Hexenal	E2heal	0.068	0.206	0.533	**0.269**
4	(Z)-2-Hexenal	Z2heal	-	0.349	0.400	**0.375**
5	(Z)-3-Hexen-1-ol	Z3heol	0.103	0.326	0.480	**0.303**
6	(E)-3-Hexenal	E3heal	-	-	0.027	**0.027**
7	(E)-2-Nonenal	E2neal	0.385	0.287	0.430	**0.367**
8	(Z)-3-Nonen-1-ol	Z3neol	0.032	0.424	0.327	**0.261**
9	(E,E)-2,6-nonadienal	EE26ndeal	0.036	0.075	0.234	**0.115**
10	(E,Z)-2,6-nonadienal	EZ26ndeal	0.113	0.281	0.539	**0.311**
11	(E,Z)-2,6-nonadienol	EZ26ndeol	0.173	0.176	0.349	**0.233**
12	(E,Z)-3,6-Nonadien-1-ol	EZ36ndeol	0.231	0.476	0.172	**0.293**
13	(E,E)-3,6-Nonadien-1-ol	EE36ndeol	-	0.034	-	**0.034**
14	Nonanal	nal	0.191	0.081	0.390	**0.221**
15	1-Nonanol	nol	0.307	0.325	0.148	**0.260**
16	(E)-6-Nonenal	E6neal	0.186	-	0.076	**0.131**
17	(Z)-6-Nonenal	Z6neal	0.103	0.059	0.358	**0.173**
18	(Z)-6-Nonenol	Z6neol	0.241	0.274	-	**0.258**
19	(E,E)-2,4-Nonadienal	EE24ndeal	0.064	-	0.072	**0.068**
20	1-Nonen-4-ol	neol	0.229	-	-	**0.229**
	**Mean**		**0.172**	**0.227**	**0.297**	**0.217**

### Linkage map development and detection of segregation distortion regions (SDRs)

**Fig. 3:**
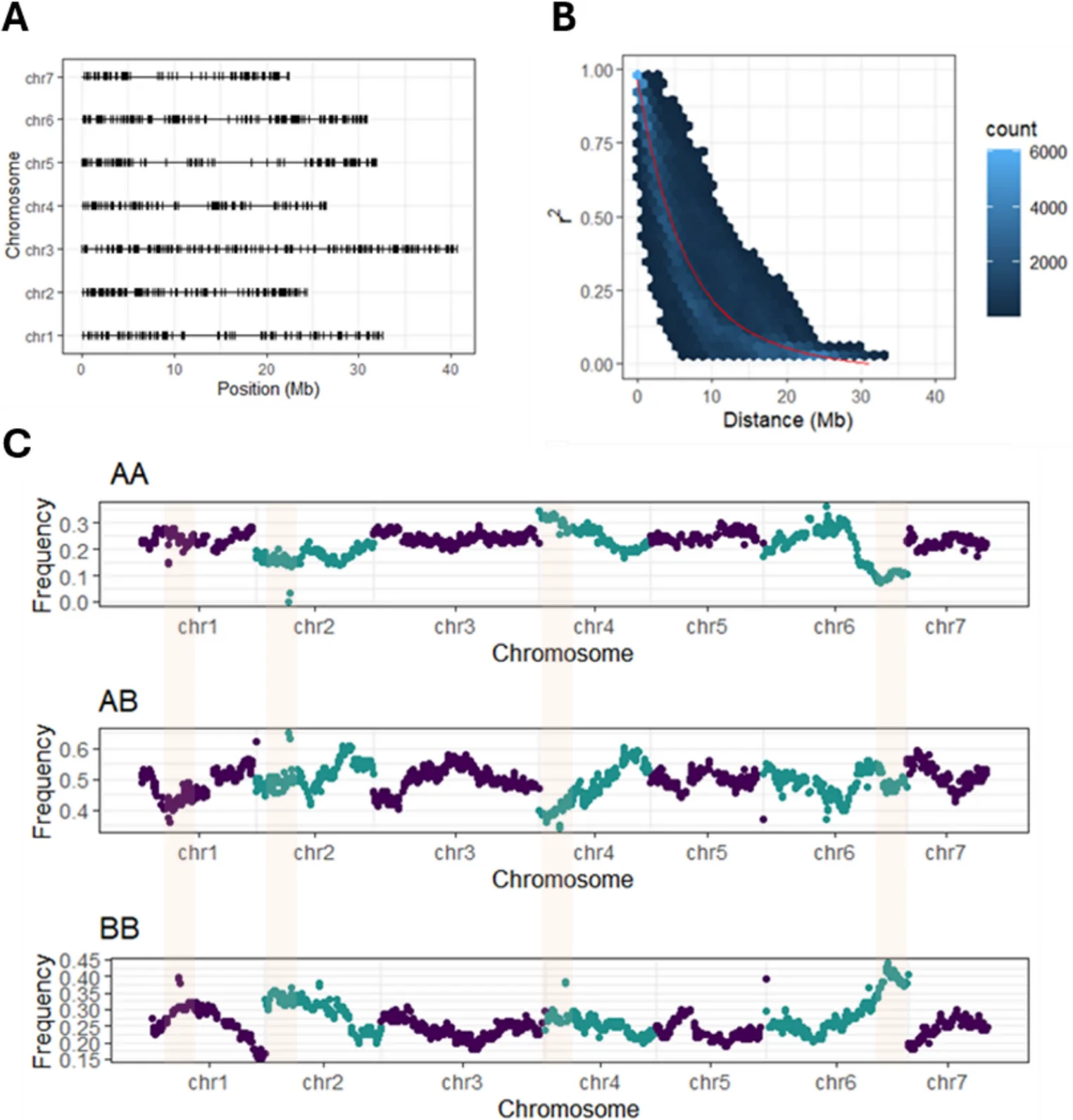
Characterization of WI7799 F_2_ (n=135) linkage map. (**A**) Marker distribution on seven chromosomes. (**B**) Hexbin plot showing LD decay over physical distances (Mbp). The red line is a monotone spline to illustrate trends of LD decay. (**C**) Marker genotype frequency across chromosomes for homozygotes WI7633 (AA), 9930 (BB), and heterozygotes (AB). Shaded bars indicate four major segregation distortion regions

For F_2_, 369,676 variants were called from GBS reads. After filtering, 1,912 high-quality binned SNPs were selected for linkage analysis. The resulting map spanned 1,076.6 cM across seven linkage groups (Table S5). The markers were evenly distributed across the genome except for several highly heterochromatic regions (Fig. 3A). The F_2_ population, derived from a single generation of selfing, exhibited smooth and relatively slow linkage disequilibrium (LD) decay, with LD values dropping below 0.2 at approximately 10 Mb (Fig. 3B). Most markers showed a monotone pattern and exhibited the expected 1:2:1 segregation ratio in genotype frequencies (0.25 AA: 0.5 AB: 0.25 BB) (Fig. 3C). Notably, chi-square goodness-of-fit tests of mapped loci in this population (Table S6) identified four major SDRs: three with 9930 alleles preferentially transmitted (Chr1: 4–7 Mbp; Chr2: 0–9 Mbp; Chr6: 21–30 Mbp) and one favoring WI7633 alleles (Chr4: 0–4 Mbp).

The WI7799RIL linkage map contained 334 binned marker loci spanning 947.9 cM (Table S5 and Fig. S3A). While the RIL map was slightly shorter than the F_2_ map (with same population size) and with lower average recombination rate (4.70 cM/Mbp in RIL versus 5.25 cM/Mbp in F_2_), the two maps span nearly identical physical distances (~ 205.1 Mbp in F_2_ versus 206.1 Mbp in RIL) (Table S5). The longer map length in F_2_ is likely due to inflated recombination caused by genotypic errors. Markers in F_2_ plants were generated with low-coverage GBS, which is prone to errors in variant calling, particularly for the heterozygous calls that are expected at ~50% of loci in an F_2_ population that require a greater read depth to resolve confidently, whereas the near-homozygous RILs are largely free of this error-prone class. Indeed, compared with the RILs, in F_2_, higher recombination rates were observed primarily on densely genotyped chromosomes, whereas the two chromosomes with the lowest marker density (Chr4 and Chr7) in F_2_ showed lower recombination rates than in RILs (Table S5). Furthermore, linkage disequilibrium (LD) decayed more rapidly in the RIL than in the F_2_ (*r*^*2*^ below 0.2 at ~7 Mb versus ~10 Mb; Fig. S3B and Fig. 3B), indicating more recombination events in the RILs as expected.

Consistent with findings on the F_2_ map, two large SDRs of ~9 Mbp in length on Chr2 and Chr6 were also detected on the RIL map (mean chi-square = 6.72 and 32.66, respectively) (Fig. S3C and Table S7). Both regions consistently favor the transmission of the 9930-derived allele. If a major-effect QTL is located within an SDR, distorted genotype frequencies may shift the phenotypic distribution of the corresponding VOCs, as seen from the skewed patterns observed for most C_6_ and C_9_ alcohols (Fig. 2).

### Univariate QTL mapping reveals a complex genetic basis of VOC variation

Univariate QTL analysis detected 284 QTL for 24 VOCs with 54, 66, 41, 57, and 66 from five population/environments, F2, F3GH, F3H, RILGH, and RILH, respectively. Details of these QTL (peak markers, LOD, PVE, additive/dominance effects, intervals, etc.) are presented in Table S8, with summary statistics and visual presentation in Table S9 and Fig. S4, respectively. The six most abundant VOCs each had at least 9 QTL (mean of 17) detected, whereas low-abundance VOCs yielded 2–6 QTL. For (E,Z)-2,6-nonadienal, the most abundant VOC in WI7633, 19 QTL were identified, of which 14 showed moderate to major effects (PVE > 15%). Most QTL exhibited negative additive and dominance effects (mean *A* = −1.69, *D* = −0.62), indicating WI7633 alleles increase (E,Z)-2,6-nonadienal content (Fig. S5A). Three consensus QTL were identified: *EZ26ndeal1.1/1.2* on Chr1 (*A* = −2.43, PVE = 12.29%), *EZ26ndeal3.2/3.3/3.4* detected exclusively in RILs on Chr3 (*A* = −2.71, PVE = 21.79%), and *EZ26ndeal4.4/4.5* on Chr4 (*A* = −2.63, PVE = 15.02%). These results suggest a complex genetic architecture underlying this key flavor VOC.

Nineteen QTL were detected for (E,Z)-2,6-nonadienol, a corresponding alcohol with 1/40–1/30 the abundance of (E,Z)-2,6-nonadienal, exhibiting low negative additive effects (average *A* = −0.12) and strong dominance effects (average *D* = −1.22). The two compounds shared QTL in three clusters: voc1.2 (*EZ26ndeal1.1*/*1.2* and *EZ26ndeol1.3*), voc7.1a (*EZ26ndeal7.1* and *EZ26ndeol7.1*), and voc7.3 (*EZ26ndeal7.3* and *EZ26ndeol7.3*). Notably, additive effects at voc1.2 showed opposite directions for this C_9_ alcohol-aldehyde pair, with significant *A* values of −2.43 and 3.65 and PVE of 12.29% and 23.64%, respectively, which were consistent with their weak negative phenotypic correlation (*r*_*s*_ = −0.15, *p* ≤ 0.001).

**Fig. 4:**
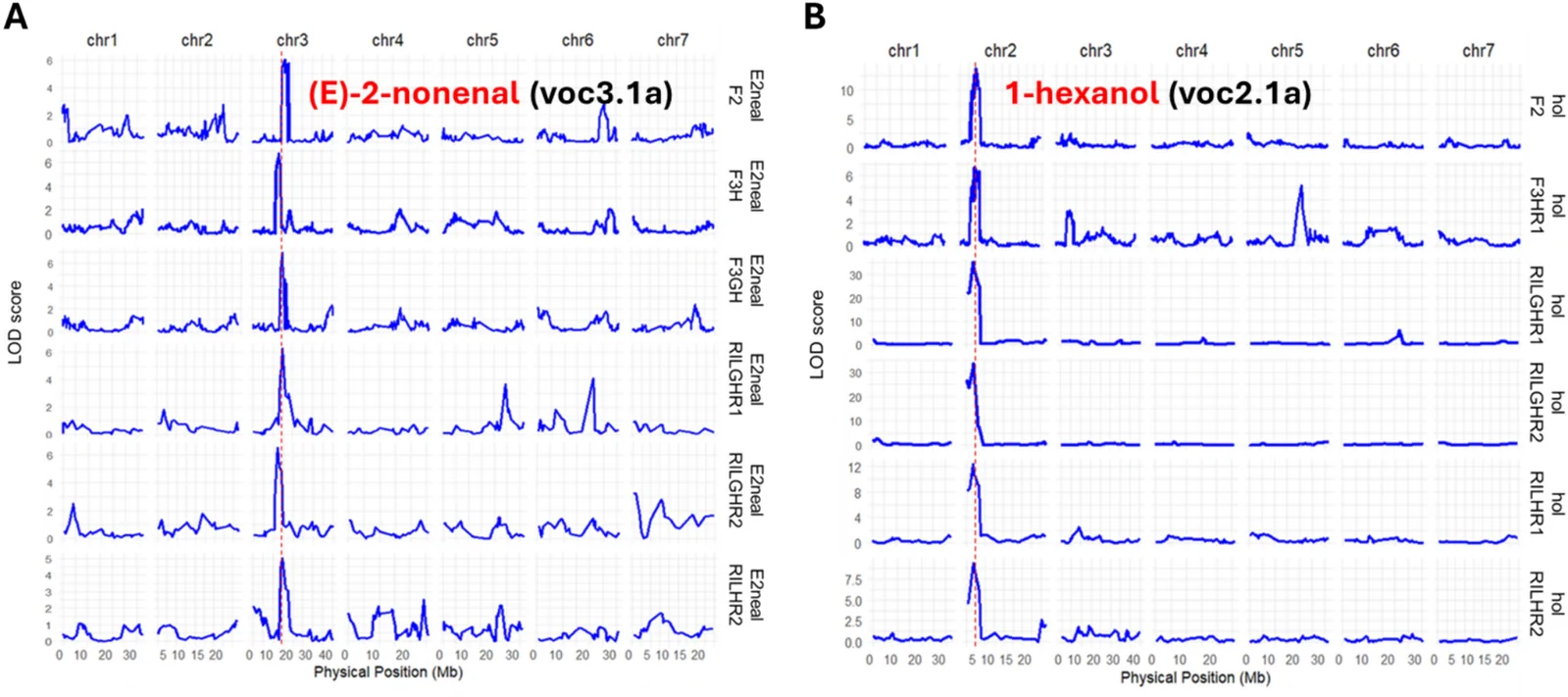
Genome-wide LOD profiles (CIM) highlighting consensus QTL in two regions for (**A**) (E)-2-nonenal (E2neal) on Chr3 and (**B**) 1-hexanol (hol) on Chr2 across three populations (F_2_, F_2:3_, and RIL) and two environments (GH = greenhouse; H = open-field). The consensus QTL for each VOC belongs to QTL subclusters voc3.1a and voc2.1a, with physical intervals of ~4.9 Mbp and 1.5 Mbp and mean PVE of 17.9% and 36.9%, respectively

Eighteen QTL were detected for (E)-2-nonenal, with 10 co-localized into three consensus QTL in three QTL clusters (mean *A* = −2.67) (Fig. S5B). A robust consensus QTL, comprising 6 QTL *E2neal3.1* to *E2neal3.6*, was consistently detected across all populations and environments, mapping to the ~12.9–17.8 Mbp region on Chr3 (LOD = 4.2–12.66, average PVE = 17.9% and *A* = −4.86). This indicates a highly stable, environment-independent, major-effect QTL underlying the accumulation of this predominant C_9_ aldehyde (Fig. 4A).

Twenty-one QTL were detected for C_6_ aldehyde hexanal, the most abundant in 9930, F_1,_ and RIL populations (mean *A* = 1.81 and *D* = −2.26) (Fig. S5C). Two consensus QTL were identified: *hal3.2/3.3/3.4/3.5* in a ~6.5 Mbp region on Chr3 detected in F_2_ and RIL under greenhouse environments (mean PVE = 14.38%, *A* = 4.38, *D* = −2.75), and *hal4.3/4.4* in a 1.5 Mbp region on Chr4 under both growing conditions (mean PVE = 20.19%, *A* = 4.57, *D* = −2.45). These QTL exhibited concordantly strong positive additive effects and negative dominance effects.

Three isomeric C_6_ aldehydes, (E)-2-hexenal, (Z)-2-hexenal, and (E)-3-hexenal, showed positive intercorrelations (Fig. 1B). Nineteen, 14, and 8 QTL were detected, all with negative additive (−2.36, −1.81, and −0.65) and dominance (−1.88, −1.24, and −1.28) effects, respectively (Fig. S5D). Of the 19 and 14 QTL for (E)-2-hexenal and (Z)-2-hexenal, 8 and 7 were co-localized in five clusters including voc1.1a (*E2heal1.1*/*1.2*, *Z2heal1.1*/*1.2*), voc3.1a (*E2heal3.2*/*3.3*/*3.4, Z2heal3.2*/*3.3*), voc6.1a (*E2heal6.1* and *Z2heal6.1*), voc6.2 (*E2heal6.2* and *Z2heal6.2*), and voc7.1b (*E2heal7.1* and *Z2heal7.1*). The shared QTL clusters exhibited concordantly predominant negative additive and dominance effects, indicating that the same parental alleles consistently altered both aldehyde abundances. This concordant allelic direction may explain their strong correlation (*r*_*s*_ = 0.83, *p* < 0.001) despite (E)-2-hexenal being 30–60 times more abundant than (Z)-2-hexenal.

Among detected alcohols, 1-hexanol and (Z)-3-hexen-1-ol were positively correlated in abundance (*r*_*s*_ = 0.64, *p* < 0.001), with 19 and 11 QTL detected, respectively. Notably, a consensus QTL for 1-hexanol (*hol2.1–2.6*) (Fig. 4B) and a consensus QTL for (Z)-3-hexen-1-ol (*Z3heol2.1*–*2.4*), detected across all populations and environments, were co-localized within a ~1.5 Mbp region on Chr2. The 1-hexanol consensus QTL exhibited a high mean LOD of 16.97 (LOD = 21.66 in RILs), high PVE of 36.94%, and strong additive and dominance effects (mean *A* = −8.23; *D* = −2.40), while the (Z)-3-hexen-1-ol consensus QTL showed PVE of 28.63% (41.77% in RILs) and mean *A* = −5.89 (*A* = −7.14 in RILs). These results demonstrate enhanced detection power with RILs and suggest a major-effect, environmentally stable QTL regulating both C_6_ alcohols.

As for (Z)-6-nonenal, 16 QTL were detected with concordant negative dominance effects, consistent with underdominance observed in F_1_ (PVE range = 6.6–38.1%, mean *A* = 1.46, *D* = −2.11). A reliable major consensus QTL (*Z6neal2.3*/*2.4*/*2.5*) was detected in both growing conditions, with mean PVE = 22.36% and *A* = 3.14. Nonanal, which showed a strong correlation with (Z)-6-nonenal (*rₛ* = 0.83, *p* < 0.001), had 9 QTL, 7 of which were co-localized with (Z)-6-nonenal QTL in five clusters and exhibited concordant effect directions (PVE ranging 10.1–42.5%). For its corresponding alcohol, 1-nonanol, 15 QTL were detected including two major consensus QTL on Chr1 (*nol1.3/1.4*) and Chr2 (*nol2.1/2.2*) with strong effects (PVE = 28.87% and 60.96%, *A* = 5.08 and −11.04, respectively).

### QTL clusters harboring environmentally independent and stable QTL for multiple VOCs

By integrating information from all 284 QTL, we identified 15 QTL clusters containing 154 QTL for 24 VOCs. Eleven clusters contained subgroups a and b with opposite additive effects (Table 2). These clusters harbored 70 of 102 QTL (68.6%) for the six most abundant and aromatically important VOCs, and the subgroup size ranged from 1 to 23 QTL.

**Table 2:** QTL clusters for 24 C_6_ and C_9_ VOCs from univariate QTL analysis in WI7799 mapping populations*

#	QTL cluster	Chr.	Peak marker position	LOD range	1.5-LOD Left	1.5-LOD Right	Additive Effect	Dominance Effect	PVE range (%)	# QTL in cluster	VOCs associated with QTL in cluster	Putative LOX pathway branches involved
1	**voc1.1a**	1	1,450,884	3.16–6.21	143,552	7,273,355	-4.35	-0.67	8.33–16.57	7	Z2heal, Z6neol, E2heal, nol	13HPOT, UKN
2	**voc1.1b**	1	1,709,026	9.33–10.39	143,552	2,625,328	2.88	-2.18	18.93–26.93	2	E2neal, EZ36ndeol	9HPOD, 9HPOT
3	**voc1.2a**	1	32,137,606	3.52–9.66	27,750,202	32,509,306	-2.73	0.02	8.35–23.81	4	E2neal, EZ26ndeal	9HPOD, 9HPOT
4	**voc1.2b**	1	31,791,378	4.20–10.62	27,750,202	32,509,306	3.41	-3.18	11.18–25.59	6	nol, EZ26ndeol, EZ36ndeol, Z3neol, Z6neol, Z6neal	9HPOD, 9HPOT, UKN
5	**voc2.1a**	2	4,371,493	3.40–33.95	3,396,451	4,824,086	-7.19	-2.28	9.26–72.49	23	EZ26ndeol, EZ36ndeol, Z3neol, hol, Z3heol, Z6neol, nol, Z2neal, EE26ndeal	9HPOD, 9HPOT, 13HPOD 13HPOT, UKN
6	**voc2.1b**	2	3,597,964	3.60–4.81	2,710,577	10,099,499	4.11	-1.65	10.09–15.57	3	Z6neal, nal	UKN
7	**voc2.2**	2	10,238,310	6.19–17.22	7,998,780	11,255,780	3.26	-1.88	8.91–42.48	7	E6neal, nal, Z2neal, Z6neal	9HPOD, UKN
8	**voc3.1a**	3	16,270,109	3.97– 12.66	12,914,770	17,801,004	-4.26	0.72	9.12–34.23	18	E2heal, E2neal, EZ26ndeal, Z2heal, Z2neal, Z6neol, nol	9HPOD, 9HPOT, 13HPOT, UKN
9	**voc3.1b**	3	13,879,294	3.97–35.48	13,877,026	16,270,109	4.30	-1.11	9.69–47.13	7	EZ36ndeol, Z3neol, Z3heol	9HPOD, 9HPOT, 13HPOT, UKN
10	**voc3.2a**	3	35,079,083	3.22–14.24	33,342,419	40,103,997	-4.68	-1.05	7.26–30.82	9	E2heal, EZ26ndeal, EE26ndeal, Z6neal, nal	9HPOT, 13HPOT, UKN
11	**voc3.2b**	3	34,246,720	5.44–9.33	32,178,481	37,606,195	4.38	-2.75	5.44–9.33	4	hal	13HPOD
12	**voc4.1**	4	5,655,847	3.30–10.18	3,285,372	7,163,873	4.00	-1.08	8.68–18.21	7	EZ36ndeol, Z3neol, Z6neol, hal, Z2heal	9HPOD, 9HPOT, 13HPOD, 13HPOT, UKN
13	**voc4.2a**	4	17,394,480	4.16–9.79	16,352,114	20,819,509	-0.80	-4.41	11.49–28.38	3	Z2heal, E2neal, EZ26ndeal	9HPOD, 9HPOT, 13HPOT
14	**voc4.2b**	4	17,586,005	4.18–11.02	17,205,059	18,192,798	3.23	-3.04	6.63—20.66	6	Z3neol, hal, Z6neal, E2heal	9HPOD, 13HPOD, 13HPOT, UKN
15	**voc4.3a**	4	24,397,821	4.37–18.37	23,317,980	24,891,253	-2.83	-	12.06–38.12	7	nal, Z6neal, EZ26ndeal, E3heal, E2neal	9HPOD, 9HPOT, 13HPOT, UKN
16	**voc4.3b**	4	24,397,821	13.48	23,317,980	24,397,821	1.89	-	29.12	1	Z2neal	9HPOD
17	**voc5.1a**	5	21,841,655	5.93–19.62	20,014,870	24,169,934	3.30	-3.50	13.05–49.42	9	hal, hol, nal, nol, Z6neal, EZ26ndeol, EZ36ndeol	9HPOT, 13HPOD, UKN
18	**voc5.1b**	5	21,841,655	8.99	14,116,630	24,169,934	-3.19	-5.39	49.14	1	neol	UKN
19	**voc6.1a**	6	20,957,428	3.47–4.49	15,869,056	21,726,330	-3.61	-	7.89–11.34	4	E2neal, E2heal, Z2heal, neol	9HPOT, 13HPOD, UKN
20	**voc6.1b**	6	20,957,428	8.92	15,869,056	21,419,544	2.78	-	24.3	1	EZ26ndeal	9HPOT
21	**voc6.2**	6	23,869,621	3.44–10.03	22,178,946	25,829,271	-3.54	-3.73	8.50–21.71	6	hal, E2heal, Z2heal, E2neal, E6neal, EZ26ndeal	9HPOD, 9HPOT, 13HPOD, 13HPOT, UKN
22	**voc7.1a**	7	2,362,263	4.50–8.28	1,300,411	3,020,781	-0.86	-1.42	11.01–16.07	3	hal, EZ26ndeal, EZ26ndeol	9HPOT, 13HPOD
23	**voc7.1b**	7	2,355,322	4.38–12.34	1,870,234	3,191,128	4.24	-6.41	10.09–53.55	4	EE36ndeol, E2heal, Z2heal	9HPOT, 13HPOT
24	**voc7.2**	7	8,849,179	7.05–14.62	5,291,372	9,817,231	4.56	-4.20	17.61–62.79	6	hal, Z3heal, E6neal, Z6neol, EE26ndeal, EZ26ndeol	9HPOT, 13HPOD, 13HPOT, UKN
25	**voc7.3a**	7	19,757,897	6.14–7.49	18,668,087	20,416,776	-2.72	-1.82	15.41–18.19	4	Z6neal, E2neal, EZ26ndeol, EZ26ndeal	9HPOD, 9HPOT, UKN
26	**voc7.3b**	7	18,152,556	3.40–5.37	12,890,052	22,314,692	2.69	-	5.97–12.35	2	Z3heol, EZ26ndeol	9HPOT, 13HPOT

*PVE: percentage of variance explained. All marker positions are from 9930v3.0. UKN: unknown; al: aldehyde; ol: alcohol

The voc2.1a subcluster, spanning ~1.5 Mb on Chr2, harbored 23 environmentally stable, major-effect QTL (mean LOD = 14.97, PVE = 35.2%, *A* = −7.47) for seven alcohols: (E,Z)-2,6-nonadienol, (E,Z)-3,6-nonadien-1-ol, (Z)-3-hexen-1-ol, (Z)-3-nonen-1-ol, (Z)-6-nonenol, 1-hexanol, and 1-nonanol. All QTL showed strong and concordant negative additive effects, implying reduced alcohol abundance associated with the 9930 alleles. In contrast, voc2.1b contained three QTL with positive additive effects for two C_9_ aldehydes: (Z)-6-nonenal and nonanal, with average *A* of 4.11.

The voc3.1 cluster harbored 25 QTL for 10 VOCs. Subgroup voc3.1a contained 18 QTL, 15 of which were associated with five aldehydes, including three dominant contributors to cucumber fruit aroma: (E,Z)-2,6-nonadienal (3 QTL), (E)-2-nonenal (6 QTL, Fig. 4A), and (E)-2-hexenal (3 QTL), with mean LOD = 6.34, PVE = 16.33%, and *A* = −4.27. Subgroup voc3.1b contained 7 QTL for three alcohols: (E,Z)-3,6-nonadien-1-ol, (Z)-3-hexen-1-ol, and (Z)-3-nonen-1-ol (mean LOD = 5.94, PVE = 16.69%, and *A* = 4.3). All VOCs regulated by QTL in voc3.1 were derived from 9-/13-HPOT and 9-HPOD (Fig. S1), suggesting a conserved biochemical basis through which the WI7633 allele enhances major aldehyde production while reducing alcohol accumulation.

The remaining 13 clusters contained environmentally stable QTL for LOX pathway-specific VOC groups. For instance, voc1.2a (4 QTL) and voc1.2b (6 QTL) subgroups were associated with two C_9_ aldehydes (PVE = 16.84%, *A* = −2.73) and five C_9_ alcohols (PVE = 17.18%, *A* = 3.13), respectively, corresponding to the 9-HPOD and 9-HPOT branches of the LOX pathway. The voc2.2 cluster contained 7 QTL for four C_9_ aldehydes in the 9-HPOT branch (PVE = 24.04%; *A* = 3.26). Additional aldehyde-specific clusters included voc3.2a (9 QTL for 5 VOCs, PVE = 14.9%, *A* = −4.68), voc3.2b (4 QTL for hexanal only, PVE = 14.38%, *A* = 4.38), voc4.3a (7 QTL for five aldehydes, PVE = 22.41%, *A* = −2.83), and voc6.2 (6 QTL for six aldehydes, PVE = 18.10%, *A* = −3.54).

### Multivariate QTL analysis validates QTL clusters for multiple aroma compounds

**Fig. 5:**
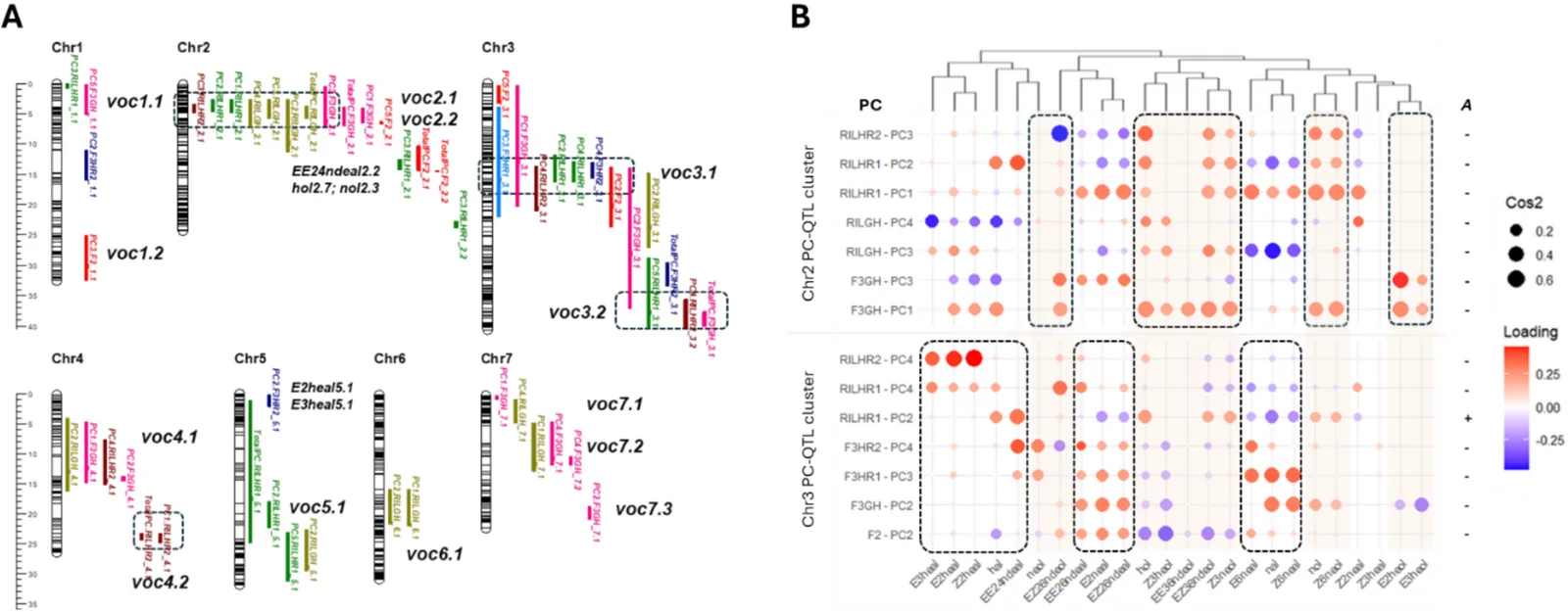
PC-based QTL analysis using the top five PCs and total PC score across multiple segregating populations and environments. (**A**) Physical distribution of PC-QTL across seven chromosomes (vertical bars). The scales to the far left show the physical length of chromosomes (9930v3.0). Horizontal lines on each chromosome are marker positions on the F_2_ linkage map. QTL clusters (boldface labels) and single-trait QTL from univariate analysis are positioned according to their confidence intervals and aligned with overlapping PC-QTL regions. (**B**) Bubble plot displaying the contribution (*cos*^*2*^) and correlation (loading values) of 24 VOCs across PCs derived from PC-QTL clusters on Chr2 (top panel, 7 PCs) and Chr3 (bottom panel, 7 PCs) co-localizing with univariate QTL clusters voc2.1 and voc3.1, respectively. Bubble size represents *cos*^*2*^ (contribution), and bubble color indicates loading direction (red, positive; blue, negative). VOCs are hierarchically clustered as shown by the top dendrogram, with alcohols distinguished by a shaded background. The rightmost column indicates the direction of additive effects (A+ and A-) for each corresponding PC-QTL (Table S10). GH = greenhouse; H = open-field; R1/R2: Biological replications 1 and 2

Principal component analysis (PCA) of 24 VOCs revealed that PC1 and PC2 explained 28.8–39.6% and 19.2–24.9% of variances, respectively (Fig. S6). QTL mapping using PC1–PC5 and Total PC scores identified 52 PC-QTL with 6, 18, and 28 from F_2_, F_2:3_, and RIL populations, respectively (Table S10). Twenty-eight of the 52 PC-QTL were co-localized with 13 of 15 univariate QTL clusters, indicating high concordance of results between the two approaches (Fig. 5A). Notably, 8 and 7 PC-QTL were co-localized with voc2.1 and voc3.1, respectively. To visualize VOCs that drive each PC-QTL, a bubble plot (Fig. 5B) was developed with two key statistics for each PC-VOC pair: *cos*^*2*^, which quantifies how strongly a VOC contributes to the variance explained by a given PC, and the loading value, which represents the correlation between each VOC and the PC axis. VOCs of contrasting colors vary in opposite directions within that PC.

Of the eight PC-QTL that co-localized with voc2.1, seven (*PC1.F3GH_2.1, PC3.F3GH_2.1, PC3.RILGH_2.1, PC4.RILGH_2.1*, *PC1.RILHR1_2.1*, *PC2.RILHR1_2.1,* and *PC3.RILHR2_2.1*) were used to interpret the underlying VOC variation, whereas *TotalPC.RILGH_2.1* was excluded because it reflects overall abundance rather than variation among specific compounds (Table S10 and Fig. 5A). The PCs associated with these seven QTL were primarily driven by variations of seven alcohols and two C_9_ aldehydes: (Z)-6-nonenal and nonanal, corresponding to QTL within the voc2.1 cluster from univariate QTL analysis (Table 2 and Fig. 5B). Mean additive and dominance effects of seven PC-QTL with positively loaded alcohols were −5.25 and −2.49, respectively (Table S10 and Fig. S7), which is consistent with univariate QTL results for the seven alcohols. Moreover, RILGH-PC3*,* RILHR1-PC1, and RILHR1-PC2 exclusively captured significant variations in (Z)-6-nonenal and nonanal. However, the two C_9_ aldehydes and the alcohol group showed inconsistent loading directions across PCs. They aligned in RILHR1-PC1, but were opposite in RILGH-PC3 and RILHR1-PC2, indicating that the variation in aldehydes and alcohols was captured independently rather than as a coordinated group. This suggests that their variation may be governed by two closely linked genes, each controlling a separate biochemical subcluster.

Similarly, seven PC-QTL co-localized with voc3.1 cluster, including *PC2.F2_3.1, PC2.F3GH_3.1, PC3.F3HR1_3.1*, *PC4.F3HR2_3.1, PC2.RILHR1_3.1, PC4.RILHR1_3.1* and *PC4.RILHR2_3.1* (Table S10). Their associated PCs primarily captured variations of 9-/13-HPOT and 9-HPOD aldehydes and alcohols, consistent with univariate QTL in voc3.1 cluster (Table 2 and Fig. 5B). Although RILHR1-PC2 showed opposite loading direction, this reflects the arbitrary orientation of PC axes rather than biological differences. The mean additive and dominance effects for positively loaded aldehydes and negatively loaded alcohols ((E,Z)-3,6-nonadien-1-ol, (Z)-3-nonen-1-ol, and (Z)-3-hexen-1-ol) were *A* = −5.07 and *D* = 0.69, consistent with the allele effects from univariate QTL results for voc3.1. Notably, RILHR2-PC4 uniquely captured significant variation in 13-HPOT-derived C_6_ aldehyde isomers (*A* = −5.31). This separate contribution of C_6_ aldehydes from C_9_ aldehydes and alcohols suggests that their accumulation is under separate genetic control, implying at least two causal genes within the voc3.1a subgroup. Together, these findings establish voc2.1 and voc3.1 as genetically complex regulatory hubs that contain multiple candidate genes controlling distinct VOC subsets.

## Discussion

### Inheritance and genetic architecture of VOC variation in cucumber

In this study, WI7633 and 9930 were selected as parental lines for developing segregating populations in QTL analysis of VOCs because they exhibit contrasting sensory properties (Williams et al. 2022). Aldehydes predominated in the profiles of WI7633 and 9930, accounting for 94.5% and 90.2% of total C_6_/C_9_ VOCs, respectively (Fig. S2). Although total VOC amounts were similar, WI7633 consistently accumulated higher levels of the four most abundant aldehydes: (E,Z)-2,6-nonadienal, (E)-2-nonenal, (E)-2-hexenal, and (Z)-6-nonenal, whereas 9930 accumulated higher levels of hexanal (Fig. 1A and Table S3). This observation aligns with earlier sensory studies showing WI7633 exhibits a stronger "cucumber-like" aroma and refreshing perception, suggesting distinct genetic regulation of VOC biosynthesis between the two parents (Williams et al. 2022). Interestingly, across WI7799 segregating populations, 18 of 20 VOCs exhibited underdominance, with F_1_ levels below mid-parent values (Table S3). Negative heterosis of VOC levels has been observed in tomato (Bineau et al. 2022) and cucumber, in which 15 cucumber F_1_ hybrids had lower abundance for multiple VOCs than the mid-parent value, including C_6_ alcohols and (E,Z)-2,6-nonadienal (Min et al. 2023). This suggests that negative heterosis of LOX-derived VOCs may be common in cucumber, though its occurrence may depend on specific VOCs, crosses, or even fruit development stages (Bineau et al. 2022). Due to the near-exclusive use of F_1_ hybrids in commercial cucumber production, this inheritance pattern warrants further investigation.

Environmental factors such as light, temperature, soil conditions, and cultivation practices are known to affect VOC accumulation (Li et al. 2019; Shan et al. 2020; Xue et al. 2024). In this study, we observed greater increases and broader variation of total VOCs in the RILs grown in the field than in the greenhouses (Table S3). Some cucumber VOCs, especially C_6_ aldehydes (green leaf volatiles), play important roles in biotic and abiotic stress responses (Matsui et al. 2006). Thus, the increased accumulation of VOCs in RILs under field conditions may be associated with elevated defense functions.

Several VOCs, particularly alcohols and some C_9_ aldehydes, displayed right-skewed distributions with clustering toward low abundance, which was more pronounced in the RIL population (Fig. 2). This pattern is mainly attributed to QTL cluster voc2.1, which harbors major-effect QTL (mean PVE 45.3% in the RILs) for these VOCs within a ~9 Mbp segregation distortion region (SDR) on Chr2 (mean chi-square = 6.72) (Fig. 3C and Fig. S3C). The skewness, however, seems to be driven more by the magnitude of QTL effect than the distortion itself. For example, among the RIL QTL within the strongly distorted SDR on Chr6 (21–30 Mbp; mean chi-square = 32.66), smaller-effect QTL *EZ26ndeal6.2* and *E2neal6.2* (PVE 14.4% and 21.7%, respectively) did not significantly skew their VOC distributions, whereas the co-localized major-effect QTL *E6neal6.1* (PVE 39.9%) produced a strongly skewed distribution (Tables S4 and S8). The persistence of these distortion regions across generations also warrants further investigation, as understanding their maintenance has important implications for marker-assisted flavor breeding in cucumber.

Most VOCs showed low broad-sense heritability (Table 1), reflecting strong environmental influence and substantial genotype-by-environment interactions. Low heritability appears common for VOCs and secondary metabolites in crops. Xu et al. (2012) estimated heritability for four C_9_ aldehydes at 0.087–0.219 in cucumber, while Bauchet et al. (2017) reported average heritability of 0.41 and 0.37 for tomato VOCs (Bauchet et al. 2017; Xu et al. 2012). However, several abundant VOCs, including (E,Z)-2,6-nonadienal (0.539 in RILs), exhibited moderate heritability, suggesting that selection for key aroma compounds remains feasible.

Data from multi-population and multi-environment analyses allowed us to detect 284 QTL for 24 VOCs (average 11.83 QTL per VOC) in cucumber (Table S8), suggesting a complex genetic architecture of VOC variation in cucumber. This contrasts with Sun et al. (2022), who detected 34 QTL for 19 VOCs (1.8 per VOC) in an RIL population (Sun et al. 2022). Discrepancies between the two studies likely reflect differences in parental VOC profiles, number of segregating populations employed, phenotyping environments, and analytical approaches. The WI7799 F_2_, F_2:3,_ and RILs were used in this study, and ~55% of the 284 QTL were detected with F_2_ and F_2:3_ populations. Due to the polygenic and complex quantitative nature of VOC variation, RILs are commonly used for genetic dissection of VOCs to increase heritability and QTL detection power; however, they may lose the power of detecting the dominance effect due to a lack of heterozygosity (Sun et al. 2022). In tomato, dominance has been shown to contribute to variation in VOCs (Bineau et al. 2022; Hagenguth et al. 2024). Here, we found that both additive and dominance effects are important in explaining the observed variance in many VOCs (Tables S8 and S10). Our work emphasizes the importance of using early-generation populations that retain heterozygosity to gain a more complete picture of the VOC genetic basis.

### Multivariate QTL analysis disentangles pleiotropy from closely linked loci?

In the univariate approach, QTL analysis was performed on each trait independently, providing high sensitivity for trait-specific genetic effects and straightforward biological interpretation. However, when analyzing many correlated traits, this approach faces multiple testing burdens and may overlook shared genetic relationships. In contrast, PC-based QTL mapping transforms correlated traits into orthogonal principal components, promising to detect genomic regions affecting multiple traits through pleiotropy or tight linkage while increasing statistical power through dimensionality reduction (Ishikawa and Namikawa 2004; Ringnér 2008). For example, using a rice RIL population, Liu et al. (2021) compared both methods in QTL analysis for 16 mineral elements and identified QTL clusters that determined covariation among elements across environments. Yano et al. (2019) conducted PC-based association analysis in a GWAS panel on eight plant architecture-related traits and found PC1 provided the most information on traits that determine rice architecture. In this study, PC-based analysis identified 52 PC-QTL, and all except one (*PC3.RILHR1_2.2*) were co-localized with univariate QTL or QTL clusters for individual VOCs. Large numbers of PC-QTL were mapped to voc2.1 and voc3.1 clusters (Table S10 and Fig. 5), validating the capacity of PC-based mapping to capture significant VOC covariances. This result is in contrast with Liu et al. (2021), who identified many new QTL from PC-based QTL analysis, and the work by Keller et al. (2025), in which PC-based QTL analysis failed to identify two important QTL clusters from univariate QTL analysis for metabolomic and transcriptomic data in an Arabidopsis RIL population. Keller et al. (2025) reasoned that, since PCs capture both trait variation and linkage in the same vector, the initial PCA-based mapping flattens information across the genome and creates a population structure prior to QTL mapping. Inherently, it is difficult to interpret the results from PC-based QTL mapping in a biological context (Ringnér 2008). As such, data reduction needs to be carefully vetted for its usage in genetic mapping to ensure that the specific question of interest is being addressed (Keller et al. 2025).

Multiple PC-QTL mapped in voc2.1 and voc3.1 clusters may suggest pleiotropy or tight linkage among these QTL. To distinguish pleiotropy from tight linkage, we propose a bubble-plot framework (Fig. 5B) that compares *cos*^*2*^ values and loading patterns of PCs associated with each PC-QTL in the cluster across multiple populations and environments. Under a pleiotropic model, a single causal gene would consistently drive variation in multiple VOCs across all conditions, resulting in consistent patterns of *cos*^*2*^ and loading directions across PCs (allowing mirrored loading signs due to the arbitrary orientation of eigenvectors). In contrast, divergent *cos*^*2*^ and loading patterns would indicate distinct causal genes at tightly linked loci. This framework, to our knowledge, has not been previously formalized in any QTL mapping studies. Our work reveals that both voc2.1 and voc3.1 QTL clusters each contains more than one causal gene, which provides critical information on the genetic architecture of VOCs associated with the two clusters. Fine mapping and functional characterization will be necessary to confirm the presence of distinct causal genes within these intervals.

Taken together, our integrated use of both approaches presents a more complete picture of the genetic complexity underlying VOC variation than what either approach could achieve: univariate analysis detected QTL clusters by identifying overlapping genomic regions for individual VOCs, whereas the PC-based framework validated and offered the potential to distinguish whether VOCs within those clusters are controlled by a single pleiotropic gene or multiple tightly linked causal genes.

### QTL clusters and underlying biological complexity

QTL clustering for correlated traits is common in genetic mapping studies. For example, VOC mapping in melon identified one cluster on Chr08 containing 92 QTL and the candidate gene *ETHQV8.1* regulating climacteric ripening (Mayobre et al. 2021). In this study, we identified 15 QTL clusters containing 154 QTL for 24 VOCs. Notably, the two cucumber-like aroma compounds, (E,Z)-2,6-nonadienal and (E)-2-nonenal, showed the highest correlation (*r*_*s*_ = 0.88). Their QTL are co-localized in six clusters, suggesting tight co-regulation of their biosynthesis. Additional positively correlated VOC pairs with co-localized QTL are isomeric (E)-2-hexenal and (Z)-2-hexenal (*r*_*s*_ = 0.83, five shared clusters), C_9_ alcohols, (Z)-3-nonen-1-ol and 1-nonanol (*r*_*s*_ = 0.84, three shared clusters), and (E,Z)-3,6-nonadien-1-ol and (Z)-3-nonen-1-ol (*r*_*s*_ = 0.79, four shared clusters).

Nevertheless, for several reasons, identifying causal genes of these QTL or QTL clusters underlying VOC variation remains challenging. First, as discussed above in the cases of voc2.1 or voc3.1, QTL within the same cluster may represent tightly linked loci rather than a single pleiotropic gene. Some clusters contained QTL for VOCs derived from multiple LOX pathway branches with opposite allele effects, suggesting substrate competition or feedback regulation rather than independent biosynthetic control. Such complexity cannot be resolved without fine mapping of QTL in each cluster. Second, the LOX pathway enzymes are encoded by gene families with members that may differ in substrate specificity, tissue expression, or developmental timing, which are poorly characterized in cucumber. For example, the (Z)-3:(E)-2 isomerase is encoded by two gene paralogs acting on C_6_ and C_9_ substrates with tissue-specific expression (Spyropoulou et al. 2017). Without tissue-specific expression data, prioritizing candidate genes could be misleading in this case. Third, environmental sensitivity further complicates interpretation, as many VOC QTL respond to growing conditions (Sun et al. 2022), reflecting dual roles in flavor and stress defense. Finally, the 24 VOCs analyzed represent only part of a broader metabolic network (Feussner and Wasternack 2002; Vincenti et al. 2019), so regulatory effects outside the pathway may create false leads. Together, these factors highlight the necessity of fine mapping of identified VOC QTL in the future to better understand the molecular mechanisms underlying VOC regulation in cucumber.

### Candidate genes for *voc2.1* and *voc3*.1 QTL clusters?

Despite the challenges described above, we can still make a reasonable speculation of possible candidate genes for the voc2.1 and voc3.1 clusters which harbor 26 and 25 QTL, respectively. Physically, the two regions span ~1.5 Mbp and 4.9 Mbp, with 157 and 394 annotated genes in the 9930v3.0 assembly, and 126 and 333 in the Gy14v2.1 genome, respectively. We examined sequence variation in voc2.1 and voc3.1 regions and identified 118 and 234 genes (in 9930v3.0; 98 and 173 in Gy14v2.1) that have at least one sequence polymorphism between 9930 and WI7633, which are listed in Table S11. Data of normalized gene expression (in FPKM) of these genes at different days post anthesis in cucumber fruit flesh were extracted from the NCBI database (SRA071224 from Wei et al. 2016; GSE116581 from Lü et al. 2018) (Table S11). Among the 352 (9930v3.0) or 271 (Gy14v2.1) annotated genes, none seems to be associated with any biosynthetic genes in the LOX pathway. Sun et al. (2022) identified a major-effect QTL *qol8-2.1* for (E,Z)-2,6-nonadienol on chromosome 2, and proposed *CsLOX8* as a candidate for this QTL, which, however, lies outside the 1.5-Mbp physical interval of voc2.1 region.

In voc2.1 region, QTL are predominantly associated with C_6_ and C_9_ alcohols rather than their aldehyde precursors, suggesting these loci do not affect upstream LOX or HPL activity but instead promote downstream aldehyde-to-alcohol conversion, potentially through enhanced ADH activity or alcohol acyltransferase (AAT) function. It is plausible that transcription regulators rather than enzyme-coding genes may be involved in regulating biosynthesis of relevant VOCs. Transcription factors (TFs) are well-established regulators of aroma compound biosynthesis in fruits (Klee and Giovannoni 2011; Li et al. 2022, 2023a). For example, in tomato, a MADS box TF (RIN) regulates LOX pathway genes *TomLOXC*, *ADH2*, and *HPL,* thus influencing C_6_ aroma production (Qin et al. 2012), while overexpression of *SlMYB75* enhances C_6_ VOC accumulation (Jian et al. 2019). The strawberry FaMYB9 protein interacts with FaLOX5 to activate LOX pathway genes (Lu et al. 2020). In cucumber, a Dof TF encoded by *CsDof1.8* was reported to transcriptionally activate *CsLOX09* to enhance C_9_ VOC biosynthesis during fruit development (Sun et al. 2024).

We reasoned that if TF genes are potential candidates in the two clusters, they should show both sequence polymorphisms and largely consistent temporal expression patterns. In the two transcriptome datasets we analyzed (Table S11), two genes in the voc2.1 region seem to meet these criteria: *CsGy2G004930* encoding a bHLH149-like protein, and *CsGy2G005840* annotated as a homolog of Arabidopsis MYB36. In both experiments, they exhibited similar expression dynamics during fruit development (Fig. S8A). Both TFs are known to form transcriptional complexes, often with WD40-repeat proteins to regulate the expression of genes involved in biosynthesis of secondary metabolites, including VOCs (Medina-Puche et al. 2015; Goossens et al. 2017; Gao et al. 2018; Lu et al. 2020; Li et al. 2023b).

In the voc3.1 region, three TF genes, *CsGy3G020170* (encoding MYB1), *CsGy3G018030* (encoding MYB44), and *CsGy3G018410* (encoding ethylene-responsive factor 109 or ERF109) showed high expression in early fruit development, then declined (Fig. S8B; Table S11). In fleshy fruits, MYBs and ERFs play an important role in regulating ripening-dependent VOC biosynthesis (Li et al. 2022, 2023a,b; Lu et al. 2020; Mayobre et al. 2021). In addition, *CsGy3G017200* encoding a BAHD acyltransferase is also of interest. Genes in the BAHD acyltransferase family have very diverse functions in plants, and one group of this family is alcohol acyltransferases (AAT), which have been shown to play an important role in VOC biosynthesis, especially volatile esters (D’Auria et al. 2007; Goulet et al. 2014; Moghe et al. 2023). Thus, these six genes represent potential candidates for QTL inside the voc2.1 and voc3.1 regions regulating VOC abundance in the LOX biosynthesis pathway which merit additional investigations in future studies.

### Implications for cucumber breeding for enhanced fruit flavor

Our study revealed a complex genetic architecture underlying VOC variation in cucumber. Each VOC is controlled by multiple QTL with strong environmental influence and generally exhibits low heritability. Both additive and dominance effects contribute to VOC variation, and the frequent negative heterosis further complicates flavor breeding. Nonetheless, the strong positive correlation and QTL clustering among many VOCs (Fig. 1B and Table 2) may provide opportunities for simultaneous selection of multiple VOCs by targeting favorable alleles within clusters (e.g., voc2.1 for alcohols, voc3.1 for aldehydes). However, the negative correlations between hexanal and major C_9_ aldehydes imply that selecting for "cucumber-like" C_9_ aldehydes may reduce “green” C_6_ notes, and vice versa. Many VOCs, particularly C_6_ aldehydes, also contribute to plant defense, meaning that enhancing preferred aroma may inadvertently affect stress tolerance, highlighting key breeding trade-offs.

As in other vegetables, breeding cucumber flavor remains challenging (Tieman et al. 2017). Major-effect QTL require further refinement and validation across diverse genetic backgrounds and environments to evaluate environmental effects, genotype-environment interactions, and potential linkage drag. This is especially important because current QTL are derived from two fresh-market inbred lines, while US field-adapted, machine-harvest pickling cucumbers with characteristically low cucumber-like flavors remain genetically uncharacterized. Additionally, significant differences in QTL performance between field and protected environments warrant consideration for allele use in different production systems. Finally, equally important is integrating consumer preference into breeding programs. QTL analysis here used relative VOC abundance, yet VOCs differ in odor thresholds, and human liking depends on the balance of key VOCs rather than absolute levels (Chen et al. 2015; Tsuzuki 2019). Future efforts to improve cucumber flavor should combine genetic approaches with sensory and consumer evaluations.

## Supplementary Information

Below is the link to the electronic supplementary material.

Supplementary file1

Supplementary file2

## Data Availability

All data pertinent to the reported work are provided in the manuscript. Additional data is available upon request to the corresponding author.
