# Supplementary material for: Univariate and multivariate QTL analyses provide insights into genetic architecture of variation of key volatile aroma compounds in cucumber (Cucumis sativus L.)
Source: Theor Appl Genet. 2026 Oct 1;139(10):288. doi: 10.1007/s00122-026-05393-5 (PMC13630885; doi:10.1007/s00122-026-05393-5)
Supplement: Supplementary file 1 [file 122_2026_5393_MOESM1_ESM.pdf]

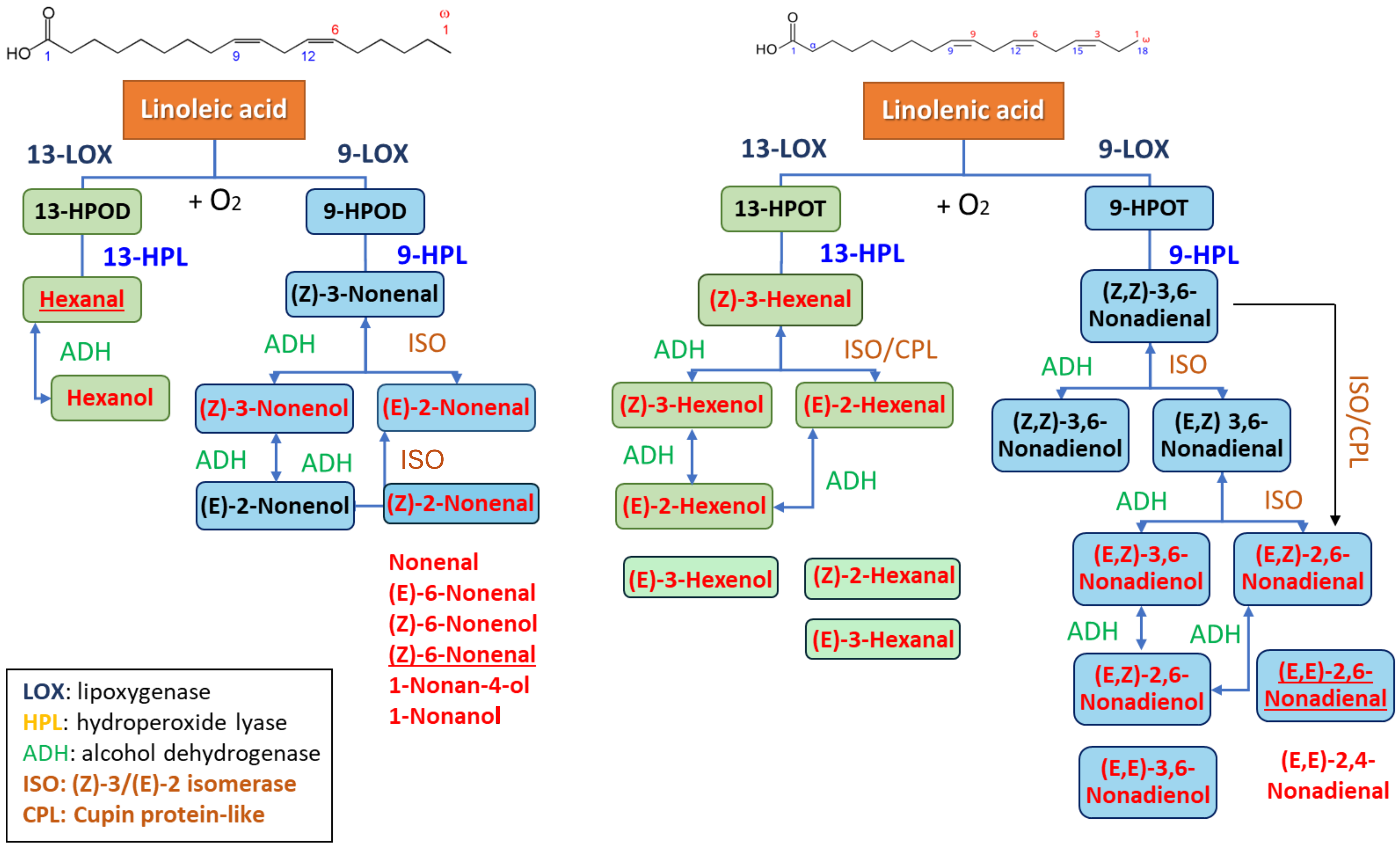

Figure S1 Key steps, biochemicals and enzymes in the lipoxygenase–hydroperoxide lyase (LOX–HPL) pathway for biosynthesis of major VOCs in commercial cucumber fruits. The names of 24 VOCs in red have QTL identified from this study. For the seven VOCs outside rectangles, their association with specific branches in the pathway is unknown. The underlined VOCs show generally negative correlations with other ones. HPOT: hydroperoxy octadecatrienoic acid (18:3), HPOD: hydroperoxy octadecadienoic acid (18:2).

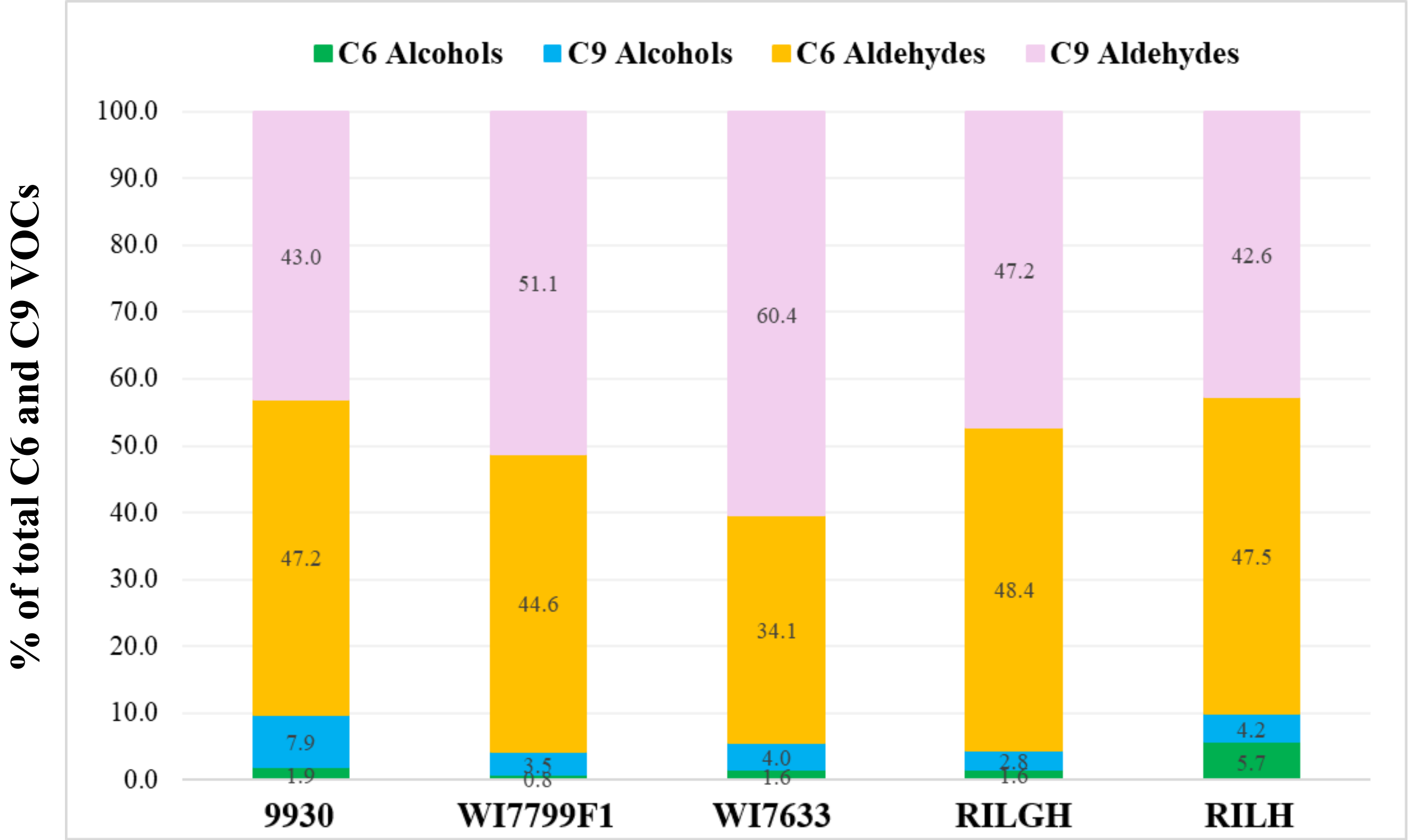

Figure S2 Stacked bar graph showing relative abundance of four groups of C_6 and C_9 VOCs in 9930, WI7633, their F_1 and the WI7799 RIL population. Values for 9930, WI7633 and WI7799F_1 are BLUEs from multiple trials. Values for RILs are normalized means of all RILs in either the greenhouse (RILGH) or open-field (RILH) trials in two years. The 20 VOCs used for this plot are listed in Supplemental Table S3.

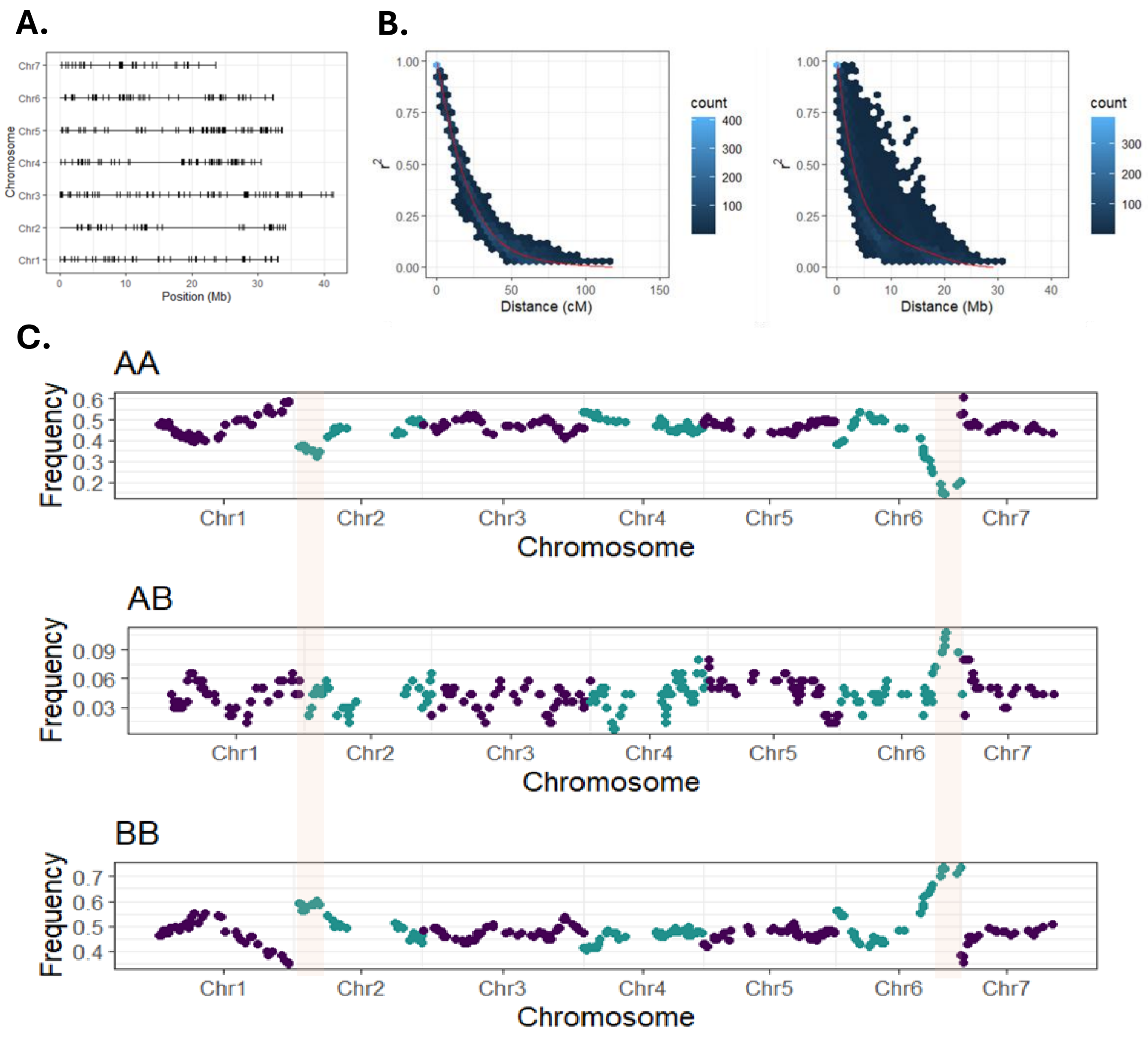

Figure S3 Characterization of WI7799 RIL linkage map. **(A)** Marker distribution on seven chromosomes. **(B)** Hexbin plot showing LD decay over physical distances (Mbp). The red line is monotone spline to illustrate trends of LD decay. **(C)** Marker genotype frequency across chromosomes for homozygotes WI7633 (AA), 9930 (BB), and heterozygotes (AB). Two shaded bars indicate segregation distorted regions on Chr2 and Chr6.

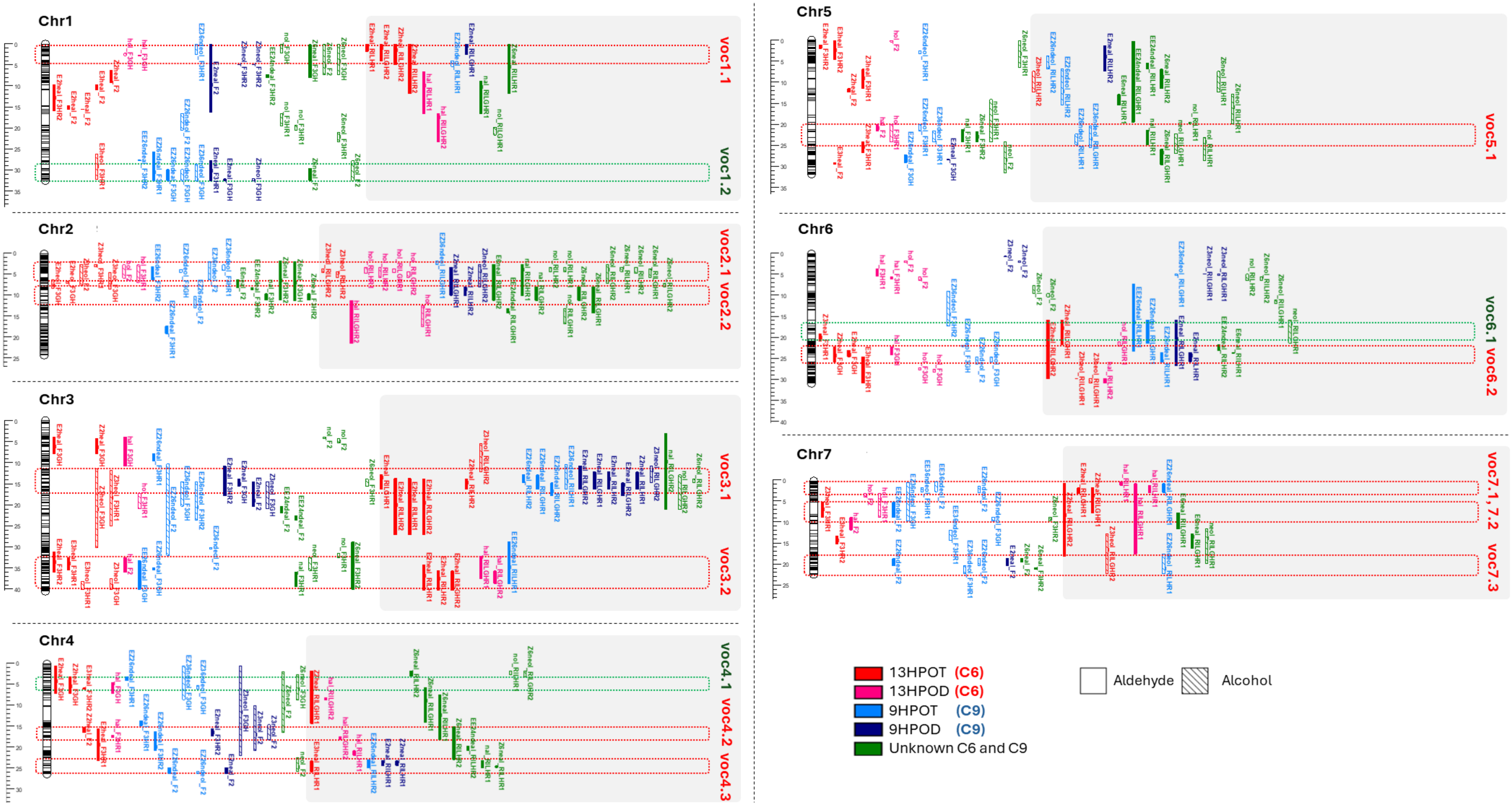

Figure S4 Distribution of univariate QTL for 24 C6 and C9 VOCs detected in WI7799 F_2, $F_{2:3}$ and RIL populations under the greenhouse (GH) and open-field (H) environments. For each chromosome, the scale to the left is 9930v3.0 physical distance. Warm- and cool-toned bars indicate QTL associated with C6 (from 13HPOD/13HPOT) and C9 (from 9HPOD/9HPOT) VOCs, respectively. Solid and hatched bars are QTL for aldehydes and alcohols, respectively. QTL in the gray and white backgrounds are identified with RIL, F_2/ $F_{2:3}$ populations, respectively. QTL in a dashed red line box belong to the same QTL cluster listed at the far right.

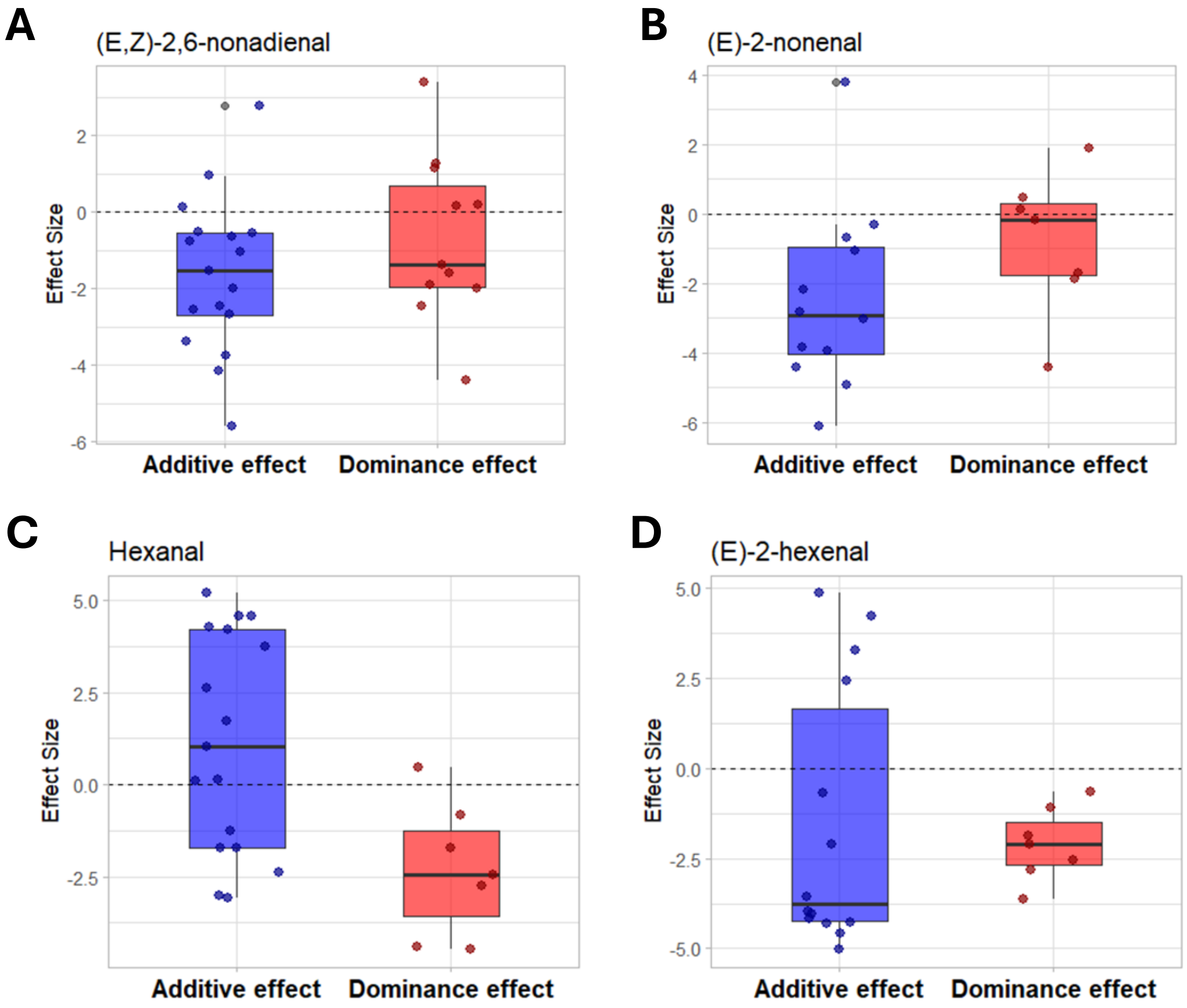

Figure S5 Box plots displaying the distribution of additive and dominant effects of all QTL identified for four major C_6 and C_9 aldehydes. Each dot represents the magnitude and direction of the additive or dominant effect of a single QTL. Note that the additive effects were obtained from all populations, while the dominance effects were from F_2 or $F_{2:3}$ populations only. WI7633 carries the reference alleles, while 9930 carries the alternative alleles.

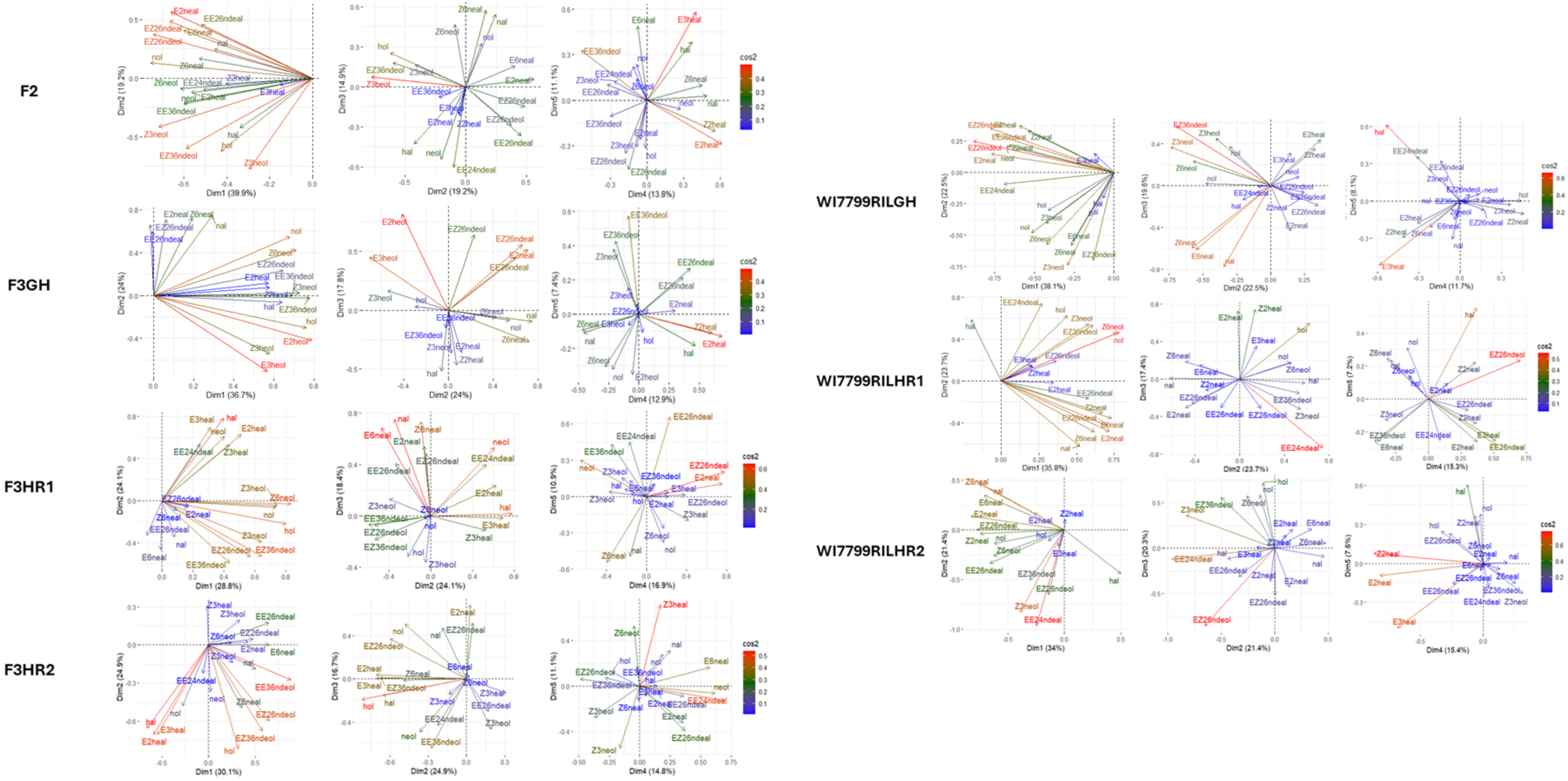

Figure S6 Biplots of the top five PCs (dimensions or Dim) from data of the WI7799 F_2, $F_{2:3}$ and RIL populations in two environments display the major patterns of multivariate VOC level variation and covariance among 24 VOCs. GH = greenhouse; H = open-field. R1/R2: Biological replications 1 and 2.

PC-QTLs mapped in voc2.1 QTL clusters

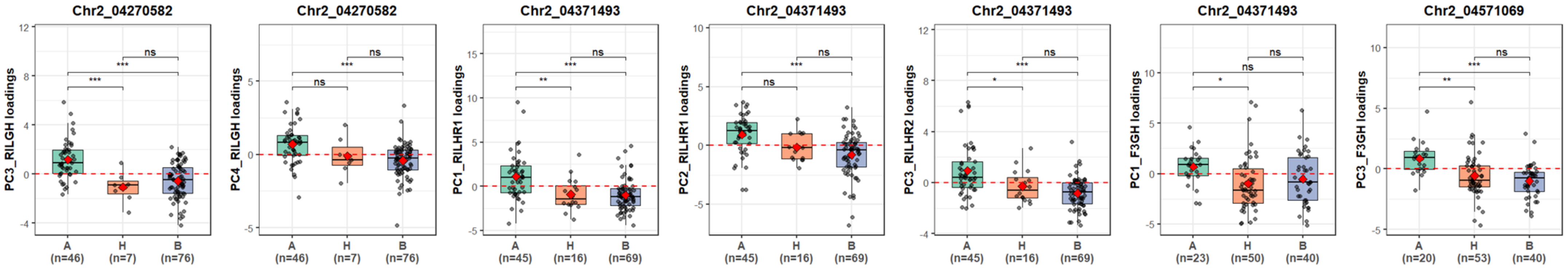

PC-QTLs mapped in voc3.1 QTL clusters

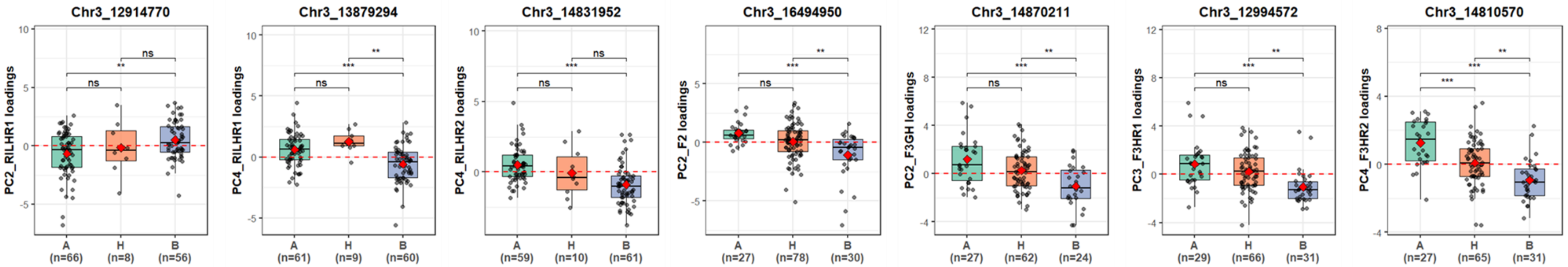

Figure S7 Allele effect plots of PC-QTL co-localized with voc2.1 and voc3.1. Each plot displays the distribution of PC scores by genotypes (A: homozygous WI7633; H: heterozygotes; B: homozygous 9930). Red dashed lines indicate zero; red diamonds indicate group means. Pairwise significance (Tukey HSD): ns, *p<0.05, **p<0.01, ***p<0.001.

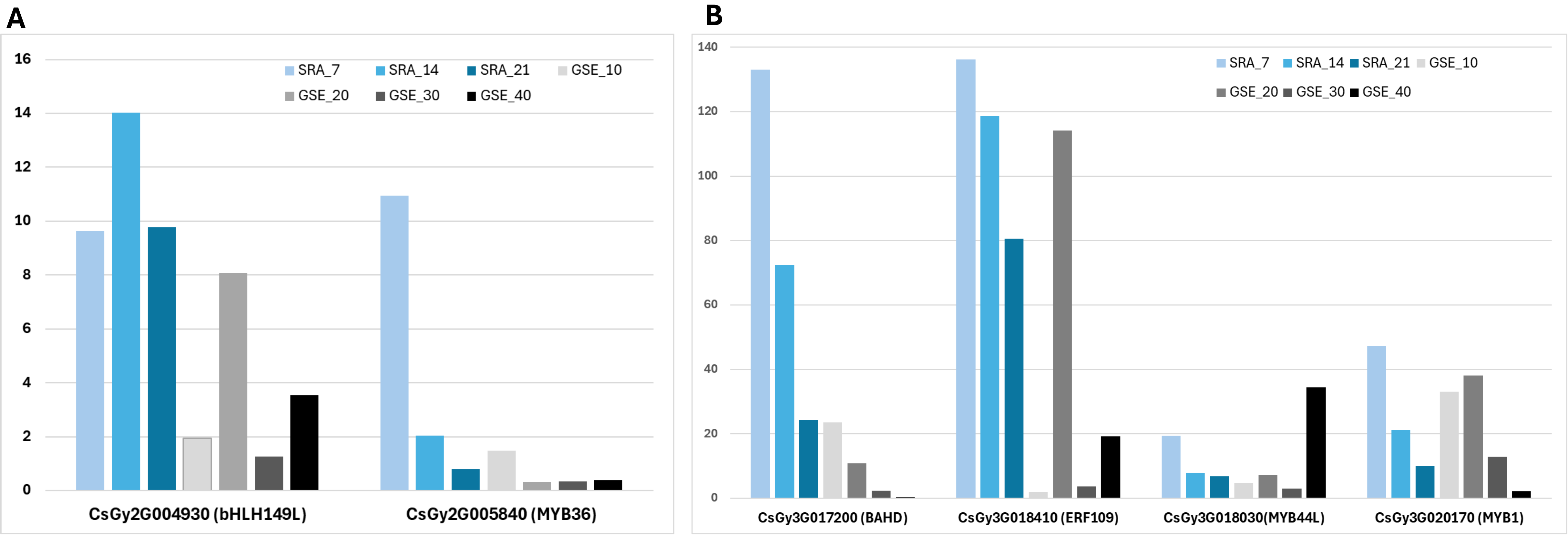

Figure S8 Expression level in cucumber fruit flesh at different days after anthesis of potential candidate genes for VOC QTL in voc2.1 **(A)** and voc3.1 **(B)** regions. Y-axis numbers are normalized gene expression value in FPKM (Fragments Per Kilobase of transcript per Million mapped reads). SRA and GSE are RNA-Seq data from NCBI accessions SRA071224 (Wei et al. 2016) and GSE116581 (Lu et al. 2018), respectively. Numbers after SRA or GSE are days after thesis of fruit flesh samples.
