# Supplementary material for: Univariate and multivariate QTL analyses provide insights into genetic architecture of variation of key volatile aroma compounds in cucumber (Cucumis sativus L.)
Source: Theor Appl Genet. 2026 Oct 1;139(10):288. doi: 10.1007/s00122-026-05393-5 (PMC13630885; doi:10.1007/s00122-026-05393-5)
Supplement: Supplementary file 2 [file 122_2026_5393_MOESM2_ESM.pdf]

Table S1. List of targeted volatile organic compounds (VOCs) in this study, and their aroma and flavor descriptions.

#	VOCs	Abbreviation	LOX branch	VOC category	Aroma/odor description	Flavor description	Reference[a]
1	**Hexanal**	hal	13HPOD	C6 aldehyde	green, fatty, leafy, vegetative, fruity and clean with a woody nuance	green, woody, vegetative, apple, grassy, citrus and orange with a fresh, lingering aftertaste	Burdock (2016)
2	**1-Hexanol**	hol	13HPOD	C6 alcohol	herbaceous, woody, fragrant, mild, sweet, green fruity	green, fruity, apple-skin, oily	Burdock (2016)
3	**(E)-2-Hexenal**	E2heal	13HPOT	C6 aldehyde	pungent green fatty, fresh fruity aldehydic, with fresh leafy apple and watermelon nuances	fruity, fresh green, herbal and vegetative, apple and melon with a slight yeasty nuance	Burdock (2016)
4	**(Z)-2-Hexenal**	Z2heal	13HPOT	C6 aldehyde	green	n/a	(Z)-2-hexenal, 16635-54-4
5	**(E)-2-Hexenol**	E2heol	13HPOT	C6 alcohol	fresh, fatty green, tomato vegetative, with leafy, green fruity and herbal nuances	fresh, leafy green, slightly fatty with grassy, fruity and juicy nuances, tomato and celery nuances	Burdock (2016)
6	**(E)-3-Hexen-1-ol**	E3heol	13HPOT	C6 alcohol	grassy green aroma	n/a	Burdock (2016)
7	**(Z)-3-Hexen-1-ol**	Z3heol	13HPOT	C6 alcohol	intense, grassy-green, characteristic herbaceous, leafy	fresh, green, raw fruity with a pungent depth	Burdock (2016)
8	**(E)-3-Hexenal**	E3heal	13HPOT	C6 aldehyde	green fruity apple	n/a	(E)-3-hexenal, 69112-21-6
9	**(Z)-3-Hexenal**	Z3heal	13HPOT	C6 aldehyde	sharp penetrating leafy green, vegetative stemmy with unripe tomato, melon and tropical nuances	strong, aromatic green, with waxy vegetative green melon and tomato nuances and a hint of cinnamon spice	Burdock (2016)
10	**(E)-2-Nonenal**	E2neal	9HPOD	C9 aldehyde	green, cucumber, aldehydic, fatty with a citrus nuance	green, soapy, cucumber/melon-like with an aldehydic, fatty nuance	Burdock (2016)
11	**(Z)-2-Nonenal**	Z2neal	9HPOD	C9 aldehyde	orris fatty waxy cucumber	n/a	(Z)-2-nonenal, 60784-31-8
12	**(Z)-3-Nonen-1-ol**	Z3neol	9HPOD	C9 alcohol	fresh waxy green melon rind tropical mushroom	waxy green spicy melon rind tropical cilantro fatty greasy	(Z)-3-nonen-1-ol, 10340-23-5
13	**(E,E)-2,6-Nonadienal**	EE26ndeal	9HPOT	C9 aldehyde	fresh citrus green cucumber melon	fresh, green cucumber skin and watermelon with a fatty nuance	Burdock (2016)
14	**(E,Z)-2,6-Nonadienal**	EZ26ndeal	9HPOT	C9 aldehyde	fresh, green cucumber	green, melon, cucumber-like with an aldehydic nuance	Burdock (2016)
15	**(E,Z)-2,6-Nonadienol**	EZ26ndeol	9HPOT	C9 alcohol	green cucumber vegetable odor	melon, watermelon, violet, mushroom-like with apple and rosy nuances	Burdock (2016)
16	**(E,Z)-3,6-Nonadien-1-ol**	EZ36ndeol	9HPOT	C9 alcohol	atty, raw vegetative-green cucumber and green pepper notes with fruity watermelon	oily, fatty raw cucumber vegetative character with green apple and unripe banana nuances	Burdock (2016)
17	**(E,E)-3,6-Nonadien-1-ol**	EE36ndeol	9HPOT	C9 alcohol	fresh green violet cucumber	n/a	3,6-nonadien-1-ol, 76649-25-7
18	**Nonanal**	nal	unknown	C9 aldehyde	sweet waxy, orange citrus with oily fatty and melon skin nuances and a slightly lactonic nuance	aldehydic citrus orange body with waxy and oily melon-like nuances	Burdock (2016)
19	**1-Nonanol**	nol	unknown	C9 alcohol	intensely waxy, sweet, green, citrus, orange-like,'reamy with fruity nuances of tamarind and melon	waxy, green, coconut, cheese, milky and creamy with citrus orange nuances	Burdock (2016)
20	**(E)-6-Nonenal**	E6neal	unknown	C9 aldehyde	n/a	n/a	(E)-6-nonenal, 2277-20-5
21	**(Z)-6-Nonenal**	Z6neal	unknown	C9 aldehyde	green, cucumber, cantaloupe, honeydew, melon	green cucumber and melon with a waxy vegetative nuance	Burdock (2016)
22	**(Z)-6-Nonenol**	Z6neol	unknown	C9 alcohol	powerful, melon, green odor.	waxy, cucumber, honeydew, green watermelon	Burdock (2016)
23	**(E,E)-2,4-Nonadienal**	EE24ndeal	unknown	C9 aldehyde	green, fatty melon, cucumber and vegetative	green, cucumber with fatty melon and chicken nuances	Burdock (2016)
24	**1-Nonen-4-ol**	neol	unknown	C9 alcohol	n/a	n/a	1-nonen-4-ol, 35192-73-5

[a]: https://www.thegoodscentscompany.com/odor/cucumber.html and Burdock GA (2016) Fenaroli's handbook of flavor ingredients. CRC Press.

Table S2. Summary of populations and experimental designs used for QTL mapping in WI7799 (9930×WI7633) F2, F2:3 and RIL populations.

#	Populations	Environments	Year and season of experiments	Experimental design [a]	Abbreviation
1	WI7799F2	WSGH, Madison WI, USA	Spring (Jan to Mar), 2021	135 F_2 plants, CRD	F2
2	WI7799F3	HARS, Hancock, WI, USA	Summer (Jun to Sep), 2021	135 F2:3 families, RCBD, 2 replicates	F3HR1, F3HR2
3	WI7799F3	WSGH, Madison WI, USA	Winter (Jan to Mar), 2021	135 F2:3 families, CRD	F3GH
4	WI7799RIL	HARS, Hancock, WI, USA	Summer (Jun to Sep), 2023	135 RILs, RCBD, 2 replicates	RILHR1, RILHR2
5	WI7799RIL	WSGH, Madison WI, USA	Winter (Jan to Mar), 2023	135 RILs, RCBD, 2 replicates	RILGHR1, RILGHR2

[a] CRD: completely randomized design; RCBD: randomized complete block design.

Table S3. Summary statistics of 20 C6 and C9 aldehydes and alcohols of founders and WI7799RIL population*

#	Names of VOCs	VOC category	Founders (BLUEs)			WI7799 RILGH		WI7799 RILH	
			9930	WI7633	WI7799F1	Mean±SD	Range (Min - Max)	Mean±SD	Range (Min - Max)
1	**1-Hexanol**	alcohol, C6	9,405,560	7,771,045	2,163,690	5236719 ± 4878225	300075 - 23741740	43135186 ± 43070499	148287 - 173366981
2	**Hexanal**	aldehyde, C6	210,865,431	120,129,991	138,238,953	166206830 ± 44008349	43778493 - 345812437	215363672 ± 53892331	95389036 - 359081653
3	**(Z)-3-Hexen-1-ol**	alcohol, C6	1,467,465	820,408	982,223	1376912 ± 1165919	76845 - 6693598	2142940 ± 1888620	205277 - 11495968
4	**(E)-2-Hexenal**	aldehyde, C6	52,282,448	66,670,148	46,416,417	36909253 ± 9902670	15835949 - 62860413	58581255 ± 19555312	13375015 - 123284975
5	**(E)-3-Hexenal**	aldehyde, C6	106,017	208,862	181,492	257082 ± 147728	16163 - 1022153	243428 ± 154481	29525 - 1034610
6	**(Z)-2-Hexenal**	aldehyde, C6	1,500,725	1,654,655	1,153,939	1198203 ± 234024	721634 - 1902151	1264827 ± 393439	369994 - 2759227
7	**(Z)-3-Nonen-1-ol**	alcohol, C9	8,458,417	1,500,181	2,563,117	1973267 ± 1259844	315289 - 8827214	5974959 ± 6464743	567972 - 42347083
8	**(E)-2-Nonenal**	aldehyde, C9	73,824,683	112,209,037	65,358,612	41553527 ± 12469553	17449645 - 90772471	78221317 ± 33389338	27636797 - 233463901
9	**(E,E)-3,6-Nonadien-1-ol**	alcohol, C9	8,071,369	1,432,796	1,244,416	889497 ± 460888	103599 - 2807131	2014680 ± 943052	733259 - 7702469
10	**(E,Z)-2,6-nonadienol**	alcohol, C9	3,355,414	3,618,854	2,800,785	2354695 ± 707392	1005804 - 5435476	3657258 ± 1110915	1569704 - 7580255
11	**(E,Z)-3,6-Nonadien-1-ol**	alcohol, C9	8,071,369	1,432,796	1,244,416	985699 ± 937755	48067 - 6797186	2941015 ± 3902611	133315 - 25106645
12	**(E,E)-2,6-nonadienal**	aldehyde, C9	2,195,639	6,629,425	3,347,541	1838767 ± 776315	578179 - 5147472	5169840 ± 2002112	1606426 - 11823764
13	**(E,Z)-2,6-nonadienal**	aldehyde, C9	100,694,134	146,379,010	88,825,762	93086472 ± 25616267	37259058 - 201525642	102702814 ± 29989123	35535791 - 214330891
14	**(Z)-6-Nonenol**	alcohol, C9	8,384,125	8,135,746	2,958,586	3903530 ± 4007857	334806 - 40199504	7264506 ± 7370850	374471 - 43624631
15	**1-Nonanol**	alcohol, C9	5,187,974	5,579,494	2,980,084	1847980 ± 2029153	26583 - 15582117	6086414 ± 6133495	224750 - 43245316
16	**1-Nonen-4-ol**	alcohol, C9	2,779,520	553,767	678,496	1130816 ± 825679	60275 - 4468590	-	-
17	**(E)-6-Nonenal**	aldehyde, C9	790,641	642,997	1,323,573	776025 ± 349107	130979 - 2587742	3350131 ± 2667868	151089 - 14347460
18	**(E,E)-2,4-Nonadienal**	aldehyde, C9	1,198,217	1,033,989	766,933	1078272 ± 464345	294183 - 2954991	3131705 ± 1439961	394387 - 12005841
19	**(Z)-6-Nonenal**	aldehyde, C9	45,145,184	47,207,320	35,617,339	49525376 ± 15831854	14965214 - 132884075	40426856 ± 22468103	8342940 - 131738578
20	**Nonanal**	aldehyde, C9	17,188,720	20,414,143	17,882,100	12373551 ± 4645045	4834818 - 30261140	23084337 ± 13540013	3928769 - 86156058
	**SUM/Mean**		560,982,982	554,024,662	416,728,471	21225123± 6535898		31829323± 13177729	

* GH: greenhouse (WSGH); H: open field (HARS)

Table S4. Phenotypic distribution characteristics (skewness, and coefficient of variation) of C6 and C9 VOCs in relative abundance.

#	VOC	Abbr.	VOC category	Population	Environment	BioRep	Mean ± SD	Coefficients of variation (CV)	Median	Skewness	p_value	Interpretation
1	1-Hexanol	hol	C6 alcohol	F2	GH		1.66 ± 1.36	82.40	1.17	1.81	0.000	Highly right-skewed
2	1-Hexanol	hol	C6 alcohol	F3	GH		1.17 ± 0.48	41.20	1.12	1.36	0.000	Highly right-skewed
3	1-Hexanol	hol	C6 alcohol	F3	H	R1	1.41 ± 1.17	83.05	0.98	1.67	0.000	Highly right-skewed
4	1-Hexanol	hol	C6 alcohol	F3	H	R2	1.18 ± 0.98	82.70	0.83	1.68	0.000	Highly right-skewed
5	1-Hexanol	hol	C6 alcohol	RIL	GH	R1	1.25 ± 1.17	93.02	0.68	1.26	0.000	Highly right-skewed
6	1-Hexanol	hol	C6 alcohol	RIL	GH	R2	1.32 ± 1.13	85.28	0.74	1.29	0.000	Highly right-skewed
7	1-Hexanol	hol	C6 alcohol	RIL	H	R1	5.14 ± 5.13	99.83	3.59	1.54	0.000	Highly right-skewed
8	1-Hexanol	hol	C6 alcohol	RIL	H	R2	5.61 ± 5.56	99.18	3.99	1.45	0.000	Highly right-skewed
9	(Z)-3-Hexen-1-ol	Z3heol	C6 alcohol	F2	GH		0.19 ± 0.11	61.45	0.15	1.42	0.000	Highly right-skewed
10	(Z)-3-Hexen-1-ol	Z3heol	C6 alcohol	F3	GH		0.16 ± 0.08	50.40	0.14	0.95	0.000	Moderately right-skewed
11	(Z)-3-Hexen-1-ol	Z3heol	C6 alcohol	F3	H	R1	0.11 ± 0.06	54.91	0.10	1.30	0.000	Highly right-skewed
12	(Z)-3-Hexen-1-ol	Z3heol	C6 alcohol	F3	H	R2	0.10 ± 0.06	56.54	0.08	1.72	0.000	Highly right-skewed
13	(Z)-3-Hexen-1-ol	Z3heol	C6 alcohol	RIL	GH	R1	0.29 ± 0.26	88.38	0.21	1.83	0.000	Highly right-skewed
14	(Z)-3-Hexen-1-ol	Z3heol	C6 alcohol	RIL	GH	R2	0.35 ± 0.27	75.67	0.29	1.57	0.000	Highly right-skewed
15	(Z)-3-Hexen-1-ol	Z3heol	C6 alcohol	RIL	H	R1	0.29 ± 0.14	47.69	0.27	1.01	0.000	Highly right-skewed
16	(Z)-3-Hexen-1-ol	Z3heol	C6 alcohol	RIL	H	R2	0.31 ± 0.16	50.72	0.27	1.44	0.000	Highly right-skewed
17	(E)-2-Hexenal	E2heal	C6 aldehyde	F2	GH		8.17 ± 2.04	24.95	7.96	0.44	0.020	Normal (approximately symmetric)
18	(E)-2-Hexenal	E2heal	C6 aldehyde	F3	GH		13.65 ± 3.28	24.00	13.33	0.69	0.003	Moderately right-skewed
19	(E)-2-Hexenal	E2heal	C6 aldehyde	F3	H	R1	8.29 ± 2.19	26.39	7.99	0.86	0.000	Moderately right-skewed
20	(E)-2-Hexenal	E2heal	C6 aldehyde	F3	H	R2	10.64 ± 4.10	38.53	9.80	0.55	0.010	Moderately right-skewed
21	(E)-2-Hexenal	E2heal	C6 aldehyde	RIL	GH	R1	8.59 ± 2.31	26.88	8.41	0.19	0.357	Not significant
22	(E)-2-Hexenal	E2heal	C6 aldehyde	RIL	GH	R2	9.08 ± 2.23	24.59	9.01	0.09	0.664	Not significant
23	(E)-2-Hexenal	E2heal	C6 aldehyde	RIL	H	R1	9.79 ± 3.15	32.15	9.55	0.51	0.015	Moderately right-skewed
24	(E)-2-Hexenal	E2heal	C6 aldehyde	RIL	H	R2	10.21 ± 3.12	30.59	9.55	0.79	0.000	Moderately right-skewed
25	Hexanal	hal	C6 aldehyde	F2	GH		29.16 ± 8.14	27.94	28.62	0.45	0.017	Normal (approximately symmetric)
26	Hexanal	hal	C6 aldehyde	F3	GH		18.60 ± 5.10	27.45	18.72	0.47	0.034	Normal (approximately symmetric)
27	Hexanal	hal	C6 aldehyde	F3	H	R1	12.34 ± 4.05	32.79	11.66	0.50	0.019	Normal (approximately symmetric)
28	Hexanal	hal	C6 aldehyde	F3	H	R2	15.20 ± 6.14	40.39	14.69	0.50	0.020	Normal (approximately symmetric)
29	Hexanal	hal	C6 aldehyde	RIL	GH	R1	39.36 ± 7.62	19.36	39.51	-0.35	0.094	Not significant
30	Hexanal	hal	C6 aldehyde	RIL	GH	R2	39.00 ± 7.90	20.25	39.64	-0.38	0.078	Not significant
31	Hexanal	hal	C6 aldehyde	RIL	H	R1	37.50 ± 9.50	25.34	35.96	0.37	0.075	Not significant
32	Hexanal	hal	C6 aldehyde	RIL	H	R2	37.03 ± 10.60	28.64	36.82	0.11	0.570	Not significant
33	(Z)-2-Hexenal	Z2heal	C6 aldehyde	F2	GH		0.21 ± 0.06	28.17	0.21	0.46	0.016	Normal (approximately symmetric)
34	(Z)-2-Hexenal	Z2heal	C6 aldehyde	F3	GH		0.36 ± 0.12	34.40	0.34	0.49	0.026	Normal (approximately symmetric)
35	(Z)-2-Hexenal	Z2heal	C6 aldehyde	F3	H	R1	0.22 ± 0.06	24.70	0.21	1.05	0.000	Highly right-skewed
36	(Z)-2-Hexenal	Z2heal	C6 aldehyde	F3	H	R2	0.26 ± 0.08	31.53	0.24	0.61	0.005	Moderately right-skewed
37	(Z)-2-Hexenal	Z2heal	C6 aldehyde	RIL	GH	R1	0.28 ± 0.06	22.93	0.27	0.71	0.001	Moderately right-skewed
38	(Z)-2-Hexenal	Z2heal	C6 aldehyde	RIL	GH	R2	0.30 ± 0.06	20.69	0.29	0.34	0.110	Not significant
39	(Z)-2-Hexenal	Z2heal	C6 aldehyde	RIL	H	R1	0.21 ± 0.06	28.13	0.20	0.48	0.021	Normal (approximately symmetric)
40	(Z)-2-Hexenal	Z2heal	C6 aldehyde	RIL	H	R2	0.22 ± 0.07	29.72	0.21	0.68	0.002	Moderately right-skewed
41	(E)-3-Hexenal	E3heal	C6 aldehyde	F2	GH		0.03 ± 0.02	68.59	0.02	2.00	0.000	Highly right-skewed
42	(E)-3-Hexenal	E3heal	C6 aldehyde	F3	GH		0.04 ± 0.01	22.01	0.04	0.00	0.995	Not significant
43	(E)-3-Hexenal	E3heal	C6 aldehyde	F3	H	R1	0.03 ± 0.02	48.34	0.03	1.03	0.000	Highly right-skewed
44	(E)-3-Hexenal	E3heal	C6 aldehyde	F3	H	R2	0.03 ± 0.02	48.08	0.03	0.31	0.130	Not significant
45	(E)-3-Hexenal	E3heal	C6 aldehyde	RIL	GH	R1	0.05 ± 0.03	54.15	0.05	0.57	0.008	Moderately right-skewed
46	(E)-3-Hexenal	E3heal	C6 aldehyde	RIL	GH	R2	0.07 ± 0.03	52.69	0.06	1.12	0.000	Highly right-skewed
47	(E)-3-Hexenal	E3heal	C6 aldehyde	RIL	H	R1	0.04 ± 0.02	51.04	0.04	0.86	0.000	Moderately right-skewed
48	(E)-3-Hexenal	E3heal	C6 aldehyde	RIL	H	R2	0.04 ± 0.02	49.60	0.04	0.96	0.000	Moderately right-skewed
49	(E,E)-3,6-Nonadien-1-ol	EE36ndeol	C9 alcohol	F2	GH		0.04 ± 0.03	85.16	0.03	2.64	0.000	Highly right-skewed

50	(E,E)-3,6-Nonadien-1-ol	EE36ndeol	C9 alcohol	F3	GH		0.02 ± 0.01	57.70	0.02	2.39	0.000	Highly right-skewed
51	(E,E)-3,6-Nonadien-1-ol	EE36ndeol	C9 alcohol	F3	H	R1	0.09 ± 0.05	58.13	0.08	1.09	0.000	Highly right-skewed
52	(E,E)-3,6-Nonadien-1-ol	EE36ndeol	C9 alcohol	F3	H	R2	0.05 ± 0.04	80.73	0.03	2.26	0.000	Highly right-skewed
53	(E,E)-3,6-Nonadien-1-ol	EE36ndeol	C9 alcohol	RIL	GH	R1	0.02 ± 0.01	36.02	0.02	1.21	0.000	Highly right-skewed
54	(E,E)-3,6-Nonadien-1-ol	EE36ndeol	C9 alcohol	RIL	GH	R2	0.03 ± 0.01	39.39	0.02	2.04	0.000	Highly right-skewed
55	(E,E)-3,6-Nonadien-1-ol	EE36ndeol	C9 alcohol	RIL	H	R1	0.05 ± 0.02	38.06	0.05	0.40	0.054	Not significant
56	(E,E)-3,6-Nonadien-1-ol	EE36ndeol	C9 alcohol	RIL	H	R2	0.05 ± 0.02	40.21	0.04	0.57	0.007	Moderately right-skewed
57	(E,Z)-2,6-Nonadienol	EZ26ndeol	C9 alcohol	F2	GH		0.85 ± 0.19	22.22	0.86	0.06	0.758	Not significant
58	(E,Z)-2,6-Nonadienol	EZ26ndeol	C9 alcohol	F3	GH		0.16 ± 0.10	62.10	0.15	1.35	0.000	Highly right-skewed
59	(E,Z)-2,6-Nonadienol	EZ26ndeol	C9 alcohol	F3	H	R1	0.30 ± 0.25	84.19	0.27	1.96	0.000	Highly right-skewed
60	(E,Z)-2,6-Nonadienol	EZ26ndeol	C9 alcohol	F3	H	R2	0.37 ± 0.25	66.37	0.33	1.29	0.000	Highly right-skewed
61	(E,Z)-2,6-Nonadienol	EZ26ndeol	C9 alcohol	RIL	GH	R1	0.55 ± 0.14	24.81	0.55	1.37	0.000	Highly right-skewed
62	(E,Z)-2,6-Nonadienol	EZ26ndeol	C9 alcohol	RIL	GH	R2	0.56 ± 0.13	22.56	0.54	1.02	0.000	Highly right-skewed
63	(E,Z)-2,6-Nonadienol	EZ26ndeol	C9 alcohol	RIL	H	R1	0.59 ± 0.15	25.59	0.57	1.08	0.000	Highly right-skewed
64	(E,Z)-2,6-Nonadienol	EZ26ndeol	C9 alcohol	RIL	H	R2	0.63 ± 0.16	26.16	0.61	0.69	0.001	Moderately right-skewed
65	(E,Z)-3,6-Nonadien-1-ol	EZ36ndeol	C9 alcohol	F2	GH		0.51 ± 0.39	76.08	0.41	2.28	0.000	Highly right-skewed
66	(E,Z)-3,6-Nonadien-1-ol	EZ36ndeol	C9 alcohol	F3	GH		0.67 ± 0.34	51.15	0.57	0.97	0.000	Moderately right-skewed
67	(E,Z)-3,6-Nonadien-1-ol	EZ36ndeol	C9 alcohol	F3	H	R1	0.86 ± 0.47	54.61	0.78	1.12	0.000	Highly right-skewed
68	(E,Z)-3,6-Nonadien-1-ol	EZ36ndeol	C9 alcohol	F3	H	R2	0.52 ± 0.34	66.12	0.43	1.55	0.000	Highly right-skewed
69	(E,Z)-3,6-Nonadien-1-ol	EZ36ndeol	C9 alcohol	RIL	GH	R1	0.21 ± 0.20	93.24	0.14	1.88	0.000	Highly right-skewed
70	(E,Z)-3,6-Nonadien-1-ol	EZ36ndeol	C9 alcohol	RIL	GH	R2	0.25 ± 0.19	76.80	0.20	1.24	0.000	Highly right-skewed
71	(E,Z)-3,6-Nonadien-1-ol	EZ36ndeol	C9 alcohol	RIL	H	R1	0.35 ± 0.32	91.64	0.23	2.00	0.000	Highly right-skewed
72	(E,Z)-3,6-Nonadien-1-ol	EZ36ndeol	C9 alcohol	RIL	H	R2	0.35 ± 0.34	97.02	0.24	2.25	0.000	Highly right-skewed
73	1-Nonen-4-ol	neol	C9 alcohol	F2	GH		0.28 ± 0.15	52.63	0.24	1.14	0.000	Highly right-skewed
74	1-Nonen-4-ol	neol	C9 alcohol	F3	GH		0.35 ± 0.06	17.79	0.34	0.60	0.008	Moderately right-skewed
75	1-Nonen-4-ol	neol	C9 alcohol	F3	H	R1	0.33 ± 0.13	40.68	0.31	0.80	0.000	Moderately right-skewed
76	1-Nonen-4-ol	neol	C9 alcohol	F3	H	R2	0.31 ± 0.13	42.09	0.29	0.68	0.002	Moderately right-skewed
77	1-Nonen-4-ol	neol	C9 alcohol	RIL	GH	R1	0.25 ± 0.19	72.87	0.23	1.72	0.000	Highly right-skewed
78	1-Nonen-4-ol	neol	C9 alcohol	RIL	GH	R2	0.27 ± 0.19	69.95	0.23	1.72	0.000	Highly right-skewed
79	1-Nonen-4-ol	neol	C9 alcohol	RIL	H	R1	0.35 ± 0.05	15.12	0.34	0.26	0.198	Not significant
80	1-Nonen-4-ol	neol	C9 alcohol	RIL	H	R2	0.36 ± 0.05	13.56	0.36	0.29	0.149	Not significant
81	1-Nonanol	nol	C9 alcohol	F2	GH		1.10 ± 0.47	42.61	0.99	1.41	0.000	Highly right-skewed
82	1-Nonanol	nol	C9 alcohol	F3	GH		1.40 ± 0.54	39.00	1.25	0.59	0.008	Moderately right-skewed
83	1-Nonanol	nol	C9 alcohol	F3	H	R1	1.76 ± 0.71	40.40	1.60	1.11	0.000	Highly right-skewed
84	1-Nonanol	nol	C9 alcohol	F3	H	R2	1.55 ± 0.89	57.35	1.35	0.82	0.000	Moderately right-skewed
85	1-Nonanol	nol	C9 alcohol	RIL	GH	R1	0.41 ± 0.34	83.23	0.30	1.57	0.000	Highly right-skewed
86	1-Nonanol	nol	C9 alcohol	RIL	GH	R2	0.42 ± 0.36	86.62	0.27	1.54	0.000	Highly right-skewed
87	1-Nonanol	nol	C9 alcohol	RIL	H	R1	0.88 ± 0.65	73.19	0.70	1.19	0.000	Highly right-skewed
88	1-Nonanol	nol	C9 alcohol	RIL	H	R2	0.85 ± 0.65	76.53	0.61	1.29	0.000	Highly right-skewed
89	(Z)-3-Nonen-1-ol	Z3neol	C9 alcohol	F2	GH		0.70 ± 0.40	56.78	0.60	2.23	0.000	Highly right-skewed
90	(Z)-3-Nonen-1-ol	Z3neol	C9 alcohol	F3	GH		0.68 ± 0.32	47.12	0.60	0.82	0.000	Moderately right-skewed
91	(Z)-3-Nonen-1-ol	Z3neol	C9 alcohol	F3	H	R1	1.44 ± 0.65	45.06	1.26	0.83	0.000	Moderately right-skewed
92	(Z)-3-Nonen-1-ol	Z3neol	C9 alcohol	F3	H	R2	0.86 ± 0.46	54.12	0.74	1.29	0.000	Highly right-skewed
93	(Z)-3-Nonen-1-ol	Z3neol	C9 alcohol	RIL	GH	R1	0.45 ± 0.28	61.76	0.42	0.86	0.000	Moderately right-skewed
94	(Z)-3-Nonen-1-ol	Z3neol	C9 alcohol	RIL	GH	R2	0.48 ± 0.28	57.61	0.40	1.19	0.000	Highly right-skewed
95	(Z)-3-Nonen-1-ol	Z3neol	C9 alcohol	RIL	H	R1	0.91 ± 0.66	72.19	0.76	2.37	0.000	Highly right-skewed
96	(Z)-3-Nonen-1-ol	Z3neol	C9 alcohol	RIL	H	R2	0.86 ± 0.54	62.61	0.76	1.60	0.000	Highly right-skewed
97	(Z)-6-Nonenol	Z6neol	C9 alcohol	F2	GH		1.17 ± 0.86	73.20	0.89	1.50	0.000	Highly right-skewed
98	(Z)-6-Nonenol	Z6neol	C9 alcohol	F3	GH		2.64 ± 1.02	38.56	2.50	0.59	0.008	Moderately right-skewed
99	(Z)-6-Nonenol	Z6neol	C9 alcohol	F3	H	R1	1.55 ± 0.79	50.72	1.42	0.84	0.000	Moderately right-skewed
100	(Z)-6-Nonenol	Z6neol	C9 alcohol	F3	H	R2	1.59 ± 1.03	65.05	1.23	1.24	0.000	Highly right-skewed
101	(Z)-6-Nonenol	Z6neol	C9 alcohol	RIL	GH	R1	0.84 ± 0.59	70.05	0.67	1.29	0.000	Highly right-skewed

102	(Z)-6-Nonenol	Z6neol	C9 alcohol	RIL	GH	R2	0.87 ± 0.66	75.92	0.65	1.90	0.000	Highly right-skewed
103	(Z)-6-Nonenol	Z6neol	C9 alcohol	RIL	H	R1	1.09 ± 0.85	78.23	0.74	1.41	0.000	Highly right-skewed
104	(Z)-6-Nonenol	Z6neol	C9 alcohol	RIL	H	R2	1.01 ± 0.88	87.10	0.70	1.58	0.000	Highly right-skewed
105	(E)-2-Nonenal	E2neal	C9 aldehyde	F2	GH		16.98 ± 4.64	27.34	17.19	-0.07	0.696	Not significant
106	(E)-2-Nonenal	E2neal	C9 aldehyde	F3	GH		20.43 ± 3.79	18.56	20.48	-0.35	0.102	Not significant
107	(E)-2-Nonenal	E2neal	C9 aldehyde	F3	H	R1	25.36 ± 4.04	15.93	25.77	-0.05	0.809	Not significant
108	(E)-2-Nonenal	E2neal	C9 aldehyde	F3	H	R2	24.89 ± 5.56	22.36	24.80	0.15	0.465	Not significant
109	(E)-2-Nonenal	E2neal	C9 aldehyde	RIL	GH	R1	9.89 ± 2.14	21.62	9.75	0.60	0.006	Moderately right-skewed
110	(E)-2-Nonenal	E2neal	C9 aldehyde	RIL	GH	R2	9.63 ± 2.17	22.50	9.46	0.74	0.001	Moderately right-skewed
111	(E)-2-Nonenal	E2neal	C9 aldehyde	RIL	H	R1	13.17 ± 4.23	32.11	12.81	0.35	0.091	Not significant
112	(E)-2-Nonenal	E2neal	C9 aldehyde	RIL	H	R2	12.90 ± 4.24	32.85	12.10	0.50	0.016	Moderately right-skewed
113	(E)-6-Nonenal	E6neal	C9 aldehyde	F2	GH		0.40 ± 0.17	43.28	0.34	1.36	0.000	Highly right-skewed
114	(E)-6-Nonenal	E6neal	C9 aldehyde	F3	GH		0.28 ± 0.04	14.58	0.28	0.61	0.007	Moderately right-skewed
115	(E)-6-Nonenal	E6neal	C9 aldehyde	F3	H	R1	0.51 ± 0.15	28.99	0.49	0.69	0.002	Moderately right-skewed
116	(E)-6-Nonenal	E6neal	C9 aldehyde	F3	H	R2	0.34 ± 0.13	37.60	0.33	1.24	0.000	Highly right-skewed
117	(E)-6-Nonenal	E6neal	C9 aldehyde	RIL	GH	R1	0.19 ± 0.08	44.36	0.17	1.52	0.000	Highly right-skewed
118	(E)-6-Nonenal	E6neal	C9 aldehyde	RIL	GH	R2	0.18 ± 0.08	44.65	0.16	3.27	0.000	Highly right-skewed
119	(E)-6-Nonenal	E6neal	C9 aldehyde	RIL	H	R1	0.50 ± 0.34	67.84	0.44	2.33	0.000	Highly right-skewed
120	(E)-6-Nonenal	E6neal	C9 aldehyde	RIL	H	R2	0.45 ± 0.29	64.83	0.38	1.05	0.000	Highly right-skewed
121	(E,E)-2,4-Nonadienal	EE24ndeal	C9 aldehyde	F2	GH		0.31 ± 0.11	34.04	0.30	0.67	0.001	Moderately right-skewed
122	(E,E)-2,4-Nonadienal	EE24ndeal	C9 aldehyde	F3	GH		0.27 ± 0.05	18.29	0.26	0.82	0.000	Moderately right-skewed
123	(E,E)-2,4-Nonadienal	EE24ndeal	C9 aldehyde	F3	H	R1	0.26 ± 0.07	26.22	0.26	0.61	0.005	Moderately right-skewed
124	(E,E)-2,4-Nonadienal	EE24ndeal	C9 aldehyde	F3	H	R2	0.25 ± 0.07	26.49	0.24	1.10	0.000	Highly right-skewed
125	(E,E)-2,4-Nonadienal	EE24ndeal	C9 aldehyde	RIL	GH	R1	0.25 ± 0.09	35.87	0.25	0.72	0.001	Moderately right-skewed
126	(E,E)-2,4-Nonadienal	EE24ndeal	C9 aldehyde	RIL	GH	R2	0.25 ± 0.09	34.53	0.24	1.18	0.000	Highly right-skewed
127	(E,E)-2,4-Nonadienal	EE24ndeal	C9 aldehyde	RIL	H	R1	0.48 ± 0.19	39.97	0.44	0.53	0.012	Moderately right-skewed
128	(E,E)-2,4-Nonadienal	EE24ndeal	C9 aldehyde	RIL	H	R2	0.55 ± 0.21	38.15	0.49	1.21	0.000	Highly right-skewed
129	(E,E)-2,6-Nonadienal	EE26ndeal	C9 aldehyde	F2	GH		1.12 ± 0.65	58.43	0.94	1.23	0.000	Highly right-skewed
130	(E,E)-2,6-Nonadienal	EE26ndeal	C9 aldehyde	F3	GH		1.32 ± 0.49	36.92	1.30	0.40	0.069	Not significant
131	(E,E)-2,6-Nonadienal	EE26ndeal	C9 aldehyde	F3	H	R1	2.08 ± 0.76	36.27	2.05	0.15	0.447	Not significant
132	(E,E)-2,6-Nonadienal	EE26ndeal	C9 aldehyde	F3	H	R2	1.55 ± 0.95	61.27	1.25	1.04	0.000	Highly right-skewed
133	(E,E)-2,6-Nonadienal	EE26ndeal	C9 aldehyde	RIL	GH	R1	0.44 ± 0.12	27.92	0.42	0.77	0.001	Moderately right-skewed
134	(E,E)-2,6-Nonadienal	EE26ndeal	C9 aldehyde	RIL	GH	R2	0.42 ± 0.17	40.17	0.38	3.84	0.000	Highly right-skewed
135	(E,E)-2,6-Nonadienal	EE26ndeal	C9 aldehyde	RIL	H	R1	0.83 ± 0.28	33.28	0.77	0.67	0.002	Moderately right-skewed
136	(E,E)-2,6-Nonadienal	EE26ndeal	C9 aldehyde	RIL	H	R2	0.92 ± 0.30	33.16	0.90	0.84	0.000	Moderately right-skewed
137	(E,Z)-2,6-Nonadienal	EZ26ndeal	C9 aldehyde	F2	GH		25.58 ± 6.32	24.71	25.83	0.05	0.792	Not significant
138	(E,Z)-2,6-Nonadienal	EZ26ndeal	C9 aldehyde	F3	GH		34.70 ± 5.76	16.60	34.85	0.07	0.727	Not significant
139	(E,Z)-2,6-Nonadienal	EZ26ndeal	C9 aldehyde	F3	H	R1	35.28 ± 4.07	11.54	35.20	-0.05	0.800	Not significant
140	(E,Z)-2,6-Nonadienal	EZ26ndeal	C9 aldehyde	F3	H	R2	34.62 ± 5.91	17.08	35.03	-0.31	0.136	Not significant
141	(E,Z)-2,6-Nonadienal	EZ26ndeal	C9 aldehyde	RIL	GH	R1	21.96 ± 5.21	23.73	21.70	0.99	0.000	Moderately right-skewed
142	(E,Z)-2,6-Nonadienal	EZ26ndeal	C9 aldehyde	RIL	GH	R2	22.07 ± 4.84	21.91	21.76	0.36	0.092	Not significant
143	(E,Z)-2,6-Nonadienal	EZ26ndeal	C9 aldehyde	RIL	H	R1	17.10 ± 3.86	22.59	16.97	-0.02	0.920	Not significant
144	(E,Z)-2,6-Nonadienal	EZ26ndeal	C9 aldehyde	RIL	H	R2	17.67 ± 4.07	23.00	17.71	-0.02	0.900	Not significant
145	Nonanal	nal	C9 aldehyde	F2	GH		2.75 ± 1.33	48.29	2.41	0.75	0.000	Moderately right-skewed
146	Nonanal	nal	C9 aldehyde	F3	GH		0.80 ± 0.37	46.43	0.75	1.12	0.000	Highly right-skewed
147	Nonanal	nal	C9 aldehyde	F3	H	R1	2.76 ± 1.23	44.64	2.60	0.73	0.001	Moderately right-skewed
148	Nonanal	nal	C9 aldehyde	F3	H	R2	1.77 ± 0.93	52.80	1.56	1.27	0.000	Highly right-skewed
149	Nonanal	nal	C9 aldehyde	RIL	GH	R1	2.97 ± 1.02	34.28	2.91	0.53	0.013	Moderately right-skewed
150	Nonanal	nal	C9 aldehyde	RIL	GH	R2	2.89 ± 0.99	34.31	2.65	0.86	0.000	Moderately right-skewed
151	Nonanal	nal	C9 aldehyde	RIL	H	R1	3.89 ± 1.78	45.71	3.53	0.62	0.004	Moderately right-skewed
152	Nonanal	nal	C9 aldehyde	RIL	H	R2	3.62 ± 1.65	45.61	3.40	0.70	0.001	Moderately right-skewed
153	(Z)-6-Nonenal	Z6neal	C9 aldehyde	F2	GH		8.80 ± 3.51	39.91	8.25	0.89	0.000	Moderately right-skewed

154	(Z)-6-Nonenal	Z6neal	C9 aldehyde	F3	GH		2.31 ± 1.12	48.44	2.02	0.91	0.000	Moderately right-skewed
155	(Z)-6-Nonenal	Z6neal	C9 aldehyde	F3	H	R1	5.01 ± 2.17	43.36	4.66	0.74	0.001	Moderately right-skewed
156	(Z)-6-Nonenal	Z6neal	C9 aldehyde	F3	H	R2	3.92 ± 1.91	48.57	3.55	1.42	0.000	Highly right-skewed
157	(Z)-6-Nonenal	Z6neal	C9 aldehyde	RIL	GH	R1	11.73 ± 3.37	28.71	10.99	0.93	0.000	Moderately right-skewed
158	(Z)-6-Nonenal	Z6neal	C9 aldehyde	RIL	GH	R2	11.58 ± 2.92	25.20	11.06	0.78	0.001	Moderately right-skewed
159	(Z)-6-Nonenal	Z6neal	C9 aldehyde	RIL	H	R1	6.85 ± 2.95	43.09	6.57	0.67	0.002	Moderately right-skewed
160	(Z)-6-Nonenal	Z6neal	C9 aldehyde	RIL	H	R2	6.36 ± 3.00	47.24	5.78	0.88	0.000	Moderately right-skewed

Table S5. Main statistics of linkage maps developed in WI7799 F2 and RIL populations.

	**# Mapped loci**		**Map Length (cM)**		**Physical coverage (bp, 9930v3.0)**		**Marker density (# loci/Mb)**		**Recombination rate (cM/Mb)**	
**Chr**	**F2**	**RIL**	**F2**	**RIL**	**F2**	**RIL**	**F2**	**RIL**	**F2**	**RIL**
**1**	231	43	145.63	133.07	32,509,306	32,504,553	7.11	1.32	4.48	4.09
**2**	257	34	149.98	118.64	24,333,780	23,910,448	10.56	1.42	6.16	4.96
**3**	443	58	211.12	157.73	37,759,086	39,443,369	11.73	1.47	5.59	4.00
**4**	213	59	142.39	151.2	26,249,184	25,849,536	8.11	2.28	5.42	5.85
**5**	254	66	142.88	133.1	31,648,555	31,257,896	8.03	2.11	4.51	4.26
**6**	340	44	182.22	135.88	30,341,511	30,850,320	11.21	1.43	6.01	4.40
**7**	174	30	102.38	118.34	22,274,909	22,314,692	7.81	1.34	4.60	5.30
**Sum/mean**	**1912**	**334**	**1076.6**	**947.96**	**205,116,331**	**206,130,814**	**9.22**	**1.63**	**5.25**	**4.70**

Table S6. Mapped SNP loci on the WI7799 F2 linkage map and identified segregation distortion regions.

#	Marker	Chr	Physical position (9930v3.0)	Genetic distance (cM)	chi-square value	p-value
1	1_142952	1	142,952	2.59	0.06	0.97
2	1_143483	1	143,483	1.23	0.40	0.82
3	1_143552	1	143,552	1.23	0.41	0.81
4	1_143761	1	143,761	2.21	0.29	0.87
5	1_148606	1	148,606	2.88	0.19	0.91
6	1_148651	1	148,651	2.63	0.19	0.91
7	1_148977	1	148,977	0	1.46	0.48
8	1_694853	1	694,853	4.28	0.13	0.94
9	1_709317	1	709,317	4.18	0.13	0.94
10	1_777241	1	777,241	4.16	0.13	0.94
11	1_777853	1	777,853	4.65	0.06	0.97
12	1_777880	1	777,880	4.65	0.06	0.97
13	1_777929	1	777,929	4.71	0.06	0.97
14	1_800582	1	800,582	5.42	0.13	0.94
15	1_802262	1	802,262	4.65	0.12	0.94
16	1_802338	1	802,338	6.09	0.41	0.81
17	1_815217	1	815,217	5.82	0.13	0.94
18	1_815556	1	815,556	5.9	0.23	0.89
19	1_828019	1	828,019	7.21	0.69	0.71
20	1_828033	1	828,033	7.28	0.69	0.71
21	1_1096621	1	1,096,621	6.62	0.51	0.77
22	1_1447411	1	1,447,411	8.9	0.57	0.75
23	1_1447853	1	1,447,853	8.46	0.36	0.84
24	1_1450884	1	1,450,884	9.53	1.46	0.48
25	1_1450950	1	1,450,950	9.53	1.46	0.48
26	1_1451102	1	1,451,102	9.87	0.52	0.77
27	1_1709026	1	1,709,026	10.54	0.57	0.75
28	1_2190158	1	2,190,158	12.95	0.41	0.81
29	1_2203172	1	2,203,172	11.19	0.51	0.77
30	1_2204761	1	2,204,761	12.35	0.23	0.89
31	1_2206723	1	2,206,723	12.11	0.36	0.84
32	1_2207914	1	2,207,914	12.51	0.23	0.89
33	1_2216937	1	2,216,937	11.84	0.36	0.84
34	1_2625328	1	2,625,328	15.95	0.69	0.71
35	1_2745981	1	2,745,981	17.82	1.53	0.46
36	1_2765658	1	2,765,658	17.02	1.07	0.59
37	1_2779727	1	2,779,727	17.2	1.07	0.59
38	1_2789870	1	2,789,870	17.66	1.69	0.43
39	1_2795389	1	2,795,389	16.52	1.20	0.55
40	1_2795403	1	2,795,403	16.67	1.20	0.55
41	1_2863804	1	2,863,804	18.38	1.92	0.38
42	1_2863837	1	2,863,837	18.61	2.65	0.27
43	1_2871991	1	2,871,991	16.86	1.07	0.59
44	1_2881708	1	2,881,708	19.46	3.83	0.15
45	1_2881906	1	2,881,906	19.46	3.83	0.15

46	1_3888021	1	3,888,021	24.57	6.38	0.04
47	1_4049144	1	4,049,144	25.99	3.59	0.17
48	1_4294570	1	4,294,570	28.02	5.78	0.06
49	1_4294579	1	4,294,579	27.71	5.78	0.06
50	1_4324129	1	4,324,129	27.29	6.38	0.04
51	1_4363620	1	4,363,620	28.89	6.10	0.05
52	1_4651504	1	4,651,504	30.56	5.78	0.06
53	1_4934549	1	4,934,549	35.58	6.10	0.05
54	1_4937738	1	4,937,738	34.74	7.07	0.03
55	1_4940110	1	4,940,110	31.77	6.38	0.04
56	1_4972393	1	4,972,393	35.26	6.10	0.05
57	1_4974472	1	4,974,472	32.48	18.69	0.00
58	1_4974479	1	4,974,479	32.5	19.38	0.00
59	1_4974480	1	4,974,480	32.87	21.21	0.00
60	1_4974481	1	4,974,481	32.87	20.54	0.00
61	1_4980046	1	4,980,046	37.56	6.10	0.05
62	1_4980096	1	4,980,096	37.27	6.10	0.05
63	1_5057984	1	5,057,984	37.26	6.38	0.04
64	1_5385542	1	5,385,542	38.71	3.59	0.17
65	1_5385613	1	5,385,613	39.97	4.38	0.11
66	1_5394050	1	5,394,050	39.47	4.38	0.11
67	1_5394075	1	5,394,075	39.74	4.38	0.11
68	1_5943100	1	5,943,100	42.04	5.77	0.06
69	1_6261686	1	6,261,686	43.46	6.85	0.03
70	1_6261766	1	6,261,766	43.46	6.85	0.03
71	1_6586327	1	6,586,327	44.28	6.85	0.03
72	1_6603064	1	6,603,064	45.39	6.79	0.03
73	1_6603066	1	6,603,066	45.17	6.79	0.03
74	1_6605857	1	6,605,857	46.58	6.06	0.05
75	1_6607229	1	6,607,229	46.38	6.06	0.05
76	1_6607633	1	6,607,633	45.14	6.06	0.05
77	1_6625221	1	6,625,221	45.9	6.38	0.04
78	1_6628521	1	6,628,521	45.74	6.38	0.04
79	1_6631514	1	6,631,514	44.39	6.66	0.04
80	1_6637941	1	6,637,941	44.82	5.83	0.05
81	1_6647981	1	6,647,981	47.22	7.11	0.03
82	1_6648321	1	6,648,321	47.38	7.11	0.03
83	1_6915045	1	6,915,045	45.54	6.06	0.05
84	1_7273355	1	7,273,355	48.84	4.03	0.13
85	1_7306931	1	7,306,931	50.74	5.60	0.06
86	1_7308788	1	7,308,788	50.21	5.67	0.06
87	1_7315043	1	7,315,043	50.25	5.83	0.05
88	1_7454118	1	7,454,118	49.57	3.20	0.20
89	1_7454769	1	7,454,769	49.87	3.20	0.20
90	1_7456456	1	7,456,456	50.21	3.94	0.14
91	1_7474754	1	7,474,754	51.09	3.31	0.19
92	1_7474840	1	7,474,840	51.3	3.31	0.19
93	1_7508684	1	7,508,684	49.32	3.27	0.20
94	1_7520917	1	7,520,917	49.99	3.20	0.20

95	1_7801545	1	7,801,545	51.82	2.58	0.28
96	1_8045187	1	8,045,187	54.45	1.57	0.46
97	1_8053826	1	8,053,826	55.38	3.20	0.20
98	1_8053833	1	8,053,833	55.27	3.20	0.20
99	1_8107685	1	8,107,685	54.61	1.48	0.48
100	1_8108060	1	8,108,060	54.29	2.03	0.36
101	1_8108150	1	8,108,150	54.21	2.03	0.36
102	1_8236301	1	8,236,301	55.22	3.20	0.20
103	1_8292652	1	8,292,652	55.12	1.97	0.37
104	1_8812653	1	8,812,653	56.67	3.27	0.20
105	1_8812834	1	8,812,834	56.52	4.89	0.09
106	1_8832732	1	8,832,732	56.88	3.27	0.20
107	1_8868145	1	8,868,145	57.25	4.03	0.13
108	1_9069079	1	9,069,079	59.35	4.83	0.09
109	1_9195230	1	9,195,230	59.08	4.83	0.09
110	1_9243746	1	9,243,746	58.48	4.83	0.09
111	1_9296871	1	9,296,871	58.43	4.83	0.09
112	1_9306386	1	9,306,386	58.83	4.83	0.09
113	1_9827591	1	9,827,591	60.48	3.98	0.14
114	1_9831177	1	9,831,177	60.7	4.38	0.11
115	1_9839304	1	9,839,304	60.91	3.98	0.14
116	1_10812154	1	10,812,154	64.3	3.20	0.20
117	1_10829409	1	10,829,409	64.85	2.58	0.28
118	1_10867915	1	10,867,915	65.89	5.67	0.06
119	1_10895145	1	10,895,145	64.52	2.58	0.28
120	1_10925061	1	10,925,061	64.7	1.97	0.37
121	1_10925063	1	10,925,063	64.64	1.97	0.37
122	1_10939252	1	10,939,252	65.38	3.27	0.20
123	1_10945604	1	10,945,604	66.14	5.83	0.05
124	1_10945733	1	10,945,733	66.29	5.83	0.05
125	1_10945842	1	10,945,842	66.5	3.92	0.14
126	1_10945970	1	10,945,970	66.7	3.92	0.14
127	1_10980449	1	10,980,449	65.89	5.67	0.06
128	1_10999522	1	10,999,522	67.3	4.89	0.09
129	1_10999616	1	10,999,616	65.59	4.03	0.13
130	1_11021329	1	11,021,329	65.68	5.83	0.05
131	1_14773440	1	14,773,440	71	2.58	0.28
132	1_14773447	1	14,773,447	71.21	2.58	0.28
133	1_14927523	1	14,927,523	71.34	2.54	0.28
134	1_15512781	1	15,512,781	71.43	3.20	0.20
135	1_15515779	1	15,515,779	73.02	3.27	0.20
136	1_15517113	1	15,517,113	70.78	3.42	0.18
137	1_15527492	1	15,527,492	72.33	4.76	0.09
138	1_15537082	1	15,537,082	74.04	2.87	0.24
139	1_15545604	1	15,545,604	73.66	2.08	0.35
140	1_15546937	1	15,546,937	71.8	3.94	0.14
141	1_15561560	1	15,561,560	74.04	2.52	0.28
142	1_15572776	1	15,572,776	73.22	3.27	0.20
143	1_15605841	1	15,605,841	72.59	4.89	0.09

144	1_15982265	1	15,982,265	76.19	3.14	0.21
145	1_16252635	1	16,252,635	77.13	2.24	0.33
146	1_16261375	1	16,261,375	77.03	2.65	0.27
147	1_16561199	1	16,561,199	78.69	1.70	0.43
148	1_16585431	1	16,585,431	78.28	1.24	0.54
149	1_19375179	1	19,375,179	88.04	1.46	0.48
150	1_19501963	1	19,501,963	87.8	1.74	0.42
151	1_19772871	1	19,772,871	89.14	1.14	0.57
152	1_19800893	1	19,800,893	89.39	1.46	0.48
153	1_19854430	1	19,854,430	88.99	0.96	0.62
154	1_19887233	1	19,887,233	89.47	1.31	0.52
155	1_19941374	1	19,941,374	90.3	2.26	0.32
156	1_19941504	1	19,941,504	90.29	2.36	0.31
157	1_19949372	1	19,949,372	90.1	1.14	0.57
158	1_19949738	1	19,949,738	89.86	0.96	0.62
159	1_20502883	1	20,502,883	93.16	0.36	0.84
160	1_20522287	1	20,522,287	92.43	0.69	0.71
161	1_20539775	1	20,539,775	92.9	0.29	0.87
162	1_20545561	1	20,545,561	92.86	0.36	0.84
163	1_20760403	1	20,760,403	93.57	0.23	0.89
164	1_20964302	1	20,964,302	94.34	0.41	0.81
165	1_20992511	1	20,992,511	94.45	0.41	0.81
166	1_21018091	1	21,018,091	94.1	0.41	0.81
167	1_22066573	1	22,066,573	96.98	0.57	0.75
168	1_22067710	1	22,067,710	96.78	0.57	0.75
169	1_22067884	1	22,067,884	97.86	0.96	0.62
170	1_22096171	1	22,096,171	97.78	0.96	0.62
171	1_22103637	1	22,103,637	97.78	0.96	0.62
172	1_22636956	1	22,636,956	100.24	0.57	0.75
173	1_23254646	1	23,254,646	102.58	0.19	0.91
174	1_23287082	1	23,287,082	103.18	0.06	0.97
175	1_23552277	1	23,552,277	103.28	0.06	0.97
176	1_23723485	1	23,723,485	104.48	0.06	0.97
177	1_23726861	1	23,726,861	104.54	0.02	0.99
178	1_24739318	1	24,739,318	109.84	0.92	0.63
179	1_25069200	1	25,069,200	110.75	0.57	0.75
180	1_25070033	1	25,070,033	110.67	0.57	0.75
181	1_25086467	1	25,086,467	112.02	0.13	0.94
182	1_25145137	1	25,145,137	111.31	0.52	0.77
183	1_25145156	1	25,145,156	111.59	0.52	0.77
184	1_25163472	1	25,163,472	111.31	0.29	0.87
185	1_25202252	1	25,202,252	111.87	0.29	0.87
186	1_25326212	1	25,326,212	113.41	0.36	0.84
187	1_25351310	1	25,351,310	113.47	0.36	0.84
188	1_25710146	1	25,710,146	114.8	0.90	0.64
189	1_26177623	1	26,177,623	116.99	0.41	0.81
190	1_26180458	1	26,180,458	116.28	0.90	0.64
191	1_26180617	1	26,180,617	117.21	0.69	0.71
192	1_26197503	1	26,197,503	116.7	0.69	0.71

193	1_26198664	1	26,198,664	117.17	0.41	0.81
194	1_26941933	1	26,941,933	120.22	1.41	0.50
195	1_27668711	1	27,668,711	121.6	1.14	0.57
196	1_27668738	1	27,668,738	121.74	0.96	0.62
197	1_27750202	1	27,750,202	122.82	0.36	0.84
198	1_27750262	1	27,750,262	122.27	1.31	0.52
199	1_27750277	1	27,750,277	122.39	0.96	0.62
200	1_27861478	1	27,861,478	123.79	0.85	0.66
201	1_27882629	1	27,882,629	124.01	0.52	0.77
202	1_27885562	1	27,885,562	123.43	1.25	0.53
203	1_27885797	1	27,885,797	123.34	0.85	0.66
204	1_28059455	1	28,059,455	123.34	1.25	0.53
205	1_28060182	1	28,060,182	124.34	0.86	0.65
206	1_28674401	1	28,674,401	126.54	2.24	0.33
207	1_29640551	1	29,640,551	128.1	2.86	0.24
208	1_29928193	1	29,928,193	128.6	3.59	0.17
209	1_30123683	1	30,123,683	130.28	2.97	0.23
210	1_30821963	1	30,821,963	132.11	5.17	0.08
211	1_31003816	1	31,003,816	132.31	5.15	0.08
212	1_31019879	1	31,019,879	131.94	4.31	0.12
213	1_31175478	1	31,175,478	132	5.17	0.08
214	1_31485080	1	31,485,080	134.74	5.15	0.08
215	1_31487079	1	31,487,079	134.41	5.15	0.08
216	1_31487372	1	31,487,372	135.06	5.22	0.07
217	1_31488042	1	31,488,042	134.41	5.22	0.07
218	1_31791378	1	31,791,378	135.41	5.38	0.07
219	1_32023709	1	32,023,709	135.94	5.38	0.07
220	1_32132344	1	32,132,344	136.13	5.62	0.06
221	1_32137376	1	32,137,376	136.9	3.64	0.16
222	1_32137408	1	32,137,408	137.35	4.05	0.13
223	1_32137436	1	32,137,436	137.35	3.64	0.16
224	1_32137606	1	32,137,606	137.35	4.05	0.13
225	1_32137698	1	32,137,698	136.87	4.05	0.13
226	1_32137728	1	32,137,728	137.35	3.32	0.19
227	1_32170754	1	32,170,754	138.41	5.38	0.07
228	1_32506255	1	32,506,255	138.25	4.61	0.10
229	1_32509306	1	32,509,306	141.1	3.59	0.17
230	2_151878	2	151,878	1.22	7.01	0.03
231	2_522960	2	522,960	1.22	7.01	0.03
232	2_535955	2	535,955	0	7.01	0.03
233	2_552548	2	552,548	2.3	6.51	0.04
234	2_639308	2	639,308	2.89	6.51	0.04
235	2_718981	2	718,981	2.9	6.51	0.04
236	2_917974	2	917,974	2.3	6.51	0.04
237	2_1107562	2	1,107,562	3.68	5.11	0.08
238	2_1186373	2	1,186,373	4.46	6.51	0.04
239	2_1335151	2	1,335,151	5.28	7.74	0.02
240	2_1492432	2	1,492,432	7.16	10.64	0.00
241	2_1500333	2	1,500,333	7.23	10.64	0.00

242	2_1887111	2	1,887,111	9.26	12.61	0.00
243	2_1890241	2	1,890,241	8.77	12.15	0.00
244	2_1893203	2	1,893,203	10.17	11.04	0.00
245	2_1905314	2	1,905,314	9.26	12.61	0.00
246	2_1957580	2	1,957,580	9.31	12.61	0.00
247	2_1964327	2	1,964,327	8.96	12.61	0.00
248	2_2088860	2	2,088,860	9.88	12.61	0.00
249	2_2148142	2	2,148,142	10.15	11.04	0.00
250	2_2157649	2	2,157,649	10.04	12.61	0.00
251	2_2179619	2	2,179,619	12.04	9.59	0.01
252	2_2209483	2	2,209,483	12.04	10.02	0.01
253	2_2392810	2	2,392,810	11.17	9.59	0.01
254	2_2689854	2	2,689,854	12.5	10.02	0.01
255	2_2689956	2	2,689,956	12.61	10.02	0.01
256	2_2706040	2	2,706,040	11.55	9.59	0.01
257	2_2708829	2	2,708,829	11.47	9.59	0.01
258	2_2708949	2	2,708,949	13.28	11.77	0.00
259	2_2709031	2	2,709,031	12.94	10.64	0.00
260	2_2713434	2	2,713,434	12.04	9.59	0.01
261	2_2818990	2	2,818,990	15.67	11.53	0.00
262	2_2825198	2	2,825,198	16.44	11.53	0.00
263	2_2874546	2	2,874,546	16.11	10.54	0.01
264	2_2875813	2	2,875,813	16.22	10.54	0.01
265	2_3170133	2	3,170,133	19.51	9.46	0.01
266	2_3301246	2	3,301,246	19.81	10.26	0.01
267	2_3301375	2	3,301,375	20.09	10.26	0.01
268	2_3301921	2	3,301,921	19.43	8.80	0.01
269	2_3302034	2	3,302,034	19.03	8.80	0.01
270	2_3303315	2	3,303,315	19.14	8.80	0.01
271	2_3303323	2	3,303,323	19.29	8.80	0.01
272	2_3400831	2	3,400,831	19.43	8.80	0.01
273	2_3439526	2	3,439,526	18.87	8.80	0.01
274	2_3462983	2	3,462,983	18.44	9.08	0.01
275	2_3473826	2	3,473,826	18.65	9.63	0.01
276	2_3559676	2	3,559,676	21.68	10.26	0.01
277	2_3595179	2	3,595,179	21.43	11.14	0.00
278	2_3597964	2	3,597,964	21.84	10.26	0.01
279	2_3918910	2	3,918,910	24.14	6.23	0.04
280	2_3918925	2	3,918,925	23.93	6.78	0.03
281	2_4024313	2	4,024,313	23.32	8.06	0.02
282	2_4235915	2	4,235,915	25.45	6.23	0.04
283	2_4287501	2	4,287,501	30.47	8.80	0.01
284	2_4295132	2	4,295,132	30.95	9.46	0.01
285	2_4295178	2	4,295,178	30.75	9.46	0.01
286	2_4371493	2	4,371,493	27.65	5.71	0.06
287	2_4371603	2	4,371,603	27.79	5.71	0.06
288	2_4556170	2	4,556,170	30.41	9.46	0.01
289	2_4571046	2	4,571,046	28.45	8.23	0.02
290	2_4571069	2	4,571,069	26.51	7.46	0.02

291	2_4605780	2	4,605,780	28.72	8.23	0.02
292	2_4605960	2	4,605,960	26.51	5.71	0.06
293	2_4636786	2	4,636,786	29.64	9.08	0.01
294	2_4694301	2	4,694,301	30.1	10.26	0.01
295	2_4694359	2	4,694,359	32.22	11.83	0.00
296	2_4807809	2	4,807,809	29.23	9.08	0.01
297	2_5777566	2	5,777,566	35.5	10.26	0.01
298	2_5777760	2	5,777,760	36.63	9.63	0.01
299	2_5777768	2	5,777,768	36.65	9.63	0.01
300	2_5777818	2	5,777,818	34.57	11.14	0.00
301	2_5777843	2	5,777,843	34.67	11.14	0.00
302	2_5778016	2	5,778,016	35.93	9.63	0.01
303	2_5792234	2	5,792,234	35.14	11.14	0.00
304	2_5946312	2	5,946,312	35.63	10.26	0.01
305	2_5986297	2	5,986,297	36.42	8.80	0.01
306	2_5998141	2	5,998,141	37.19	10.26	0.01
307	2_5998161	2	5,998,161	36.63	9.63	0.01
308	2_6038117	2	6,038,117	37.68	8.75	0.01
309	2_6038449	2	6,038,449	37.73	8.75	0.01
310	2_6130347	2	6,130,347	39.62	8.23	0.02
311	2_6302782	2	6,302,782	40.02	7.34	0.03
312	2_6314840	2	6,314,840	39.39	8.23	0.02
313	2_6314939	2	6,314,939	48.17	37.90	0.00
314	2_6314958	2	6,314,958	48.17	37.90	0.00
315	2_6473847	2	6,473,847	38.71	8.80	0.01
316	2_6474226	2	6,474,226	39.39	8.80	0.01
317	2_6474624	2	6,474,624	39.04	8.80	0.01
318	2_6531947	2	6,531,947	40.57	6.94	0.03
319	2_6741335	2	6,741,335	40.91	6.51	0.04
320	2_6741404	2	6,741,404	41.18	6.51	0.04
321	2_7015092	2	7,015,092	43.19	8.06	0.02
322	2_7199035	2	7,199,035	46.1	11.94	0.00
323	2_7203096	2	7,203,096	44.49	10.20	0.01
324	2_7215274	2	7,215,274	45.5	11.03	0.00
325	2_7215322	2	7,215,322	45.32	11.03	0.00
326	2_7706522	2	7,706,522	44.49	7.41	0.02
327	2_7706537	2	7,706,537	44.32	7.41	0.02
328	2_7714991	2	7,714,991	43.82	6.78	0.03
329	2_7994419	2	7,994,419	54.91	8.06	0.02
330	2_8004354	2	8,004,354	54.69	8.06	0.02
331	2_8029836	2	8,029,836	55.44	8.06	0.02
332	2_8265377	2	8,265,377	53.36	8.06	0.02
333	2_8265641	2	8,265,641	54.54	7.46	0.02
334	2_8265840	2	8,265,840	54.32	8.06	0.02
335	2_8277989	2	8,277,989	51.8	9.46	0.01
336	2_8278002	2	8,278,002	51.66	9.46	0.01
337	2_8320927	2	8,320,927	50.32	27.88	0.00
338	2_8360188	2	8,360,188	53.05	8.06	0.02
339	2_8361432	2	8,361,432	52.58	8.75	0.01

340	2_8633934	2	8,633,934	56.01	6.17	0.05
341	2_8842310	2	8,842,310	57.49	7.57	0.02
342	2_9699386	2	9,699,386	61.06	4.59	0.10
343	2_10140569	2	10,140,569	63.06	3.21	0.20
344	2_10218937	2	10,218,937	63.28	2.92	0.23
345	2_10238310	2	10,238,310	63.54	2.92	0.23
346	2_10271021	2	10,271,021	63.84	2.92	0.23
347	2_11201384	2	11,201,384	67.41	3.94	0.14
348	2_11209303	2	11,209,303	67.98	3.99	0.14
349	2_11209309	2	11,209,309	67.86	3.99	0.14
350	2_11209353	2	11,209,353	68.23	3.99	0.14
351	2_11291671	2	11,291,671	69.43	3.99	0.14
352	2_11291751	2	11,291,751	69.59	3.99	0.14
353	2_11845631	2	11,845,631	71.82	12.44	0.00
354	2_11845636	2	11,845,636	71.76	13.83	0.00
355	2_11845729	2	11,845,729	71.76	13.83	0.00
356	2_11845841	2	11,845,841	71.63	12.44	0.00
357	2_12364524	2	12,364,524	73.87	4.15	0.13
358	2_12528438	2	12,528,438	76.47	4.38	0.11
359	2_12567819	2	12,567,819	75.97	4.38	0.11
360	2_12573930	2	12,573,930	76.18	4.38	0.11
361	2_12913139	2	12,913,139	80.64	3.76	0.15
362	2_12922494	2	12,922,494	81.11	3.76	0.15
363	2_12964090	2	12,964,090	80.95	3.76	0.15
364	2_12964173	2	12,964,173	79.84	3.76	0.15
365	2_13236010	2	13,236,010	79.84	3.76	0.15
366	2_13271033	2	13,271,033	81.38	3.21	0.20
367	2_13273280	2	13,273,280	81.77	2.92	0.23
368	2_13310039	2	13,310,039	81.98	3.21	0.20
369	2_13312628	2	13,312,628	82.12	3.59	0.17
370	2_13390230	2	13,390,230	81.98	3.21	0.20
371	2_13519051	2	13,519,051	82.93	2.80	0.25
372	2_13519096	2	13,519,096	83.11	2.80	0.25
373	2_13532387	2	13,532,387	83.35	3.15	0.21
374	2_13564523	2	13,564,523	93.81	47.78	0.00
375	2_14360862	2	14,360,862	88.85	7.34	0.03
376	2_14360863	2	14,360,863	88.4	7.01	0.03
377	2_14363128	2	14,363,128	85.53	3.59	0.17
378	2_14432019	2	14,432,019	86.22	3.59	0.17
379	2_14445339	2	14,445,339	91.39	14.38	0.00
380	2_14445345	2	14,445,345	92.14	20.33	0.00
381	2_14476270	2	14,476,270	94.38	48.23	0.00
382	2_14478101	2	14,478,101	94.36	48.23	0.00
383	2_14488278	2	14,488,278	87.27	3.21	0.20
384	2_14490136	2	14,490,136	85.12	3.59	0.17
385	2_15111463	2	15,111,463	97.08	6.44	0.04
386	2_15113147	2	15,113,147	99.07	3.92	0.14
387	2_15113274	2	15,113,274	98.55	3.92	0.14
388	2_15120696	2	15,120,696	100.04	4.61	0.10

389	2_15150047	2	15,150,047	101.14	4.43	0.11
390	2_15154586	2	15,154,586	101.65	3.71	0.16
391	2_15155426	2	15,155,426	102.72	3.92	0.14
392	2_15181473	2	15,181,473	102.19	3.71	0.16
393	2_15513656	2	15,513,656	102.72	3.71	0.16
394	2_16933398	2	16,933,398	105.47	8.40	0.02
395	2_16988223	2	16,988,223	105.98	7.97	0.02
396	2_17433691	2	17,433,691	108.48	5.66	0.06
397	2_17477051	2	17,477,051	109.3	5.94	0.05
398	2_17489172	2	17,489,172	109.39	5.94	0.05
399	2_17492788	2	17,492,788	108.97	5.94	0.05
400	2_17944215	2	17,944,215	112.53	5.06	0.08
401	2_18166428	2	18,166,428	113.36	5.22	0.07
402	2_18177160	2	18,177,160	113.79	5.22	0.07
403	2_18445257	2	18,445,257	114.98	5.62	0.06
404	2_18445294	2	18,445,294	114.88	5.62	0.06
405	2_18478151	2	18,478,151	115.69	4.87	0.09
406	2_18478773	2	18,478,773	115.48	4.87	0.09
407	2_18853976	2	18,853,976	118.84	6.23	0.04
408	2_18854099	2	18,854,099	120.48	7.17	0.03
409	2_18854192	2	18,854,192	119.88	6.10	0.05
410	2_18854707	2	18,854,707	120.2	6.23	0.04
411	2_18892027	2	18,892,027	121.04	7.07	0.03
412	2_19036974	2	19,036,974	122.37	7.06	0.03
413	2_19054167	2	19,054,167	122.13	7.07	0.03
414	2_19104387	2	19,104,387	123.09	8.13	0.02
415	2_19140683	2	19,140,683	122.88	8.13	0.02
416	2_19170675	2	19,170,675	124.19	8.12	0.02
417	2_19720198	2	19,720,198	127.77	7.52	0.02
418	2_19735773	2	19,735,773	128.99	7.11	0.03
419	2_19759419	2	19,759,419	128.67	6.73	0.03
420	2_19774895	2	19,774,895	128.57	6.73	0.03
421	2_19775051	2	19,775,051	129.05	7.11	0.03
422	2_19818379	2	19,818,379	129.3	7.57	0.02
423	2_19948256	2	19,948,256	130.76	4.59	0.10
424	2_19948296	2	19,948,296	130.15	5.50	0.06
425	2_19948411	2	19,948,411	130.32	4.59	0.10
426	2_19948468	2	19,948,468	130.58	4.59	0.10
427	2_19954763	2	19,954,763	130.15	5.50	0.06
428	2_20162556	2	20,162,556	131.84	5.06	0.08
429	2_20601150	2	20,601,150	136.38	5.15	0.08
430	2_20601283	2	20,601,283	135.83	5.90	0.05
431	2_20601328	2	20,601,328	136.33	5.15	0.08
432	2_20632190	2	20,632,190	136.66	6.01	0.05
433	2_20767890	2	20,767,890	138.12	5.60	0.06
434	2_20768261	2	20,768,261	138.39	5.60	0.06
435	2_20772473	2	20,772,473	137.51	7.74	0.02
436	2_20780302	2	20,780,302	137.62	6.94	0.03
437	2_20847149	2	20,847,149	137.86	5.60	0.06

438	2_20871668	2	20,871,668	137.86	5.60	0.06
439	2_20875024	2	20,875,024	137.14	6.78	0.03
440	2_21032386	2	21,032,386	142.61	1.35	0.51
441	2_21372224	2	21,372,224	144.02	2.37	0.31
442	2_21373611	2	21,373,611	143.72	1.87	0.39
443	2_21522433	2	21,522,433	145.73	1.31	0.52
444	2_21530113	2	21,530,113	146.21	1.07	0.59
445	2_21565749	2	21,565,749	150.31	1.80	0.41
446	2_21588134	2	21,588,134	149.33	1.80	0.41
447	2_21685139	2	21,685,139	153.94	1.35	0.51
448	2_21722626	2	21,722,626	154.13	1.35	0.51
449	2_21725502	2	21,725,502	151.86	1.07	0.59
450	2_21755294	2	21,755,294	152.99	0.85	0.66
451	2_21764378	2	21,764,378	150.79	1.53	0.46
452	2_21764674	2	21,764,674	151.3	1.80	0.41
453	2_21766448	2	21,766,448	148.1	0.90	0.64
454	2_21777795	2	21,777,795	148.75	1.35	0.51
455	2_21777809	2	21,777,809	148.82	1.35	0.51
456	2_21778154	2	21,778,154	153.28	0.85	0.66
457	2_21778332	2	21,778,332	153.23	0.85	0.66
458	2_21807059	2	21,807,059	151.42	2.15	0.34
459	2_21810042	2	21,810,042	151.53	1.80	0.41
460	2_21811296	2	21,811,296	148.34	1.31	0.52
461	2_21822335	2	21,822,335	149.75	1.80	0.41
462	2_21877998	2	21,877,998	149.95	1.80	0.41
463	2_21878090	2	21,878,090	149.83	1.80	0.41
464	2_21880076	2	21,880,076	152.8	1.07	0.59
465	2_21898068	2	21,898,068	149.5	1.80	0.41
466	2_21911696	2	21,911,696	152.19	1.07	0.59
467	2_21949524	2	21,949,524	147.58	0.69	0.71
468	2_21957602	2	21,957,602	152.17	1.07	0.59
469	2_21968173	2	21,968,173	150.49	1.80	0.41
470	2_22021917	2	22,021,917	153.68	0.85	0.66
471	2_22060024	2	22,060,024	152.55	1.07	0.59
472	2_22063114	2	22,063,114	151.04	1.53	0.46
473	2_22063239	2	22,063,239	151.15	1.53	0.46
474	2_22073082	2	22,073,082	152.27	1.31	0.52
475	2_22080536	2	22,080,536	147.59	0.40	0.82
476	2_22080924	2	22,080,924	153.48	0.85	0.66
477	2_22093007	2	22,093,007	147.43	1.53	0.46
478	2_22096073	2	22,096,073	153.72	0.92	0.63
479	2_22183112	2	22,183,112	148.72	1.35	0.51
480	2_22466275	2	22,466,275	155.42	0.69	0.71
481	2_22586019	2	22,586,019	156.34	0.57	0.75
482	2_22769763	2	22,769,763	157.27	0.69	0.71
483	2_23047400	2	23,047,400	159.38	1.63	0.44
484	2_23062961	2	23,062,961	159.7	1.63	0.44
485	2_23227850	2	23,227,850	159.38	1.63	0.44
486	2_23253567	2	23,253,567	160.49	1.80	0.41

487	2_23535378	2	23,535,378	162.83	1.57	0.46
488	2_23535379	2	23,535,379	162.83	1.57	0.46
489	2_23537629	2	23,537,629	163.04	1.57	0.46
490	2_24060647	2	24,060,647	164.75	0.90	0.64
491	2_24333780	2	24,333,780	167.21	0.92	0.63
492	2_24334421	2	24,334,421	166.38	0.92	0.63
493	3_13060	3	13,060	3.65	3.71	0.16
494	3_271209	3	271,209	1.2	3.71	0.16
495	3_298013	3	298,013	1.2	3.14	0.21
496	3_313483	3	313,483	1.94	3.71	0.16
497	3_319136	3	319,136	2.16	4.37	0.11
498	3_319453	3	319,453	2.23	4.37	0.11
499	3_319657	3	319,657	3.1	3.08	0.21
500	3_347148	3	347,148	0.45	5.11	0.08
501	3_347188	3	347,188	0	5.11	0.08
502	3_384298	3	384,298	2.7	3.71	0.16
503	3_387158	3	387,158	3.42	3.14	0.21
504	3_391026	3	391,026	3.19	3.08	0.21
505	3_392142	3	392,142	1.26	3.14	0.21
506	3_393861	3	393,861	2.07	4.37	0.11
507	3_414117	3	414,117	1.69	3.14	0.21
508	3_414137	3	414,137	1.69	3.14	0.21
509	3_443599	3	443,599	2.6	3.71	0.16
510	3_548547	3	548,547	1.21	3.14	0.21
511	3_604525	3	604,525	2.4	4.37	0.11
512	3_604552	3	604,552	2.43	3.71	0.16
513	3_630642	3	630,642	3.54	3.71	0.16
514	3_1628557	3	1,628,557	3.04	3.08	0.21
515	3_1632345	3	1,632,345	4.56	3.71	0.16
516	3_1646758	3	1,646,758	4.41	3.71	0.16
517	3_1646818	3	1,646,818	4.52	3.71	0.16
518	3_1668737	3	1,668,737	4.08	3.14	0.21
519	3_1668913	3	1,668,913	4.14	3.14	0.21
520	3_1668986	3	1,668,986	4.01	3.14	0.21
521	3_2170269	3	2,170,269	5.03	3.14	0.21
522	3_2777096	3	2,777,096	7.75	3.08	0.21
523	3_2779961	3	2,779,961	7.4	3.14	0.21
524	3_2917160	3	2,917,160	7.48	2.54	0.28
525	3_2917176	3	2,917,176	7.51	2.54	0.28
526	3_2917304	3	2,917,304	7.57	3.08	0.21
527	3_2917342	3	2,917,342	7.96	3.71	0.16
528	3_3343817	3	3,343,817	9.12	4.43	0.11
529	3_3346547	3	3,346,547	9.39	3.31	0.19
530	3_3349259	3	3,349,259	9.28	3.83	0.15
531	3_3485061	3	3,485,061	10.3	2.08	0.35
532	3_3846735	3	3,846,735	12.23	2.54	0.28
533	3_3866090	3	3,866,090	13.08	2.65	0.27
534	3_3866450	3	3,866,450	12.72	3.14	0.21
535	3_3878273	3	3,878,273	13.03	2.65	0.27

536	3_3879952	3	3,879,952	12.89	2.65	0.27
537	3_3885212	3	3,885,212	13.72	3.14	0.21
538	3_3885315	3	3,885,315	13.77	3.14	0.21
539	3_4000013	3	4,000,013	14.8	3.14	0.21
540	3_4063840	3	4,063,840	17.79	3.71	0.16
541	3_4064522	3	4,064,522	19.83	3.71	0.16
542	3_4175838	3	4,175,838	15.65	3.08	0.21
543	3_4175928	3	4,175,928	15.79	3.08	0.21
544	3_4190229	3	4,190,229	19.02	3.83	0.15
545	3_4191676	3	4,191,676	19.12	3.83	0.15
546	3_4191931	3	4,191,931	19.61	3.14	0.21
547	3_4219401	3	4,219,401	20.31	3.71	0.16
548	3_4233180	3	4,233,180	20.45	3.71	0.16
549	3_4233229	3	4,233,229	20.5	3.71	0.16
550	3_4283906	3	4,283,906	18.14	4.37	0.11
551	3_4328076	3	4,328,076	18.3	3.71	0.16
552	3_4332453	3	4,332,453	20.01	3.08	0.21
553	3_4338292	3	4,338,292	18.81	4.43	0.11
554	3_4344167	3	4,344,167	15.3	3.71	0.16
555	3_4353094	3	4,353,094	18.59	3.71	0.16
556	3_4364610	3	4,364,610	16.14	3.71	0.16
557	3_4365332	3	4,365,332	19.38	3.14	0.21
558	3_5055123	3	5,055,123	20.95	3.14	0.21
559	3_5055334	3	5,055,334	21.17	3.14	0.21
560	3_5056510	3	5,056,510	17.38	3.08	0.21
561	3_5062699	3	5,062,699	21.51	3.08	0.21
562	3_5074906	3	5,074,906	21.33	3.71	0.16
563	3_5079630	3	5,079,630	17.06	2.54	0.28
564	3_5079646	3	5,079,646	16.83	3.14	0.21
565	3_5079659	3	5,079,659	16.61	3.14	0.21
566	3_5210097	3	5,210,097	21.79	3.08	0.21
567	3_5210195	3	5,210,195	21.73	3.08	0.21
568	3_5582062	3	5,582,062	24.27	2.65	0.27
569	3_5698492	3	5,698,492	24	2.65	0.27
570	3_5771355	3	5,771,355	23.74	3.14	0.21
571	3_5828192	3	5,828,192	23.8	3.14	0.21
572	3_5929783	3	5,929,783	23.62	3.14	0.21
573	3_5933951	3	5,933,951	25.21	2.54	0.28
574	3_5953666	3	5,953,666	23.31	3.14	0.21
575	3_5954395	3	5,954,395	26.86	3.08	0.21
576	3_5954569	3	5,954,569	27.22	3.71	0.16
577	3_6017474	3	6,017,474	24.77	2.65	0.27
578	3_6081074	3	6,081,074	25.21	2.54	0.28
579	3_6131749	3	6,131,749	24.95	2.65	0.27
580	3_7241659	3	7,241,659	31.61	2.08	0.35
581	3_7365605	3	7,365,605	33.92	0.40	0.82
582	3_7365769	3	7,365,769	33.54	0.69	0.71
583	3_7365851	3	7,365,851	33.74	0.69	0.71
584	3_7383282	3	7,383,282	34.16	0.47	0.79

585	3_7400006	3	7,400,006	34.89	0.36	0.84
586	3_7784369	3	7,784,369	36.36	0.41	0.81
587	3_7837476	3	7,837,476	36.11	0.47	0.79
588	3_7837677	3	7,837,677	35.76	0.36	0.84
589	3_8170896	3	8,170,896	38.49	0.36	0.84
590	3_8196108	3	8,196,108	40.42	0.08	0.96
591	3_8215264	3	8,215,264	38.84	0.23	0.89
592	3_8215829	3	8,215,829	38.4	0.29	0.87
593	3_8220010	3	8,220,010	39.41	0.13	0.94
594	3_8221272	3	8,221,272	39.5	0.13	0.94
595	3_8323371	3	8,323,371	38.49	0.29	0.87
596	3_8333874	3	8,333,874	39.75	0.08	0.96
597	3_8333952	3	8,333,952	40.2	0.01	1.00
598	3_8701925	3	8,701,925	37.97	0.36	0.84
599	3_8708179	3	8,708,179	38.49	0.29	0.87
600	3_8987209	3	8,987,209	41.72	0.30	0.86
601	3_8987241	3	8,987,241	41.89	0.30	0.86
602	3_8993836	3	8,993,836	39.41	0.23	0.89
603	3_8994020	3	8,994,020	42.53	0.12	0.94
604	3_9000781	3	9,000,781	41.56	0.12	0.94
605	3_9286244	3	9,286,244	43.81	0.13	0.94
606	3_9286319	3	9,286,319	43.89	0.13	0.94
607	3_9380734	3	9,380,734	44.64	0.12	0.94
608	3_9624718	3	9,624,718	46.31	0.12	0.94
609	3_9639091	3	9,639,091	45.35	0.12	0.94
610	3_9681268	3	9,681,268	45.52	0.13	0.94
611	3_9744701	3	9,744,701	46.04	0.19	0.91
612	3_9760937	3	9,760,937	46.31	0.19	0.91
613	3_9770069	3	9,770,069	45.71	0.12	0.94
614	3_9770163	3	9,770,163	45.56	0.13	0.94
615	3_10255482	3	10,255,482	47.61	0.13	0.94
616	3_10261861	3	10,261,861	47.21	0.13	0.94
617	3_10268364	3	10,268,364	46.97	0.06	0.97
618	3_10356720	3	10,356,720	48.6	0.06	0.97
619	3_10359912	3	10,359,912	48.85	0.08	0.96
620	3_10383344	3	10,383,344	49.12	0.08	0.96
621	3_10700857	3	10,700,857	51.62	0.02	0.99
622	3_10700946	3	10,700,946	51.54	0.02	0.99
623	3_10755206	3	10,755,206	52.74	0.06	0.97
624	3_10755250	3	10,755,250	52.99	0.06	0.97
625	3_11439629	3	11,439,629	57	0.30	0.86
626	3_11441838	3	11,441,838	55.84	0.40	0.82
627	3_11470072	3	11,470,072	56.62	0.30	0.86
628	3_11482752	3	11,482,752	56.61	0.40	0.82
629	3_11504677	3	11,504,677	56.08	0.40	0.82
630	3_11525500	3	11,525,500	55.86	0.40	0.82
631	3_11738453	3	11,738,453	58.24	0.19	0.91
632	3_11760693	3	11,760,693	58.79	0.58	0.75
633	3_11782739	3	11,782,739	57.85	0.19	0.91

634	3_12112461	3	12,112,461	60.34	0.69	0.71
635	3_12127434	3	12,127,434	62.11	0.57	0.75
636	3_12134809	3	12,134,809	62.28	0.69	0.71
637	3_12233062	3	12,233,062	60.34	0.69	0.71
638	3_12244891	3	12,244,891	61.28	0.58	0.75
639	3_12249281	3	12,249,281	62	0.13	0.94
640	3_12249303	3	12,249,303	62	0.13	0.94
641	3_12249330	3	12,249,330	61.92	0.13	0.94
642	3_12249359	3	12,249,359	61.66	0.19	0.91
643	3_12253259	3	12,253,259	60.89	0.90	0.64
644	3_12598880	3	12,598,880	64.07	0.69	0.71
645	3_12908752	3	12,908,752	65.4	0.90	0.64
646	3_12914525	3	12,914,525	65.8	0.90	0.64
647	3_12914555	3	12,914,555	65.93	0.90	0.64
648	3_12917327	3	12,917,327	65.06	0.90	0.64
649	3_12926874	3	12,926,874	64.52	0.90	0.64
650	3_12933795	3	12,933,795	64.78	0.90	0.64
651	3_12941200	3	12,941,200	66.13	0.58	0.75
652	3_12948476	3	12,948,476	67.1	0.19	0.91
653	3_12948518	3	12,948,518	67.1	0.19	0.91
654	3_12994572	3	12,994,572	65.4	0.90	0.64
655	3_13695672	3	13,695,672	71.84	0.19	0.91
656	3_13735387	3	13,735,387	72.5	0.06	0.97
657	3_13741368	3	13,741,368	72.24	0.19	0.91
658	3_13746135	3	13,746,135	71.84	0.19	0.91
659	3_13746967	3	13,746,967	71.11	0.08	0.96
660	3_13746999	3	13,746,999	71.19	0.06	0.97
661	3_13747035	3	13,747,035	70.97	0.08	0.96
662	3_13784446	3	13,784,446	72.79	0.02	0.99
663	3_13809779	3	13,809,779	73.26	0.06	0.97
664	3_13810442	3	13,810,442	73.63	0.13	0.94
665	3_13810589	3	13,810,589	73.76	0.13	0.94
666	3_13810691	3	13,810,691	73.77	0.13	0.94
667	3_13876954	3	13,876,954	74.32	0.51	0.77
668	3_13877026	3	13,877,026	74.4	0.51	0.77
669	3_14522168	3	14,522,168	77.12	0.69	0.71
670	3_14522712	3	14,522,712	77.41	0.69	0.71
671	3_14522730	3	14,522,730	77.48	0.69	0.71
672	3_14526460	3	14,526,460	77.93	0.40	0.82
673	3_14528405	3	14,528,405	78.31	0.06	0.97
674	3_14528589	3	14,528,589	78.43	0.06	0.97
675	3_14548237	3	14,548,237	76.79	0.52	0.77
676	3_14626767	3	14,626,767	80.82	0.69	0.71
677	3_14636569	3	14,636,569	81.64	0.69	0.71
678	3_14660896	3	14,660,896	81.02	0.69	0.71
679	3_14666096	3	14,666,096	81.33	0.69	0.71
680	3_14666319	3	14,666,319	80.64	0.69	0.71
681	3_14810007	3	14,810,007	80.02	0.58	0.75
682	3_14810570	3	14,810,570	80.11	0.58	0.75

683	3_14814082	3	14,814,082	80.41	0.58	0.75
684	3_14831952	3	14,831,952	79.67	0.19	0.91
685	3_14857638	3	14,857,638	80.47	0.40	0.82
686	3_14870211	3	14,870,211	79.85	0.34	0.84
687	3_14874087	3	14,874,087	81.64	0.69	0.71
688	3_15621241	3	15,621,241	85.24	2.03	0.36
689	3_15695182	3	15,695,182	90.33	1.57	0.46
690	3_15877675	3	15,877,675	86.3	2.03	0.36
691	3_15890843	3	15,890,843	90.92	2.03	0.36
692	3_15961453	3	15,961,453	85.97	2.52	0.28
693	3_15961767	3	15,961,767	90.16	1.57	0.46
694	3_16234935	3	16,234,935	89.43	1.20	0.55
695	3_16254464	3	16,254,464	86.73	1.57	0.46
696	3_16268360	3	16,268,360	86.46	2.03	0.36
697	3_16271789	3	16,271,789	93.13	1.53	0.46
698	3_16271844	3	16,271,844	87.24	1.57	0.46
699	3_16271861	3	16,271,861	87.11	1.57	0.46
700	3_16271914	3	16,271,914	86.11	2.52	0.28
701	3_16321377	3	16,321,377	93.32	1.80	0.41
702	3_16321525	3	16,321,525	93.47	2.15	0.34
703	3_16337097	3	16,337,097	88.59	2.03	0.36
704	3_16494950	3	16,494,950	91.6	2.15	0.34
705	3_16614209	3	16,614,209	89.98	1.57	0.46
706	3_16635263	3	16,635,263	92.67	1.80	0.41
707	3_16635296	3	16,635,296	92.51	1.80	0.41
708	3_16635355	3	16,635,355	92.01	1.80	0.41
709	3_16635357	3	16,635,357	92.26	1.80	0.41
710	3_16660479	3	16,660,479	90.51	1.57	0.46
711	3_16693995	3	16,693,995	87.8	2.52	0.28
712	3_16694218	3	16,694,218	88.26	2.03	0.36
713	3_16694236	3	16,694,236	88.14	2.03	0.36
714	3_16694425	3	16,694,425	88.38	2.03	0.36
715	3_16757620	3	16,757,620	89.03	1.57	0.46
716	3_16757840	3	16,757,840	88.14	2.03	0.36
717	3_16757909	3	16,757,909	89.03	1.57	0.46
718	3_17262318	3	17,262,318	94.76	1.63	0.44
719	3_17708831	3	17,708,831	100.75	0.58	0.75
720	3_17709676	3	17,709,676	97.22	1.80	0.41
721	3_17735412	3	17,735,412	98.28	1.53	0.46
722	3_17735552	3	17,735,552	97.99	1.35	0.51
723	3_17738272	3	17,738,272	96.23	1.31	0.52
724	3_17789594	3	17,789,594	102.4	0.57	0.75
725	3_17791145	3	17,791,145	102.54	0.92	0.63
726	3_17791196	3	17,791,196	102.54	0.92	0.63
727	3_17797460	3	17,797,460	101.94	1.07	0.59
728	3_17808932	3	17,808,932	101.18	0.90	0.64
729	3_17809266	3	17,809,266	96.77	1.80	0.41
730	3_17809373	3	17,809,373	96.53	1.80	0.41
731	3_17815818	3	17,815,818	97.48	1.53	0.46

732	3_17840220	3	17,840,220	97.25	1.53	0.46
733	3_18349380	3	18,349,380	103.22	2.15	0.34
734	3_18349433	3	18,349,433	103	2.15	0.34
735	3_18364263	3	18,364,263	98.93	0.90	0.64
736	3_18364277	3	18,364,277	100.46	1.20	0.55
737	3_18364315	3	18,364,315	99.18	0.90	0.64
738	3_18364341	3	18,364,341	99.8	0.90	0.64
739	3_18364360	3	18,364,360	99.42	0.90	0.64
740	3_18364364	3	18,364,364	99.58	0.90	0.64
741	3_18364477	3	18,364,477	98.53	1.31	0.52
742	3_18823416	3	18,823,416	100.46	1.20	0.55
743	3_19253003	3	19,253,003	104.38	2.37	0.31
744	3_19493744	3	19,493,744	104.08	2.37	0.31
745	3_19530148	3	19,530,148	104.8	3.03	0.22
746	3_19559083	3	19,559,083	104.21	2.37	0.31
747	3_20247363	3	20,247,363	106.22	3.92	0.14
748	3_20917609	3	20,917,609	108.57	2.15	0.34
749	3_21107965	3	21,107,965	109.26	2.37	0.31
750	3_21962723	3	21,962,723	110.94	2.47	0.29
751	3_21975345	3	21,975,345	111.21	1.87	0.39
752	3_21998335	3	21,998,335	110.94	2.47	0.29
753	3_22081427	3	22,081,427	111.93	1.87	0.39
754	3_22311916	3	22,311,916	112.21	1.87	0.39
755	3_22550360	3	22,550,360	111.21	1.87	0.39
756	3_22701717	3	22,701,717	112.22	1.87	0.39
757	3_22710390	3	22,710,390	111.77	1.87	0.39
758	3_22729218	3	22,729,218	113.22	2.08	0.35
759	3_22730362	3	22,730,362	112.76	2.08	0.35
760	3_23645586	3	23,645,586	116.08	2.24	0.33
761	3_23672190	3	23,672,190	115.91	2.24	0.33
762	3_23672191	3	23,672,191	115.96	2.24	0.33
763	3_23686434	3	23,686,434	115.63	2.24	0.33
764	3_23686566	3	23,686,566	115.19	1.25	0.53
765	3_23691056	3	23,691,056	114.71	1.74	0.42
766	3_23691090	3	23,691,090	115.13	1.25	0.53
767	3_23717240	3	23,717,240	115.4	1.74	0.42
768	3_24531511	3	24,531,511	117.33	1.25	0.53
769	3_25135149	3	25,135,149	116.86	1.25	0.53
770	3_25136812	3	25,136,812	117.33	1.25	0.53
771	3_25298790	3	25,298,790	118.12	1.31	0.52
772	3_26660507	3	26,660,507	120.08	1.14	0.57
773	3_26764693	3	26,764,693	121.96	0.90	0.64
774	3_26768428	3	26,768,428	122.07	0.69	0.71
775	3_26866345	3	26,866,345	122.97	0.57	0.75
776	3_26866691	3	26,866,691	122.5	0.69	0.71
777	3_26868790	3	26,868,790	123.22	0.57	0.75
778	3_27208062	3	27,208,062	121.96	0.90	0.64
779	3_27214274	3	27,214,274	121.65	1.20	0.55
780	3_27214441	3	27,214,441	124.58	0.36	0.84

781	3_27220377	3	27,220,377	124.04	0.57	0.75
782	3_27230830	3	27,230,830	121.08	2.58	0.28
783	3_27230907	3	27,230,907	120.91	2.58	0.28
784	3_27230996	3	27,230,996	120.89	2.58	0.28
785	3_27308438	3	27,308,438	124.89	0.36	0.84
786	3_27308557	3	27,308,557	124.32	0.57	0.75
787	3_27565517	3	27,565,517	123.96	0.86	0.65
788	3_27565711	3	27,565,711	125.95	0.36	0.84
789	3_27570468	3	27,570,468	124.04	0.86	0.65
790	3_27570750	3	27,570,750	123.65	0.86	0.65
791	3_28141838	3	28,141,838	128.28	0.41	0.81
792	3_28615750	3	28,615,750	130.93	0.96	0.62
793	3_28635718	3	28,635,718	132.38	0.86	0.65
794	3_28636482	3	28,636,482	130.27	0.57	0.75
795	3_28769081	3	28,769,081	131.38	0.96	0.62
796	3_28811806	3	28,811,806	129.9	0.36	0.84
797	3_28814852	3	28,814,852	129.9	0.36	0.84
798	3_28820581	3	28,820,581	131.09	0.96	0.62
799	3_29058240	3	29,058,240	129.74	0.36	0.84
800	3_29073547	3	29,073,547	131.72	0.96	0.62
801	3_29073566	3	29,073,566	131.87	0.96	0.62
802	3_29073604	3	29,073,604	131.81	0.96	0.62
803	3_29431857	3	29,431,857	133.93	0.92	0.63
804	3_29454353	3	29,454,353	134.11	0.57	0.75
805	3_29544646	3	29,544,646	135.4	0.13	0.94
806	3_29552649	3	29,552,649	135.64	0.12	0.94
807	3_29552673	3	29,552,673	135.64	0.12	0.94
808	3_29950770	3	29,950,770	136.6	0.13	0.94
809	3_29994274	3	29,994,274	136.17	0.13	0.94
810	3_30022239	3	30,022,239	141.16	1.07	0.59
811	3_30022243	3	30,022,243	141.16	1.07	0.59
812	3_30022279	3	30,022,279	141.16	1.07	0.59
813	3_30033109	3	30,033,109	141.88	0.02	0.99
814	3_30050166	3	30,050,166	136.84	0.06	0.97
815	3_30051276	3	30,051,276	136.41	0.13	0.94
816	3_30074658	3	30,074,658	142.27	0.08	0.96
817	3_30115827	3	30,115,827	140.32	0.02	0.99
818	3_30190813	3	30,190,813	137.38	0.36	0.84
819	3_30191074	3	30,191,074	137.18	0.17	0.92
820	3_30191200	3	30,191,200	140.05	0.17	0.92
821	3_30197655	3	30,197,655	142.86	0.06	0.97
822	3_30272874	3	30,272,874	137.68	0.17	0.92
823	3_30534122	3	30,534,122	137.49	0.17	0.92
824	3_30534544	3	30,534,544	143.15	0.06	0.97
825	3_30534604	3	30,534,604	143.49	0.06	0.97
826	3_30534658	3	30,534,658	143.32	0.06	0.97
827	3_30693991	3	30,693,991	144.03	0.08	0.96
828	3_30696184	3	30,696,184	138.18	0.36	0.84
829	3_30706906	3	30,706,906	139.61	0.36	0.84

830	3_30949901	3	30,949,901	144.51	0.08	0.96
831	3_30956483	3	30,956,483	144.37	0.08	0.96
832	3_31240931	3	31,240,931	144.75	0.08	0.96
833	3_31288848	3	31,288,848	139.34	0.36	0.84
834	3_31308317	3	31,308,317	138.76	0.36	0.84
835	3_31337870	3	31,337,870	139.03	0.36	0.84
836	3_31975995	3	31,975,995	147.74	0.17	0.92
837	3_31976077	3	31,976,077	147.64	0.08	0.96
838	3_31976358	3	31,976,358	147.74	0.08	0.96
839	3_31976385	3	31,976,385	146.89	0.08	0.96
840	3_32057082	3	32,057,082	147.12	0.08	0.96
841	3_32057128	3	32,057,128	147.12	0.08	0.96
842	3_32061967	3	32,061,967	148.32	0.08	0.96
843	3_32085894	3	32,085,894	148.49	0.08	0.96
844	3_32097331	3	32,097,331	149	0.01	1.00
845	3_32106043	3	32,106,043	149.15	0.01	1.00
846	3_32488686	3	32,488,686	153.38	0.19	0.91
847	3_32499091	3	32,499,091	152.53	0.13	0.94
848	3_33312662	3	33,312,662	156.21	0.47	0.79
849	3_33315774	3	33,315,774	156.77	0.23	0.89
850	3_33316593	3	33,316,593	157.33	0.23	0.89
851	3_33336526	3	33,336,526	156.71	0.23	0.89
852	3_33342419	3	33,342,419	157.37	0.23	0.89
853	3_33353426	3	33,353,426	156.21	0.23	0.89
854	3_33363900	3	33,363,900	157.03	0.19	0.91
855	3_33368892	3	33,368,892	156.45	0.40	0.82
856	3_33368995	3	33,368,995	156.38	0.40	0.82
857	3_33709250	3	33,709,250	158.36	0.57	0.75
858	3_33776034	3	33,776,034	158.44	0.57	0.75
859	3_33833447	3	33,833,447	159.46	0.23	0.89
860	3_33917999	3	33,917,999	159.71	0.23	0.89
861	3_33918155	3	33,918,155	159.62	0.23	0.89
862	3_33921341	3	33,921,341	159.34	0.23	0.89
863	3_33923693	3	33,923,693	159.84	0.19	0.91
864	3_33928368	3	33,928,368	160.04	0.19	0.91
865	3_33928399	3	33,928,399	160.16	0.19	0.91
866	3_33928991	3	33,928,991	159.21	0.23	0.89
867	3_34149037	3	34,149,037	161.02	0.23	0.89
868	3_34246720	3	34,246,720	161.67	0.17	0.92
869	3_34246773	3	34,246,773	161.53	0.17	0.92
870	3_34247041	3	34,247,041	161.74	0.08	0.96
871	3_34254346	3	34,254,346	161.41	0.08	0.96
872	3_34714089	3	34,714,089	163.88	0.23	0.89
873	3_34729791	3	34,729,791	163.7	0.23	0.89
874	3_34784211	3	34,784,211	164.35	0.19	0.91
875	3_34803090	3	34,803,090	164.35	0.19	0.91
876	3_34810977	3	34,810,977	164.44	0.19	0.91
877	3_34984604	3	34,984,604	166.5	0.23	0.89
878	3_34995681	3	34,995,681	166.76	0.17	0.92

879	3_34995682	3	34,995,682	167.67	0.17	0.92
880	3_34995696	3	34,995,696	166.35	0.23	0.89
881	3_34995719	3	34,995,719	166.71	0.17	0.92
882	3_35003175	3	35,003,175	167.05	0.08	0.96
883	3_35079083	3	35,079,083	167.28	0.36	0.84
884	3_35244559	3	35,244,559	167.67	0.17	0.92
885	3_35404990	3	35,404,990	169.46	0.23	0.89
886	3_35420887	3	35,420,887	169.79	0.17	0.92
887	3_35424095	3	35,424,095	169.67	0.17	0.92
888	3_35425930	3	35,425,930	169.46	0.23	0.89
889	3_35621667	3	35,621,667	170.52	0.02	0.99
890	3_35630635	3	35,630,635	170.29	0.01	1.00
891	3_35641325	3	35,641,325	170.77	0.02	0.99
892	3_35683725	3	35,683,725	170.89	0.02	0.99
893	3_35726455	3	35,726,455	170.75	0.02	0.99
894	3_35996982	3	35,996,982	172.75	0.17	0.92
895	3_36100078	3	36,100,078	174.07	0.86	0.65
896	3_36100087	3	36,100,087	174.13	0.86	0.65
897	3_36272171	3	36,272,171	174.93	0.57	0.75
898	3_36307129	3	36,307,129	174.79	1.18	0.55
899	3_36311183	3	36,311,183	175.4	0.86	0.65
900	3_36406121	3	36,406,121	176.64	0.47	0.79
901	3_36406166	3	36,406,166	176.64	0.47	0.79
902	3_37085428	3	37,085,428	178.84	0.08	0.96
903	3_37136776	3	37,136,776	179.26	0.23	0.89
904	3_37547438	3	37,547,438	181.16	0.57	0.75
905	3_37547633	3	37,547,633	181.21	0.57	0.75
906	3_37584346	3	37,584,346	181.58	0.57	0.75
907	3_37584403	3	37,584,403	181.68	0.57	0.75
908	3_37586895	3	37,586,895	180.77	0.62	0.73
909	3_37637669	3	37,637,669	182.17	0.62	0.73
910	3_37759086	3	37,759,086	183.24	0.47	0.79
911	3_38213275	3	38,213,275	184.68	0.36	0.84
912	3_38213450	3	38,213,450	184.68	0.36	0.84
913	3_38223849	3	38,223,849	184.77	0.36	0.84
914	3_38239066	3	38,239,066	184.64	0.36	0.84
915	3_38351971	3	38,351,971	184.64	0.36	0.84
916	3_38636603	3	38,636,603	185.54	0.36	0.84
917	3_39444373	3	39,444,373	187.95	0.69	0.71
918	3_39583702	3	39,583,702	187.99	0.69	0.71
919	3_39748057	3	39,748,057	188.83	0.69	0.71
920	3_39748100	3	39,748,100	188.92	0.69	0.71
921	3_39938749	3	39,938,749	189.89	1.07	0.59
922	3_39951338	3	39,951,338	189.66	0.75	0.69
923	3_40097920	3	40,097,920	190.72	1.07	0.59
924	3_40103823	3	40,103,823	192.83	2.71	0.26
925	3_40103997	3	40,103,997	193.28	2.71	0.26
926	3_40104054	3	40,104,054	191.13	1.07	0.59
927	3_40164574	3	40,164,574	190.73	1.07	0.59

928	3_40231874	3	40,231,874	190.52	1.07	0.59
929	3_40546082	3	40,546,082	191.13	1.07	0.59
930	4_185571	4	185,571	0	11.71	0.00
931	4_408992	4	408,992	3.21	10.08	0.01
932	4_411179	4	411,179	3.21	9.25	0.01
933	4_738359	4	738,359	5.09	8.92	0.01
934	4_741987	4	741,987	4.53	9.80	0.01
935	4_1140808	4	1,140,808	9.17	11.81	0.00
936	4_1140958	4	1,140,958	9.17	11.81	0.00
937	4_1141071	4	1,141,071	8.65	10.76	0.00
938	4_1158787	4	1,158,787	8.92	11.98	0.00
939	4_1158924	4	1,158,924	8.65	11.98	0.00
940	4_1161506	4	1,161,506	9.7	9.63	0.01
941	4_1166183	4	1,166,183	9.7	10.76	0.00
942	4_1173388	4	1,173,388	9.52	10.65	0.00
943	4_1383122	4	1,383,122	10.86	9.63	0.01
944	4_1416864	4	1,416,864	11.43	9.63	0.01
945	4_1550944	4	1,550,944	12.07	9.63	0.01
946	4_1834612	4	1,834,612	12.54	10.76	0.00
947	4_2142457	4	2,142,457	14.03	9.25	0.01
948	4_2542468	4	2,542,468	17.99	9.25	0.01
949	4_2570124	4	2,570,124	17.92	10.47	0.01
950	4_2764652	4	2,764,652	19.07	9.25	0.01
951	4_2764669	4	2,764,669	18.56	8.51	0.01
952	4_2764815	4	2,764,815	19.01	9.25	0.01
953	4_2794899	4	2,794,899	19.44	8.51	0.01
954	4_3227610	4	3,227,610	21.29	9.70	0.01
955	4_3285372	4	3,285,372	21.46	10.47	0.01
956	4_3287161	4	3,287,161	21.29	8.51	0.01
957	4_3362673	4	3,362,673	20.69	7.41	0.02
958	4_4102604	4	4,102,604	27.91	6.10	0.05
959	4_4104794	4	4,104,794	27.61	5.45	0.07
960	4_4110924	4	4,110,924	29.19	5.94	0.05
961	4_4111024	4	4,111,024	28.88	6.85	0.03
962	4_4158309	4	4,158,309	28.72	6.85	0.03
963	4_4159581	4	4,159,581	28.34	5.94	0.05
964	4_4206774	4	4,206,774	27.61	3.83	0.15
965	4_4228682	4	4,228,682	27.2	5.11	0.08
966	4_4266351	4	4,266,351	25.26	21.32	0.00
967	4_4266477	4	4,266,477	25.22	23.24	0.00
968	4_4666314	4	4,666,314	31.21	5.11	0.08
969	4_4666652	4	4,666,652	31.54	5.11	0.08
970	4_5274514	4	5,274,514	34.95	3.31	0.19
971	4_5444935	4	5,444,935	34.8	4.59	0.10
972	4_5655745	4	5,655,745	36.3	3.83	0.15
973	4_5655847	4	5,655,847	36.39	4.59	0.10
974	4_5768646	4	5,768,646	36.94	4.43	0.11
975	4_5768663	4	5,768,663	37.06	4.43	0.11
976	4_5768902	4	5,768,902	37.48	3.14	0.21

977	4_5846024	4	5,846,024	38.62	2.54	0.28
978	4_5857685	4	5,857,685	38.82	2.54	0.28
979	4_5871248	4	5,871,248	39.02	2.08	0.35
980	4_5871275	4	5,871,275	38.46	3.08	0.21
981	4_5871399	4	5,871,399	38.35	3.71	0.16
982	4_5885870	4	5,885,870	39.64	3.08	0.21
983	4_6178587	4	6,178,587	42.42	2.02	0.36
984	4_6178680	4	6,178,680	42.8	2.54	0.28
985	4_6190112	4	6,190,112	42.07	2.08	0.35
986	4_6190273	4	6,190,273	42.34	2.02	0.36
987	4_6238867	4	6,238,867	41.25	2.65	0.27
988	4_6239398	4	6,239,398	41.12	2.08	0.35
989	4_6253748	4	6,253,748	41.82	1.70	0.43
990	4_6571543	4	6,571,543	41.81	2.24	0.33
991	4_6571684	4	6,571,684	43.16	2.02	0.36
992	4_7022901	4	7,022,901	44.67	2.02	0.36
993	4_7163784	4	7,163,784	44.84	2.54	0.28
994	4_7163873	4	7,163,873	44.67	1.24	0.54
995	4_7173096	4	7,173,096	43.98	1.59	0.45
996	4_7583045	4	7,583,045	47.19	1.59	0.45
997	4_7583145	4	7,583,145	48.36	1.24	0.54
998	4_7618353	4	7,618,353	47	2.02	0.36
999	4_8168537	4	8,168,537	51.25	0.62	0.73
1000	4_8173375	4	8,173,375	51.6	0.47	0.79
1001	4_8728554	4	8,728,554	53.59	0.40	0.82
1002	4_10084155	4	10,084,155	57.09	1.70	0.43
1003	4_10087987	4	10,087,987	56.79	2.24	0.33
1004	4_10145822	4	10,145,822	57.28	2.08	0.35
1005	4_10207727	4	10,207,727	56.63	2.24	0.33
1006	4_10244023	4	10,244,023	56.18	1.92	0.38
1007	4_10395617	4	10,395,617	58.66	3.71	0.16
1008	4_10395768	4	10,395,768	58.01	3.14	0.21
1009	4_13673833	4	13,673,833	60.61	4.37	0.11
1010	4_13743409	4	13,743,409	61.16	3.08	0.21
1011	4_13743498	4	13,743,498	61.26	2.08	0.35
1012	4_14101646	4	14,101,646	63.42	3.08	0.21
1013	4_14127026	4	14,127,026	63.74	2.02	0.36
1014	4_14138031	4	14,138,031	63.49	2.54	0.28
1015	4_14141549	4	14,141,549	63.26	2.54	0.28
1016	4_14251000	4	14,251,000	67.46	1.59	0.45
1017	4_14351658	4	14,351,658	66.89	3.59	0.17
1018	4_14351706	4	14,351,706	66.79	3.59	0.17
1019	4_14352107	4	14,352,107	67.13	1.70	0.43
1020	4_14355840	4	14,355,840	67.8	2.02	0.36
1021	4_14368275	4	14,368,275	64.45	1.18	0.55
1022	4_14386339	4	14,386,339	64.85	1.24	0.54
1023	4_14399269	4	14,399,269	67.61	2.02	0.36
1024	4_14422588	4	14,422,588	65.23	1.59	0.45
1025	4_14422641	4	14,422,641	68.16	1.59	0.45

1026	4_14432464	4	14,432,464	68.33	1.24	0.54
1027	4_14432560	4	14,432,560	65.16	1.59	0.45
1028	4_14442351	4	14,442,351	65.23	1.59	0.45
1029	4_14453279	4	14,453,279	66.12	1.59	0.45
1030	4_14453359	4	14,453,359	65.87	1.59	0.45
1031	4_14453395	4	14,453,395	65.62	2.02	0.36
1032	4_14453445	4	14,453,445	66.52	2.24	0.33
1033	4_14453475	4	14,453,475	66.37	1.70	0.43
1034	4_14540456	4	14,540,456	68.88	0.86	0.65
1035	4_14603918	4	14,603,918	69.6	0.62	0.73
1036	4_14641217	4	14,641,217	70.67	0.97	0.62
1037	4_14690935	4	14,690,935	69.98	0.86	0.65
1038	4_14731311	4	14,731,311	71.31	2.24	0.33
1039	4_14731312	4	14,731,312	71.22	2.24	0.33
1040	4_14756527	4	14,756,527	70.39	0.62	0.73
1041	4_14764310	4	14,764,310	72.41	2.03	0.36
1042	4_14764313	4	14,764,313	72.42	2.03	0.36
1043	4_14764484	4	14,764,484	72.57	1.20	0.55
1044	4_14769456	4	14,769,456	71.74	0.79	0.67
1045	4_14781439	4	14,781,439	70.63	0.97	0.62
1046	4_14903878	4	14,903,878	74.49	1.20	0.55
1047	4_14904144	4	14,904,144	74.21	1.20	0.55
1048	4_15188127	4	15,188,127	77.4	1.20	0.55
1049	4_15188388	4	15,188,388	77.47	1.20	0.55
1050	4_15203515	4	15,203,515	77.51	1.07	0.59
1051	4_15203570	4	15,203,570	77.58	1.07	0.59
1052	4_15213346	4	15,213,346	76.62	0.57	0.75
1053	4_15213350	4	15,213,350	76.88	0.36	0.84
1054	4_15263286	4	15,263,286	78.2	0.47	0.79
1055	4_15559111	4	15,559,111	80.72	0.17	0.92
1056	4_15559207	4	15,559,207	80.72	0.17	0.92
1057	4_15560273	4	15,560,273	81.11	0.23	0.89
1058	4_15587582	4	15,587,582	81.45	0.19	0.91
1059	4_15612988	4	15,612,988	82.39	0.40	0.82
1060	4_15613078	4	15,613,078	82.39	0.40	0.82
1061	4_15613478	4	15,613,478	82.85	0.79	0.67
1062	4_15613509	4	15,613,509	83.06	0.97	0.62
1063	4_15623507	4	15,623,507	82.16	0.19	0.91
1064	4_16280019	4	16,280,019	89.13	0.29	0.87
1065	4_16352114	4	16,352,114	90.72	0.36	0.84
1066	4_16352246	4	16,352,246	89.06	0.13	0.94
1067	4_16402418	4	16,402,418	88.99	0.13	0.94
1068	4_16478324	4	16,478,324	88.3	0.68	0.71
1069	4_16493685	4	16,493,685	90.29	0.06	0.97
1070	4_16493916	4	16,493,916	90.39	0.13	0.94
1071	4_16493923	4	16,493,923	90.62	0.13	0.94
1072	4_16510691	4	16,510,691	89.46	0.36	0.84
1073	4_16510766	4	16,510,766	88.3	0.69	0.71
1074	4_16510856	4	16,510,856	89.87	0.19	0.91

1075	4_16515079	4	16,515,079	88.39	0.23	0.89
1076	4_17205059	4	17,205,059	93.83	0.90	0.64
1077	4_17394480	4	17,394,480	96.02	0.86	0.65
1078	4_17416227	4	17,416,227	96.96	0.96	0.62
1079	4_17416330	4	17,416,330	96.96	0.96	0.62
1080	4_17418291	4	17,418,291	96.7	0.69	0.71
1081	4_17418686	4	17,418,686	96.15	0.86	0.65
1082	4_17676173	4	17,676,173	98.33	0.85	0.66
1083	4_18242735	4	18,242,735	99.78	0.96	0.62
1084	4_18259366	4	18,259,366	99.36	0.86	0.65
1085	4_18285752	4	18,285,752	99.56	1.14	0.57
1086	4_18285948	4	18,285,948	99.73	1.14	0.57
1087	4_18288234	4	18,288,234	99.22	0.57	0.75
1088	4_20819509	4	20,819,509	107.03	1.74	0.42
1089	4_20912038	4	20,912,038	108.21	1.53	0.46
1090	4_20912056	4	20,912,056	108.24	1.53	0.46
1091	4_20980672	4	20,980,672	107.81	1.53	0.46
1092	4_21247181	4	21,247,181	107.66	1.53	0.46
1093	4_21689556	4	21,689,556	109.68	2.31	0.31
1094	4_21689880	4	21,689,880	109.61	2.31	0.31
1095	4_21699749	4	21,699,749	109.1	2.47	0.29
1096	4_21705866	4	21,705,866	108.99	2.71	0.26
1097	4_21707219	4	21,707,219	109.83	2.97	0.23
1098	4_21982300	4	21,982,300	108.99	2.71	0.26
1099	4_22028248	4	22,028,248	111.03	2.75	0.25
1100	4_22104691	4	22,104,691	110.79	2.08	0.35
1101	4_22104881	4	22,104,881	110.79	2.08	0.35
1102	4_22490651	4	22,490,651	112.9	3.43	0.18
1103	4_22494976	4	22,494,976	112.36	3.43	0.18
1104	4_22499162	4	22,499,162	113.38	3.43	0.18
1105	4_22501414	4	22,501,414	112.46	3.43	0.18
1106	4_23167471	4	23,167,471	115.88	2.52	0.28
1107	4_23194239	4	23,194,239	115.89	2.52	0.28
1108	4_23308783	4	23,308,783	117.14	3.92	0.14
1109	4_23324467	4	23,324,467	116.9	3.21	0.20
1110	4_23915410	4	23,915,410	120.17	4.50	0.11
1111	4_23934265	4	23,934,265	120.13	4.50	0.11
1112	4_24323875	4	24,323,875	124.06	2.03	0.36
1113	4_24328683	4	24,328,683	123.21	2.58	0.28
1114	4_24343327	4	24,343,327	123.54	2.03	0.36
1115	4_24432640	4	24,432,640	123.88	2.52	0.28
1116	4_24487335	4	24,487,335	126.49	2.52	0.28
1117	4_24490937	4	24,490,937	125.69	1.63	0.44
1118	4_24491091	4	24,491,091	125.22	1.20	0.55
1119	4_24497831	4	24,497,831	126.33	1.80	0.41
1120	4_24497863	4	24,497,863	126.32	1.80	0.41
1121	4_24891787	4	24,891,787	128.36	2.15	0.34
1122	4_25781223	4	25,781,223	135.36	2.03	0.36
1123	4_25781328	4	25,781,328	135.06	1.57	0.46

1124	4_25804285	4	25,804,285	134.68	1.31	0.52
1125	4_25829453	4	25,829,453	134.69	1.63	0.44
1126	4_25879048	4	25,879,048	135.11	2.03	0.36
1127	4_25960034	4	25,960,034	136.02	0.90	0.64
1128	4_25960080	4	25,960,080	135.98	0.90	0.64
1129	4_26131746	4	26,131,746	136.48	2.03	0.36
1130	4_26174968	4	26,174,968	137.2	0.90	0.64
1131	4_26188071	4	26,188,071	137.14	0.90	0.64
1132	4_26192559	4	26,192,559	139.51	0.01	1.00
1133	4_26192560	4	26,192,560	139.23	0.01	1.00
1134	4_26231562	4	26,231,562	137.74	0.29	0.87
1135	4_26243547	4	26,243,547	137.32	0.90	0.64
1136	4_26249184	4	26,249,184	142.24	0.06	0.97
1137	4_26249310	4	26,249,310	139.67	0.58	0.75
1138	4_26249466	4	26,249,466	139.67	0.58	0.75
1139	4_26251153	4	26,251,153	139.51	0.30	0.86
1140	4_26351799	4	26,351,799	138.03	0.69	0.71
1141	4_26356074	4	26,356,074	137.63	0.30	0.86
1142	4_26367503	4	26,367,503	137.99	0.57	0.75
1143	5_19942	5	19,942	1.69	0.06	0.97
1144	5_52949	5	52,949	1.21	0.06	0.97
1145	5_62768	5	62,768	1.61	0.02	0.99
1146	5_69729	5	69,729	0.54	0.08	0.96
1147	5_93208	5	93,208	1.45	0.06	0.97
1148	5_104789	5	104,789	0	0.19	0.91
1149	5_115234	5	115,234	1.45	0.06	0.97
1150	5_119993	5	119,993	1.06	0.08	0.96
1151	5_240018	5	240,018	2.94	0.02	0.99
1152	5_330823	5	330,823	3.15	0.02	0.99
1153	5_393358	5	393,358	2.34	0.36	0.84
1154	5_393532	5	393,532	2.8	0.23	0.89
1155	5_404121	5	404,121	2.94	0.12	0.94
1156	5_410137	5	410,137	4.17	0.19	0.91
1157	5_540788	5	540,788	4.77	0.19	0.91
1158	5_846533	5	846,533	6.73	0.68	0.71
1159	5_846687	5	846,687	6.6	0.41	0.81
1160	5_855162	5	855,162	6.32	0.06	0.97
1161	5_1053860	5	1,053,860	8.39	0.52	0.77
1162	5_1133443	5	1,133,443	7.97	0.52	0.77
1163	5_1635549	5	1,635,549	10.74	0.34	0.84
1164	5_1710580	5	1,710,580	10.66	0.34	0.84
1165	5_1893048	5	1,893,048	10.06	0.30	0.86
1166	5_1911311	5	1,911,311	11.09	0.58	0.75
1167	5_1946191	5	1,946,191	10.4	0.58	0.75
1168	5_2117964	5	2,117,964	11.59	0.34	0.84
1169	5_2476557	5	2,476,557	14	0.69	0.71
1170	5_2789680	5	2,789,680	15.17	0.13	0.94
1171	5_2839348	5	2,839,348	15.32	0.47	0.79
1172	5_2839635	5	2,839,635	15.17	0.47	0.79

1173	5_2975829	5	2,975,829	16.46	0.19	0.91
1174	5_2992624	5	2,992,624	16.58	0.12	0.94
1175	5_3041888	5	3,041,888	17.31	0.08	0.96
1176	5_3042891	5	3,042,891	17.3	0.02	0.99
1177	5_3282543	5	3,282,543	18.2	0.19	0.91
1178	5_3297117	5	3,297,117	17.5	0.17	0.92
1179	5_3310826	5	3,310,826	17.82	0.06	0.97
1180	5_3320012	5	3,320,012	18.4	0.08	0.96
1181	5_3322795	5	3,322,795	17.22	0.02	0.99
1182	5_3327104	5	3,327,104	18.09	0.19	0.91
1183	5_3392693	5	3,392,693	19.31	0.47	0.79
1184	5_3686608	5	3,686,608	21.12	0.19	0.91
1185	5_3724192	5	3,724,192	20.58	0.19	0.91
1186	5_3736397	5	3,736,397	21.31	0.06	0.97
1187	5_3778475	5	3,778,475	20.92	0.40	0.82
1188	5_3780171	5	3,780,171	20.79	0.40	0.82
1189	5_3926697	5	3,926,697	22.14	0.40	0.82
1190	5_3931000	5	3,931,000	22.36	0.69	0.71
1191	5_4051433	5	4,051,433	22.41	0.69	0.71
1192	5_4238917	5	4,238,917	24.38	0.47	0.79
1193	5_4282880	5	4,282,880	25.65	0.68	0.71
1194	5_4293953	5	4,293,953	25.16	0.69	0.71
1195	5_4295246	5	4,295,246	25.5	0.68	0.71
1196	5_4305625	5	4,305,625	24.72	0.40	0.82
1197	5_4329338	5	4,329,338	24.76	0.40	0.82
1198	5_4535463	5	4,535,463	27.56	0.41	0.81
1199	5_4554819	5	4,554,819	27.54	0.41	0.81
1200	5_4579792	5	4,579,792	28.48	0.40	0.82
1201	5_4585625	5	4,585,625	28.11	0.69	0.71
1202	5_4737241	5	4,737,241	29.12	0.68	0.71
1203	5_5010082	5	5,010,082	30	1.46	0.48
1204	5_5126399	5	5,126,399	31.91	1.07	0.59
1205	5_5138316	5	5,138,316	31.23	1.48	0.48
1206	5_5217603	5	5,217,603	32.68	1.48	0.48
1207	5_5218053	5	5,218,053	31.52	1.07	0.59
1208	5_5219383	5	5,219,383	32.58	1.07	0.59
1209	5_5229739	5	5,229,739	32.37	1.20	0.55
1210	5_5455402	5	5,455,402	35.89	3.21	0.20
1211	5_5456252	5	5,456,252	34.63	2.03	0.36
1212	5_5461336	5	5,461,336	35.89	3.92	0.14
1213	5_5461498	5	5,461,498	34.39	2.03	0.36
1214	5_5467357	5	5,467,357	33.92	1.97	0.37
1215	5_5467417	5	5,467,417	34.16	2.54	0.28
1216	5_6403064	5	6,403,064	39.33	3.21	0.20
1217	5_6433530	5	6,433,530	40.19	2.87	0.24
1218	5_6436767	5	6,436,767	39.57	2.52	0.28
1219	5_6437643	5	6,437,643	39.74	2.52	0.28
1220	5_6811207	5	6,811,207	42.34	2.92	0.23
1221	5_6870590	5	6,870,590	41.19	2.87	0.24

1222	5_6870716	5	6,870,716	41.6	2.87	0.24
1223	5_6870717	5	6,870,717	41.4	2.87	0.24
1224	5_6889298	5	6,889,298	42.03	2.26	0.32
1225	5_6902941	5	6,902,941	41.82	2.52	0.28
1226	5_9087011	5	9,087,011	47.48	2.58	0.28
1227	5_9194546	5	9,194,546	47.37	1.98	0.37
1228	5_11523901	5	11,523,901	52.05	0.41	0.81
1229	5_11755588	5	11,755,588	53.59	0.19	0.91
1230	5_11755612	5	11,755,612	54	0.01	1.00
1231	5_11841275	5	11,841,275	61.39	0.40	0.82
1232	5_11841790	5	11,841,790	54.88	0.13	0.94
1233	5_12287827	5	12,287,827	61.07	0.57	0.75
1234	5_12376878	5	12,376,878	59.37	0.85	0.66
1235	5_12381787	5	12,381,787	60.37	0.19	0.91
1236	5_12381865	5	12,381,865	60.55	0.19	0.91
1237	5_12382046	5	12,382,046	60.26	0.13	0.94
1238	5_12382191	5	12,382,191	54.88	0.52	0.77
1239	5_12382356	5	12,382,356	58.55	0.40	0.82
1240	5_12382382	5	12,382,382	59.15	0.85	0.66
1241	5_12382428	5	12,382,428	59.54	0.90	0.64
1242	5_12695147	5	12,695,147	55.61	0.57	0.75
1243	5_12911761	5	12,911,761	56.25	0.51	0.77
1244	5_12911810	5	12,911,810	56.11	0.51	0.77
1245	5_12911820	5	12,911,820	56.2	0.51	0.77
1246	5_12911857	5	12,911,857	56.04	0.36	0.84
1247	5_12918083	5	12,918,083	57.11	0.57	0.75
1248	5_12921097	5	12,921,097	57.77	0.36	0.84
1249	5_12921224	5	12,921,224	57.59	0.36	0.84
1250	5_12926184	5	12,926,184	58.1	0.12	0.94
1251	5_13032265	5	13,032,265	56.9	0.57	0.75
1252	5_13701788	5	13,701,788	62.11	0.29	0.87
1253	5_14116630	5	14,116,630	63.75	0.29	0.87
1254	5_14342413	5	14,342,413	63.22	0.30	0.86
1255	5_14342558	5	14,342,558	63.03	0.12	0.94
1256	5_18324437	5	18,324,437	67.11	0.85	0.66
1257	5_20014870	5	20,014,870	71.55	0.06	0.97
1258	5_20015134	5	20,015,134	70.5	0.06	0.97
1259	5_21262634	5	21,262,634	76.03	1.35	0.51
1260	5_21531051	5	21,531,051	77.65	0.92	0.63
1261	5_21840436	5	21,840,436	78.97	1.63	0.44
1262	5_21841655	5	21,841,655	79.42	0.85	0.66
1263	5_24162975	5	24,162,975	89.14	0.86	0.65
1264	5_24162980	5	24,162,980	89.62	1.24	0.54
1265	5_24169934	5	24,169,934	87.99	0.13	0.94
1266	5_24736893	5	24,736,893	92.26	1.70	0.43
1267	5_24868025	5	24,868,025	93.57	2.02	0.36
1268	5_24993062	5	24,993,062	94.77	3.21	0.20
1269	5_25028943	5	25,028,943	94	2.75	0.25
1270	5_25039810	5	25,039,810	94.4	2.75	0.25

1271	5_25043620	5	25,043,620	94.43	2.75	0.25
1272	5_25049600	5	25,049,600	94.26	2.75	0.25
1273	5_25583469	5	25,583,469	97.57	2.43	0.30
1274	5_25591294	5	25,591,294	97.26	2.02	0.36
1275	5_25602257	5	25,602,257	96.83	2.43	0.30
1276	5_25612519	5	25,612,519	97.94	2.43	0.30
1277	5_25613633	5	25,613,633	97.2	2.02	0.36
1278	5_25820890	5	25,820,890	98.92	2.71	0.26
1279	5_25820896	5	25,820,896	98.86	2.71	0.26
1280	5_25834090	5	25,834,090	98.47	2.02	0.36
1281	5_25931839	5	25,931,839	99.74	1.14	0.57
1282	5_25931913	5	25,931,913	99.67	1.46	0.48
1283	5_26449299	5	26,449,299	103.97	0.51	0.77
1284	5_26525660	5	26,525,660	104.52	0.75	0.69
1285	5_26533486	5	26,533,486	103.97	0.51	0.77
1286	5_26545429	5	26,545,429	104.33	0.69	0.71
1287	5_26713176	5	26,713,176	105.01	0.68	0.71
1288	5_26718472	5	26,718,472	104.85	0.68	0.71
1289	5_26804403	5	26,804,403	103.34	0.40	0.82
1290	5_26807520	5	26,807,520	105.12	1.03	0.60
1291	5_26823550	5	26,823,550	105.71	1.53	0.46
1292	5_26845053	5	26,845,053	103.97	0.41	0.81
1293	5_26869126	5	26,869,126	105.27	1.46	0.48
1294	5_26880474	5	26,880,474	103.93	0.41	0.81
1295	5_27050960	5	27,050,960	107.97	0.41	0.81
1296	5_27050979	5	27,050,979	107.98	0.41	0.81
1297	5_27054606	5	27,054,606	108.1	0.23	0.89
1298	5_27064857	5	27,064,857	106.28	1.03	0.60
1299	5_27073539	5	27,073,539	107.43	0.68	0.71
1300	5_27073843	5	27,073,843	107.65	0.68	0.71
1301	5_27098561	5	27,098,561	107.15	0.68	0.71
1302	5_27098774	5	27,098,774	106.53	1.03	0.60
1303	5_27101679	5	27,101,679	107.19	0.68	0.71
1304	5_27164074	5	27,164,074	106.65	1.03	0.60
1305	5_27211151	5	27,211,151	107.1	1.03	0.60
1306	5_27212024	5	27,212,024	108.49	0.41	0.81
1307	5_27296600	5	27,296,600	108.49	0.68	0.71
1308	5_28380512	5	28,380,512	111.69	2.58	0.28
1309	5_28380581	5	28,380,581	111.71	1.98	0.37
1310	5_28447330	5	28,447,330	112.9	0.68	0.71
1311	5_28450968	5	28,450,968	112.37	2.71	0.26
1312	5_28451004	5	28,451,004	112.37	2.71	0.26
1313	5_28457533	5	28,457,533	110.82	1.20	0.55
1314	5_28504478	5	28,504,478	113.27	1.07	0.59
1315	5_28504514	5	28,504,514	109.39	1.07	0.59
1316	5_28536104	5	28,536,104	113.6	1.57	0.46
1317	5_28538543	5	28,538,543	111.04	1.57	0.46
1318	5_28555203	5	28,555,203	110.43	1.57	0.46
1319	5_28586898	5	28,586,898	109.95	1.48	0.48

1320	5_28587006	5	28,587,006	110.14	1.48	0.48
1321	5_28587464	5	28,587,464	114.09	2.03	0.36
1322	5_28593833	5	28,593,833	109.69	1.48	0.48
1323	5_28611067	5	28,611,067	114.29	1.57	0.46
1324	5_28629278	5	28,629,278	110.32	2.03	0.36
1325	5_28694206	5	28,694,206	116.34	0.75	0.69
1326	5_28699538	5	28,699,538	114.88	1.57	0.46
1327	5_28700954	5	28,700,954	115.51	1.20	0.55
1328	5_28706664	5	28,706,664	116.24	0.68	0.71
1329	5_28714177	5	28,714,177	116.03	1.07	0.59
1330	5_28728983	5	28,728,983	115.9	1.20	0.55
1331	5_28730946	5	28,730,946	115.8	1.20	0.55
1332	5_28748681	5	28,748,681	115.73	1.20	0.55
1333	5_28784667	5	28,784,667	116.14	0.75	0.69
1334	5_28788287	5	28,788,287	115.99	1.20	0.55
1335	5_28795242	5	28,795,242	115.1	1.57	0.46
1336	5_29002926	5	29,002,926	117.49	1.14	0.57
1337	5_29022783	5	29,022,783	118.11	0.75	0.69
1338	5_29022841	5	29,022,841	118.16	0.75	0.69
1339	5_29033337	5	29,033,337	120.62	1.41	0.50
1340	5_29045825	5	29,045,825	118.42	0.86	0.65
1341	5_29049648	5	29,049,648	118.26	0.90	0.64
1342	5_29050414	5	29,050,414	117.68	0.96	0.62
1343	5_29053560	5	29,053,560	122.51	0.41	0.81
1344	5_29053681	5	29,053,681	117.42	1.41	0.50
1345	5_29081188	5	29,081,188	117.88	1.14	0.57
1346	5_29096504	5	29,096,504	116.91	1.41	0.50
1347	5_29096636	5	29,096,636	116.79	1.14	0.57
1348	5_29097013	5	29,097,013	125.46	0.57	0.75
1349	5_29110431	5	29,110,431	121.48	1.76	0.42
1350	5_29110740	5	29,110,740	121.21	1.76	0.42
1351	5_29112220	5	29,112,220	121.81	1.41	0.50
1352	5_29167051	5	29,167,051	120.53	1.20	0.55
1353	5_29169080	5	29,169,080	124.02	0.51	0.77
1354	5_29173123	5	29,173,123	120.3	1.20	0.55
1355	5_29191089	5	29,191,089	125.71	0.57	0.75
1356	5_29193443	5	29,193,443	125.22	0.23	0.89
1357	5_29214883	5	29,214,883	124.5	0.68	0.71
1358	5_29297791	5	29,297,791	123.18	0.23	0.89
1359	5_29298218	5	29,298,218	123.16	0.23	0.89
1360	5_29316282	5	29,316,282	119.38	0.19	0.91
1361	5_29316382	5	29,316,382	119.64	0.40	0.82
1362	5_29379817	5	29,379,817	126.36	0.51	0.77
1363	5_29379994	5	29,379,994	124.02	0.41	0.81
1364	5_29416654	5	29,416,654	124.55	0.68	0.71
1365	5_29416835	5	29,416,835	120.07	0.75	0.69
1366	5_29492830	5	29,492,830	125.04	0.40	0.82
1367	5_29674223	5	29,674,223	127.05	0.23	0.89
1368	5_29698365	5	29,698,365	128.34	0.68	0.71

1369	5_29968666	5	29,968,666	127.79	0.68	0.71
1370	5_30007257	5	30,007,257	128.95	0.19	0.91
1371	5_30008711	5	30,008,711	127.61	0.41	0.81
1372	5_30013360	5	30,013,360	128.1	1.03	0.60
1373	5_30031643	5	30,031,643	128.19	1.03	0.60
1374	5_30069055	5	30,069,055	128.57	0.40	0.82
1375	5_30133255	5	30,133,255	129.27	0.08	0.96
1376	5_30133403	5	30,133,403	127.79	0.68	0.71
1377	5_30868598	5	30,868,598	131.01	0.13	0.94
1378	5_30872293	5	30,872,293	131.34	0.41	0.81
1379	5_30900092	5	30,900,092	130.64	0.13	0.94
1380	5_30904356	5	30,904,356	132.45	0.75	0.69
1381	5_30922652	5	30,922,652	132.9	0.68	0.71
1382	5_31371242	5	31,371,242	132.03	0.75	0.69
1383	5_31371288	5	31,371,288	131.82	1.07	0.59
1384	5_31380502	5	31,380,502	132.34	0.51	0.77
1385	5_31496281	5	31,496,281	133.23	0.19	0.91
1386	5_31629569	5	31,629,569	135.36	0.52	0.77
1387	5_31643597	5	31,643,597	136.06	0.57	0.75
1388	5_31648555	5	31,648,555	145.19	18.52	0.00
1389	5_31681895	5	31,681,895	139.52	0.06	0.97
1390	5_31749588	5	31,749,588	135.36	0.85	0.66
1391	5_31749711	5	31,749,711	134.86	0.52	0.77
1392	5_31820579	5	31,820,579	134.36	0.13	0.94
1393	5_31820625	5	31,820,625	134.53	0.06	0.97
1394	5_31828425	5	31,828,425	135.36	0.52	0.77
1395	5_31828593	5	31,828,593	134.86	0.29	0.87
1396	6_194056	6	194,056	15.48	1.24	0.54
1397	6_194241	6	194,241	15.5	1.24	0.54
1398	6_340830	6	340,830	15.65	1.24	0.54
1399	6_343528	6	343,528	15.02	1.74	0.42
1400	6_343639	6	343,639	13.87	2.26	0.32
1401	6_343647	6	343,647	14.19	2.31	0.31
1402	6_351815	6	351,815	14.8	1.74	0.42
1403	6_455748	6	455,748	14.8	1.74	0.42
1404	6_455864	6	455,864	14.87	1.74	0.42
1405	6_457739	6	457,739	13.53	2.26	0.32
1406	6_637207	6	637,207	17.04	1.74	0.42
1407	6_652763	6	652,763	17.43	1.74	0.42
1408	6_653996	6	653,996	17.33	1.74	0.42
1409	6_663669	6	663,669	17.67	2.26	0.32
1410	6_793224	6	793,224	18.64	1.31	0.52
1411	6_810791	6	810,791	9.05	2.80	0.25
1412	6_940313	6	940,313	19.12	1.24	0.54
1413	6_1558004	6	1,558,004	22.7	0.23	0.89
1414	6_1558027	6	1,558,027	22.8	0.23	0.89
1415	6_1558485	6	1,558,485	23.99	0.23	0.89
1416	6_1619071	6	1,619,071	23.44	0.23	0.89
1417	6_1619206	6	1,619,206	22.51	0.23	0.89

1418	6_1668421	6	1,668,421	22.15	0.19	0.91
1419	6_1674909	6	1,674,909	21.92	0.19	0.91
1420	6_1675098	6	1,675,098	21.31	0.19	0.91
1421	6_1844747	6	1,844,747	0.47	21.95	0.00
1422	6_1844757	6	1,844,757	0	21.95	0.00
1423	6_1844774	6	1,844,774	1.17	21.94	0.00
1424	6_1925225	6	1,925,225	24.26	0.13	0.94
1425	6_1971959	6	1,971,959	23.56	0.23	0.89
1426	6_2087152	6	2,087,152	26.6	0.02	0.99
1427	6_2103831	6	2,103,831	25.95	0.06	0.97
1428	6_2111120	6	2,111,120	25.59	0.02	0.99
1429	6_2111221	6	2,111,221	25.59	0.02	0.99
1430	6_2134445	6	2,134,445	26.28	0.02	0.99
1431	6_2210171	6	2,210,171	29.45	2.87	0.24
1432	6_2210317	6	2,210,317	29.45	2.87	0.24
1433	6_2210378	6	2,210,378	29.45	2.87	0.24
1434	6_2210406	6	2,210,406	27.94	0.08	0.96
1435	6_2214688	6	2,214,688	27.1	0.08	0.96
1436	6_2215679	6	2,215,679	25.27	0.02	0.99
1437	6_2215703	6	2,215,703	25.29	0.02	0.99
1438	6_2215863	6	2,215,863	27.34	0.08	0.96
1439	6_2225626	6	2,225,626	28.64	0.23	0.89
1440	6_2242174	6	2,242,174	28.24	0.23	0.89
1441	6_2555151	6	2,555,151	27.53	0.08	0.96
1442	6_3102548	6	3,102,548	31.71	0.69	0.71
1443	6_3137031	6	3,137,031	32.32	0.52	0.77
1444	6_3137188	6	3,137,188	32.37	0.52	0.77
1445	6_3137211	6	3,137,211	32.02	0.57	0.75
1446	6_3137348	6	3,137,348	31.34	0.90	0.64
1447	6_3138534	6	3,138,534	33.22	0.52	0.77
1448	6_3138672	6	3,138,672	33.11	0.52	0.77
1449	6_3160436	6	3,160,436	33.55	0.52	0.77
1450	6_3160505	6	3,160,505	34.48	0.57	0.75
1451	6_3399913	6	3,399,913	32.89	0.52	0.77
1452	6_3782095	6	3,782,095	36.84	0.13	0.94
1453	6_3782186	6	3,782,186	36.84	0.13	0.94
1454	6_3785664	6	3,785,664	36.73	0.13	0.94
1455	6_3808119	6	3,808,119	37.31	0.29	0.87
1456	6_4261491	6	4,261,491	40.39	0.06	0.97
1457	6_4412775	6	4,412,775	41.33	0.06	0.97
1458	6_4551921	6	4,551,921	44.2	0.23	0.89
1459	6_4570324	6	4,570,324	44.17	0.08	0.96
1460	6_4870815	6	4,870,815	45.47	0.57	0.75
1461	6_4956061	6	4,956,061	45.15	0.86	0.65
1462	6_4956089	6	4,956,089	45.15	0.86	0.65
1463	6_4959872	6	4,959,872	47.15	0.36	0.84
1464	6_4960165	6	4,960,165	47.06	0.36	0.84
1465	6_4965263	6	4,965,263	46.54	0.36	0.84
1466	6_4978694	6	4,978,694	46.19	0.36	0.84

1467	6_5001791	6	5,001,791	46.75	0.36	0.84
1468	6_5002206	6	5,002,206	46.38	0.36	0.84
1469	6_5002580	6	5,002,580	46.64	0.36	0.84
1470	6_5006668	6	5,006,668	45.56	0.57	0.75
1471	6_5047764	6	5,047,764	46.29	0.36	0.84
1472	6_5309302	6	5,309,302	49.64	0.23	0.89
1473	6_5320090	6	5,320,090	49.36	0.23	0.89
1474	6_5320406	6	5,320,406	49.15	0.19	0.91
1475	6_5328448	6	5,328,448	49.03	0.19	0.91
1476	6_5328536	6	5,328,536	48.93	0.19	0.91
1477	6_5347011	6	5,347,011	48.7	0.19	0.91
1478	6_5450309	6	5,450,309	50.15	0.19	0.91
1479	6_5566465	6	5,566,465	52.23	0.69	0.71
1480	6_5578228	6	5,578,228	52.35	0.69	0.71
1481	6_5614376	6	5,614,376	52.56	0.69	0.71
1482	6_5617652	6	5,617,652	51.71	1.03	0.60
1483	6_5617897	6	5,617,897	51.67	1.03	0.60
1484	6_5617987	6	5,617,987	51.58	0.68	0.71
1485	6_5618014	6	5,618,014	51.8	1.07	0.59
1486	6_5618053	6	5,618,053	52.2	0.69	0.71
1487	6_5935118	6	5,935,118	53.65	0.52	0.77
1488	6_5935190	6	5,935,190	53.58	0.52	0.77
1489	6_6178240	6	6,178,240	56.19	0.23	0.89
1490	6_6178739	6	6,178,739	54.24	0.06	0.97
1491	6_6198924	6	6,198,924	54.89	0.06	0.97
1492	6_6198972	6	6,198,972	54.57	0.06	0.97
1493	6_6251720	6	6,251,720	55.58	0.13	0.94
1494	6_6254764	6	6,254,764	55.37	0.13	0.94
1495	6_6261488	6	6,261,488	55.2	0.12	0.94
1496	6_6533905	6	6,533,905	57.92	0.68	0.71
1497	6_7170104	6	7,170,104	61.68	4.72	0.09
1498	6_7246382	6	7,246,382	62.07	4.72	0.09
1499	6_7323557	6	7,323,557	62.07	4.72	0.09
1500	6_7445117	6	7,445,117	62.32	3.92	0.14
1501	6_7562939	6	7,562,939	63.03	4.76	0.09
1502	6_8993176	6	8,993,176	70.43	1.41	0.50
1503	6_8993317	6	8,993,317	69.91	1.41	0.50
1504	6_8993454	6	8,993,454	69.85	1.41	0.50
1505	6_9034743	6	9,034,743	77.99	1.59	0.45
1506	6_9034837	6	9,034,837	71.2	1.57	0.46
1507	6_9034878	6	9,034,878	71.07	1.57	0.46
1508	6_9040033	6	9,040,033	71.37	1.76	0.42
1509	6_9359366	6	9,359,366	72.55	0.90	0.64
1510	6_9563241	6	9,563,241	73.32	1.41	0.50
1511	6_9563486	6	9,563,486	73.02	1.20	0.55
1512	6_9566200	6	9,566,200	73.02	1.20	0.55
1513	6_9581279	6	9,581,279	73.54	1.41	0.50
1514	6_9581287	6	9,581,287	73.59	1.41	0.50
1515	6_9585961	6	9,585,961	74.24	1.57	0.46

1516	6_9597717	6	9,597,717	73.73	1.41	0.50
1517	6_9849708	6	9,849,708	75.78	1.03	0.60
1518	6_9849799	6	9,849,799	76.22	1.20	0.55
1519	6_9856179	6	9,856,179	77.14	1.70	0.43
1520	6_9856292	6	9,856,292	76.51	1.41	0.50
1521	6_9874957	6	9,874,957	75.43	1.46	0.48
1522	6_9875180	6	9,875,180	75.19	1.46	0.48
1523	6_10025179	6	10,025,179	78.44	1.18	0.55
1524	6_10025183	6	10,025,183	78.67	0.86	0.65
1525	6_10128885	6	10,128,885	81.91	1.92	0.38
1526	6_10175020	6	10,175,020	82.64	2.87	0.24
1527	6_10200792	6	10,200,792	89.56	5.27	0.07
1528	6_10200809	6	10,200,809	89.59	5.27	0.07
1529	6_10200842	6	10,200,842	79.52	1.41	0.50
1530	6_10212524	6	10,212,524	82.13	2.52	0.28
1531	6_10222667	6	10,222,667	82.15	2.52	0.28
1532	6_10222740	6	10,222,740	81	1.92	0.38
1533	6_10269922	6	10,269,922	81.46	1.92	0.38
1534	6_10281453	6	10,281,453	90	3.59	0.17
1535	6_10287235	6	10,287,235	81.67	1.92	0.38
1536	6_10315805	6	10,315,805	81.21	1.92	0.38
1537	6_10324539	6	10,324,539	82.3	2.52	0.28
1538	6_10340869	6	10,340,869	92.4	1.41	0.50
1539	6_10383204	6	10,383,204	80.08	1.41	0.50
1540	6_10405882	6	10,405,882	86.35	1.24	0.54
1541	6_10409199	6	10,409,199	91.94	1.41	0.50
1542	6_10434838	6	10,434,838	85.74	2.02	0.36
1543	6_10434866	6	10,434,866	86.18	1.59	0.45
1544	6_10434977	6	10,434,977	85.49	2.02	0.36
1545	6_10441244	6	10,441,244	90.97	2.08	0.35
1546	6_10443737	6	10,443,737	80.39	1.92	0.38
1547	6_10509390	6	10,509,390	91.33	1.70	0.43
1548	6_10510784	6	10,510,784	91.73	1.70	0.43
1549	6_10519930	6	10,519,930	83.33	1.70	0.43
1550	6_10531226	6	10,531,226	80.85	1.41	0.50
1551	6_10561076	6	10,561,076	87.97	8.41	0.01
1552	6_10561095	6	10,561,095	88.07	7.28	0.03
1553	6_10561120	6	10,561,120	88.12	7.28	0.03
1554	6_10561189	6	10,561,189	88.11	7.28	0.03
1555	6_10562260	6	10,562,260	87.01	2.24	0.33
1556	6_10563288	6	10,563,288	90.54	2.65	0.27
1557	6_10609210	6	10,609,210	83.63	2.08	0.35
1558	6_10903761	6	10,903,761	84.16	3.08	0.21
1559	6_10911949	6	10,911,949	93.23	2.24	0.33
1560	6_10930025	6	10,930,025	84.25	2.54	0.28
1561	6_10938057	6	10,938,057	84.88	2.02	0.36
1562	6_10938062	6	10,938,062	84.56	2.54	0.28
1563	6_10938219	6	10,938,219	84.53	2.54	0.28
1564	6_11053713	6	11,053,713	94.33	2.87	0.24

1565	6_11053746	6	11,053,746	94.23	2.87	0.24
1566	6_11064331	6	11,064,331	94.18	2.87	0.24
1567	6_11605299	6	11,605,299	96.31	1.69	0.43
1568	6_11605710	6	11,605,710	95.76	1.92	0.38
1569	6_11822728	6	11,822,728	95.59	1.92	0.38
1570	6_11837991	6	11,837,991	96.04	1.92	0.38
1571	6_11871505	6	11,871,505	95.66	1.92	0.38
1572	6_12341953	6	12,341,953	98.61	3.66	0.16
1573	6_12342109	6	12,342,109	98.5	3.66	0.16
1574	6_12664576	6	12,664,576	99.05	3.42	0.18
1575	6_12683551	6	12,683,551	98.26	3.66	0.16
1576	6_13287866	6	13,287,866	99.42	4.22	0.12
1577	6_13467168	6	13,467,168	99.98	5.77	0.06
1578	6_13467261	6	13,467,261	100.18	6.23	0.04
1579	6_15884981	6	15,884,981	102.64	7.84	0.02
1580	6_16762009	6	16,762,009	103.05	6.38	0.04
1581	6_17333171	6	17,333,171	103.52	5.45	0.07
1582	6_17333402	6	17,333,402	103.5	5.45	0.07
1583	6_17757034	6	17,757,034	104.44	6.10	0.05
1584	6_17757038	6	17,757,038	103.98	6.10	0.05
1585	6_17769776	6	17,769,776	106.13	7.07	0.03
1586	6_17781607	6	17,781,607	104.85	7.67	0.02
1587	6_17781800	6	17,781,800	104.72	6.85	0.03
1588	6_17788093	6	17,788,093	104.67	6.10	0.05
1589	6_17796594	6	17,796,594	105.93	7.07	0.03
1590	6_17796603	6	17,796,603	105.9	7.07	0.03
1591	6_17870457	6	17,870,457	104.08	6.10	0.05
1592	6_17885200	6	17,885,200	104.32	6.10	0.05
1593	6_18214279	6	18,214,279	105.71	7.84	0.02
1594	6_18226524	6	18,226,524	106.71	5.22	0.07
1595	6_18226554	6	18,226,554	106.46	5.22	0.07
1596	6_18333853	6	18,333,853	105.9	7.07	0.03
1597	6_18333889	6	18,333,889	107.03	4.59	0.10
1598	6_18414429	6	18,414,429	105.66	6.85	0.03
1599	6_18414479	6	18,414,479	105.39	6.73	0.03
1600	6_18929642	6	18,929,642	108.59	3.08	0.21
1601	6_19159725	6	19,159,725	109.49	3.08	0.21
1602	6_20313945	6	20,313,945	114.04	1.41	0.50
1603	6_20317016	6	20,317,016	114.21	1.41	0.50
1604	6_20324169	6	20,324,169	113.77	1.70	0.43
1605	6_20325569	6	20,325,569	113.44	1.70	0.43
1606	6_20366910	6	20,366,910	115.51	1.20	0.55
1607	6_20366954	6	20,366,954	115.58	1.20	0.55
1608	6_20384843	6	20,384,843	114.95	1.20	0.55
1609	6_20390494	6	20,390,494	115.24	1.20	0.55
1610	6_20393360	6	20,393,360	114.4	1.41	0.50
1611	6_20924265	6	20,924,265	118.29	1.97	0.37
1612	6_20924350	6	20,924,350	118.44	1.48	0.48
1613	6_20924383	6	20,924,383	118.54	1.48	0.48

1614	6_20970341	6	20,970,341	118.67	1.48	0.48
1615	6_21109210	6	21,109,210	120.38	2.92	0.23
1616	6_21192633	6	21,192,633	120.56	2.92	0.23
1617	6_21398464	6	21,398,464	122.2	4.59	0.10
1618	6_21404304	6	21,404,304	122.55	4.59	0.10
1619	6_21413157	6	21,413,157	122.2	4.59	0.10
1620	6_21626270	6	21,626,270	124.68	6.44	0.04
1621	6_21628855	6	21,628,855	124.68	6.44	0.04
1622	6_21631387	6	21,631,387	124.68	6.44	0.04
1623	6_21762821	6	21,762,821	127.38	8.40	0.02
1624	6_21837524	6	21,837,524	125.96	7.97	0.02
1625	6_21842582	6	21,842,582	126.34	8.40	0.02
1626	6_21842598	6	21,842,598	126.72	8.40	0.02
1627	6_21843648	6	21,843,648	126.11	7.97	0.02
1628	6_21867578	6	21,867,578	127.03	8.40	0.02
1629	6_21869180	6	21,869,180	126.59	8.40	0.02
1630	6_21869473	6	21,869,473	126.62	8.40	0.02
1631	6_21895036	6	21,895,036	126.23	7.97	0.02
1632	6_21956705	6	21,956,705	127.95	9.52	0.01
1633	6_21956770	6	21,956,770	128.1	9.52	0.01
1634	6_22178946	6	22,178,946	130.4	9.80	0.01
1635	6_22699562	6	22,699,562	131.5	9.31	0.01
1636	6_22784302	6	22,784,302	133.38	9.52	0.01
1637	6_22784319	6	22,784,319	133.38	9.52	0.01
1638	6_22788421	6	22,788,421	133.21	9.52	0.01
1639	6_22789693	6	22,789,693	134.64	9.31	0.01
1640	6_22913222	6	22,913,222	132.07	9.80	0.01
1641	6_23016661	6	23,016,661	132.21	9.80	0.01
1642	6_23044992	6	23,044,992	134.25	9.80	0.01
1643	6_23070429	6	23,070,429	134.49	9.31	0.01
1644	6_23093662	6	23,093,662	132.97	9.80	0.01
1645	6_23147658	6	23,147,658	134.35	9.31	0.01
1646	6_23236811	6	23,236,811	135.57	9.92	0.01
1647	6_23252906	6	23,252,906	135.56	9.92	0.01
1648	6_23265358	6	23,265,358	136.51	9.63	0.01
1649	6_23277454	6	23,277,454	137.4	9.92	0.01
1650	6_23303961	6	23,303,961	137.39	10.30	0.01
1651	6_23303984	6	23,303,984	137.39	10.30	0.01
1652	6_23366828	6	23,366,828	136.16	9.63	0.01
1653	6_23374914	6	23,374,914	137.05	9.92	0.01
1654	6_23432445	6	23,432,445	136.95	9.92	0.01
1655	6_23549190	6	23,549,190	139.12	11.31	0.00
1656	6_23549401	6	23,549,401	139.57	10.37	0.01
1657	6_23549414	6	23,549,414	139.4	10.37	0.01
1658	6_23562654	6	23,562,654	138.76	11.31	0.00
1659	6_23870074	6	23,870,074	140.71	13.43	0.00
1660	6_23870211	6	23,870,211	140.5	12.33	0.00
1661	6_24132776	6	24,132,776	142.89	15.80	0.00
1662	6_24572240	6	24,572,240	145.58	18.97	0.00

1663	6_24623145	6	24,623,145	146.46	19.70	0.00
1664	6_24627533	6	24,627,533	145.64	18.31	0.00
1665	6_24627600	6	24,627,600	145.64	19.70	0.00
1666	6_24657001	6	24,657,001	146.14	19.70	0.00
1667	6_25799486	6	25,799,486	149.87	24.08	0.00
1668	6_25810464	6	25,810,464	149.53	25.52	0.00
1669	6_25825565	6	25,825,565	149.53	25.52	0.00
1670	6_25832359	6	25,832,359	149.87	25.52	0.00
1671	6_25832826	6	25,832,826	150.23	24.08	0.00
1672	6_25832865	6	25,832,865	149.97	24.08	0.00
1673	6_25894802	6	25,894,802	150.68	25.52	0.00
1674	6_26141589	6	26,141,589	152.7	28.89	0.00
1675	6_26147197	6	26,147,197	151.99	27.42	0.00
1676	6_26157497	6	26,157,497	153.14	29.94	0.00
1677	6_26274428	6	26,274,428	151.83	27.42	0.00
1678	6_26281957	6	26,281,957	152.32	27.42	0.00
1679	6_26310762	6	26,310,762	154.26	34.97	0.00
1680	6_26310849	6	26,310,849	154.39	34.97	0.00
1681	6_26310860	6	26,310,860	154.39	34.97	0.00
1682	6_26312563	6	26,312,563	152.79	28.89	0.00
1683	6_26826643	6	26,826,643	157.17	33.92	0.00
1684	6_26833875	6	26,833,875	157.52	32.40	0.00
1685	6_26833961	6	26,833,961	157.8	32.40	0.00
1686	6_26893647	6	26,893,647	158.14	33.92	0.00
1687	6_27121331	6	27,121,331	158.7	34.43	0.00
1688	6_27151952	6	27,151,952	159.18	36.15	0.00
1689	6_27160467	6	27,160,467	159.56	36.15	0.00
1690	6_27180375	6	27,180,375	159.24	36.15	0.00
1691	6_27507068	6	27,507,068	161.55	29.77	0.00
1692	6_27638879	6	27,638,879	163.34	30.96	0.00
1693	6_27639644	6	27,639,644	162.7	29.77	0.00
1694	6_27653151	6	27,653,151	163.15	29.77	0.00
1695	6_27653282	6	27,653,282	162.93	29.77	0.00
1696	6_28262683	6	28,262,683	165.18	30.16	0.00
1697	6_28268980	6	28,268,980	167.34	26.71	0.00
1698	6_28293088	6	28,293,088	167.36	26.71	0.00
1699	6_28293184	6	28,293,184	167.53	26.71	0.00
1700	6_28293312	6	28,293,312	167.2	26.71	0.00
1701	6_28378432	6	28,378,432	165.71	28.66	0.00
1702	6_28473212	6	28,473,212	166.65	26.71	0.00
1703	6_28479466	6	28,479,466	166.32	27.64	0.00
1704	6_28527838	6	28,527,838	166.17	28.66	0.00
1705	6_28527993	6	28,527,993	166.88	26.71	0.00
1706	6_28621573	6	28,621,573	165.9	27.25	0.00
1707	6_28672825	6	28,672,825	167.07	25.24	0.00
1708	6_28840911	6	28,840,911	165.95	27.25	0.00
1709	6_29342862	6	29,342,862	170.83	25.85	0.00
1710	6_29399022	6	29,399,022	171.85	22.94	0.00
1711	6_29408973	6	29,408,973	172.76	24.36	0.00

1712	6_29410706	6	29,410,706	171.94	22.94	0.00
1713	6_29433388	6	29,433,388	172.17	22.94	0.00
1714	6_29440931	6	29,440,931	172.17	22.94	0.00
1715	6_29441307	6	29,441,307	172.49	22.94	0.00
1716	6_29662407	6	29,662,407	174.06	22.94	0.00
1717	6_29759349	6	29,759,349	173.94	22.94	0.00
1718	6_30027302	6	30,027,302	176.12	21.62	0.00
1719	6_30027633	6	30,027,633	175.98	21.62	0.00
1720	6_30027680	6	30,027,680	175.31	20.76	0.00
1721	6_30030609	6	30,030,609	176.63	20.76	0.00
1722	6_30032868	6	30,032,868	176.33	21.62	0.00
1723	6_30033031	6	30,033,031	177.18	21.62	0.00
1724	6_30205810	6	30,205,810	178.36	23.57	0.00
1725	6_30338106	6	30,338,106	181.59	23.57	0.00
1726	6_30341511	6	30,341,511	185.58	23.57	0.00
1727	6_30354188	6	30,354,188	181.17	23.52	0.00
1728	6_30354283	6	30,354,283	181.03	23.52	0.00
1729	6_30354332	6	30,354,332	180.77	24.68	0.00
1730	6_30354387	6	30,354,387	181.32	22.38	0.00
1731	6_30542472	6	30,542,472	182.07	21.28	0.00
1732	6_30595640	6	30,595,640	181.98	22.38	0.00
1733	6_30595781	6	30,595,781	182.05	22.38	0.00
1734	6_30647467	6	30,647,467	180.55	24.68	0.00
1735	6_30683303	6	30,683,303	183.53	23.57	0.00
1736	6_30683597	6	30,683,597	183.53	23.57	0.00
1737	6_30855972	6	30,855,972	182.83	21.34	0.00
1738	6_30872059	6	30,872,059	182.99	22.38	0.00
1739	6_30892157	6	30,892,157	182.99	21.34	0.00
1740	7_344231	7	344,231	0.11	1.53	0.46
1741	7_361737	7	361,737	0	2.08	0.35
1742	7_586981	7	586,981	1.93	3.03	0.22
1743	7_597902	7	597,902	1.99	3.03	0.22
1744	7_735605	7	735,605	2.84	2.37	0.31
1745	7_1017210	7	1,017,210	5.26	2.47	0.29
1746	7_1362935	7	1,362,935	6.39	1.74	0.42
1747	7_1372415	7	1,372,415	5.67	2.80	0.25
1748	7_1372520	7	1,372,520	5.67	2.80	0.25
1749	7_1375009	7	1,375,009	5.43	2.24	0.33
1750	7_1473123	7	1,473,123	6.56	2.31	0.31
1751	7_1675663	7	1,675,663	8.49	1.74	0.42
1752	7_1781145	7	1,781,145	8.92	1.70	0.43
1753	7_1789552	7	1,789,552	9.19	1.70	0.43
1754	7_1850656	7	1,850,656	9.93	1.87	0.39
1755	7_1870234	7	1,870,234	10.21	1.35	0.51
1756	7_2355259	7	2,355,259	14.87	5.15	0.08
1757	7_2355322	7	2,355,322	14.49	3.76	0.15
1758	7_2439640	7	2,439,640	14.9	5.10	0.08
1759	7_2520627	7	2,520,627	15.68	5.10	0.08
1760	7_2594557	7	2,594,557	15.84	4.38	0.11

1761	7_2819375	7	2,819,375	16.3	3.10	0.21
1762	7_3020780	7	3,020,780	17.2	3.76	0.15
1763	7_3021079	7	3,021,079	18.44	2.52	0.28
1764	7_3031087	7	3,031,087	22.37	1.24	0.54
1765	7_3062797	7	3,062,797	18.23	3.76	0.15
1766	7_3068771	7	3,068,771	17.88	3.21	0.20
1767	7_3069787	7	3,069,787	18.81	2.15	0.34
1768	7_3081347	7	3,081,347	18.14	3.21	0.20
1769	7_3097380	7	3,097,380	18.35	3.10	0.21
1770	7_3131901	7	3,131,901	19.56	2.58	0.28
1771	7_3132222	7	3,132,222	19.28	2.15	0.34
1772	7_3158938	7	3,158,938	20.81	2.15	0.34
1773	7_3210606	7	3,210,606	21.26	1.53	0.46
1774	7_3214998	7	3,214,998	22.97	1.53	0.46
1775	7_3628958	7	3,628,958	22.9	1.53	0.46
1776	7_3842731	7	3,842,731	22.9	1.35	0.51
1777	7_3842790	7	3,842,790	22.9	1.35	0.51
1778	7_4131473	7	4,131,473	25.2	1.87	0.39
1779	7_4375004	7	4,375,004	27.14	2.71	0.26
1780	7_4494898	7	4,494,898	27.78	2.71	0.26
1781	7_4555913	7	4,555,913	26.89	1.87	0.39
1782	7_4586785	7	4,586,785	28.47	1.74	0.42
1783	7_4587008	7	4,587,008	28.34	1.87	0.39
1784	7_4639007	7	4,639,007	28.68	1.87	0.39
1785	7_4654816	7	4,654,816	28.68	1.87	0.39
1786	7_4719834	7	4,719,834	28.98	0.92	0.63
1787	7_4834320	7	4,834,320	31.6	0.90	0.64
1788	7_4839914	7	4,839,914	30.28	0.19	0.91
1789	7_4840086	7	4,840,086	30.53	0.40	0.82
1790	7_4859285	7	4,859,285	31.33	1.31	0.52
1791	7_4931773	7	4,931,773	32.23	0.90	0.64
1792	7_5247255	7	5,247,255	33.16	1.20	0.55
1793	7_5251903	7	5,251,903	33.88	0.85	0.66
1794	7_5291372	7	5,291,372	34.84	3.21	0.20
1795	7_8237164	7	8,237,164	45.11	0.57	0.75
1796	7_8469691	7	8,469,691	44.49	0.57	0.75
1797	7_8780391	7	8,780,391	45.88	1.07	0.59
1798	7_8848963	7	8,848,963	37.94	27.77	0.00
1799	7_8849155	7	8,849,155	39.21	32.19	0.00
1800	7_8849161	7	8,849,161	39.21	32.19	0.00
1801	7_8849179	7	8,849,179	39.21	32.19	0.00
1802	7_8849234	7	8,849,234	39.23	29.94	0.00
1803	7_9469929	7	9,469,929	44.49	1.31	0.52
1804	7_9817231	7	9,817,231	43.21	1.20	0.55
1805	7_9817355	7	9,817,355	43.97	0.90	0.64
1806	7_9817356	7	9,817,356	43.58	0.90	0.64
1807	7_10312610	7	10,312,610	47.63	0.57	0.75
1808	7_10347932	7	10,347,932	47.78	0.69	0.71
1809	7_10348574	7	10,348,574	47.78	0.57	0.75

1810	7_10366570	7	10,366,570	47.83	0.57	0.75
1811	7_10378201	7	10,378,201	47.78	0.69	0.71
1812	7_11873378	7	11,873,378	49.75	0.36	0.84
1813	7_11873441	7	11,873,441	49.79	0.36	0.84
1814	7_11916371	7	11,916,371	50.45	0.85	0.66
1815	7_11916437	7	11,916,437	50.57	0.85	0.66
1816	7_12894447	7	12,894,447	54.55	0.90	0.64
1817	7_12920699	7	12,920,699	54.45	0.90	0.64
1818	7_13438694	7	13,438,694	56.07	1.07	0.59
1819	7_13438739	7	13,438,739	56.79	0.75	0.69
1820	7_13439651	7	13,439,651	56.58	1.07	0.59
1821	7_13439783	7	13,439,783	55.75	1.57	0.46
1822	7_13439888	7	13,439,888	55.99	1.07	0.59
1823	7_14373496	7	14,373,496	59.43	0.69	0.71
1824	7_14819586	7	14,819,586	59.83	0.51	0.77
1825	7_15206539	7	15,206,539	61.45	0.06	0.97
1826	7_15206679	7	15,206,679	61.34	0.06	0.97
1827	7_15256864	7	15,256,864	61.76	0.08	0.96
1828	7_15274639	7	15,274,639	62.74	0.57	0.75
1829	7_15274642	7	15,274,642	62.71	0.57	0.75
1830	7_15274724	7	15,274,724	62.18	0.08	0.96
1831	7_16111289	7	16,111,289	67.45	0.13	0.94
1832	7_16247361	7	16,247,361	66.73	0.36	0.84
1833	7_16256469	7	16,256,469	65.37	0.06	0.97
1834	7_16256625	7	16,256,625	65.38	0.06	0.97
1835	7_16261094	7	16,261,094	65.81	0.13	0.94
1836	7_16261264	7	16,261,264	66.11	0.36	0.84
1837	7_16272947	7	16,272,947	67.04	0.36	0.84
1838	7_16272962	7	16,272,962	66.89	0.36	0.84
1839	7_16273177	7	16,273,177	65.88	0.29	0.87
1840	7_16273796	7	16,273,796	66.23	0.36	0.84
1841	7_16580392	7	16,580,392	70.4	1.41	0.50
1842	7_16584776	7	16,584,776	69.43	0.62	0.73
1843	7_16584849	7	16,584,849	69.75	0.86	0.65
1844	7_16619540	7	16,619,540	69.84	1.24	0.54
1845	7_17208684	7	17,208,684	74.83	2.87	0.24
1846	7_17213844	7	17,213,844	74.83	2.87	0.24
1847	7_17330487	7	17,330,487	75.34	3.59	0.17
1848	7_17544198	7	17,544,198	76.08	2.87	0.24
1849	7_17554680	7	17,554,680	76.32	2.87	0.24
1850	7_17744706	7	17,744,706	77.9	1.70	0.43
1851	7_17816346	7	17,816,346	78.78	0.97	0.62
1852	7_17816839	7	17,816,839	78.78	0.97	0.62
1853	7_17888987	7	17,888,987	79.83	0.97	0.62
1854	7_17931016	7	17,931,016	81.31	0.23	0.89
1855	7_17931064	7	17,931,064	81.28	0.23	0.89
1856	7_18121651	7	18,121,651	81.86	0.97	0.62
1857	7_18514901	7	18,514,901	83.48	0.97	0.62
1858	7_18668087	7	18,668,087	84.54	1.41	0.50

1859	7_18800910	7	18,800,910	86.48	1.20	0.55
1860	7_18981521	7	18,981,521	86.1	2.58	0.28
1861	7_18981798	7	18,981,798	86.15	2.58	0.28
1862	7_18989224	7	18,989,224	85.88	1.53	0.46
1863	7_18993172	7	18,993,172	85.61	0.69	0.71
1864	7_19005530	7	19,005,530	86.45	1.07	0.59
1865	7_19023724	7	19,023,724	85.43	0.69	0.71
1866	7_19560129	7	19,560,129	90.55	0.08	0.96
1867	7_19560192	7	19,560,192	90.36	0.08	0.96
1868	7_19560215	7	19,560,215	90.04	0.36	0.84
1869	7_19561901	7	19,561,901	92.57	0.23	0.89
1870	7_19577371	7	19,577,371	92.67	0.13	0.94
1871	7_19584382	7	19,584,382	92.11	0.97	0.62
1872	7_19584480	7	19,584,480	92.03	0.97	0.62
1873	7_19620833	7	19,620,833	91.48	0.13	0.94
1874	7_19622719	7	19,622,719	91.31	0.12	0.94
1875	7_19735649	7	19,735,649	93.36	0.06	0.97
1876	7_19749820	7	19,749,820	91.67	0.41	0.81
1877	7_19757897	7	19,757,897	94.06	0.13	0.94
1878	7_19758653	7	19,758,653	94.16	0.13	0.94
1879	7_19816815	7	19,816,815	93.74	0.08	0.96
1880	7_20309612	7	20,309,612	95.54	0.29	0.87
1881	7_20310022	7	20,310,022	95.45	0.29	0.87
1882	7_20416776	7	20,416,776	96.39	0.36	0.84
1883	7_20727167	7	20,727,167	103.85	2.75	0.25
1884	7_20727233	7	20,727,233	103.85	3.21	0.20
1885	7_20727258	7	20,727,258	103.94	2.75	0.25
1886	7_20730041	7	20,730,041	103.18	1.14	0.57
1887	7_20799496	7	20,799,496	100.37	0.51	0.77
1888	7_20922872	7	20,922,872	99.88	0.75	0.69
1889	7_20925778	7	20,925,778	102.61	0.23	0.89
1890	7_20926058	7	20,926,058	100.04	0.51	0.77
1891	7_20953865	7	20,953,865	100.19	0.36	0.84
1892	7_20953907	7	20,953,907	102.09	0.06	0.97
1893	7_20958480	7	20,958,480	102.39	0.06	0.97
1894	7_21032365	7	21,032,365	100.25	0.36	0.84
1895	7_21099515	7	21,099,515	101.15	0.29	0.87
1896	7_21118535	7	21,118,535	100.68	0.36	0.84
1897	7_21119014	7	21,119,014	101.76	0.12	0.94
1898	7_21124745	7	21,124,745	101.38	0.13	0.94
1899	7_21124787	7	21,124,787	100.86	0.29	0.87
1900	7_21238524	7	21,238,524	104.57	0.69	0.71
1901	7_21266167	7	21,266,167	101.26	0.29	0.87
1902	7_21285487	7	21,285,487	107.36	0.19	0.91
1903	7_21290598	7	21,290,598	105.8	0.51	0.77
1904	7_21293507	7	21,293,507	105.21	0.51	0.77
1905	7_21312927	7	21,312,927	105.71	0.36	0.84
1906	7_21315349	7	21,315,349	107.5	0.02	0.99
1907	7_21327908	7	21,327,908	107.46	0.06	0.97

1908	7_21331377	7	21,331,377	106.85	0.23	0.89
1909	7_21331527	7	21,331,527	106.2	0.41	0.81
1910	7_21335040	7	21,335,040	107.25	0.23	0.89
1911	7_21345307	7	21,345,307	106.75	0.36	0.84
1912	7_21414466	7	21,414,466	106.57	0.36	0.84
1913	7_22274909	7	22,274,909	110.33	0.57	0.75
1914	7_22275017	7	22,275,017	112.2	0.85	0.66
1915	7_22279232	7	22,279,232	111.69	0.57	0.75
1916	7_22279352	7	22,279,352	111.37	0.57	0.75
1917	7_22286698	7	22,286,698	111.02	0.57	0.75
1918	7_22286991	7	22,286,991	111.2	0.69	0.71
1919	7_22288953	7	22,288,953	110.71	0.30	0.86
1920	7_22354269	7	22,354,269	111.82	0.30	0.86

Table S7. Mapped SNP loci on WI7799 RIL linkage map using DArTag SNP genotyping.

#	Marker loci	Position (cM)	Chr	Physical position (Gy14v2.0)	Physical position (9930v3.0)	chi-square value	p-value
1	Chr1_00042990	0	Chr1	42,990	62,710	0.39	0.53
2	Chr1_00675681	3.4	Chr1	675,681	703,345	0.00	1.00
3	Chr1_00793837	5.97	Chr1	793,837	821,561	0.13	0.72
4	Chr1_01783917	9.13	Chr1	1,783,917	1,814,448	0.03	0.86
5	Chr1_01770411	9.13	Chr1	1,770,411	1,800,963	0.00	1.00
6	Chr1_02177181	12.79	Chr1	2,177,181	2,204,761	0.03	0.86
7	Chr1_02726144	15.43	Chr1	2,726,144	no hit	0.07	0.79
8	Chr1_03949642	20.65	Chr1	3,949,642	3,981,185	0.20	0.65
9	Chr1_03950288	20.67	Chr1	3,950,288	3,981,831	0.13	0.72
10	Chr1_04943736	26.04	Chr1	4,943,736	4,972,196	0.20	0.65
11	Chr1_05364698	28.69	Chr1	5,364,698	5,394,075	0.82	0.37
12	Chr1_06902189	34.84	Chr1	6,902,189	6,908,503	1.37	0.24
13	Chr1_06640945	34.84	Chr1	6,640,945	6,650,871	2.03	0.15
14	Chr1_07310057	37.27	Chr1	7,310,057	7,315,288	0.82	0.37
15	Chr1_07514663	37.51	Chr1	7,514,663	7,517,400	0.41	0.52
16	Chr1_07825347	38.2	Chr1	7,825,347	7,826,100	1.18	0.28
17	Chr1_08051069	40.41	Chr1	8,051,069	8,051,977	2.10	0.15
18	Chr1_08284304	41.7	Chr1	8,284,304	8,280,718	2.98	0.08
19	Chr1_08873058	42.47	Chr1	8,873,058	8,867,313	4.37	0.04
20	Chr1_09807184	45.85	Chr1	9,807,184	9,825,689	1.37	0.24
21	Chr1_10923293	48.32	Chr1	10,923,293	10,952,961	1.83	0.18
22	Chr1_11579522	50.37	Chr1	11,579,522	11,774,072	2.93	0.09
23	Chr1_14866545	53.8	Chr1	14,866,545	14,770,584	2.61	0.11
24	Chr1_15029650	54.42	Chr1	15,029,650	14,927,835	1.80	0.18
25	Chr1_15684369	54.81	Chr1	15,684,369	15,512,052	1.56	0.21
26	Chr1_16756540	63.24	Chr1	16,756,540	16,559,383	0.00	1.00
27	Chr1_19471374	76.68	Chr1	19,471,374	19,343,903	0.07	0.79
28	Chr1_19887404	78.1	Chr1	19,887,404	19,759,990	0.00	1.00
29	Chr1_20464540	80.01	Chr1	20,464,540	no hit	0.07	0.79
30	Chr1_21853430	83.47	Chr1	21,853,430	no hit	0.29	0.59
31	Chr1_23300122	90.89	Chr1	23,300,122	23,256,450	0.01	0.93
32	Chr1_23570878	91.29	Chr1	23,570,878	23,526,773	0.29	0.59
33	Chr1_25159025	101.06	Chr1	25,159,025	25,065,564	1.37	0.24
34	Chr1_27572481	113.27	Chr1	27,572,481	27,489,455	2.43	0.12
35	Chr1_27759219	113.85	Chr1	27,759,219	27,674,046	3.03	0.08
36	Chr1_27840491	114.06	Chr1	27,840,491	27,760,659	2.75	0.10
37	Chr1_28772037	116.58	Chr1	28,772,037	28,672,775	4.52	0.03
38	Chr1_31096954	126.24	Chr1	31,096,954	30,967,761	3.19	0.07
39	Chr1_31187561	126.42	Chr1	31,187,561	no hit	2.84	0.09
40	Chr1_32041177	130.16	Chr1	32,041,177	31,571,850	1.66	0.20
41	Chr1_31949505	130.16	Chr1	31,949,505	31,485,767	5.34	0.02
42	Chr1_32911888	132.03	Chr1	32,911,888	no hit	6.23	0.01
43	Chr1_33005010	133.07	Chr1	33,005,010	32,504,553	6.34	0.01
44	Chr2_02554207	0	Chr2	2,554,207	2,710,577	7.63	0.01
45	Chr2_02677292	1.23	Chr2	2,677,292	2,834,735	9.31	0.00
46	Chr2_03195277	2.84	Chr2	3,195,277	3,396,451	6.23	0.01
47	Chr2_04173417	8.65	Chr2	4,173,417	4,371,493	4.03	0.04
48	Chr2_04641963	10.88	Chr2	4,641,963	4,824,086	5.25	0.02
49	Chr2_04446282	11.19	Chr2	4,446,282	4,650,059	6.13	0.01
50	Chr2_05620990	17.07	Chr2	5,620,990	5,789,934	7.38	0.01
51	Chr2_05732918	17.36	Chr2	5,732,918	5,900,885	5.83	0.02
52	Chr2_05821438	17.78	Chr2	5,821,438	5,994,692	6.95	0.01
53	Chr2_06125305	20.62	Chr2	6,125,305	6,297,341	7.38	0.01

54	Chr2_07047706	24.19	Chr2	7,047,706	7,209,657	7.38	0.01
55	Chr2_07030144	24.29	Chr2	7,030,144	7,192,095	7.81	0.01
56	Chr2_07840608	27.81	Chr2	7,840,608	7,998,780	6.02	0.01
57	Chr2_09936043	37.02	Chr2	9,936,043	10,099,499	1.77	0.18
58	Chr2_09941435	37.02	Chr2	9,941,435	10,104,891	2.53	0.11
59	Chr2_11119537	40.28	Chr2	11,119,537	11,255,780	2.31	0.13
60	Chr2_11713449	42.41	Chr2	11,713,449	11,844,713	0.51	0.48
61	Chr2_12231618	45.36	Chr2	12,231,618	12,365,758	0.78	0.38
62	Chr2_12403934	47.72	Chr2	12,403,934	12,538,952	1.33	0.25
63	Chr2_12790844	50.2	Chr2	12,790,844	12,941,066	0.64	0.42
64	Chr2_13120731	50.5	Chr2	13,120,731	13,272,902	0.51	0.48
65	Chr2_14689901	52.78	Chr2	14,689,901	14,337,244	0.29	0.59
66	Chr2_15528234	56.39	Chr2	15,528,234	15,152,539	0.13	0.72
67	Chr2_27087852	60.73	Chr2	27,087,852	16,893,264	0.97	0.33
68	Chr2_27685487	65.71	Chr2	27,685,487	17,485,610	0.51	0.48
69	Chr2_27876290	72.28	Chr2	27,876,290	17,671,558	1.35	0.24
70	Chr2_30934348	92.17	Chr2	30,934,348	20,767,632	1.05	0.31
71	Chr2_31663466	98.03	Chr2	31,663,466	21,524,459	0.42	0.52
72	Chr2_31882163	100.09	Chr2	31,882,163	21,738,761	0.08	0.78
73	Chr2_31821504	100.38	Chr2	31,821,504	21,678,937	0.03	0.86
74	Chr2_32891507	110.97	Chr2	32,891,507	22,728,323	0.83	0.36
75	Chr2_33235576	113.38	Chr2	33,235,576	23,055,046	1.18	0.28
76	Chr2_33727347	117.67	Chr2	33,727,347	23,540,171	3.51	0.06
77	Chr2_34112954	118.64	Chr2	34,112,954	23,910,448	0.30	0.58
78	Chr3_01474731	0	Chr3	1,474,731	1,631,027	0.30	0.59
79	Chr3_00161385	0.39	Chr3	161,385	292,831	0.40	0.53
80	Chr3_00040563	1.01	Chr3	40,563	no hit	0.20	0.65
81	Chr3_02628735	4.18	Chr3	2,628,735	2,779,496	0.13	0.72
82	Chr3_03717768	6.9	Chr3	3,717,768	3,859,366	0.40	0.53
83	Chr3_04160081	7.41	Chr3	4,160,081	4,300,793	0.30	0.58
84	Chr3_04110929	7.41	Chr3	4,110,929	4,251,614	3.64	0.06
85	Chr3_04164621	8.55	Chr3	4,164,621	4,305,339	0.21	0.65
86	Chr3_04931932	10.54	Chr3	4,931,932	5,070,112	0.07	0.79
87	Chr3_05259343	12.24	Chr3	5,259,343	5,415,740	0.03	0.86
88	Chr3_05930147	12.63	Chr3	5,930,147	6,082,956	0.03	0.86
89	Chr3_05266043	12.63	Chr3	5,266,043	5,422,440	0.01	0.93
90	Chr3_05613707	12.97	Chr3	5,613,707	5,759,631	0.03	0.86
91	Chr3_08553561	29.69	Chr3	8,553,561	8,701,817	0.21	0.65
92	Chr3_09052339	33.27	Chr3	9,052,339	9,200,650	0.13	0.72
93	Chr3_10234865	37.36	Chr3	10,234,865	10,394,214	0.40	0.53
94	Chr3_10546352	39.32	Chr3	10,546,352	10,703,689	0.52	0.47
95	Chr3_11610211	47.01	Chr3	11,610,211	11,768,986	0.81	0.37
96	Chr3_12044989	51.03	Chr3	12,044,989	12,209,494	0.39	0.53
97	Chr3_13144064	52.56	Chr3	13,144,064	12,914,770	0.97	0.33
98	Chr3_13965880	56.22	Chr3	13,965,880	13,737,934	0.39	0.53
99	Chr3_14111970	58.13	Chr3	14,111,970	13,879,294	0.07	0.79
100	Chr3_15061942	63.53	Chr3	15,061,942	14,831,952	0.00	1.00
101	Chr3_16491949	70.64	Chr3	16,491,949	16,270,109	0.82	0.37
102	Chr3_17988391	72.76	Chr3	17,988,391	17,801,004	1.20	0.27
103	Chr3_18002819	72.81	Chr3	18,002,819	17,817,994	0.85	0.36
104	Chr3_18024951	72.81	Chr3	18,024,951	17,840,394	0.40	0.52
105	Chr3_18733959	74.85	Chr3	18,733,959	18,599,804	0.82	0.37
106	Chr3_19700268	76.98	Chr3	19,700,268	19,557,195	0.30	0.59
107	Chr3_21259570	79.49	Chr3	21,259,570	21,103,235	0.07	0.79
108	Chr3_24122264	80.46	Chr3	24,122,264	23,570,938	0.13	0.72
109	Chr3_22369420	80.46	Chr3	22,369,420	21,958,497	0.07	0.79
110	Chr3_23265420	80.48	Chr3	23,265,420	22,721,889	0.13	0.72

111	Chr3_22452666	81.16	Chr3	22,452,666	22,041,702	0.70	0.40
112	Chr3_22386501	82.36	Chr3	22,386,501	21,975,544	2.25	0.13
113	Chr3_25265646	84.13	Chr3	25,265,646	24,654,561	0.83	0.36
114	Chr3_27887794	90.34	Chr3	27,887,794	27,059,020	0.21	0.65
115	Chr3_28272707	91.22	Chr3	28,272,707	27,438,811	0.07	0.79
116	Chr3_28312700	91.59	Chr3	28,312,700	27,478,232	0.13	0.72
117	Chr3_29651777	96.92	Chr3	29,651,777	28,807,221	0.13	0.72
118	Chr3_30264793	100.18	Chr3	30,264,793	29,418,348	0.13	0.72
119	Chr3_30838807	103.06	Chr3	30,838,807	29,992,446	0.00	1.00
120	Chr3_31138697	103.78	Chr3	31,138,697	30,292,030	0.03	0.86
121	Chr3_31743279	105.38	Chr3	31,743,279	30,868,749	0.13	0.72
122	Chr3_32701704	109.2	Chr3	32,701,704	31,853,185	0.00	1.00
123	Chr3_32939110	111.33	Chr3	32,939,110	32,094,810	0.00	1.00
124	Chr3_33022610	113.38	Chr3	33,022,610	32,178,481	0.01	0.93
125	Chr3_34197423	124.5	Chr3	34,197,423	33,358,789	0.07	0.79
126	Chr3_34769709	125.77	Chr3	34,769,709	33,930,903	0.28	0.60
127	Chr3_35068623	127.13	Chr3	35,068,623	34,224,464	0.19	0.66
128	Chr3_36275060	134.23	Chr3	36,275,060	35,426,850	2.00	0.16
129	Chr3_36254933	135.98	Chr3	36,254,933	35,406,509	8.98	0.00
130	Chr3_36485387	135.98	Chr3	36,485,387	35,638,234	1.77	0.18
131	Chr3_36572968	136.88	Chr3	36,572,968	35,723,906	1.14	0.29
132	Chr3_39494731	151.23	Chr3	39,494,731	38,652,277	0.20	0.65
133	Chr3_39502285	151.48	Chr3	39,502,285	38,659,796	0.30	0.59
134	Chr3_40281546	154.98	Chr3	40,281,546	39,443,369	0.01	0.93
135	Chr3_41139070	157.73	Chr3	41,139,070	no hit	0.01	0.93
136	Chr4_00126803	0	Chr4	126,803	121,488	1.80	0.18
137	Chr4_00733327	5.21	Chr4	733,327	740,918	2.06	0.15
138	Chr4_01828560	15.43	Chr4	1,828,560	1,832,529	2.57	0.11
139	Chr4_02765435	23.24	Chr4	2,765,435	2,762,508	0.97	0.33
140	Chr4_02793245	24.11	Chr4	2,793,245	2,790,441	1.56	0.21
141	Chr4_03194713	26.34	Chr4	3,194,713	no hit	1.33	0.25
142	Chr4_03201550	26.98	Chr4	3,201,550	no hit	2.93	0.09
143	Chr4_03414567	27.65	Chr4	3,414,567	3,295,707	2.00	0.16
144	Chr4_03871802	30.15	Chr4	3,871,802	3,746,259	1.56	0.21
145	Chr4_04239965	32.45	Chr4	4,239,965	4,117,975	0.28	0.60
146	Chr4_05769082	42.01	Chr4	5,769,082	5,639,670	0.38	0.54
147	Chr4_05972211	42.92	Chr4	5,972,211	5,842,482	0.19	0.66
148	Chr4_06305147	43.74	Chr4	6,305,147	6,184,334	0.19	0.66
149	Chr4_07713153	49.24	Chr4	7,713,153	7,591,076	0.50	0.48
150	Chr4_08273018	55.89	Chr4	8,273,018	8,154,447	0.39	0.53
151	Chr4_08965293	59.69	Chr4	8,965,293	8,729,548	0.78	0.38
152	Chr4_10261994	62.81	Chr4	10,261,994	9,918,054	0.78	0.38
153	Chr4_10550510	63.13	Chr4	10,550,510	10,199,668	0.95	0.33
154	Chr4_10553779	63.43	Chr4	10,553,779	10,202,939	0.50	0.48
155	Chr4_18469190	69.45	Chr4	18,469,190	14,108,878	0.20	0.65
156	Chr4_18609621	69.76	Chr4	18,609,621	14,249,604	0.13	0.72
157	Chr4_18753052	70.8	Chr4	18,753,052	14,399,731	0.03	0.86
158	Chr4_18803475	71.87	Chr4	18,803,475	14,450,333	0.13	0.72
159	Chr4_19568497	77.7	Chr4	19,568,497	15,211,958	0.64	0.42
160	Chr4_19663931	79.54	Chr4	19,663,931	15,307,350	1.83	0.18
161	Chr4_19679337	80.1	Chr4	19,679,337	15,322,700	1.14	0.29
162	Chr4_19925410	82.56	Chr4	19,925,410	15,560,116	0.07	0.79
163	Chr4_19925567	82.56	Chr4	19,925,567	15,560,273	5.83	0.02
164	Chr4_19941373	83.73	Chr4	19,941,373	15,576,299	0.03	0.86
165	Chr4_20591943	88.05	Chr4	20,591,943	16,284,396	0.07	0.79
166	Chr4_20623063	88.91	Chr4	20,623,063	no hit	0.30	0.59
167	Chr4_20708624	89.62	Chr4	20,708,624	16,397,147	0.07	0.79

168	Chr4_20830953	90.24	Chr4	20,830,953	16,520,194	0.20	0.65
169	Chr4_21923834	96.81	Chr4	21,923,834	17,586,005	0.01	0.93
170	Chr4_22544988	98.81	Chr4	22,544,988	18,192,798	0.03	0.86
171	Chr4_22901424	98.81	Chr4	22,901,424	18,551,963	1.47	0.23
172	Chr4_23048483	99.6	Chr4	23,048,483	18,694,247	0.07	0.79
173	Chr4_22955984	99.7	Chr4	22,955,984	18,606,541	0.03	0.86
174	Chr4_23326921	100.71	Chr4	23,326,921	18,974,358	0.20	0.65
175	Chr4_23679590	102.16	Chr4	23,679,590	no hit	0.08	0.78
176	Chr4_23725131	103.49	Chr4	23,725,131	19,372,689	0.03	0.86
177	Chr4_23893336	104.39	Chr4	23,893,336	19,541,072	0.21	0.65
178	Chr4_24083777	105.32	Chr4	24,083,777	19,737,613	0.03	0.86
179	Chr4_24434698	107.02	Chr4	24,434,698	20,016,081	0.07	0.79
180	Chr4_25437481	111.67	Chr4	25,437,481	20,926,389	0.01	0.93
181	Chr4_25872198	112.53	Chr4	25,872,198	21,368,075	0.07	0.79
182	Chr4_25973875	112.7	Chr4	25,973,875	21,470,106	0.03	0.86
183	Chr4_26097531	113.61	Chr4	26,097,531	21,573,996	0.21	0.65
184	Chr4_26438043	114.52	Chr4	26,438,043	21,925,296	0.30	0.59
185	Chr4_26561162	115.42	Chr4	26,561,162	22,037,092	0.21	0.65
186	Chr4_26791467	116.71	Chr4	26,791,467	22,265,880	0.01	0.93
187	Chr4_27018091	120.36	Chr4	27,018,091	22,492,472	0.07	0.79
188	Chr4_27562362	123.12	Chr4	27,562,362	23,028,001	0.01	0.93
189	Chr4_27573046	123.83	Chr4	27,573,046	23,038,993	0.01	0.93
190	Chr4_27852627	126.13	Chr4	27,852,627	23,317,980	0.69	0.41
191	Chr4_28929526	136.11	Chr4	28,929,526	24,397,821	0.08	0.78
192	Chr4_29005157	136.97	Chr4	29,005,157	24,473,476	0.03	0.85
193	Chr4_29444662	139.53	Chr4	29,444,662	24,891,253	0.00	1.00
194	Chr4_30408369	151.2	Chr4	30,408,369	25,849,536	0.01	0.93
195	Chr5_00319487	0	Chr5	319,487	325,517	0.85	0.36
196	Chr5_00258634	0.42	Chr5	258,634	264,610	1.02	0.31
197	Chr5_00417959	2.56	Chr5	417,959	424,395	0.85	0.36
198	Chr5_00842846	6.89	Chr5	842,846	849,390	1.61	0.20
199	Chr5_01265330	7.6	Chr5	1,265,330	1,271,255	1.37	0.24
200	Chr5_01132333	7.72	Chr5	1,132,333	1,142,027	1.61	0.20
201	Chr5_02822582	16.45	Chr5	2,822,582	2,831,929	0.29	0.59
202	Chr5_03031671	17.94	Chr5	3,031,671	3,036,439	0.03	0.86
203	Chr5_03525216	21.92	Chr5	3,525,216	3,525,840	1.00	0.32
204	Chr5_03774121	23.63	Chr5	3,774,121	3,763,914	0.13	0.72
205	Chr5_05182471	36.81	Chr5	5,182,471	5,230,193	0.01	0.93
206	Chr5_05416694	38.16	Chr5	5,416,694	5,467,191	0.13	0.72
207	Chr5_06777958	45.4	Chr5	6,777,958	6,903,854	0.52	0.47
208	Chr5_06757030	45.4	Chr5	6,757,030	6,887,777	0.30	0.58
209	Chr5_07280009	49.7	Chr5	7,280,009	7,359,200	0.03	0.86
210	Chr5_11545575	56.32	Chr5	11,545,575	11,523,346	0.68	0.41
211	Chr5_11776846	56.74	Chr5	11,776,846	11,755,072	0.42	0.52
212	Chr5_12302410	58.41	Chr5	12,302,410	12,327,989	0.08	0.78
213	Chr5_12913752	58.74	Chr5	12,913,752	12,938,773	0.14	0.71
214	Chr5_12894905	58.97	Chr5	12,894,905	12,919,926	0.03	0.85
215	Chr5_17577242	63.59	Chr5	17,577,242	15,435,643	0.13	0.72
216	Chr5_17981300	63.8	Chr5	17,981,300	15,827,532	0.13	0.72
217	Chr5_19676586	65.09	Chr5	19,676,586	17,769,477	0.08	0.78
218	Chr5_19801449	65.59	Chr5	19,801,449	17,894,389	0.13	0.72
219	Chr5_20162839	66.78	Chr5	20,162,839	18,251,176	0.08	0.78
220	Chr5_21690344	71.43	Chr5	21,690,344	19,773,641	0.07	0.79
221	Chr5_21698121	71.46	Chr5	21,698,121	19,781,427	0.13	0.72
222	Chr5_21431970	71.65	Chr5	21,431,970	19,520,759	0.03	0.86
223	Chr5_22106624	75.37	Chr5	22,106,624	20,187,292	0.20	0.65
224	Chr5_22356573	76.22	Chr5	22,356,573	20,437,489	0.40	0.53

225	Chr5_22354964	76.22	Chr5	22,354,964	20,435,880	0.54	0.46
226	Chr5_22359236	76.93	Chr5	22,359,236	20,440,152	0.67	0.41
227	Chr5_22358109	76.98	Chr5	22,358,109	20,439,025	0.52	0.47
228	Chr5_22830695	79.62	Chr5	22,830,695	20,883,856	0.98	0.32
229	Chr5_23003037	82.04	Chr5	23,003,037	21,060,213	0.20	0.65
230	Chr5_23004116	82.33	Chr5	23,004,116	21,061,292	0.01	0.93
231	Chr5_23173506	84.05	Chr5	23,173,506	21,228,878	0.30	0.59
232	Chr5_23291547	84.9	Chr5	23,291,547	21,347,037	0.30	0.59
233	Chr5_23731205	86.84	Chr5	23,731,205	21,786,914	0.21	0.65
234	Chr5_23916713	87.3	Chr5	23,916,713	21,972,639	0.03	0.86
235	Chr5_24031132	88.23	Chr5	24,031,132	22,087,080	0.00	1.00
236	Chr5_24123195	88.65	Chr5	24,123,195	22,185,103	0.01	0.93
237	Chr5_24197761	89.53	Chr5	24,197,761	22,259,813	0.00	1.00
238	Chr5_24572298	90.72	Chr5	24,572,298	22,638,163	0.03	0.86
239	Chr5_25004725	90.99	Chr5	25,004,725	23,064,539	0.07	0.79
240	Chr5_26704286	101.89	Chr5	26,704,286	24,759,262	0.03	0.85
241	Chr5_27560453	108.92	Chr5	27,560,453	25,606,087	0.03	0.85
242	Chr5_27717414	110.36	Chr5	27,717,414	25,758,554	0.03	0.85
243	Chr5_27893600	111.29	Chr5	27,893,600	25,936,285	0.00	1.00
244	Chr5_28523108	117.1	Chr5	28,523,108	26,563,189	0.01	0.93
245	Chr5_28824639	117.53	Chr5	28,824,639	26,864,806	0.00	1.00
246	Chr5_29056893	118.88	Chr5	29,056,893	27,097,206	0.08	0.78
247	Chr5_29065322	119.47	Chr5	29,065,322	no hit	0.22	0.64
248	Chr5_29127185	120.35	Chr5	29,127,185	no hit	0.14	0.71
249	Chr5_30556580	124.68	Chr5	30,556,580	28,735,158	0.13	0.72
250	Chr5_30317751	124.68	Chr5	30,317,751	28,494,928	0.13	0.72
251	Chr5_30463866	124.99	Chr5	30,463,866	28,641,075	0.21	0.65
252	Chr5_30822907	125.78	Chr5	30,822,907	29,001,488	0.40	0.52
253	Chr5_31014466	126.56	Chr5	31,014,466	29,191,779	0.21	0.65
254	Chr5_31157691	127.39	Chr5	31,157,691	29,335,063	0.07	0.79
255	Chr5_31436010	128.22	Chr5	31,436,010	29,613,496	0.00	1.00
256	Chr5_31865726	130.12	Chr5	31,865,726	30,037,992	0.03	0.86
257	Chr5_31962678	131.35	Chr5	31,962,678	30,134,952	0.03	0.86
258	Chr5_32702308	132.05	Chr5	32,702,308	30,876,481	0.01	0.93
259	Chr5_33509445	133.1	Chr5	33,509,445	31,681,895	0.14	0.71
260	Chr5_33083667	133.1	Chr5	33,083,667	31,257,896	0.03	0.86
261	Chr6_00805512	0	Chr6	805,512	794,497	4.23	0.04
262	Chr6_00876697	0.5	Chr6	876,697	865,631	3.90	0.05
263	Chr6_01687197	2.87	Chr6	1,687,197	1,668,462	2.98	0.08
264	Chr6_02240763	4.37	Chr6	2,240,763	2,225,302	2.98	0.08
265	Chr6_04270109	16.79	Chr6	4,270,109	4,255,625	0.01	0.93
266	Chr6_04966302	22.08	Chr6	4,966,302	4,955,743	0.01	0.93
267	Chr6_05053945	22.08	Chr6	5,053,945	5,043,365	0.20	0.65
268	Chr6_05235714	24	Chr6	5,235,714	5,224,564	0.03	0.86
269	Chr6_05587054	24.97	Chr6	5,587,054	5,569,377	0.03	0.86
270	Chr6_06530585	34.06	Chr6	6,530,585	6,507,586	0.40	0.53
271	Chr6_07367165	35.79	Chr6	7,367,165	7,349,708	1.40	0.24
272	Chr6_08938543	46.95	Chr6	8,938,543	8,926,514	0.82	0.37
273	Chr6_09076564	47.68	Chr6	9,076,564	9,064,943	0.67	0.41
274	Chr6_09583186	49.46	Chr6	9,583,186	9,563,241	0.52	0.47
275	Chr6_09429756	49.61	Chr6	9,429,756	9,413,299	0.40	0.53
276	Chr6_10016356	52.38	Chr6	10,016,356	9,997,594	0.01	0.93
277	Chr6_10299492	53.46	Chr6	10,299,492	10,274,650	0.07	0.79
278	Chr6_10535611	54.8	Chr6	10,535,611	10,511,520	0.01	0.93
279	Chr6_11071398	54.94	Chr6	11,071,398	11,058,959	0.03	0.86
280	Chr6_11864242	56.24	Chr6	11,864,242	11,849,261	0.03	0.86
281	Chr6_12149834	57.64	Chr6	12,149,834	12,105,026	0.00	1.00

282	Chr6_12106443	57.64	Chr6	12,106,443	12,061,511	0.07	0.79
283	Chr6_12489557	60.25	Chr6	12,489,557	no hit	0.07	0.79
284	Chr6_17910694	64.54	Chr6	17,910,694	no hit	0.20	0.65
285	Chr6_16421910	64.69	Chr6	16,421,910	15,869,056	0.40	0.53
286	Chr6_22034829	83.28	Chr6	22,034,829	20,957,428	5.00	0.03
287	Chr6_22507693	88.61	Chr6	22,507,693	21,419,544	10.12	0.00
288	Chr6_22813475	90.63	Chr6	22,813,475	21,726,330	14.23	0.00
289	Chr6_22857985	90.77	Chr6	22,857,985	21,770,888	13.67	0.00
290	Chr6_23035213	91.36	Chr6	23,035,213	21,947,922	15.61	0.00
291	Chr6_23148340	92.92	Chr6	23,148,340	22,058,543	15.28	0.00
292	Chr6_24166346	96.71	Chr6	24,166,346	23,078,385	15.87	0.00
293	Chr6_24367015	98.43	Chr6	24,367,015	23,279,048	18.89	0.00
294	Chr6_24641481	99.86	Chr6	24,641,481	no hit	22.53	0.00
295	Chr6_25065135	101.89	Chr6	25,065,135	23,869,621	29.25	0.00
296	Chr6_27071188	108.94	Chr6	27,071,188	25,829,271	41.66	0.00
297	Chr6_27580023	111.83	Chr6	27,580,023	26,273,766	54.24	0.00
298	Chr6_27730129	112.46	Chr6	27,730,129	26,423,879	56.98	0.00
299	Chr6_27586317	112.46	Chr6	27,586,317	26,280,041	56.08	0.00
300	Chr6_27771159	113.06	Chr6	27,771,159	26,464,938	58.88	0.00
301	Chr6_28142607	117.11	Chr6	28,142,607	26,837,386	59.80	0.00
302	Chr6_28805203	119.69	Chr6	28,805,203	27,500,392	55.13	0.00
303	Chr6_31102403	128.8	Chr6	31,102,403	29,808,101	43.20	0.00
304	Chr6_32143146	135.88	Chr6	32,143,146	30,850,320	34.13	0.00
305	Chr7_00302420	0	Chr7	302,420	361,940	0.14	0.71
306	Chr7_00819712	4.5	Chr7	819,712	876,953	1.92	0.17
307	Chr7_01271440	8.76	Chr7	1,271,440	1,300,411	2.13	0.14
308	Chr7_01835202	13.95	Chr7	1,835,202	1,870,234	0.31	0.58
309	Chr7_02321968	20.82	Chr7	2,321,968	2,362,263	0.01	0.93
310	Chr7_02975142	26.91	Chr7	2,975,142	3,020,781	0.03	0.86
311	Chr7_02973539	26.91	Chr7	2,973,539	3,019,179	0.14	0.71
312	Chr7_02984116	27.52	Chr7	2,984,116	3,029,741	0.07	0.79
313	Chr7_03145670	29.75	Chr7	3,145,670	3,191,128	0.01	0.93
314	Chr7_03672899	31.62	Chr7	3,672,899	3,836,198	0.03	0.86
315	Chr7_04681221	38.32	Chr7	4,681,221	4,836,123	0.01	0.93
316	Chr7_07498883	45.13	Chr7	7,498,883	7,781,806	0.66	0.42
317	Chr7_08953032	45.94	Chr7	8,953,032	8,242,508	0.40	0.53
318	Chr7_09087270	46.36	Chr7	9,087,270	8,360,347	0.29	0.59
319	Chr7_09270474	46.58	Chr7	9,270,474	8,540,084	0.20	0.65
320	Chr7_09490319	47.46	Chr7	9,490,319	8,762,280	0.07	0.79
321	Chr7_10961840	47.82	Chr7	10,961,840	9,818,405	0.03	0.86
322	Chr7_11440251	49.06	Chr7	11,440,251	10,299,961	0.00	1.00
323	Chr7_12810348	53.57	Chr7	12,810,348	11,682,399	0.01	0.93
324	Chr7_12811064	54.3	Chr7	12,811,064	11,683,115	0.07	0.79
325	Chr7_14012943	57.04	Chr7	14,012,943	12,890,052	0.07	0.79
326	Chr7_14568238	58.15	Chr7	14,568,238	13,434,632	0.66	0.42
327	Chr7_17433363	70.97	Chr7	17,433,363	16,249,736	0.03	0.86
328	Chr7_17673497	73.03	Chr7	17,673,497	16,489,023	0.01	0.93
329	Chr7_18745513	79.85	Chr7	18,745,513	17,556,378	0.07	0.79
330	Chr7_19253346	85.18	Chr7	19,253,346	18,058,481	0.07	0.79
331	Chr7_19347365	85.59	Chr7	19,347,365	18,152,556	0.03	0.86
332	Chr7_21026599	100.22	Chr7	21,026,599	19,816,815	0.52	0.47
333	Chr7_21035053	101	Chr7	21,035,053	19,825,254	1.35	0.24
334	Chr7_23633502	118.34	Chr7	23,633,502	22,314,692	0.52	0.47

Table S8. QTL mapping results for volatile organic compounds (VOCs) in WI7799 segregating populations across multiple environments

#	QTL	VOCs	Mapping Populations	Environments or Replications	QTL cluster	Chr	Nearest marker	LOD score	PVE (%)	Left border (9930v3)	Left LOD	Right border (9930v3.0)	Right LOD	*A*	*D*
1	***E2heal1.1***	E2heal	RIL	H.R1	*voc1.1a*	1	62,710	4.64	9.49	62,710	4.64	1,814,448	2.79	-4.23	
2	***E2heal1.2***	E2heal	RIL	GH.R2	*voc1.1a*	1	1,814,448	3.65	8.33	62,710	3.22	3,981,831	1.94	-3.79	
3	***E2heal1.3***	E2heal	F3	H.R2		1	11,021,329	7.10	17.83	9,839,304	4.78	15,982,265	5.59	-1.94	-2.92
4	***E2heal1.4***	E2heal	F2	GH		1	15,527,492	5.04	12.73	14,773,447	3.09	15,605,841	0.00	4.83	1.30
5	***E2heal1.5***	E2heal	F2	GH		1	15,605,841	5.43	13.79	15,527,492	0.00	15,545,604	1.81	-4.96	-1.66
6	***E2heal3.1***	E2heal	F3	GH		3	6,017,474	4.97	13.50	3,846,735	3.36	7,837,677	3.22	4.86	-0.66
7	***E2heal3.2***	E2heal	RIL	GH.R1	*voc3.1a*	3	14,831,952	5.47	12.56	12,914,770	3.06	16,270,109	3.85	-4.29	
8	***E2heal3.3***	E2heal	RIL	H.R2	*voc3.1a*	3	16,270,109	4.42	10.23	13,737,934	2.98	27,059,020	3.34	-4.42	
9	***E2heal3.4***	E2heal	RIL	GH.R2	*voc3.1a*	3	16,270,109	3.97	9.12	13,879,294	2.23	27,059,020	1.79	-4.08	
10	***E2heal3.5***	E2heal	RIL	H.R1		3	21,975,544	5.17	10.68	13,737,934	3.58	27,059,020	3.22	-4.58	
11	***E2heal3.6***	E2heal	F3	H.R2	*voc3.2a*	3	32,499,091	4.32	10.31	31,240,931	2.44	35,996,982	2.51	-3.98	-2.12
12	***E2heal3.7***	E2heal	RIL	H.R1	*voc3.2a*	3	35,723,906	6.68	14.18	34,224,464	3.81	40,306,100	5.82	-5.66	
13	***E2heal3.8***	E2heal	RIL	H.R2	*voc3.2a*	3	38,652,277	7.59	18.55	35,723,906	6.29	40,306,100	6.66	-6.03	
14	***E2heal3.9***	E2heal	RIL	GH.R2	*voc3.2a*	3	39,443,369	3.86	8.84	35,723,906	1.92	40,306,100	2.77	-3.36	
15	***E2heal4.1***	E2heal	F3	H.R1	*voc4.2b*	4	17,676,173	4.55	10.68	15,613,509	1.67	23,167,471	2.01	2.44	-3.63
16	***E2heal5.1***	E2heal	F3	H.R2		5	1,893,048	6.79	16.95	1,133,443	4.86	2,117,964	4.94	-2.09	-2.55
17	***E2heal6.1***	E2heal	RIL	GH.R2	*voc6.1a*	6	20,957,428	3.47	7.89	15,869,056	0.03	29,808,101	0.99	-3.57	
18	***E2heal6.2***	E2heal	F3	GH	*voc6.2*	6	23,870,074	3.75	11.83	23,093,662	2.23	24,572,240	2.12	-4.15	-2.82
19	***E2heal7.1***	E2heal	RIL	GH.R1	*voc7.1b*	7	2,362,263	4.87	11.08	1,870,234	2.54	3,191,128	3.42	4.23	
20	***E2heol2.1***	E2heol	F3	GH		2	7,015,092	9.92	42.30	6,741,404	6.40	7,199,035	6.53	-7.38	3.94
21	***E2heol2.2***	E2heol	F3	GH		2	8,278,002	7.34	28.83	6,314,939	0.25	8,361,432	1.27	6.29	-4.21
22	***E2neal1.1***	E2neal	RIL	GH.R1	*voc1.1b*	1	1,800,963	9.33	18.93	62,710	8.55	2,508,462	4.11	4.29	
23	***E2neal1.2***	E2neal	F2	GH		1	5,394,075	3.79	9.14	148,606	1.86	16,261,375	1.91	-3.93	-1.89
24	***E2neal1.3***	E2neal	F3	H.R1	*voc1.2a*	1	30,123,683	6.07	18.96	27,750,202	4.47	32,509,306	4.82	-5.32	-0.56
25	***E2neal1.4***	E2neal	F3	GH	*voc1.2a*	1	32,137,606	9.66	23.81	32,132,344	7.81	32,509,306	7.82	-0.73	0.22
26	***E2neal3.1***	E2neal	F3	H.R2	*voc3.1a*	3	12,948,518	5.44	17.41	10,755,250	3.20	17,808,932	3.44	-5.19	-0.20
27	***E2neal3.2***	E2neal	RIL	H.R2	*voc3.1a*	3	14,831,952	4.20	13.07	12,209,494	3.10	16,270,109	2.65	-4.49	
28	***E2neal3.3***	E2neal	F3	GH	*voc3.1a*	3	14,831,952	7.59	17.94	13,810,691	6.08	15,621,241	4.84	-6.06	0.46
29	***E2neal3.4***	E2neal	RIL	GH.R1	*voc3.1a*	3	14,831,952	4.58	13.11	12,209,494	2.96	16,270,109	1.78	-4.62	
30	***E2neal3.5***	E2neal	RIL	GH.R2	*voc3.1a*	3	16,270,109	12.66	34.23	14,831,952	8.24	17,801,004	9.65	-4.47	
31	***E2neal3.6***	E2neal	F2	GH	*voc3.1a*	3	16,757,620	4.78	11.71	14,548,237	3.17	20,247,363	3.03	-4.33	1.89
32	***E2neal4.1***	E2neal	F3	H.R2	*voc4.2a*	4	17,205,059	4.16	11.49	15,560,273	2.48	17,416,330	2.58	-1.07	-4.42
33	***E2neal4.2***	E2neal	RIL	H.R1	*voc4.3a*	4	24,397,821	14.11	32.82	23,317,980	4.20	24,397,821	10.78	-2.18	
34	***E2neal4.3***	E2neal	F2	GH	*voc4.3a*	4	26,249,310	5.90	14.77	24,891,787	2.59	26,249,184	5.52	-0.67	-1.68
35	***E2neal5.1***	E2neal	RIL	H.R2		5	3,763,914	4.77	8.56	1,271,255	3.62	7,359,200	3.35	3.78	
36	***E2neal5.2***	E2neal	F3	GH		5	28,451,004	9.57	23.53	28,380,581	7.27	28,447,330	6.52	-0.30	0.45
37	***E2neal6.1***	E2neal	RIL	GH.R1	*voc6.1a*	6	20,957,428	4.21	11.33	15,869,056	1.21	26,837,386	2.57	-3.83	
38	***E2neal6.2***	E2neal	RIL	H.R1	*voc6.2*	6	23,869,621	10.03	21.71	23,553,542	3.78	25,829,271	2.81	-6.10	
39	***E2neal7.1***	E2neal	F2	GH	*voc7.3a*	7	19,816,815	6.14	15.41	18,668,087	4.36	20,416,776	3.87	-2.81	-0.84
40	***E3heal1.1***	E3heal	F2	GH		1	10,925,063	7.24	22.74	9,839,304	5.25	10,999,616	5.19	-3.19	1.20
41	***E3heal3.1***	E3heal	F3	H.R1		3	33,342,419	4.24	11.98	32,499,091	2.57	35,404,990	2.58	-0.92	-4.41
42	***E3heal4.1***	E3heal	F3	H.R2		4	6,238,867	6.69	20.29	5,885,870	4.64	6,190,273	4.56	3.33	0.11
43	***E3heal4.2***	E3heal	RIL	H.R1	*voc4.3a*	4	24,891,253	10.20	16.79	23,317,980	2.47	25,849,536	9.34	-2.22	

44	***E3heal5.1***	E3heal	F3	H.R2		5	1,893,048	4.08	12.73	69,729	1.78	4,554,819	2.56	-3.07	-3.18
45	***E3heal5.2***	E3heal	F2	GH		5	29,316,282	6.65	20.65	29,045,825	4.14	29,416,835	4.32	1.44	0.88
46	***E3heal6.1***	E3heal	F3	H.R1		6	26,312,563	3.96	11.12	24,627,600	2.03	30,855,972	2.32	-4.34	-2.76
47	***E3heal7.1***	E3heal	F3	H.R2		7	14,373,496	6.80	20.67	13,439,783	4.87	15,274,642	5.19	3.77	-0.82
48	***E3heol1.1***	E3heol	F3	H.R1		1	28,674,401	4.11	26.15	26,180,617	2.02	32,170,754	2.19	-4.53	-0.43
49	***E3heol3.1***	E3heol	F3	H.R1		3	40,164,574	4.72	31.11	38,223,849	3.01	40,103,997	3.65	-4.97	0.90
50	***E6neal2.1***	E6neal	F2	GH		2	8,320,927	11.42	37.80	6,314,939	0.48	8,278,002	1.26	-3.46	-2.87
51	***E6neal2.2***	E6neal	RIL	GH.R2	*voc2.2*	2	10,099,499	10.47	31.20	2,710,577	9.24	11,255,780	4.40	1.73	
52	***E6neal5.1***	E6neal	RIL	H.R1		5	12,919,926	9.60	41.01	12,919,926	5.71	15,435,643	6.60	3.67	
53	***E6neal6.1***	E6neal	RIL	H.R1	*voc6.2*	6	23,869,621	9.40	39.86	23,553,514	7.89	23,869,621	7.71	-1.53	
54	***E6neal7.1***	E6neal	RIL	GH.R1	*voc7.2*	7	8,540,084	7.05	20.11	7,781,806	5.85	11,682,399	1.77	3.37	
55	***E6neal7.2***	E6neal	RIL	GH.R1		7	12,890,052	7.09	20.26	12,890,052	4.89	16,249,736	6.52	-0.79	
56	***EE24ndeal1.1***	EE24ndeal	F3	H.R2		1	7,801,545	10.12	29.82	7,273,355	7.82	8,108,150	8.26	-1.59	5.27
57	***EE24ndeal2.1***	EE24ndeal	F3	H.R2		2	7,994,419	7.48	20.99	8,320,927	4.17	8,842,310	5.58	-2.45	-2.93
58	***EE24ndeal2.2***	EE24ndeal	RIL	GH.R1		2	14,337,244	12.31	34.27	13,272,902	10.87	14,337,244	6.32	-0.61	
59	***EE24ndeal3.1***	EE24ndeal	F2	GH		3	20,917,609	16.56	36.25	20,247,363	9.36	21,962,723	6.47	5.45	3.12
60	***EE24ndeal3.2***	EE24ndeal	F2	GH		3	22,729,218	16.26	35.43	22,701,717	8.55	23,691,056	11.96	-5.45	-0.05
61	***EE24ndeal4.1***	EE24ndeal	F2	GH		4	4,159,581	3.45	6.19	2,142,457	1.40	26,249,184	1.67	2.86	-2.77
62	***EE24ndeal4.2***	EE24ndeal	RIL	H.R2		4	20,016,081	14.47	45.28	19,737,613	13.46	20,926,389	7.30	-7.10	
63	***EE24ndeal5.1***	EE24ndeal	RIL	GH.R1		5	5,230,193	10.57	28.46	264,610	9.77	19,520,759	7.07	6.98	
64	***EE24ndeal5.2***	EE24ndeal	RIL	H.R1		5	5,467,191	8.61	29.18	5,467,191	5.53	6,903,854	5.60	-4.09	
65	***EE24ndeal6.1***	EE24ndeal	RIL	H.R2		6	22,058,543	14.01	43.37	21,726,330	13.40	23,078,385	6.34	-0.20	
66	***EE26ndeal1.1***	EE26ndeal	F3	H.R2		1	27,750,262	9.10	25.54	27,668,738	7.39	27,750,202	6.82	4.55	-1.24
67	***EE26ndeal2.1***	EE26ndeal	F3	H.R2	*voc2.1a*	2	4,571,046	8.38	23.21	3,301,921	6.71	6,314,958	6.04	-3.87	-0.42
68	***EE26ndeal3.1***	EE26ndeal	RIL	H.R1	*voc3.2a*	3	31,853,185	4.01	12.70	28,807,221	2.08	38,652,277	1.66	-3.83	
69	***EE26ndeal3.2***	EE26ndeal	F3	GH	*voc3.2a*	3	35,630,635	4.61	16.08	33,342,419	2.95	40,103,997	3.33	-4.72	-0.70
70	***EE26ndeal7.1***	EE26ndeal	F2	GH	*voc7.2*	7	8,848,963	7.73	17.61	5,291,372	4.28	8,849,161	2.62	2.11	-2.16
71	***EE36ndeol7.1***	EE36ndeol	F2	GH	*voc7.1b*	7	735,605	7.93	25.09	361,737	7.89	2,819,375	5.70	1.71	-2.63
72	***EE36ndeol7.2***	EE36ndeol	F3	H.R1	*voc7.1b*	7	2,355,322	12.34	53.55	1,870,234	7.25	2,819,375	6.78	4.16	-6.41
73	***EE36ndeol7.3***	EE36ndeol	F3	H.R1		7	12,894,447	11.83	50.37	11,916,437	8.75	14,373,496	5.73	1.12	3.01
74	***EE36ndeol7.4***	EE36ndeol	F2	GH		7	15,206,679	23.77	61.26	14,819,586	14.12	15,206,539	15.00	3.62	-4.16
75	***EE36ndeol7.5***	EE36ndeol	F2	GH		7	16,584,849	22.61	56.71	16,272,962	18.19	16,619,540	15.26	-3.17	3.75
76	***EZ26ndeal1.1***	EZ26ndeal	F3	H.R1	*voc1.2a*	1	30,123,683	3.52	8.35	25,710,146	1.66	32,509,306	2.17	-3.97	0.47
77	***EZ26ndeal1.2***	EZ26ndeal	F3	GH	*voc1.2a*	1	32,137,436	8.44	16.23	29,928,193	6.50	32,509,306	6.00	-0.90	-0.06
78	***EZ26ndeal2.1***	EZ26ndeal	F3	H.R1		2	17,944,215	7.49	21.91	17,433,691	5.25	19,170,675	5.13	0.13	-1.37
79	***EZ26ndeal3.1***	EZ26ndeal	F3	H.R1		3	9,000,781	8.33	24.75	7,784,369	6.50	9,639,091	6.15	-2.69	1.14
80	***EZ26ndeal3.2***	EZ26ndeal	RIL	H.R2	*voc3.1a*	3	13,879,294	8.39	15.98	12,914,770	4.15	14,831,952	4.97	-2.40	
81	***EZ26ndeal3.3***	EZ26ndeal	RIL	GH.R1	*voc3.1a*	3	14,831,952	7.56	20.09	12,914,770	5.32	16,270,109	4.35	-1.59	
82	***EZ26ndeal3.4***	EZ26ndeal	RIL	GH.R2	*voc3.1a*	3	16,270,109	10.62	29.29	14,831,952	7.92	17,801,004	7.63	-4.14	
83	***EZ26ndeal3.5***	EZ26ndeal	F3	GH	*voc3.2a*	3	35,079,083	14.24	30.82	35,003,175	11.28	35,424,095	11.47	-5.59	-1.91
84	***EZ26ndeal4.1***	EZ26ndeal	F3	H.R1		4	4,266,477	6.25	15.59	3,285,372	1.37	4,228,682	1.36	0.94	3.38
85	***EZ26ndeal4.2***	EZ26ndeal	F3	H.R1		4	14,731,312	7.40	18.84	13,743,498	4.87	14,904,144	5.28	-1.04	-2.47
86	***EZ26ndeal4.3***	EZ26ndeal	F3	H.R2	*voc4.2a*	4	17,394,480	9.79	28.38	16,352,114	6.20	20,819,509	6.33	-0.54	-4.40
87	***EZ26ndeal4.4***	EZ26ndeal	RIL	H.R2	*voc4.3a*	4	24,397,821	4.37	12.06	23,038,993	3.06	24,891,253	2.54	-3.74	
88	***EZ26ndeal4.5***	EZ26ndeal	F2	GH	*voc4.3a*	4	25,960,080	7.20	17.97	24,891,787	3.27	26,249,184	5.73	-1.52	-2.01
89	***EZ26ndeal5.1***	EZ26ndeal	F3	GH		5	28,586,898	8.62	16.64	27,296,600	6.86	29,045,825	6.06	-0.50	1.28
90	***EZ26ndeal6.1***	EZ26ndeal	RIL	GH.R1	*voc6.1b*	6	20,957,428	8.92	24.30	15,869,056	5.12	21,419,544	6.34	2.78	
91	***EZ26ndeal6.2***	EZ26ndeal	RIL	H.R1	*voc6.2*	6	23,869,621	7.64	14.35	23,553,542	4.51	25,829,271	3.27	-3.37	
92	***EZ26ndeal7.1***	EZ26ndeal	RIL	GH.R1	*voc7.1a*	7	2,362,263	4.50	11.01	874,961	0.49	3,019,179	1.43	-0.65	

93	***EZ26ndeal7.2***	EZ26ndeal	F3	GH		7	4,839,914	7.61	14.39	4,131,473	5.41	22,286,698	4.67	-0.75	0.16
94	***EZ26ndeal7.3***	EZ26ndeal	F2	GH	*voc7.3a*	7	19,584,382	6.87	17.07	18,668,087	5.11	20,416,776	4.28	-2.57	-1.58
95	***EZ26ndeol1.1***	EZ26ndeol	RIL	H.R1		1	4,972,196	9.84	24.77	3,981,831	4.47	5,394,075	8.34	5.72	
96	***EZ26ndeol1.2***	EZ26ndeol	F2	GH		1	19,941,374	6.91	13.34	16,561,199	2.54	20,522,287	4.80	-0.80	-3.60
97	***EZ26ndeol1.3***	EZ26ndeol	F3	GH	*voc1.2b*	1	32,132,344	10.19	23.64	29,928,193	4.91	32,509,306	9.46	3.65	-3.96
98	***EZ26ndeol2.1***	EZ26ndeol	F3	GH	*voc2.1a*	2	4,371,493	4.99	28.31	3,918,910	2.62	4,571,046	3.37	-3.77	-2.79
99	***EZ26ndeol2.2***	EZ26ndeol	F2	GH		2	11,845,636	6.86	13.24	10,271,021	3.64	12,964,173	5.31	-1.91	-1.66
100	***EZ26ndeol3.1***	EZ26ndeol	F2	GH		3	23,691,056	4.20	7.76	10,255,482	2.04	31,976,385	2.52	0.58	-0.38
101	***EZ26ndeol4.1***	EZ26ndeol	F2	GH		4	25,781,223	6.00	17.17	25,781,328	4.50	26,249,184	4.42	-1.24	-2.07
102	***EZ26ndeol5.1***	EZ26ndeol	F3	H.R1		5	2,839,348	9.96	19.55	2,476,557	7.13	3,322,795	6.60	2.37	2.23
103	***EZ26ndeol5.2***	EZ26ndeol	RIL	H.R2		5	5,467,191	8.10	27.67	3,763,914	1.87	6,903,854	4.92	-3.79	
104	***EZ26ndeol5.3***	EZ26ndeol	RIL	H.R2		5	12,919,926	6.96	23.15	6,887,777	3.63	15,435,643	5.99	-2.66	
105	***EZ26ndeol5.4***	EZ26ndeol	F3	H.R1	*voc5.1a*	5	21,262,634	19.62	49.42	20,014,870	10.74	21,531,051	17.63	3.74	-2.99
106	***EZ26ndeol5.5***	EZ26ndeol	RIL	H.R1	*voc5.1a*	5	23,064,539	10.56	26.95	22,259,813	9.88	24,759,262	7.04	0.96	
107	***EZ26ndeol6.1***	EZ26ndeol	F3	GH		6	21,762,821	10.80	49.56	21,867,578	7.12	22,178,946	7.39	-3.52	0.27
108	***EZ26ndeol6.2***	EZ26ndeol	F2	GH		6	25,825,565	5.44	16.38	24,572,240	3.89	26,310,762	3.10	-5.17	-2.26
109	***EZ26ndeol6.3***	EZ26ndeol	F3	GH		6	25,825,565	9.87	43.76	24,623,145	4.53	25,832,865	7.08	3.52	1.44
110	***EZ26ndeol7.1***	EZ26ndeol	F2	GH	*voc7.1a*	7	2,355,322	6.80	13.11	1,473,123	2.46	3,132,222	3.90	-0.22	-1.42
111	***EZ26ndeol7.2***	EZ26ndeol	F3	GH	*voc7.2*	7	8,849,179	14.62	62.79	8,848,963	3.47	9,817,231	3.94	2.34	1.08
112	***EZ26ndeol7.3***	EZ26ndeol	RIL	H.R1	*voc7.3b*	7	18,152,556	5.37	12.35	17,556,378	3.83	22,314,692	2.42	1.42	
113	***EZ26ndeol7.4***	EZ26ndeol	F2	GH	*voc7.3a*	7	19,757,897	6.32	18.19	18,668,087	4.11	20,310,022	4.46	-3.53	-0.99
114	***EZ36ndeol1.1***	EZ36ndeol	F3	H.R1	*voc1.1b*	1	1,709,026	10.39	26.93	143,552	7.14	2,625,328	5.35	1.46	-2.18
115	***EZ36ndeol1.2***	EZ36ndeol	F3	GH	*voc1.2b*	1	32,506,255	5.46	16.19	28,674,401	3.91	32,509,306	4.77	3.65	-4.36
116	***EZ36ndeol2.1***	EZ36ndeol	RIL	GH.R1	*voc2.1a*	2	2,710,577	17.60	44.00	2,710,577	17.60	1,814,448	6.47	-8.70	
117	***EZ36ndeol2.2***	EZ36ndeol	F3	H.R1	*voc2.1a*	2	3,462,983	4.87	17.33	2,825,198	3.02	4,235,915	2.64	-4.61	-2.24
118	***EZ36ndeol2.3***	EZ36ndeol	F2	GH	*voc2.1a*	2	6,038,117	4.23	6.24	1,957,580	1.97	6,314,958	1.25	-4.34	-1.99
119	***EZ36ndeol3.1***	EZ36ndeol	RIL	H.R1	*voc3.1b*	3	14,831,952	4.81	9.69	10,394,214	3.07	16,270,109	2.95	4.65	
120	***EZ36ndeol3.2***	EZ36ndeol	F3	GH	*voc3.1b*	3	15,961,767	5.61	16.66	14,528,589	3.38	20,247,363	3.31	5.25	0.20
121	***EZ36ndeol3.3***	EZ36ndeol	F3	H.R2	*voc3.1b*	3	16,321,525	11.30	32.67	14,636,569	6.31	23,691,056	8.85	2.64	-2.56
122	***EZ36ndeol3.4***	EZ36ndeol	F2	GH		3	30,191,074	13.75	18.91	30,050,166	11.79	30,534,122	10.17	2.01	-2.98
123	***EZ36ndeol4.1***	EZ36ndeol	F3	GH	*voc4.1*	4	4,266,477	4.55	11.75	738,359	2.67	8,728,554	2.70	4.42	-1.24
124	***EZ36ndeol4.2***	EZ36ndeol	F3	GH	*voc4.1*	4	5,768,902	10.18	18.21	5,444,935	7.19	6,239,398	7.27	3.46	0.98
125	***EZ36ndeol5.1***	EZ36ndeol	RIL	GH.R1		5	21,061,292	4.76	8.01	20,437,489	2.99	25,606,087	1.51	-4.06	
126	***EZ36ndeol5.2***	EZ36ndeol	F3	H.R1	*voc5.1a*	5	24,169,934	14.28	32.62	21,841,655	10.70	24,169,934	12.35	4.85	-3.30
127	***EZ36ndeol6.1***	EZ36ndeol	RIL	GH.R1		6	5,224,564	14.05	32.82	4,955,743	4.69	5,224,564	1.28	0.10	
128	***EZ36ndeol6.2***	EZ36ndeol	F3	H.R2		6	16,762,009	9.25	25.69	9,040,033	6.26	17,333,402	5.03	-2.31	-1.50
129	***EZ36ndeol7.1***	EZ36ndeol	F3	H.R1		7	20,727,167	6.61	12.45	20,416,776	4.05	22,275,017	5.90	2.01	2.55
130	***hal1.1***	hal	RIL	H.R1		1	8,280,718	8.24	15.98	6,650,871	5.89	16,559,383	5.14	-1.73	
131	***hal1.2***	hal	RIL	GH.R2		1	21,727,015	6.53	11.21	16,559,383	4.48	23,256,450	4.12	-1.25	
132	***hal3.1***	hal	F3	GH		3	10,268,364	5.66	14.75	3,885,315	4.06	10,755,206	3.48	5.19	-0.82
133	***hal3.2***	hal	RIL	H.R1	*voc3.2b*	3	31,853,185	3.35	5.98	12,209,494	1.13	33,930,903	1.78	3.75	
134	***hal3.3***	hal	RIL	GH.R1	*voc3.2b*	3	34,224,464	5.44	15.78	32,178,481	3.90	37,606,195	3.09	5.11	
135	***hal3.4***	hal	F2	GH	*voc3.2b*	3	34,246,720	9.33	22.39	32,488,686	7.80	35,996,982	5.66	3.87	-2.75
136	***hal3.5***	hal	RIL	GH.R2	*voc3.2b*	3	37,606,195	7.62	13.36	35,723,906	4.21	38,652,277	5.31	4.77	
137	***hal4.1***	hal	F3	GH	*voc4.1*	4	5,655,745	4.58	11.69	4,666,652	2.58	7,163,873	2.99	4.23	-1.71
138	***hal4.2***	hal	RIL	GH.R2		4	8,154,447	12.96	25.28	8,154,447	7.70	8,729,548	6.34	-3.02	
139	***hal4.3***	hal	F3	H.R1	*voc4.2b*	4	17,418,291	9.10	19.72	17,205,059	6.96	17,676,173	6.29	4.71	-2.45
140	***hal4.4***	hal	RIL	GH.R2	*voc4.2b*	4	17,586,005	11.02	20.66	17,586,005	8.41	18,192,798	9.44	4.43	
141	***hal4.5***	hal	RIL	H.R1		4	21,573,996	7.53	14.42	20,926,389	6.31	22,037,092	6.05	1.04	

142	***hal5.1***	hal	F2	GH	*voc5.1a*	5	21,262,634	8.34	19.69	20,014,870	6.48	21,531,051	5.50	1.72	-4.47
143	***hal6.1***	hal	F3	H.R1		6	4,570,324	5.05	15.04	3,808,119	2.14	5,347,011	2.02	2.20	-4.37
144	***hal6.2***	hal	F3	H.R1		6	5,935,190	6.39	19.49	5,617,652	3.84	6,178,739	3.26	-1.92	5.28
145	***hal6.3***	hal	F3	GH	*voc6.2*	6	23,374,914	4.82	12.36	22,178,946	2.70	24,132,776	3.10	-3.08	-4.67
146	***hal6.4***	hal	RIL	H.R2		6	30,850,320	7.70	21.93	29,808,101	4.65	30,850,320	7.65	0.11	
147	***hal7.1***	hal	RIL	GH.R1		7	361,940	3.40	9.51	874,962	3.30	17,556,378	0.23	3.97	
148	***hal7.2***	hal	RIL	H.R1		7	876,953	9.38	18.56	361,940	6.78	1,300,411	1.90	1.29	
149	***hal7.3***	hal	RIL	H.R1	*voc7.1a*	7	2,362,263	8.28	16.07	1,300,411	7.10	3,020,781	5.91	-1.72	
150	***hal7.4***	hal	F2	GH	*voc7.2*	7	8,237,164	8.40	19.85	8,849,234	1.28	11,873,378	4.31	4.29	-4.41
151	***hol1.1***	hol	F3	GH		1	2,625,328	15.29	44.06	2,190,158	2.38	2,795,389	3.84	-6.30	6.10
152	***hol1.2***	hol	F3	GH		1	2,789,870	14.76	42.08	2,779,727	9.13	2,863,804	9.09	6.22	-5.90
153	***hol2.1***	hol	F2	GH	*voc2.1a*	2	3,462,983	10.72	9.58	2,825,198	7.36	5,998,141	8.43	-6.91	-2.57
154	***hol2.2***	hol	F3	H.R1	*voc2.1a*	2	4,371,493	4.48	12.47	2,825,198	1.71	7,015,092	2.70	-4.34	-2.24
155	***hol2.3***	hol	RIL	H.R1	*voc2.1a*	2	4,371,493	9.48	40.90	3,396,451	6.27	4,824,086	7.22	-7.17	
156	***hol2.4***	hol	RIL	H.R2	*voc2.1a*	2	4,371,493	9.87	32.84	3,396,451	6.63	5,789,934	6.48	-7.43	
157	***hol2.5***	hol	RIL	GH.R1	*voc2.1a*	2	4,371,493	33.95	53.39	3,674,089	24.40	4,650,059	30.28	-6.09	
158	***hol2.6***	hol	RIL	GH.R2	*voc2.1a*	2	4,650,059	33.35	72.49	4,371,493	31.21	5,789,934	22.96	-17.44	
159	***hol2.7***	hol	RIL	GH.R1		2	14,337,244	7.04	6.46	12,552,513	6.59	17,485,610	6.50	-0.94	
160	***hol3.1***	hol	F3	H.R1		3	17,815,818	15.68	40.12	17,262,318	9.59	20,917,609	9.04	6.73	-1.89
161	***hol5.1***	hol	F2	GH		5	69,729	8.26	11.37	104,789	6.70	393,358	4.43	2.20	-3.77
162	***hol5.2***	hol	F3	H.R1	*voc5.1a*	5	21,531,051	5.93	16.95	20,014,870	3.66	24,162,980	3.01	4.40	-3.32
163	***hol6.1***	hol	F2	GH		6	1,844,757	7.25	6.17	1,844,757	7.25	2,214,688	4.63	-2.20	-2.20
164	***hol6.2***	hol	F2	GH		6	6,254,764	8.31	7.17	5,614,376	5.75	6,533,905	6.49	2.20	-2.20
165	***hol6.3***	hol	RIL	GH.R1		6	21,419,544	5.76	5.16	20,957,428	2.43	22,058,543	3.95	-0.66	
166	***hol6.4***	hol	F3	GH		6	27,639,644	18.45	50.10	27,160,467	6.45	27,653,151	2.87	1.30	2.96
167	***hol6.5***	hol	F3	GH		6	27,653,151	18.45	50.10	27,639,644	16.07	28,262,683	6.21	-1.30	4.60
168	***hol7.1***	hol	F2	GH		7	4,131,473	16.72	16.31	3,214,998	1.81	4,131,473	14.75	-4.30	-3.72
169	***hol7.2***	hol	F3	H.R1		7	5,291,372	14.47	36.19	3,214,998	8.57	8,848,963	11.37	-6.07	-1.97
170	***nal1.1***	nal	RIL	GH.R1		1	14,770,584	5.53	16.04	8,867,313	3.29	16,559,383	2.62	-3.51	
171	***nal2.1***	nal	RIL	GH.R1	*voc2.1b*	2	2,710,577	3.60	10.09	2,710,577	3.60	10,099,499	1.25	4.07	
172	***nal2.2***	nal	RIL	GH.R2	*voc2.2*	2	10,099,499	6.19	18.61	7,998,780	0.66	11,255,780	1.38	2.59	
173	***nal2.3***	nal	F3	H.R2	*voc2.2*	2	10,238,310	17.22	42.48	9,699,386	7.22	11,209,309	15.44	6.33	-1.82
174	***nal3.1***	nal	RIL	GH.R2		3	12,914,770	3.36	9.57	3,081,180	0.37	21,103,235	0.85	-1.98	
175	***nal3.2***	nal	F3	H.R1	*voc3.2a*	3	37,584,346	4.39	10.22	35,996,982	2.58	39,444,373	2.13	-4.37	-0.91
176	***nal4.1***	nal	RIL	H.R1	*voc4.3a*	4	24,473,476	12.38	24.37	23,317,980	2.26	24,891,253	9.97	-5.69	
177	***nal5.1***	nal	F3	H.R1	*voc5.1a*	5	21,841,655	6.93	13.05	21,262,634	5.23	24,169,934	5.21	4.39	-3.50
178	***nal5.2***	nal	RIL	H.R1	*voc5.1a*	5	23,064,539	5.17	15.96	21,347,037	3.57	24,759,262	3.69	4.61	
179	***neol3.1***	neol	F3	H.R1		3	33,709,250	9.37	52.53	32,488,686	3.72	35,621,667	2.16	-3.98	-3.95
180	***neol4.1***	neol	F2	GH		4	23,324,467	9.99	30.57	22,499,162	7.24	25,804,285	6.03	1.78	-2.80
181	***neol5.1***	neol	F3	H.R1	*voc5.1b*	5	21,841,655	8.99	49.14	14,116,630	6.58	24,169,934	3.53	-3.19	-5.39
182	***neol5.2***	neol	RIL	GH.R1		5	24,759,262	3.66	9.87	22,259,813	1.93	26,864,806	0.41	3.62	
183	***neol5.3***	neol	F2	GH		5	31,820,579	8.09	23.87	24,162,980	4.78	31,643,597	5.33	0.30	-1.12
184	***neol6.1***	neol	RIL	GH.R1	*voc6.1a*	6	20,957,428	4.17	11.34	15,869,056	1.30	21,419,544	1.96	-3.55	
185	***neol7.1***	neol	RIL	GH.R1		7	12,890,052	3.86	10.45	11,682,399	2.27	19,825,254	1.58	3.30	
186	***nol1.1***	nol	F3	GH	*voc1.1a*	1	1,450,884	6.21	13.52	143,552	4.18	2,203,172	3.43	-5.37	-0.51
187	***nol1.2***	nol	F3	H.R1		1	19,501,963	10.05	28.95	16,561,199	7.25	19,501,963	5.45	5.06	3.86
188	***nol1.3***	nol	F3	H.R1		1	19,941,374	9.01	25.46	19,375,179	5.54	20,522,287	2.09	-5.03	-0.12
189	***nol1.4***	nol	RIL	GH.R1		1	20,334,148	11.64	32.28	19,759,990	6.53	21,727,015	9.82	-5.12	

190	***nol1.5***	nol	F3	H.R1	*voc1.2b*	1	32,137,728	8.74	17.92	31,487,372	6.85	32,506,255	5.90	0.16	-1.11
191	***nol2.1***	nol	RIL	H.R2	*voc2.1a*	2	3,396,451	24.06	58.83	3,396,451	23.86	4,371,493	16.28	-10.89	
192	***nol2.2***	nol	RIL	H.R1	*voc2.1a*	2	4,371,493	28.25	63.09	3,396,451	26.31	4,371,493	27.10	-11.19	
193	***nol2.3***	nol	RIL	GH.R1		2	14,337,244	10.21	27.54	13,272,902	5.77	16,893,264	4.76	-0.22	
194	***nol3.1***	nol	F2	GH		3	4,175,838	5.01	22.41	3,885,315	2.16	4,175,928	3.03	3.66	5.01
195	***nol3.2***	nol	F2	GH		3	4,328,076	5.01	22.41	4,175,838	0.00	5,210,097	1.19	-3.66	-5.01
196	***nol3.3***	nol	RIL	GH.R2	*voc3.1a*	3	16,270,109	4.50	18.57	12,914,770	1.87	21,103,235	2.38	-1.18	
197	***nol4.1***	nol	RIL	H.R1		4	2,762,508	11.60	18.52	1,832,529	3.20	2,790,441	11.48	-1.08	
198	***nol5.1***	nol	RIL	H.R1		5	19,520,759	15.04	25.01	19,773,641	15.04	19,520,759	15.04	-0.20	
199	***nol5.2***	nol	RIL	H.R1	*voc5.1a*	5	24,759,262	14.76	24.42	23,064,539	9.81	28,735,158	3.35	3.63	
200	***nol6.1***	nol	RIL	H.R2		6	5,224,564	14.43	28.62	5,043,365	4.62	6,507,586	13.68	0.20	
201	***Z2heal1.1***	Z2heal	RIL	H.R2	*voc1.1a*	1	62,710	3.78	10.01	62,710	3.78	11,774,072	1.09	-3.54	
202	***Z2heal1.2***	Z2heal	RIL	GH.R2	*voc1.1a*	1	1,800,963	4.20	10.69	62,710	3.31	4,972,196	2.82	-4.37	
203	***Z2heal1.3***	Z2heal	F2	GH		1	7,454,118	7.19	40.65	6,261,686	5.30	9,296,871	5.04	-3.74	-1.86
204	***Z2heal3.1***	Z2heal	F3	GH		3	6,017,474	6.34	16.57	4,344,167	4.46	7,837,677	4.59	5.58	-0.66
205	***Z2heal3.2***	Z2heal	RIL	H.R2	*voc3.1a*	3	14,831,952	7.10	19.94	13,879,294	6.46	16,270,109	4.71	-5.97	
206	***Z2heal3.3***	Z2heal	RIL	GH.R2	*voc3.1a*	3	14,831,952	4.02	10.17	12,914,770	1.51	38,652,277	1.52	-3.53	
207	***Z2heal4.1***	Z2heal	RIL	GH.R1		4	3,295,707	4.42	10.19	1,832,529	2.23	14,399,731	2.81	-4.21	
208	***Z2heal4.2***	Z2heal	F3	GH	*voc4.1*	4	5,655,847	5.12	13.07	3,285,372	2.79	7,163,873	3.40	3.89	-2.77
209	***Z2heal4.3***	Z2heal	RIL	GH.R2		4	8,729,548	4.17	10.60	740,918	1.56	21,572,651	1.60	-3.20	
210	***Z2heal4.4***	Z2heal	F2	GH	*voc4.2a*	4	15,559,207	8.36	29.16	15,263,286	6.54	16,510,766	6.57	-3.57	3.19
211	***Z2heal5.1***	Z2heal	F2	GH		5	11,755,612	7.13	40.21	11,523,901	4.97	12,382,356	4.32	-0.57	-1.64
212	***Z2heal6.1***	Z2heal	RIL	GH.R1	*voc6.1a*	6	20,957,428	4.49	10.37	15,869,056	0.30	21,726,330	1.70	-3.49	
213	***Z2heal6.2***	Z2heal	F3	GH	*voc6.2*	6	23,374,914	3.44	8.50	22,178,946	1.88	25,825,565	1.26	-2.99	-3.70
214	***Z2heal7.1***	Z2heal	RIL	GH.R1	*voc7.1b*	7	3,029,741	4.38	10.09	1,870,234	2.54	7,781,806	2.96	4.33	
215	***Z2neal2.1***	Z2neal	RIL	GH.R1	*voc2.1a*	2	3,674,089	4.81	16.13	3,396,451	4.56	10,099,499	3.11	-4.63	
216	***Z2neal2.2***	Z2neal	RIL	H.R2	*voc2.2*	2	10,099,499	6.78	8.91	7,998,780	4.96	10,099,499	3.65	2.78	
217	***Z2neal3.1***	Z2neal	RIL	H.R1	*voc3.1a*	3	14,831,952	4.29	10.08	12,209,494	3.42	16,270,109	1.79	-4.42	
218	***Z2neal4.1***	Z2neal	RIL	H.R1	*voc4.3b*	4	24,397,821	13.48	29.12	23,317,980	7.50	24,397,821	10.75	1.89	
219	***Z3heal5.1***	Z3heal	F3	H.R1		5	9,194,546	6.58	14.78	6,889,298	5.08	11,523,901	3.65	0.15	0.49
220	***Z3heal5.2***	Z3heal	F3	H.R1		5	25,613,633	5.84	12.79	24,162,980	4.02	26,804,403	3.68	-2.59	-0.40
221	***Z3heal6.1***	Z3heal	F3	H.R1		6	20,317,016	15.28	46.34	19,159,725	13.01	20,924,265	11.95	8.46	-8.46
222	***Z3heal7.1***	Z3heal	F3	H.R1	*voc7.2*	7	8,849,155	13.94	40.31	5,291,372	2.32	8,849,234	1.83	8.46	-8.46
223	***Z3heol2.1***	Z3heol	F3	H.R1	*voc2.1a*	2	3,439,526	6.27	16.19	2,825,198	2.58	3,301,246	2.76	-5.46	2.60
224	***Z3heol2.2***	Z3heol	F2	GH	*voc2.1a*	2	4,235,915	4.73	14.79	2,708,949	2.18	7,706,537	2.79	-3.83	-3.42
225	***Z3heol2.3***	Z3heol	RIL	GH.R2	*voc2.1a*	2	4,270,582	16.07	35.74	3,674,089	11.81	4,650,059	13.49	-9.43	
226	***Z3heol2.4***	Z3heol	RIL	H.R2	*voc2.1a*	2	4,371,493	9.49	47.81	4,371,493	9.49	5,789,934	2.30	-4.84	
227	***Z3heol2.5***	Z3heol	F3	GH		2	6,531,947	5.55	19.53	4,694,359	2.90	8,633,934	3.40	-5.25	-0.96
228	***Z3heol3.1***	Z3heol	RIL	GH.R2		3	8,701,817	3.39	5.94	5,422,440	1.97	12,209,494	2.33	3.96	
229	***Z3heol3.2***	Z3heol	F3	GH	*voc3.1b*	3	14,810,570	3.97	15.84	11,439,629	2.05	30,074,658	2.27	4.22	-1.45
230	***Z3heol3.3***	Z3heol	F3	H.R1	*voc3.1b*	3	16,494,950	5.48	13.96	11,760,693	3.19	25,135,149	3.37	4.18	-2.62
231	***Z3heol3.4***	Z3heol	F3	GH		3	40,231,874	5.41	18.99	37,637,669	3.83	40,103,997	5.28	5.12	-0.83
232	***Z3heol5.1***	Z3heol	RIL	H.R2		5	11,523,346	9.16	45.05	7,359,200	9.07	12,327,989	8.36	-4.18	
233	***Z3heol7.1***	Z3heol	RIL	GH.R2	*voc7.3b*	7	18,058,481	3.40	5.97	12,890,052	1.62	22,314,692	1.84	3.96	
234	***Z3neol1.1***	Z3neol	F3	H.R2		1	4,974,472	4.54	14.26	4,940,110	0.88	4,974,479	0.00	-2.99	4.33
235	***Z3neol1.2***	Z3neol	F3	H.R2		1	4,974,479	4.32	13.50	4,974,472	0.22	4,974,480	1.45	3.23	-4.03
236	***Z3neol1.3***	Z3neol	F3	GH	*voc1.2b*	1	32,506,255	5.65	16.98	32,132,344	3.91	32,509,306	4.87	3.94	-4.26
237	***Z3neol2.1***	Z3neol	RIL	GH.R2	*voc2.1a*	2	4,270,582	3.40	9.26	2,710,577	2.91	7,998,780	1.46	-3.97	

238	***Z3neol3.1***	Z3neol	RIL	GH.R2	*voc3.1b*	3	13,879,294	4.61	12.84	10,703,689	3.20	14,831,952	2.69	4.11	
239	***Z3neol3.2***	Z3neol	F3	GH	*voc3.1b*	3	17,735,552	5.80	15.13	14,528,589	3.32	20,917,609	4.13	5.04	1.16
240	***Z3neol4.1***	Z3neol	F3	GH	*voc4.1*	4	4,266,477	3.90	9.81	738,359	1.58	22,104,691	1.92	3.80	-1.77
241	***Z3neol4.2***	Z3neol	F2	GH	*voc4.2b*	4	15,188,127	8.17	20.16	10,145,822	6.17	20,819,509	2.62	6.20	-1.93
242	***Z3neol4.3***	Z3neol	F2	GH	*voc4.2b*	4	15,203,570	4.72	8.08	14,690,935	3.22	18,285,752	3.03	4.19	-1.74
243	***Z3neol6.1***	Z3neol	F2	GH		6	663,669	14.31	28.14	652,763	6.35	793,224	10.24	-1.53	0.45
244	***Z3neol6.2***	Z3neol	F2	GH		6	2,087,152	13.87	27.11	1,925,225	7.89	2,214,688	10.58	2.57	3.88
245	***Z3neol6.3***	Z3neol	RIL	GH.R1		6	4,955,743	7.09	20.36	4,955,743	7.09	4,955,743	7.09	4.38	
246	***Z3neol6.4***	Z3neol	RIL	GH.R1		6	4,955,743	6.85	19.59	4,955,743	0.00	5,224,564	6.85	-4.24	
247	***Z6neal1.1***	Z6neal	F2	GH	*voc1.2b*	1	31,791,378	10.62	25.59	29,640,551	6.77	32,509,306	7.33	4.76	-3.23
248	***Z6neal2.1***	Z6neal	F3	H.R2	*voc2.1b*	2	3,597,964	4.81	15.57	1,893,203	3.12	11,209,309	3.04	3.99	-1.65
249	***Z6neal2.2***	Z6neal	F3	GH	*voc2.1b*	2	6,741,335	4.40	15.41	1,957,580	2.63	11,291,671	2.82	4.28	-1.01
250	***Z6neal2.3***	Z6neal	RIL	GH.R1	*voc2.2*	2	10,099,499	5.17	11.86	7,998,780	0.48	14,337,244	2.39	2.25	
251	***Z6neal2.4***	Z6neal	RIL	GH.R2	*voc2.2*	2	10,099,499	12.50	24.60	7,998,780	0.35	11,255,780	5.12	1.72	
252	***Z6neal2.5***	Z6neal	F3	H.R2	*voc2.2*	2	10,238,310	14.17	30.62	9,699,386	11.21	11,201,384	11.19	5.45	-1.93
253	***Z6neal3.1***	Z6neal	F3	H.R2	*voc3.2a*	3	37,584,346	4.43	12.44	28,811,806	2.67	40,103,997	3.19	-4.57	-0.67
254	***Z6neal4.1***	Z6neal	RIL	H.R2		4	2,790,441	11.81	32.75	1,832,529	3.66	3,104,887	10.79	3.69	
255	***Z6neal4.2***	Z6neal	RIL	GH.R1		4	7,591,076	6.47	15.19	5,842,482	4.32	14,126,117	4.15	5.18	
256	***Z6neal4.3***	Z6neal	RIL	H.R2	*voc4.2b*	4	17,586,005	4.18	6.63	15,211,958	2.36	23,028,001	1.21	1.33	
257	***Z6neal4.4***	Z6neal	RIL	H.R1	*voc4.3a*	4	24,891,253	18.37	38.12	24,473,476	12.31	24,891,253	12.35	-3.76	
258	***Z6neal5.1***	Z6neal	RIL	H.R2		5	7,359,200	10.70	29.08	6,903,854	9.02	11,523,346	4.62	5.33	
259	***Z6neal5.2***	Z6neal	F3	H.R2	*voc5.1a*	5	21,841,655	15.69	34.91	21,840,436	13.22	24,169,934	12.78	1.70	-3.40
260	***Z6neal5.3***	Z6neal	RIL	GH.R1		5	27,100,000	6.07	14.15	25,936,285	3.88	29,613,496	5.20	-5.37	
261	***Z6neal7.1***	Z6neal	F2	GH	*voc7.3a*	7	19,023,724	7.49	17.22	18,668,087	5.57	19,560,215	0.62	-1.96	-3.84
262	***Z6neal7.2***	Z6neal	F3	H.R2		7	20,953,907	9.08	17.82	21,266,167	6.98	20,925,778	6.96	-0.62	-1.12
263	***Z6neol1.1***	Z6neol	F2	GH	*voc1.1a*	1	1,450,884	5.24	16.57	148,977	4.19	7,273,355	3.34	-5.03	-0.65
264	***Z6neol1.2***	Z6neol	F3	GH	*voc1.1a*	1	1,450,884	5.10	15.78	148,977	3.89	7,273,355	3.30	-4.93	-0.85
265	***Z6neol1.3***	Z6neol	F3	H.R1		1	22,636,956	9.63	28.02	20,992,511	7.65	23,254,646	7.20	2.22	-0.63
266	***Z6neol1.4***	Z6neol	F2	GH	*voc1.2b*	1	32,506,255	4.20	11.18	27,750,202	2.69	32,509,306	3.22	4.26	-2.19
267	***Z6neol2.1***	Z6neol	RIL	GH.R2	*voc2.1a*	2	3,396,451	33.12	41.70	2,710,577	33.09	7,209,657	31.76	-10.53	
268	***Z6neol2.2***	Z6neol	RIL	GH.R1	*voc2.1a*	2	4,270,582	20.19	47.54	3,674,089	18.57	5,789,934	16.23	-7.68	
269	***Z6neol2.3***	Z6neol	RIL	H.R1	*voc2.1a*	2	4,371,493	14.26	32.96	3,396,451	12.52	4,371,493	12.83	-8.41	
270	***Z6neol2.4***	Z6neol	RIL	H.R2	*voc2.1a*	2	4,371,493	20.93	53.69	3,396,451	19.71	4,824,086	19.89	-9.86	
271	***Z6neol2.5***	Z6neol	RIL	GH.R2		2	7,209,657	21.73	21.33	7,209,657	21.73	7,998,780	18.97	2.25	
272	***Z6neol3.1***	Z6neol	RIL	GH.R2	*voc3.1a*	3	13,879,294	35.48	47.13	13,879,294	32.21	14,831,952	24.68	-0.67	
273	***Z6neol3.2***	Z6neol	F3	H.R1	*voc3.1a*	3	14,526,460	9.88	28.95	13,877,026	7.56	15,621,241	6.56	-1.56	2.19
274	***Z6neol4.1***	Z6neol	RIL	GH.R2		4	2,762,508	20.59	19.72	1,832,529	9.15	3,295,707	19.33	-9.05	
275	***Z6neol4.2***	Z6neol	F2	GH	*voc4.1*	4	5,274,514	4.09	12.66	2,142,457	2.02	16,510,766	2.23	4.37	-0.40
276	***Z6neol4.3***	Z6neol	F3	GH	*voc4.1*	4	5,274,514	4.90	15.12	2,764,669	3.22	8,728,554	2.17	4.77	-0.70
277	***Z6neol5.1***	Z6neol	F3	H.R1		5	4,554,819	3.90	9.88	119,993	2.19	6,403,064	1.82	4.18	0.14
278	***Z6neol5.2***	Z6neol	RIL	H.R1		5	11,523,346	4.68	8.96	7,359,200	2.24	12,327,989	2.86	3.43	
279	***Z6neol5.3***	Z6neol	RIL	H.R1		5	18,251,176	4.89	9.40	12,919,926	2.18	19,773,641	1.47	-2.35	
280	***Z6neol6.1***	Z6neol	RIL	H.R2		6	6,507,586	12.44	26.51	5,569,377	8.52	6,507,586	12.44	-0.68	
281	***Z6neol6.2***	Z6neol	F2	GH		6	9,359,366	4.95	12.33	7,562,939	1.33	9,566,200	2.15	3.95	3.61
282	***Z6neol6.3***	Z6neol	F2	GH		6	10,443,737	6.55	16.69	9,597,717	3.36	10,409,199	4.60	-4.17	-4.73
283	***Z6neol6.4***	Z6neol	RIL	GH.R1		6	11,058,959	11.43	22.78	11,058,959	11.43	11,866,660	11.07	-0.79	
284	***Z6neol7.1***	Z6neol	F3	H.R2	*voc7.2*	7	8,849,234	11.57	24.34	8,849,234	3.26	9,817,231	8.81	6.78	-7.04

*A/D: Additive/Dominance effects; PVE: percentage of variance explained. All marker positions are from 9930v3.0. GH: greenhouse (WSGH); H: open field (HARS); R1/R2: replication 1/2

Table S9. Main statistics of QTL for 24 VOCs detected from three populations and two environments

#	VOCs	# QTL	F2	F3H	F3GH	RILH	RILGH
1	**E2heal**	**19**	2	4	2	5	6
2	**E2heol**	**2**	0	0	2	0	0
3	**E2neal**	**18**	4	3	3	4	4
4	**E3heal**	**8**	2	5	0	1	0
5	**E3heol**	**2**	0	2	0	0	0
6	**E6neal**	**6**	1	0	0	2	3
7	**EE24ndeal**	**10**	3	2	0	3	2
8	**EE26ndeal**	**5**	1	2	1	1	0
9	**EE36ndeol**	**5**	3	2	0	0	0
10	**EZ26ndeal**	**19**	2	6	4	3	4
11	**EZ26ndeol**	**19**	7	2	5	5	0
12	**EZ36ndeol**	**16**	2	6	4	1	3
13	**hal**	**21**	3	3	3	6	6
14	**hol**	**19**	5	4	4	2	4
15	**nal**	**9**	0	3	0	2	4
16	**neol**	**7**	2	2	0	0	3
17	**nol**	**15**	2	3	1	6	3
18	**Z2heal**	**14**	3	0	3	2	6
19	**Z2neal**	**4**	0	0	0	3	1
20	**Z3heal**	**4**	0	4	0	0	0
21	**Z3heol**	**11**	1	2	3	2	3
22	**Z3neol**	**13**	4	2	3	0	4
23	**Z6neal**	**16**	2	5	1	4	4
24	**Z6neol**	**22**	5	4	2	5	6
	**SUM**	**284**	54	66	41	57	66

Table S10. QTL for VOCs detected with principal components (PC) in WI7799 populations.

#	QTL	Trait	Environments or Replications	PC#	Populations	QTL cluster	Chr	Nearest marker	LOD score	PVE (%)	Left_border	Left LOD	Right_border	Right LOD	*A*	*D*
1	*PC3.RILHR1_1.1*	PC3_RILHR1	H.R1	PC3	RIL	voc1.1a, voc1.1b	1	62,710	4.32	10.20	62,710	4.32	821,561	2.52	3.86	
2	*PC5.F3GH_1.1*	PC5_F3GH	GH	PC5	F3	voc1.1a, voc1.1b	1	1,447,411	4.27	15.00	148,606	2.57	5,057,984	2.26	4.51	-0.66
3	*PC2.F3HR2_1.1*	PC2_F3HR2	H.R2	PC2	F3		1	15,517,113	9.32	25.95	10,999,522	6.84	15,982,265	6.76	2.02	1.58
4	*PC3.F2_1.1*	PC3_F2	GH	PC3	F2	voc1.2a, voc1.2b	1	30,123,683	3.78	12.09	25,069,200	1.82	32,509,306	2.60	4.19	-1.30
5	*PC3.RILGH_2.1*	PC3_RILGH	GH	PC3	RIL	voc2.1a, voc2.1b	2	4,270,582	8.35	24.63	2,710,577	7.67	5,789,934	6.10	-6.36	
6	*PC4.RILGH_2.1*	PC4_RILGH	GH	PC4	RIL	voc2.1a, voc2.1b	2	4,270,582	4.75	11.98	2,710,577	4.07	7,209,657	2.47	-4.61	
7	*TotalPC.RILGH_2.1*	TotalPC_RILGH	GH	TotalPC	RIL	voc2.1a, voc2.1b	2	4,270,582	13.05	35.72	3,674,089	10.22	5,789,934	9.85	8.50	
8	*PC2.RILHR1_2.1*	PC2_RILHR1	H.R1	PC2	RIL	voc2.1a, voc2.1b	2	4,371,493	6.78	15.92	2,710,577	6.21	4,650,059	5.28	-5.71	
9	*PC1.RILHR1_2.1*	PC1_RILHR1	H.R1	PC1	RIL	voc2.1a, voc2.1b	2	4,371,493	6.33	19.29	2,710,577	4.71	4,824,086	3.65	-5.45	
10	*PC3.RILHR2_2.1*	PC3_RILHR2	H.R2	PC3	RIL	voc2.1a, voc2.1b	2	4,371,493	7.79	23.05	3,396,451	5.91	4,824,086	5.94	-6.33	
11	*PC1.F3GH_2.1*	PC1_F3GH	GH	PC1	F3	voc2.1a, voc2.1b	2	4,371,493	4.81	10.47	4,024,313	3.30	6,473,847	2.81	-3.46	-3.72
12	*PC3.F3GH_2.1*	PC3_F3GH	GH	PC3	F3	voc2.1a, voc2.1b	2	4,571,069	4.76	16.56	535,955	3.96	7,015,092	2.55	-4.83	-1.26
13	*TotalPC.F3GH_2.1*	TotalPC_F3GH	GH	TotalPC	F3		2	6,302,782	7.46	21.51	3,918,910	4.73	7,015,092	5.72	6.13	1.82
14	*PC5.F2_2.1*	PC5_F2	GH	PC5	F2	voc2.2	2	7,706,537	11.97	32.73	6,741,404	10.32	6,314,958	6.63	3.79	4.35
15	*PC2.RILGH_2.1*	PC2_RILGH	GH	PC2	RIL	voc2.2	2	10,099,499	3.97	8.46	2,710,577	2.66	11,255,780	0.85	-0.17	
16	*PC3.RILHR1_2.1*	PC3_RILHR1	H.R1	PC3	RIL		2	13,272,902	5.98	14.55	12,538,952	4.29	14,337,244	3.64	3.88	
17	*TotalPC.F2_2.1*	TotalPC_F2	GH	TotalPC	F2		2	13,532,387	7.55	22.19	10,271,021	4.71	14,432,019	5.70	0.06	-0.20
18	*TotalPC.F2_2.2*	TotalPC_F2	GH	TotalPC	F2		2	14,360,862	7.24	21.18	14,363,128	2.16	14,445,339	5.60	3.25	1.44
19	*PC3.RILHR1_2.2*	PC3_RILHR1	H.R1	PC3	RIL		2	23,910,448	6.73	16.58	22,728,323	3.36	23,910,448	6.73	-2.82	
20	*PC5.F2_3.1*	PC5_F2	GH	PC5	F2		3	2,170,269	7.49	18.90	347,188	6.18	3,343,817	4.62	1.67	2.91
21	*PC2.RILHR1_3.1*	PC2_RILHR1	H.R1	PC2	RIL	voc3.1a, voc3.1b	3	12,914,770	5.79	13.34	11,768,986	3.86	16,270,109	2.63	5.05	
22	*PC3.F3HR1_3.1*	PC3_F3HR1	H.R1	PC3	F3	voc3.1a, voc3.1b	3	12,994,572	4.92	15.65	3,846,735	3.30	21,975,345	2.80	-4.69	1.24
23	*PC4.RILHR1_3.1*	PC4_RILHR1	H.R1	PC4	RIL	voc3.1a, voc3.1b	3	13,879,294	6.35	15.36	12,914,770	4.23	16,270,109	3.65	-4.74	
24	*PC4.F3HR2_3.1*	PC4_F3HR2	H.R2	PC4	F3	voc3.1a, voc3.1b	3	14,810,570	7.70	23.70	12,948,476	5.53	15,621,241	4.86	-6.30	-0.66
25	*PC4.RILHR2_3.1*	PC4_RILHR2	H.R2	PC4	RIL	voc3.1a, voc3.1b	3	14,831,952	5.88	13.48	13,737,934	4.15	21,103,235	4.34	-5.31	
26	*PC2.F3GH_3.1*	PC2_F3GH	GH	PC2	F3	voc3.1a, voc3.1b	3	14,870,211	5.02	12.23	13,877,026	2.76	37,085,428	3.36	-4.67	1.50
27	*PC2.F2_3.1*	PC2_F2	GH	PC2	F2	voc3.1a, voc3.1b	3	16,494,950	4.75	14.97	13,810,691	3.04	23,691,056	2.51	-4.72	0.69
28	*PC1.F3GH_3.1*	PC1_F3GH	GH	PC1	F3		3	19,530,148	8.26	19.27	347,188	6.77	20,247,363	4.84	2.76	0.20
29	*PC2.RILGH_3.1*	PC2_RILGH	GH	PC2	RIL		3	23,570,938	5.08	11.02	14,831,952	3.41	27,059,020	2.00	-4.71	
30	*TotalPC.F3HR2_3.1*	TotalPC_F3HR2	H.R2	TotalPC	F3	voc3.2a, voc3.2b	3	30,033,109	3.26	10.84	29,454,353	1.31	33,363,900	1.55	-0.18	3.93
31	*PC5.RILHR1_3.1*	PC5_RILHR1	H.R1	PC5	RIL	voc3.2a, voc3.2b	3	33,358,789	3.66	10.71	28,807,221	1.78	40,306,100	3.00	4.11	
32	*PC4.RILHR2_3.2*	PC4_RILHR2	H.R2	PC4	RIL	voc3.2a, voc3.2b	3	39,443,369	4.03	8.93	35,638,234	2.31	40,306,100	3.74	-4.03	
33	*TotalPC.F3GH_3.1*	TotalPC_F3GH	GH	TotalPC	F3	voc3.2a, voc3.2b	3	39,583,702	6.69	18.99	37,637,669	4.53	40,103,997	5.53	-5.41	2.16
34	*PC1.F3GH_4.1*	PC1_F3GH	GH	PC1	F3	voc4.1	4	5,274,514	5.77	12.81	4,666,652	3.78	14,904,144	3.77	5.07	-0.83
35	*PC2.RILGH_4.1*	PC2_RILGH	GH	PC2	RIL		4	7,591,076	4.87	10.54	4,117,975	2.63	16,397,147	3.34	-4.54	
36	*PC4.RILHR2_4.1*	PC4_RILHR2	H.R2	PC4	RIL		4	14,108,878	3.92	8.67	7,591,076	2.00	15,211,958	2.28	-3.24	
37	*PC2.F3GH_4.1*	PC2_F3GH	GH	PC2	F3		4	14,442,351	9.02	23.79	13,743,498	6.07	14,641,217	7.31	2.19	0.67
38	*TotalPC.RILHR2_4.1*	TotalPC_RILHR2	H.R2	TotalPC	RIL	voc4.3a, voc4.3b	4	24,397,821	5.28	16.26	23,317,980	2.66	24,397,821	3.69	2.79	
39	*PC1.RILHR2_4.1*	PC1_RILHR2	H.R2	PC1	RIL	voc4.3a, voc4.3b	4	24,397,821	6.16	18.70	23,317,980	3.78	24,891,253	4.28	4.04	
40	*PC2.F3HR2_5.1*	PC2_F3HR2	H.R2	PC2	F3		5	119,993	10.04	28.32	104,789	9.07	2,117,964	8.07	1.56	3.58
41	*PC2.RILHR1_5.1*	PC2_RILHR1	H.R1	PC2	RIL	voc5.1a, voc5.1b	5	20,883,856	3.98	8.90	17,894,389	2.04	22,259,813	2.26	-3.94	
42	*TotalPC.RILHR1_5.1*	TotalPC_RILHR1	H.R1	TotalPC	RIL	voc5.1a, voc5.1b	5	22,087,080	4.00	12.68	1,142,027	2.37	24,759,262	2.29	-4.27	
43	*PC2.RILGH_5.1*	PC2_RILGH	GH	PC2	RIL		5	24,759,262	5.24	11.41	22,638,163	3.27	29,498,144	3.62	4.68	
44	*PC5.RILHR1_5.1*	PC5_RILHR1	H.R1	PC5	RIL		5	30,134,952	2.91	8.40	23,064,539	1.04	31,257,896	2.03	-3.52	
45	*PC2.RILGH_6.1*	PC2_RILGH	GH	PC2	RIL	voc6.1a, voc6.1b	6	20,957,428	5.28	11.50	15,869,056	1.99	21,726,330	2.49	-4.88	
46	*PC1.RILGH_6.1*	PC1_RILGH	GH	PC1	RIL	voc6.1a, voc6.1b	6	20,957,428	3.24	9.45	15,869,056	0.69	22,058,543	1.51	3.60	
47	*PC1.F3GH_7.1*	PC1_F3GH	GH	PC1	F3	voc7.1a, voc7.1b	7	735,605	7.46	17.11	344,231	4.63	1,017,210	4.97	2.73	-1.35
48	*PC4.RILGH_7.1*	PC4_RILGH	GH	PC4	RIL	voc7.1a., voc7.1b	7	874,962	3.86	9.60	874,962	3.56	4,836,123	1.74	-3.08	
49	*PC4.F3GH_7.1*	PC4_F3GH	GH	PC4	F3	voc7.2	7	4,840,086	6.22	19.56	4,719,834	5.27	11,873,378	4.45	3.59	1.97
50	*PC1.RILGH_7.1*	PC1_RILGH	GH	PC1	RIL	voc7.2	7	7,781,806	3.31	9.66	4,836,123	1.80	12,890,052	1.75	-3.74	
51	*PC4.F3GH_7.2*	PC4_F3GH	GH	PC4	F3		7	11,873,378	7.78	25.22	10,366,570	2.12	11,916,371	2.86	-0.93	1.58
52	*PC2.F3GH_7.1*	PC2_F3GH	GH	PC2	F3	voc7.3a, voc7.3b	7	19,560,215	8.58	22.43	18,800,910	6.81	20,922,872	5.76	2.41	0.72

*A/D: Additive/Dominance effects; PVE: percentage of variance explained. All marker positions are from 9930v3.0. GH: greenhouse (WSGH); H: open field (HARS); R1/R2: replication 1/2

Table S11. Annotated genes in voc2.1 and voc3.1 QTL cluster regions in Gy14v2.1 and 9930v3.0 genomes and expression level from two previous studies*

#	QTL cluster	Gene_ID (Gy14v2.1) and annotation	Gene_ID (9930v3.0) and annotation	Normalized FPKM_SRA071224			Normalized FPKM_GSE116581			
				FL_7**	FL_14	FL_21	FL_10	FL_20	FL_30	FL_40
1	VOC2.1	CsGy2G004600.1: GUB_WAK_bind domain-containing protein	CsaV3_2G006830: wall-associated receptor kinase-like 20	0	0.16	0	0.23	0	0	0
2	VOC2.1	CsGy2G004610.2: Pre-mRNA-splicing factor 38	CsaV3_2G006840: Pre-mRNA-splicing factor 38A	45.44	61.41	66.85	44.14	43.27	56.2	43.57
3	VOC2.1	CsGy2G004630.2: t-SNARE coiled-coil homology domain-containing	CsaV3_2G006850: Syntaxin, putative	12.64	11.49	13.45	17.55	22.88	11.59	9.1
4	VOC2.1	CsGy2G004640.2: Importin subunit alpha	CsaV3_2G006860: Importin subunit alpha	204.78	301.12	265.99	156.96	244.9	395.5	217.7
5	VOC2.1	CsGy2G004650.2: J domain-containing protein	CsaV3_2G006870: chaperone protein DnaJ-like	233.4	226.51	151.95	124.04	246.13	282.01	129.68
6	VOC2.1	no match	CsaV3_2G006880: Unknown protein	7.44	3.42	7.55	7.04	11.76	6.05	4.76
7	VOC2.1	CsGy2G004660.2: Calmodulin binding protein-like protein	CsaV3_2G006890: Calmodulin binding protein-like protein	7.41	4.21	6.65	10.21	16.15	9.89	8.78
8	VOC2.1	CsGy2G004670.2: ATP-dependent DNA helicase	CsaV3_2G006900: ATP-dependent DNA helicase	0.78	0.27	0.22	1.04	0.56	0.35	0.57
9	VOC2.1	CsGy2G004680.2: GPI-anchored adhesin-like protein	CsaV3_2G006910: DUF4378 domain protein	10.1	9.72	8.01	10.19	8.46	3.44	3.42
10	VOC2.1	no match	CsaV3_2G006920: Unknown protein	3.37	1.86	2.34	0	0.32	0.32	1.73
11	VOC2.1	CsGy2G004690.2: Stress response NST1-like protein	CsaV3_2G006930: Calmodulin-binding protein	12.29	4.14	2.49	15.33	14.73	1.43	0.23
12	VOC2.1	CsGy2G004710.2: U1-type domain-containing protein	CsaV3_2G006940: zinc finger matrin-type protein 2	102.7	177.29	176.93	108.81	151.05	224.75	136.51
13	VOC2.1	no match	CsaV3_2G006950: Unknown protein	22.69	32.53	27.01	16.99	26.55	37.95	0
14	VOC2.1	CsGy2G004720.2: Pentatricopeptide repeat	CsaV3_2G006960: Pentatricopeptide repeat	3.98	2.72	1.58	2.73	1.54	1.31	1.31
15	VOC2.1	CsGy2G004730.2: GTP-binding protein SAR1A	CsaV3_2G006970: GTP-binding protein SAR1A	93.07	74.17	69.69	143.6	97.72	39.97	51.2
16	VOC2.1	CsGy2G004740.2: Usp domain-containing protein	CsaV3_2G006980: Universal stress protein A-like protein	9.19	13.6	11.45	13.22	24.73	23.91	36.32
17	VOC2.1	CsGy2G004750.2: Calreticulin	CsaV3_2G006990: calreticulin	569.35	403.86	207.45	437.06	329.66	128.91	270.08
18	VOC2.1	CsGy2G004760.2: H(+)-exporting diphosphatase	CsaV3_2G007000: Pyrophosphate-energized vacuolar membrane proton pump, putative	2.4	2.43	1.9	0.38	1.4	1.44	5.04
19	VOC2.1	CsGy2G004770.2: PseudoU_synth_2 domain-containing protein	CsaV3_2G007010: Pseudouridine synthase	9.05	5.58	5.22	9.45	7.29	5.08	6.56
20	VOC2.1	CsGy2G004780.1: Antigenic heat-stable protein	CsaV3_2G007020: Peptide chain release factor	33.84	36.38	25.44	44.5	33.82	26.68	42.3
21	VOC2.1	CsGy2G004790.1: urease accessory protein F	CsaV3_2G007030: Urease accessory protein UreF	13.44	10.55	8.96	8.32	9.24	4.46	3.46
22	VOC2.1	CsGy2G004800.2: Lon protease homolog, mitochondrial	CsaV3_2G007040: Lon protease homolog, mitochondrial	13.02	10.82	9.44	17.04	15.86	5.92	7.75
23	VOC2.1	CsGy2G004820.2: Pentatricopeptide repeat-containing protein	CsaV3_2G007050: pentatricopeptide repeat-containing protein At1g09820	4.76	2.96	2.47	3.89	2.32	2.42	1.59
24	VOC2.1	CsGy2G004830.2: Nicotianamine synthase-like	CsaV3_2G007060: Mediator of RNA polymerase II transcription subunit like	19.78	17.92	20.42	17.24	20.35	18.62	18.21
25	VOC2.1	no match	CsaV3_2G007070: Zinc finger BED domain-containing protein DAYSLEEPER	9.32	8.54	7.89	11.33	9.22	10.35	11.91
26	VOC2.1	CsGy2G004840.1: nicotianamine synthase	CsaV3_2G007080: Nicotianamine synthase	2.24	1.99	1.33	3.44	0.56	1.28	0.81
27	VOC2.1	no match	CsaV3_2G007090: Nicotianamine synthase-like protein	0	0.1	0	0.13	0	0	0.28
28	VOC2.1	CsGy2G004860.2: histidine kinase CKI1	CsaV3_2G007100: Histidine kinase	0.16	0	0	0.45	0.14	0.03	0.04
29	VOC2.1	CsGy2G004870.2: Pentatricopeptide repeat-containing protein	CsaV3_2G007110: pentatricopeptide repeat-containing protein At3g62470, mitochondrial-like	7.55	7.77	6.22	7.6	6.01	3.88	5.52
30	VOC2.1	no match	CsaV3_2G007120: Homeobox protein knotted-1-like	0.13	0.1	0	1.88	0.67	0.08	0.45
31	VOC2.1	CsGy2G004880.2: Protein EFR3-like protein B	CsaV3_2G007130: protein EFR3 homolog B-like	27.27	12.43	7.46	19.09	13.19	5.42	7.12
32	VOC2.1	no match	CsaV3_2G007140: RNA polymerase II-associated 3	12.35	13.08	13.63	17.98	21.26	19.16	30.09
33	VOC2.1	CsGy2G004890.2: rab9 effector protein with kelch motifs-like	CsaV3_2G007150: Rab9 effector protein with Kelch	3.55	7.54	3.22	2.23	3.35	6.1	1.63
34	VOC2.1	CsGy2G004910.2: Pectinesterase	CsaV3_2G007170: Pectinesterase	0	0	0	0	0.23	0.22	0
35	VOC2.1	**CsGy2G004930.1: Transcription factor bHLH149-like protein**	**CsaV3_2G007180: transcription factor bHLH149-like**	9.64	14.02	9.79	1.94	8.07	1.27	3.55
36	VOC2.1	no match	CsaV3_2G007190: Unknown protein	30.1	23.65	12.5	22.41	30.9	18.09	13.26
37	VOC2.1	CsGy2G004950.1: Importin subunit alpha	CsaV3_2G007200: Importin subunit alpha	73.06	59.17	64.38	79.5	75.08	67.22	63.45
38	VOC2.1	CsGy2G005050.1: GDSL esterase/lipase	CsaV3_2G007270: Gdsl esterase/lipase	4.01	4.9	4.35	7.1	5.91	2.27	0.59
39	VOC2.1	CsGy2G005070.2: Glucose-6-phosphate 1-dehydrogenase	CsaV3_2G007280: Glucose-6-phosphate 1-dehydrogenase	8	6.69	6.92	21.29	16.72	10.42	13.01
40	VOC2.1	CsGy2G005090.2: Protein kinase domain-containing protein	CsaV3_2G007300: myosin-2 heavy chain, non muscle-like	5.71	15.86	10.77	8.07	9.27	11.03	11.88
41	VOC2.1	CsGy2G005120.1: Aminotran_5 domain-containing protein	CsaV3_2G007310: Isopenicillin N epimerase	43.68	38.1	36.43	43.66	34.49	34.81	39.75
42	VOC2.1	CsGy2G005200.2: protein NRT1/ PTR FAMILY 4.5-like	CsaV3_2G007400: protein NRT1/ PTR FAMILY 4	13.34	6.46	2.8	9.98	7.37	0.19	0.06
43	VOC2.1	CsGy2G005210.1: Germin-like protein	CsaV3_2G007410: Germin-like protein 1	103.81	54.77	51.52	126.3	110.08	70.24	6.62
44	VOC2.1	no match	CsaV3_2G007420: Unknown protein	1.94	1.61	6.98	7.7	6.31	16.44	7.43
45	VOC2.1	CsGy2G005220.2: Loricrin	CsaV3_2G007430: Protein FAM227A like	8.38	21.2	38.46	9.71	12.76	38.27	64.64
46	VOC2.1	CsGy2G005240.1: Ubiquitin carboxyl-terminal hydrolase	CsaV3_2G007460: Ubiquitin carboxyl-terminal hydrolase, putative	54.22	66.37	73.09	59.77	74.29	130.16	96.78
47	VOC2.1	CsGy2G005270.2: 26S proteasome non-ATPase regulatory subunit 9	CsaV3_2G007490: 26S proteasome non-ATPase regulatory subunit 9	28.87	33.4	48.98	16.76	22.92	42	51.2
48	VOC2.1	CsGy2G005290.2: CYTOSOL_AP domain-containing protein	CsaV3_2G007500: Leucine aminopeptidase	0.08	0.25	0.51	0.24	0.09	0.21	0.1
49	VOC2.1	no match	CsaV3_2G007510: Leucine aminopeptidase	0.23	0.06	0.26	0.75	0.05	0.04	0
50	VOC2.1	no match	CsaV3_2G007520: Leucine aminopeptidase	93.61	95.59	106.34	113.86	90.68	76.8	119.34
51	VOC2.1	CsGy2G005320.2: Cyclic nucleotide-binding domain-containing protein	CsaV3_2G007530: Cyclic nucleotide-gated ion channel, putative	48.34	67.5	92.07	75.34	88.67	127.82	99.56
52	VOC2.1	CsGy2G005370.2: zinc finger protein 830-like	CsaV3_2G007580: hepatoma-derived growth factor-related protein 2-like isoform X2	15.56	16.01	10.92	5.99	10.96	6.68	5.29
53	VOC2.1	CsGy2G005380.1: UPF0057 membrane protein	CsaV3_2G007590: UPF0057 membrane protein At4g30660	32.28	38.64	25.97	22.48	14.84	10.99	14.37
54	VOC2.1	CsGy2G005390.2: F-box protein SKIP1	CsaV3_2G007600: F-box protein SKIP1	19.97	21.69	19.17	24.94	29.84	20.04	13.16
55	VOC2.1	CsGy2G005400.2: protein MULTIPOLAR SPINDLE 1	CsaV3_2G007610: Protein multipolar spindle 1	19.46	22.25	18.97	13.62	29.93	19.57	1.05
56	VOC2.1	CsGy2G005420.2: zinc finger CCCH domain-containing protein 40-like	CsaV3_2G007640: Pre-mRNA-splicing factor RBM22	82.92	79.97	93.49	62.84	64.1	85.18	54.48
57	VOC2.1	CsGy2G005430.2: FAD_binding_3 domain-containing protein	CsaV3_2G007650: FAD/NAD(P)-binding oxidoreductase family protein	2.44	2.45	2.7	8.03	9.43	10.86	6.28
58	VOC2.1	CsGy2G005440.2: Ubiquitin-like domain-containing protein	CsaV3_2G007660: Ubiquitin-like protein	152.59	144.49	163.3	192.61	233.91	159.22	146.08
59	VOC2.1	CsGy2G005450.1: DNA-directed RNA polymerase	CsaV3_2G007670: DNA-directed RNA polymerase	5.48	6.25	4.89	9.06	11.07	7.87	6.21
60	VOC2.1	CsGy2G005460.2: Carboxypeptidase	CsaV3_2G007680: Carboxypeptidase	143.03	148.76	173.31	94.62	103.31	194.92	155.28
61	VOC2.1	CsGy2G005470.2: ATG11 domain-containing protein	CsaV3_2G007690: autophagy-related protein 11	16.79	16.29	18.27	11.29	8.89	12.91	29.63
62	VOC2.1	CsGy2G005480.2: 50S ribosomal protein L35 isoform 1	CsaV3_2G007700: ATP-dependent DNA helicase	24.91	24.42	34.32	18.48	20.77	21.38	22.41
63	VOC2.1	no match	CsaV3_2G007720: DUF594 family protein	3.59	0.69	0.3	3.38	1.04	0.07	0.05
64	VOC2.1	CsGy2G005500.1: GDP-L-fucose synthase	CsaV3_2G007730: GDP-L-fucose synthase	179.81	188.18	161.44	149.13	199.15	171.42	50.62
65	VOC2.1	CsGy2G005510.1: nuclear pore complex protein NUP43	CsaV3_2G007740: nuclear pore complex protein NUP43	18.8	14.62	9.29	17.7	17.56	9.25	7.67
66	VOC2.1	CsGy2G005520.1: S-adenosylmethionine decarboxylase proenzyme	CsaV3_2G007750: S-adenosylmethionine decarboxylase proenzyme	52.52	1873.7	1269.64	152	424.5	1737.4	585.78
67	VOC2.1	no match	CsaV3_2G007760: S-adenosylmethionine decarboxylase proenzyme-like	23.03	1089.5	594.89	147.34	173.09	813.32	653.63
68	VOC2.1	CsGy2G005530.2: Autophagy-related protein 18b isoform X1	CsaV3_2G007770: Autophagy-related 18	5.24	5.61	5.89	6.07	9.88	10.4	12.61
69	VOC2.1	CsGy2G005540.2: transmembrane protein 208 homolog	CsaV3_2G007780: transmembrane protein 208 homolog	26.29	37.01	34.79	40.27	37.22	30.63	56.55

70	VOC2.1	CsGy2G005550.2: TPR_REGION domain-containing protein	CsaV3_2G007790: tetratricopeptide repeat protein 1	41.25	54.51	65.48	41.36	42.51	33.47	127.75
71	VOC2.1	CsGy2G005560.2: 5'-deoxynucleotidase	CsaV3_2G007800: HD domain-containing protein 2	28.71	19.65	17.78	32.77	20.88	14.09	20.16
72	VOC2.1	CsGy2G005570.2: NAD(P)-bd_dom domain-containing protein	CsaV3_2G007810: UDP-glucuronate 4-epimerase	78.03	70.52	114.08	43.17	57.59	114.34	39.78
73	VOC2.1	CsGy2G005590.1: protein FLX-like 4	CsaV3_2G007820: protein FLX-like 4	22.52	20.28	21.52	26.69	25.94	16.96	7.66
74	VOC2.1	CsGy2G005600.2: Cytochrome c oxidase subunit	CsaV3_2G007830: Cytochrome c oxidase subunit	365.38	288.98	194.45	283.2	280.87	164.15	187.89
75	VOC2.1	CsGy2G005610.1: tetraspanin-8	CsaV3_2G007840: Tetraspanin	127.89	58.39	38.49	75.66	33.34	16.95	45.8
76	VOC2.1	CsGy2G005630.2: WAT1-related protein	CsaV3_2G007860: WAT1-related protein	693.56	417.82	1066.32	1624.3	1378.6	790.63	110.5
77	VOC2.1	CsGy2G005670.1: Transcription factor IBH1	CsaV3_2G007890: Transcription factor like	57.54	132.09	141.7	46.32	72.28	114.57	422.38
78	VOC2.1	CsGy2G005690.2: E3 ubiquitin-protein ligase XBAT32	CsaV3_2G007900: E3 ubiquitin-protein ligase XBAT33	32.01	64.85	69.56	17.68	33.67	43.12	34.62
79	VOC2.1	CsGy2G005700.1: RING-type domain-containing protein	CsaV3_2G007910: RING-H2 finger protein ATL13	0.47	0.94	0.47	0.12	0	0	0.13
80	VOC2.1	CsGy2G005710.1: RING-type domain-containing protein	CsaV3_2G007920: RING-H2 finger protein ATL56	18.81	21.18	13.25	6.72	18.74	12.04	5.45
81	VOC2.1	CsGy2G005720.1: AAA domain-containing protein	CsaV3_2G007930: Aaa-atpase	0.18	2.22	0.37	0	0	0	0
82	VOC2.1	CsGy2G005750.2: CASP-like protein	CsaV3_2G007950: CASP-like protein	0.21	0.71	1.25	0.49	0	0.74	0.18
83	VOC2.1	CsGy2G005790.2: Xyloglucan endotransglucosylase/hydrolase	CsaV3_2G007980: Xyloglucan endotransglucosylase/hydrolase	0	10	4.96	0.17	0	0	0
84	VOC2.1	CsGy2G005800.1: UBA domain-containing protein	CsaV3_2G007990: UBX domain-containing protein 1-like isoform X1	1.68	0.85	0.43	2.54	1.56	1.3	1.74
85	VOC2.1	CsGy2G005810.1: FGGY carbohydrate kinase domain-containing protein	CsaV3_2G008000: Carbohydrate kinase, FGGY-related	7.78	27.86	45.02	8.01	19.54	79.29	32.45
86	VOC2.1	CsGy2G005820.2: 26S proteasome regulatory subunit 4 homolog A	CsaV3_2G008010: 26S proteasome regulatory subunit 4 homolog	155.96	127.13	132.79	188.35	132.16	102.94	108.41
87	VOC2.1	no match	CsaV3_2G008020: Kinase family protein	27.6	32.05	53.9	44.44	66.88	77.81	55.03
88	VOC2.1	**CsGy2G005840.2: MYB transcription factor 36 (AT5G57620,**	**CsaV3_2G008030: MYB transcription factor**	10.94	2.04	0.8	1.48	0.32	0.33	0.38
89	VOC2.1	no match	CsaV3_2G008060: Protein DETOXIFICATION	0.42	0.35	0.07	0	0.04	0	0
90	VOC2.1	CsGy2G005880.1: Xylose isomerase	CsaV3_2G008080: Xylose isomerase	204.19	109.63	79.17	136.79	111.08	97.71	120.92
91	VOC2.1	CsGy2G005890.1: ADP-ribosylation factor 3	CsaV3_2G008090: ADP-ribosylation factor 1	54.86	62.68	57.15	104.36	68.6	47.55	68.58
92	VOC2.1	CsGy2G005900.2: Unknown protein	CsaV3_2G008100: (R)-mandelonitrile lyase 1-like	0	0.12	0	0.08	0.04	0	0
93	VOC2.1	CsGy2G005970.2: (R)-mandelonitrile lyase 1-like	CsaV3_2G008130: (R)-mandelonitrile lyase 1-like	0.3	0.12	0.25	0.15	0	0	0
94	VOC2.1	no match	CsaV3_2G008170: (R)-mandelonitrile lyase 1-like	0	0.31	0.87	0	0.05	0	0.07
95	VOC2.1	no match	CsaV3_2G008180: Unknown protein	0.9	0.25	0	0.32	0.95	1.25	0.26
96	VOC2.1	no match	CsaV3_2G008190: (R)-mandelonitrile lyase 1-like	0.3	0.18	0.25	0.4	0	0	0
97	VOC2.1	CsGy2G006010.1: zinc finger protein CONSTANS-LIKE 5-like	CsaV3_2G008220: zinc finger protein CONSTANS-LIKE 5-like	18.72	12.05	22.07	17.02	22.27	11.75	0.21
98	VOC2.1	CsGy2G006050.2: diacylglycerol kinase	CsaV3_2G008260: Diacylglycerol kinase	35.4	25.6	27.77	51.47	45.67	22.2	10.6
99	VOC2.1	CsGy2G006060.2: Chaperone protein dnaJ	CsaV3_2G008280: Chaperone DnaJ	2.55	1	27.79	6.92	12.39	12.2	13.5
100	VOC2.1	CsGy2G006070.1: DUF1685 family protein	CsaV3_2G008290: DUF1685 family protein	0.67	0.19	0.19	0.28	0	0.28	0
101	VOC2.1	CsGy2G006080.1: Clp R domain-containing protein	CsaV3_2G008310: chaperone protein ClpB	41.21	45.55	40.13	29.59	41.85	40.3	45.39
102	VOC2.1	CsGy2G006090.2: Chlorophyll A-B binding protein	CsaV3_2G008330: Chlorophyll A-B binding protein	97.69	68.09	51.95	99.79	164.86	118.89	72.41
103	VOC2.1	CsGy2G006100.2: SET domain-containing protein	CsaV3_2G008340: Carboxylase-lysine N-methyltransferase	5.2	4.43	2.64	9.13	6.45	4.27	5.14
104	VOC2.1	no match	CsaV3_2G008350: Unknown protein	0.5	0	0	0	0.84	0	0
105	VOC2.1	CsGy2G006110.2: Syntaxin-6_N domain-containing protein	CsaV3_2G008360: Syntaxin/t-SNARE family protein	28.45	13.19	12.39	54.52	30.37	12.89	11.95
106	VOC2.1	CsGy2G006120.1: RECA_2 domain-containing protein	CsaV3_2G008370: DNA repair protein XRCC3 homolog	2.06	1.71	0.8	0.76	0.57	0.81	0.47
107	VOC2.1	CsGy2G006130.2: Auxin-responsive protein	CsaV3_2G008380: Auxin-responsive protein	0	0.23	0.23	0	0.18	0.15	0
108	VOC2.1	no match	CsaV3_2G008400: Unknown protein	0	0.42	0.42	0	0	0.28	0
109	VOC2.1	CsGy2G006150.2: NADPH--cytochrome P450 reductase	CsaV3_2G008410: NADPH--cytochrome P450 reductase	69.94	106.44	132.38	72.49	150	305.74	149.56
110	VOC2.1	CsGy2G006160.2: Afadin-and alpha-actinin-binding protein isoform 1	CsaV3_2G008420: afadin- and alpha-actinin-binding protein	22.61	15.77	12.75	32.73	25.63	9.37	10.87
111	VOC2.1	CsGy2G006170.1: OSBP(Oxysterol binding protein)-related protein 4B,	CsaV3_2G008430: OSBP(Oxysterol binding protein)-related protein 4B, putative	0	0.17	0	0	0.12	0.47	0
112	VOC2.1	CsGy2G006180.1: 40S ribosomal protein S8	CsaV3_2G008440: 40S ribosomal protein S8	807.43	737.77	647.37	787.33	1109.4	1017.1	423.83
113	VOC2.1	CsGy2G006190.2: Fibronectin type-III domain-containing protein	CsaV3_2G008450: VIN3-like protein 2	17.62	11.18	8.33	23.5	23.09	10.2	6.08
114	VOC2.1	CsGy2G006210.2: PAS domain-containing protein	CsaV3_2G008460: Adagio protein 1	46.55	57.17	52.48	46.52	54.57	62.48	61.55
115	VOC2.1	CsGy2G006270.2: 1-acyl-sn-glycerol-3-phosphate acyltransferase	CsaV3_2G008470: 1-acyl-sn-glycerol-3-phosphate acyltransferase	154.01	38.07	40.88	27.13	14.91	17.18	18.99
116	VOC2.1	CsGy2G006280.1: DUF616 protein	CsaV3_2G008480: Plant/F20M13-60 protein	23.21	28.75	26.49	32.9	29.95	27.62	29.38
117	VOC2.1	CsGy2G006300.1: Pentatricopeptide repeat-containing protein	CsaV3_2G008490: Pentatricopeptide repeat protein	9.05	6.83	5.41	7.93	6.9	5.38	9.38
118	VOC2.1	CsGy2G006310.2: Homogentisate phytyltransferase 1, chloroplastic	CsaV3_2G008500: Homogentisate phytyltransferase	18.07	16.28	12.47	49.45	34.99	20.16	71.58
119	VOC3.1	CsGy3G017110.1: Cleavage and polyadenylation specificity factor subunit	CsaV3_3G017220: Cleavage and polyadenylation specificity factor subunit 2	13.97	11.76	12.56	19.79	19.54	17.38	11.84
120	VOC3.1	CsGy3G017120.2: SLH domain-containing protein	CsaV3_3G017230: Unknown protein	28	29.83	34.51	51.19	54.66	55.97	58.04
121	VOC3.1	no match	CsaV3_3G017240: 60S ribosomal protein L36	1056.2	1131.7	828.31	977.02	928.12	560.73	509.16
122	VOC3.1	CsGy3G017130.2: U-box domain-containing protein 32	CsaV3_3G017250: U-box domain-containing protein 32-like	3.66	4.89	8.29	4.3	5	6.83	11.94
123	VOC3.1	no match	CsaV3_3G017260: C2H2-type domain-containing protein	15.26	18.78	22.09	23.17	21.01	18.58	24.69
124	VOC3.1	CsGy3G017150.2: Protein kinase domain-containing protein	CsaV3_3G017270: Mitogen-activated protein kinase kinase kinase YODA-like	16.92	12.49	13.87	21.81	22.96	19.34	9.29
125	VOC3.1	CsGy3G017160.2: CitMHS domain-containing protein	CsaV3_3G017280: silicon efflux transporter LSI2	71.58	23.26	34.98	89.4	73.02	20.12	21.66
126	VOC3.1	no match	CsaV3_3G017290: Unknown protein	25.24	8.68	17.8	46.21	34.2	13.06	11.62
127	VOC3.1	CsGy3G017170.2: Hydroxyproline-rich glycoprotein family protein	CsaV3_3G017300: Hydroxyproline-rich glycoprotein family protein	49.91	46.54	35.39	36.73	41.02	23.55	20.46
128	VOC3.1	CsGy3G017180.2: sec-independent protein translocase protein TATB, chlo	CsaV3_3G017320: sec-independent protein translocase protein TATB, chloroplastic	22.24	18.07	16.81	39.26	37.32	19.45	19.1
129	VOC3.1	**CsGy3G017200.2: BAHD acyltransferase DCR**	**CsaV3_3G017330: BAHD acyltransferase DCR**	132.97	72.35	24.24	23.55	10.89	2.27	0.24
130	VOC3.1	CsGy3G017210.1: F-box domain-containing protein	CsaV3_3G017340: F-box domain-containing protein	5.64	2.88	4.78	9.47	4.77	4.63	5.51
131	VOC3.1	CsGy3G017220.1: C2 domain-containing protein	CsaV3_3G017350: C2 domain-containing protein	442.67	389.41	355.18	305.05	305.09	178.56	294.31
132	VOC3.1	CsGy3G017230.1: rRNA 2'-O-methyltransferase fibrillarin 2	CsaV3_3G017360: rRNA 2'-O-methyltransferase fibrillarin	78.57	76.67	66.44	56.64	85.37	54.16	44.47
133	VOC3.1	CsGy3G017240.2: protein PATRONUS 1-like	CsaV3_3G017370: protein PATRONUS 1-like	10.75	2.08	3.42	5.38	3.06	1.84	0.85
134	VOC3.1	CsGy3G017250.2: FAD-binding FR-type domain-containing protein	CsaV3_3G017380: ferric reduction oxidase 4-like	0.86	0.29	0.29	1.37	0.95	0.3	0.42
135	VOC3.1	CsGy3G017260.2: BnaA10g28370D protein	CsaV3_3G017390: Protein of unknown function (DUF819)	29.84	26.6	31.31	36.52	26.18	29.51	34.25
136	VOC3.1	CsGy3G017310.2: Beta-carotene hydroxylase	CsaV3_3G017450: Beta-carotene hydroxylase	19.39	95.88	180.47	27.69	33.2	405.55	2142.2
137	VOC3.1	CsGy3G017320.2: Protein kinase domain-containing protein	CsaV3_3G017460: Protein kinase domain-containing protein	0	0.1	0.58	0.14	0.07	0.54	0.66
138	VOC3.1	CsGy3G017350.2: HTH myb-type domain-containing protein	CsaV3_3G017490: protein REVEILLE 6-like isoform X2	52.82	46.79	56	10.75	13.79	32.52	54.7
139	VOC3.1	no match	CsaV3_3G017500: Unknown protein	45.63	10.77	15.9	6.41	36.12	1.18	71.18
140	VOC3.1	CsGy3G017360.1: DUF4477 domain-containing protein	CsaV3_3G017510: DUF4477 domain-containing protein	5.53	6.71	8.85	10.47	8.35	8.96	9.66
141	VOC3.1	CsGy3G017380.2: DNA-directed primase/polymerase protein	CsaV3_3G017530: DNA-directed primase/polymerase protein	0.67	0.17	0.22	0.78	0.51	0.19	0.12
142	VOC3.1	CsGy3G017390.1: delta(1)-pyrroline-2-carboxylate reductase	CsaV3_3G017540: delta(1)-pyrroline-2-carboxylate reductase	48.39	43.52	31.37	37.23	47.84	52.2	14.93

143	VOC3.1	CsGy3G017410.2: Pentatricopeptide repeat superfamily protein, putative i	CsaV3_3G017550: Pentatricopeptide repeat (PPR) superfamily protein	51.35	35.3	27.94	45.56	33.87	21.43	36.78
144	VOC3.1	no match	CsaV3_3G017560: Protein ENHANCED DOWNY MILDEW 2	0.37	0.61	0.46	0	0.23	0	1.71
145	VOC3.1	CsGy3G017440.1: NADH dehydrogenase (Ubiquinone) 1 alpha subcompl	CsaV3_3G017590: NADH dehydrogenase [ubiquinone] 1 alpha subcomplex subunit 5	258.22	177.43	164.57	250.21	232.03	178.12	171.68
146	VOC3.1	CsGy3G017450.2: Protein SGT1-like protein	CsaV3_3G017600: Protein SGT1-like protein	96	98.56	93.84	203.37	217.6	108.78	147.5
147	VOC3.1	CsGy3G017460.2: ABC transporter G family member 8	CsaV3_3G017610: ABC transporter-like	24.9	15.17	5.97	6.35	2.24	0.2	0.06
148	VOC3.1	no match	CsaV3_3G017620: Membrane-associated kinase regulator 5 isoform X2	8.94	4.55	3.93	5.38	3.04	1.15	8.66
149	VOC3.1	CsGy3G017490.2: Gamma-tubulin complex component	CsaV3_3G017640: Gamma-tubulin complex component	4.53	4.52	4.23	5.89	5.39	4.43	7.74
150	VOC3.1	CsGy3G017630.2: cAMP-regulated phosphoprotein 19-related protein	CsaV3_3G017750: cAMP-regulated phosphoprotein 19-related protein	267.94	123.37	82.65	285.66	269.2	53.99	48.86
151	VOC3.1	no match	CsaV3_3G017760: Unknown protein	30.26	15.59	17.66	81.52	72.06	19.43	11.46
152	VOC3.1	CsGy3G017660.2: Protein-lysine N-methyltransferase	CsaV3_3G017790: Protein-lysine N-methyltransferase E5676_scaffold451G00020	44.44	51.95	41.47	45.81	60.26	57.16	55.09
153	VOC3.1	CsGy3G017670.2: Pentatricopeptide repeat-containing protein	CsaV3_3G017800: Pentatricopeptide repeat-containing protein	4	4.8	6.78	7.28	7.33	10.16	5.79
154	VOC3.1	CsGy3G017680.2: homeobox-leucine zipper protein HDG11-like	CsaV3_3G017810: homeobox-leucine zipper protein HDG11-like	6.6	7.65	6.67	5.56	9.66	6.71	11.79
155	VOC3.1	CsGy3G017690.2: Nicastrin	CsaV3_3G017820: Nicastrin	19.27	22.76	21.51	23.21	15.91	16.25	33.34
156	VOC3.1	CsGy3G017720.2: TPR_REGION domain-containing protein	CsaV3_3G017830: Kinesin light chain 3 isoform X1	5.98	4.11	5.57	8.07	9.11	3.88	5.05
157	VOC3.1	no match	CsaV3_3G017840: 40S ribosomal protein S19	1.57	1.04	1.04	2.17	1.19	1.09	2.71
158	VOC3.1	CsGy3G017740.2: Pentatricopeptide repeat-containing protein	CsaV3_3G017850: Pentatricopeptide repeat	3.54	3.23	2.79	2.19	4.85	3.91	1.66
159	VOC3.1	no match	CsaV3_3G017870: aspartic proteinase PCS1	16.16	34.16	42.68	9.14	20.72	13.82	12.02
160	VOC3.1	CsGy3G017780.2: NADH dehydrogenase [ubiquinone] iron-sulfur protein	CsaV3_3G017880: NADH-ubiquinone dehydrogenase, mitochondrial, putative	111.14	84.53	83.85	116.63	87.56	77.3	69.94
161	VOC3.1	CsGy3G017790.2: Methionine aminopeptidase 2	CsaV3_3G017890: Methionine aminopeptidase 2	126.3	176.63	139.58	116.01	142.98	165.82	95.66
162	VOC3.1	no match	CsaV3_3G017950: Unknown protein	2.11	1.31	1.75	2.25	1.95	3.12	5.5
163	VOC3.1	CsGy3G017850.2: protein GAMETE EXPRESSED 1	CsaV3_3G017990: protein GAMETE EXPRESSED 1-like	0.44	0.18	0	0	0	0	0
164	VOC3.1	CsGy3G017870.2: ALA-interacting subunit	CsaV3_3G018000: ALA-interacting subunit	38.55	52.61	45.31	51.92	43.91	36.63	58.41
165	VOC3.1	CsGy3G017880.1: C2H2-type domain-containing protein	CsaV3_3G018010: zinc finger protein 4-like	1.35	3.85	5.48	1.23	0.7	3.99	1.14
166	VOC3.1	CsGy3G017890.2: Autophagy protein 5	CsaV3_3G018020: Autophagy protein 5	9.44	10.55	7.84	7.74	13.37	11.16	14.74
167	VOC3.1	CsGy3G017900.2: High chlorophyll fluorescence phenotype 173 protein	CsaV3_3G018030: Protein HIGH CHLOROPHYLL FLUORESCENCE PHENOTYPE 173, chlo	11.06	8.12	12.49	18.57	23.55	21.22	10.72
168	VOC3.1	CsGy3G017910.2: DUF547 domain-containing protein	CsaV3_3G018040: Protein of unknown function, DUF547	17.51	11.72	11.75	25.21	21.77	9.09	5.62
169	VOC3.1	CsGy3G017920.2: Mitochondrial import inner membrane translocase subu	CsaV3_3G018050: Unknown protein	114.15	41.73	36.76	44.28	77.17	41.5	14.52
170	VOC3.1	CsGy3G017930.2: MFS domain-containing protein	CsaV3_3G018060: organic cation/carnitine transporter 7	14.83	12.9	16.01	15.44	15.07	14.39	9.32
171	VOC3.1	CsGy3G017940.2: Kinetochore protein	CsaV3_3G018070: Unknown protein	5.21	3.4	4.33	8.6	9.3	5.51	1.71
172	VOC3.1	CsGy3G017950.2: Auxin-responsive GH3 family protein	CsaV3_3G018080: Auxin-responsive GH3 family protein	6.61	9.22	3.76	1.19	3.62	3.27	5.91
173	VOC3.1	CsGy3G017960.1: homeobox protein HAT3.1 isoform X2	CsaV3_3G018090: homeobox protein HAT3.1 isoform X2	3.86	3.59	4.4	6.17	3.94	6.31	5.81
174	VOC3.1	CsGy3G017970.2: homeobox protein HAT3.1	CsaV3_3G018100: homeobox protein HAT3.1-like	5.99	6.74	5.59	8.51	7.17	5.44	5.6
175	VOC3.1	no match	CsaV3_3G018110: zinc finger protein 7-like	0.39	3.74	3.92	1.33	2.6	4.75	1.22
176	VOC3.1	no match	CsaV3_3G018120: Unknown protein	1.7	2.12	2.48	2.73	2.88	5.16	6.33
177	VOC3.1	CsGy3G017980.2: Six-hairpin glycosidase-like protein	CsaV3_3G018130: Caspase-6 protein	97.92	85.44	79.53	71.01	85.99	54.55	30.8
178	VOC3.1	CsGy3G017990.2: Glyco_hyd_65N_2 domain-containing protein	CsaV3_3G018140: alpha-L-fucosidase 2	11.01	3.37	1.35	12.16	9.46	4.71	0.26
179	VOC3.1	no match	CsaV3_3G018150: GRF-type domain-containing protein	0	0.06	0.96	0.08	0	0.09	0.07
180	VOC3.1	CsGy3G018000.1: LYR motif-containing protein	CsaV3_3G018160: LYR motif-containing protein At3g19508	47.8	40.01	24.72	33.62	38.44	13.96	16.84
181	VOC3.1	CsGy3G018010.2: S-methyl-5-thioribose kinase	CsaV3_3G018170: S-methyl-5-thioribose kinase	51.4	54.84	38.93	70.09	52.13	40.86	30.68
182	VOC3.1	CsGy3G018020.1: cinnamyl alcohol dehydrogenase 5	CsaV3_3G018190: Cinnamyl alcohol dehydrogenase	61.01	141.85	127.21	121.3	171.71	96.38	41.5
183	VOC3.1	**CsGy3G018030.1: transcription factor MYB44-like**	**CsaV3_3G018200: transcription factor MYB44-like**	19.32	7.78	6.88	4.58	7.15	2.92	34.38
184	VOC3.1	CsGy3G018040.1: PRKR-interacting protein 1	CsaV3_3G018210: PRKR-interacting protein 1	57.33	56.23	63.18	59.59	51.9	48.69	48.95
185	VOC3.1	CsGy3G018050.1: Reticulon-like protein	CsaV3_3G018220: Reticulon-like protein	41.07	42.57	38.48	46.06	42.83	29.47	19.45
186	VOC3.1	CsGy3G018080.2: CitMHS domain-containing protein	CsaV3_3G018250: sodium/proton antiporter 1-like	168.45	147.15	140.89	163.06	178.72	141.94	76.7
187	VOC3.1	CsGy3G018090.1: Unknown protein	CsaV3_3G018260: Unknown protein	35.69	29.57	30.01	46.84	75.03	59.96	0
188	VOC3.1	CsGy3G018130.2: Actin-related protein 2/3 complex subunit 4	CsaV3_3G018320: Actin-related protein 2/3 complex subunit 4	59.8	61.6	65.06	133.39	116.98	159.57	124.05
189	VOC3.1	CsGy3G018140.2: Glycosyltransferase	CsaV3_3G018340: Glycosyltransferase	10.03	30.87	25.07	14.47	11.32	13.59	15.87
190	VOC3.1	CsGy3G018150.2: CBS domain-containing protein CBSX1, chloroplastic-	CsaV3_3G018360: CBS domain-containing protein CBSX1, chloroplastic-like	70.17	48.36	66.98	88.08	55.22	51.89	165.63
191	VOC3.1	no match	CsaV3_3G018410: Unknown protein	10.76	5.52	6.39	20.58	17.55	14.98	14.82
192	VOC3.1	no match	CsaV3_3G018420: Exocyst complex component	8.87	7.01	5.58	9.78	8.25	5.53	11.39
193	VOC3.1	CsGy3G018210.1: RING-type E3 ubiquitin transferase	CsaV3_3G018440: RING-type E3 ubiquitin transferase	7.6	19.05	18.39	4.21	10.07	17.93	19.54
194	VOC3.1	**CsGy3G018410.2: AP2/ERF domain-containing protein**	**CsaV3_3G018600: Ethylene-responsive transcription factor ERF**	136.17	118.68	80.47	2.02	114.14	3.66	19.23
195	VOC3.1	no match	CsaV3_3G018650: Unknown protein	6.92	6.18	8.41	2.56	7.36	12.59	0.45
196	VOC3.1	CsGy3G018460.1: TF-B3 domain-containing protein	CsaV3_3G018660: B3 domain-containing protein	8.13	10.76	13.3	15.48	15.78	22.71	18.68
197	VOC3.1	CsGy3G018470.2: Peptide methionine sulfoxide reductase msrA	CsaV3_3G018670: Protein of unknown function DUF829, transmembrane 53	28.44	31.66	36.75	28.09	30.05	43.97	31.52
198	VOC3.1	CsGy3G018480.1: Pinin_SDK_memA domain-containing protein	CsaV3_3G018690: Pinin_SDK_memA domain-containing protein	52.01	47.2	57.76	85.3	138.4	116.33	60.58
199	VOC3.1	CsGy3G018490.2: LEA_2 domain-containing protein	CsaV3_3G018700: LEA_2 domain-containing protein	12.78	16.72	15.42	6.5	11.09	13.54	11.47
200	VOC3.1	CsGy3G018500.2: Tubby-like F-box protein	CsaV3_3G018710: Tubby-like F-box protein	54.83	64.82	57.54	54.69	62.31	53.39	50.48
201	VOC3.1	CsGy3G018510.2: Unknown protein	CsaV3_3G018720: Tubby-like F-box protein	267.31	304.01	265.3	388.36	523.06	555.85	789.43
202	VOC3.1	no match	CsaV3_3G018750: Unknown protein	12.63	16.1	11.3	13.82	18.05	36.46	11.47
203	VOC3.1	no match	CsaV3_3G018760: Unknown protein	15.54	13.78	9.91	16.25	21.82	38.34	7.41
204	VOC3.1	no match	CsaV3_3G018770: Sphingoid long-chain bases kinase 2	11.6	13.46	9.64	20.71	16.94	22.02	9.39
205	VOC3.1	CsGy3G018560.2: Cytochrome b-c1 complex subunit 6	CsaV3_3G018780: cytochrome b-c1 complex subunit 6-like	103.23	94.2	103.41	179.18	128.41	87.58	91.16
206	VOC3.1	CsGy3G018580.2: kinetochore protein NDC80 homolog	CsaV3_3G018800: Kinetochore protein NDC80-like protein	8.3	7.05	5.47	7.19	6.23	3.75	5.29
207	VOC3.1	no match	CsaV3_3G018810: Unknown protein	5.78	6.92	2.67	1.37	5.94	3.45	0
208	VOC3.1	no match	CsaV3_3G018820: Unknown protein	1.25	3.11	2.43	5.63	6.99	2.79	5.15
209	VOC3.1	CsGy3G018610.2: Prolyl-tRNA synthetase	CsaV3_3G018830: Prolyl-tRNA synthetase	18.2	11.52	11.01	21.48	20.45	14.39	13.46
210	VOC3.1	CsGy3G018630.1: 6-phosphogluconate dehydrogenase	CsaV3_3G018840: 6-phosphogluconate dehydrogenase family protein	1.14	2.06	1.2	1.06	1.29	2.26	1.07
211	VOC3.1	no match	CsaV3_3G018850: Maternal effect embryo arrest 9	173.96	137.34	174.28	131.8	89.61	82.68	84.73
212	VOC3.1	CsGy3G018640.2: GOLD domain-containing protein	CsaV3_3G018860: transmembrane emp24 domain-containing protein p24delta3-like	57.21	62.2	67.89	48.36	61.8	80.06	57.79
213	VOC3.1	CsGy3G018650.2: proline-rich protein 36-like	CsaV3_3G018880: leucine-rich repeat extensin-like protein 2	16.66	29.34	43.18	16.35	17.91	30.77	40.86
214	VOC3.1	CsGy3G018660.2: RING-type domain-containing protein	CsaV3_3G018890: E3 ubiquitin-protein ligase RNF126 isoform X1	25.26	45.21	67.26	17.66	29.8	46.16	0
215	VOC3.1	CsGy3G018670.2: pathogenesis-related thaumatin-like protein 3.5	CsaV3_3G018900: Pathogenesis-related thaumatin superfamily protein	2.84	0.59	0.24	0.83	0.95	0.08	0

216	VOC3.1	CsGy3G018680.1: Patatin	CsaV3_3G018910: Patatin	36	39.07	37.7	34.24	34.58	33.95	35.25
217	VOC3.1	CsGy3G018690.2: Phytocyanin domain-containing protein	CsaV3_3G018920: Basic blue protein-like	0.65	17.17	16.41	0	0	0	0
218	VOC3.1	no match	CsaV3_3G018940: basic blue protein-like	0	0.26	0.53	0	0	0	0
219	VOC3.1	no match	CsaV3_3G018960: basic blue protein-like	7.39	21.3	15.21	13.19	7.71	0	1.17
220	VOC3.1	CsGy3G018740.2: Pentatricopeptide repeat-containing protein	CsaV3_3G018970: Pentatricopeptide repeat-containing protein	0.94	0.59	1.4	3.17	3.15	3.92	3.53
221	VOC3.1	CsGy3G018750.2: Sulfurtransferase	CsaV3_3G018980: Sulfurtransferase	68.43	48.01	42.97	90.04	80.11	54.22	47.23
222	VOC3.1	CsGy3G018760.1: 40S ribosomal protein S16	CsaV3_3G018990: 40S ribosomal protein S16	81.24	55.29	50.56	92.02	95.39	30.38	24.93
223	VOC3.1	CsGy3G018770.2: Unknown protein	CsaV3_3G019000: Unknown protein	7.41	10.39	9.95	18.95	20.16	12.91	24.48
224	VOC3.1	no match	CsaV3_3G019010: Nucleolar complex protein 2-like protein	6.55	6.68	8.47	10.28	10.4	10.04	13.84
225	VOC3.1	CsGy3G018790.1: Nigrin b-like	CsaV3_3G019020: Nigrin b-like	0.14	0.23	2.96	0	0	10.28	4.04
226	VOC3.1	CsGy3G018800.2: 6-phosphogluconate dehydrogenase	CsaV3_3G019040: 6-phosphogluconate dehydrogenase family protein	8.18	10.38	11.5	10.16	12.96	16.94	7.49
227	VOC3.1	no match	CsaV3_3G019050: Nigrin b-like	0.55	0.68	5.49	0	0.09	11.3	45.94
228	VOC3.1	CsGy3G018830.2: glutelin type-A 2-like	CsaV3_3G019060: glutelin type-A 2-like	61.49	31.08	29.98	22.71	28.21	24.59	188.04
229	VOC3.1	no match	CsaV3_3G019070: glutelin type-A 2-like	372.62	270.5	307.19	446.69	389.62	403.66	542.6
230	VOC3.1	CsGy3G018860.2: DNA-directed RNA polymerase subunit alpha	CsaV3_3G019080: DNA-directed RNA polymerase subunit alpha	99.62	48.74	66.73	28.39	14.44	31.28	30.79
231	VOC3.1	CsGy3G018900.2: Importin N-terminal domain-containing protein	CsaV3_3G019110: importin-4-like	33.02	37.19	45.06	52.5	53.03	75.36	73.43
232	VOC3.1	CsGy3G018910.2: Membralin-like protein	CsaV3_3G019120: membralin-like	19.82	20.33	23.39	28.41	27.04	25.1	37.21
233	VOC3.1	no match	CsaV3_3G019130: Unknown protein	0.56	0.47	1.87	0	0.36	0	0
234	VOC3.1	CsGy3G018920.2: Protein kinase domain-containing protein	CsaV3_3G019140: Protein kinase domain-containing protein	16.97	13.14	14	5.98	10.22	3.77	12.98
235	VOC3.1	CsGy3G018930.2: UDP-glucose:glycoprotein glucosyltransferase	CsaV3_3G019150: UDP-glucose:glycoprotein glucosyltransferase	11.51	9.29	12.6	22.83	28.01	21.68	19.24
236	VOC3.1	CsGy3G018950.1: UPF0496 protein	CsaV3_3G019170: Unknown protein	3.21	10.46	9.2	1.38	1.51	0.76	0.1
237	VOC3.1	CsGy3G018960.2: Serine hydroxymethyltransferase	CsaV3_3G019180: Serine hydroxymethyltransferase	18.72	21.98	24.22	27.77	20.18	19.84	28.07
238	VOC3.1	CsGy3G018970.2: DNA helicase	CsaV3_3G019190: DNA helicase	1.06	0.58	0.35	2.44	1	0.26	0.71
239	VOC3.1	CsGy3G018980.2: Transmembrane 9 superfamily member	CsaV3_3G019200: Transmembrane 9 superfamily member	92.41	78.79	79.58	119.91	96.44	51.79	55.89
240	VOC3.1	CsGy3G018990.2: Ferredoxin	CsaV3_3G019210: Ferredoxin	18.75	2.03	8.35	15.69	14.32	9.86	0.38
241	VOC3.1	no match	CsaV3_3G019220: Ferredoxin	977.9	248.48	166.32	673.84	461.92	338.1	55.97
242	VOC3.1	CsGy3G019010.2: MLO-like protein	CsaV3_3G019230: MLO-like protein	18.9	18.67	22.52	39.09	46.97	58.95	22.96
243	VOC3.1	CsGy3G019040.2: Magnesium transporter	CsaV3_3G019260: Magnesium transporter	32.82	34.3	32.49	29.05	35.08	46.04	45.75
244	VOC3.1	CsGy3G019070.2: COBRA-like protein	CsaV3_3G019280: COBRA-like protein	2.5	1.13	0.85	3.19	3.41	0.66	0.2
245	VOC3.1	no match	CsaV3_3G019290: Unknown protein	3.2	6.62	3.54	4.83	7.56	3.66	8.06
246	VOC3.1	CsGy3G019160.2: DExH-box ATP-dependent RNA helicase DExH7, chlc	CsaV3_3G019320: DExH-box ATP-dependent RNA helicase DExH7, chloroplastic	7.39	8.79	9	10.44	13.05	15.13	20.81
247	VOC3.1	no match	CsaV3_3G019330: Unknown protein	0	0.28	0.28	0	0.59	0	1.03
248	VOC3.1	no match	CsaV3_3G019340: 60S ribosomal protein L7-1	0.3	0.25	0	0	0	0.19	0
249	VOC3.1	CsGy3G019190.1: hyccin	CsaV3_3G019360: hyccin	2.28	10.99	9.29	4.52	6.6	12.82	10.44
250	VOC3.1	CsGy3G019230.2: receptor-like protein 12	CsaV3_3G019400: LRRNT_2 domain-containing protein	0	0.09	0	0.12	0.29	0	0
251	VOC3.1	CsGy3G019250.2: Transmembrane protein	CsaV3_3G019410: Transmembrane protein	22.45	11.28	14.37	24.72	16.13	6.58	3.95
252	VOC3.1	no match	CsaV3_3G019420: Unknown protein	1.65	0.91	0.91	1.17	2.69	1.54	0
253	VOC3.1	CsGy3G019280.2: Beta-glucuronosyltransferase GlcAT14B-like	CsaV3_3G019440: Beta-glucuronosyltransferase GlcAT14B-like	14.42	18.09	39.22	10.77	13.49	16.27	19.98
254	VOC3.1	CsGy3G019320.2: S4 RNA-binding domain-containing protein	CsaV3_3G019460: S4 RNA-binding domain-containing protein	10.37	6.71	7.15	21.84	18.81	17.87	8.62
255	VOC3.1	CsGy3G019330.2: Enolase-phosphatase E1	CsaV3_3G019470: Enolase-phosphatase E1	8.5	5.52	4.9	9.93	10.46	9.34	9.55
256	VOC3.1	CsGy3G019370.2: Fatty acid oxidation complex subunit alpha	CsaV3_3G019500: 3-hydroxybutyryl-CoA dehydrogenase	57.12	82.6	130.66	66.76	62.61	104.06	192.46
257	VOC3.1	no match	CsaV3_3G019510: 50S ribosomal protein L33-like	98.31	148.54	230.39	63.9	122.43	184.17	10.15
258	VOC3.1	CsGy3G019430.2: DUF3082 domain-containing protein	CsaV3_3G019560: LOW QUALITY PROTEIN: uncharacterized protein LOC103496210	20.2	4.77	1.94	22.25	14.77	0.9	0.26
259	VOC3.1	no match	CsaV3_3G019570: DUF3082 domain-containing protein	19.03	13.87	5.47	31.06	18.65	3.29	7.41
260	VOC3.1	CsGy3G019440.1: glutamic acid-rich protein isoform X1	CsaV3_3G019580: glutamic acid-rich protein isoform X1	65.28	33.71	21.98	31.8	20.34	13.43	12.34
261	VOC3.1	CsGy3G019460.2: PKS_ER domain-containing protein	CsaV3_3G019590: reticulon-4-interacting protein 1, mitochondrial	9.84	10.17	7.99	11.73	13.23	7.46	6.21
262	VOC3.1	CsGy3G019550.2: RRM domain-containing protein	CsaV3_3G019670: RNA-binding protein 2-like isoform X2	47.78	56.86	74.48	55.32	57.59	50.7	28.32
263	VOC3.1	no match	CsaV3_3G019680: Dof-type domain-containing protein	8.96	31.32	36.9	20.41	26.2	28.75	10.79
264	VOC3.1	CsGy3G019560.2: 3'-5' exonuclease domain-containing protein	CsaV3_3G019690: 3'-5' exonuclease domain-containing protein	6.25	5.11	7.62	16.88	14.95	9.21	9.82
265	VOC3.1	CsGy3G019570.1: thylakoid lumenal 17.4 kDa protein, chloroplastic	CsaV3_3G019700: thylakoid lumenal 17.4 kDa protein, chloroplastic	53.75	19.45	11.59	43.49	31.24	6.34	2.1
266	VOC3.1	CsGy3G019580.2: Calcium-binding EF hand-like protein	CsaV3_3G019710: reticulocalbin-2	1.53	1.56	2.65	6.5	11.37	2.84	0.4
267	VOC3.1	CsGy3G019610.2: KH domain-containing protein	CsaV3_3G019740: RNA-binding KH domain-containing protein	19.6	20.67	18.25	31.01	35.26	25.22	19.16
268	VOC3.1	CsGy3G019620.2: 50S ribosomal protein L9, chloroplastic	CsaV3_3G019750: 50S ribosomal protein L9, chloroplastic	46.77	35.38	31.63	38.06	41.47	40.7	32.91
269	VOC3.1	CsGy3G019660.2: Myosin heavy chain	CsaV3_3G019790: Myosin heavy chain	1.39	2.02	1.15	0.99	0.42	0.88	0.39
270	VOC3.1	no match	CsaV3_3G019800: myosin-7B	8.75	4.34	3.86	11.23	6.37	3.25	1.08
271	VOC3.1	no match	CsaV3_3G019810: Unknown protein	1.25	0.35	0.87	2.41	1.69	1	0.29
272	VOC3.1	CsGy3G019670.2: DUF4005 domain-containing protein	CsaV3_3G019820: protein IQ-DOMAIN 1	3.92	17.74	8.46	0.98	0.81	0.15	0.56
273	VOC3.1	CsGy3G019750.1: calcineurin B-like protein 4	CsaV3_3G019930: calcineurin B-like protein 4	2.28	3.46	7.42	7.09	6.08	2.12	0.43
274	VOC3.1	CsGy3G019770.1: CCT domain-containing protein	CsaV3_3G019950: CCT domain-containing protein	363.24	316.05	258.09	551.82	703.75	338.15	21.21
275	VOC3.1	CsGy3G019780.1: Histone-lysine N-methyltransferase	CsaV3_3G019960: histone-lysine N-methyltransferase ATX4	4.39	4.41	5.42	6.79	11.55	10.23	8.1
276	VOC3.1	CsGy3G019790.2: Potassium transporter	CsaV3_3G019990: Potassium transporter	19.82	22.96	21.79	23.93	25.12	31.7	22.59
277	VOC3.1	no match	CsaV3_3G020020: Unknown protein	151.81	133.24	87.34	105.71	73.75	37.08	54.57
278	VOC3.1	CsGy3G019810.1: Protein CbbY-like	CsaV3_3G020030: Family of unknown function (DUF716)	128.34	108.03	75.22	83.78	64	35.81	52
279	VOC3.1	no match	CsaV3_3G020040: Unknown protein	42.51	35.22	33.07	52.27	28.45	11.95	26.68
280	VOC3.1	CsGy3G019820.1: Xpo1 domain-containing protein	CsaV3_3G020050: Protein of unknown function (DUF803)	24.02	24.72	28.34	47.44	46.89	32.77	20.04
281	VOC3.1	CsGy3G019830.1: DUF761 domain-containing protein	CsaV3_3G020060: Unknown protein	25.56	20.71	10.69	5.61	8.37	1.09	0.16
282	VOC3.1	CsGy3G019840.1: BnaA09g02830D protein	CsaV3_3G020070: Unknown protein	15.74	9.73	11	17.94	11.3	9.33	7.29
283	VOC3.1	CsGy3G019860.2: DEAD-box ATP-dependent RNA helicase 41	CsaV3_3G020080: DEAD-box ATP-dependent RNA helicase	8.2	10.45	13.63	10.71	14.36	20.7	19.09
284	VOC3.1	no match	CsaV3_3G020090: Unknown protein	21.2	25.88	23.06	9.59	13.43	26.22	14.84
285	VOC3.1	CsGy3G019870.2: OTU domain-containing protein	CsaV3_3G020100: OTU domain-containing protein	13.55	16.99	16.44	8.46	9.34	13.38	14.02
286	VOC3.1	CsGy3G019880.2: Glyceraldehyde-3-phosphate dehydrogenase	CsaV3_3G020110: Glyceraldehyde-3-phosphate dehydrogenase	278.26	53.47	62.6	226.28	137.45	82.59	2.22
287	VOC3.1	no match	CsaV3_3G020120: WD repeat-containing protein LWD1	59.5	40.21	35.76	20.56	32.83	27.31	20.3
288	VOC3.1	CsGy3G019890.2: Acetyl-coenzyme A synthetase	CsaV3_3G020130: Acetyl-coenzyme A synthetase	63.62	73.58	41.61	74.13	80.8	54.27	38.16

289	VOC3.1	CsGy3G019900.2: Xpo1 domain-containing protein	CsaV3_3G020140: transportin-3 isoform X1	11.58	12.6	11.34	12.64	15.96	15.61	18.48
290	VOC3.1	CsGy3G019970.2: protein root UVB sensitive 1, chloroplastic	CsaV3_3G020160: protein root UVB sensitive 1, chloroplastic isoform X1	10.37	4.6	3.68	8.81	8.62	4.86	9.54
291	VOC3.1	CsGy3G020030.1: Chloroplastic group iib intron splicing facilitator crs2, c	CsaV3_3G020220: chloroplastic group IIB intron splicing facilitator CRS2-B, chloroplastic	8.26	4.7	5.52	14.1	13.46	7.87	11.89
292	VOC3.1	no match	CsaV3_3G020230: Unknown protein	0.72	0.6	0	0	0	0	0
293	VOC3.1	no match	CsaV3_3G020240: Homeobox Hox-B3-like protein	10.47	0.82	0.49	1.97	0.78	0.11	0
294	VOC3.1	CsGy3G020040.2: DUF4378 domain-containing protein/VARLMGL dom	CsaV3_3G020250: DUF4378 domain-containing protein/VARLMGL domain-containing protein	47.36	34.91	31.69	27.6	20.07	25.15	31.58
295	VOC3.1	no match	CsaV3_3G020260: Unknown protein	35.49	23.89	17.51	15.36	16.32	26.45	0
296	VOC3.1	CsGy3G020050.2: MADS-box domain-containing protein	CsaV3_3G020270: MADS-box domain-containing protein	0.97	1.07	0.94	0.69	0.18	0	0.23
297	VOC3.1	CsGy3G020060.2: Elongator complex protein 5	CsaV3_3G020280: Elongator complex protein 5	10.12	11.86	8.94	14.46	14.52	9.65	10.13
298	VOC3.1	CsGy3G020070.2: Transcription elongation factor SPT4 homolog	CsaV3_3G020290: Transcription elongation factor SPT4 homolog	70.58	51.32	45.7	46.79	53.88	47.63	51.05
299	VOC3.1	CsGy3G020080.1: DUF775 domain-containing protein	CsaV3_3G020300: DUF775 domain-containing protein	38.13	24.31	34.7	56.25	41.98	14.43	22.6
300	VOC3.1	CsGy3G020100.2: ABC1 domain-containing protein	CsaV3_3G020320: AarF domain-containing protein kinase 1	7.95	8.45	8.84	14.61	18.98	16.39	13.46
301	VOC3.1	CsGy3G020130.2: Mental retardation GTPase activating protein	CsaV3_3G020330: Mental retardation GTPase activating protein	19.3	16.44	13.45	27.31	19.86	13.53	9.94
302	VOC3.1	no match	CsaV3_3G020340: Unknown protein	27.7	26.48	46.46	16.19	34.95	31.91	31.67
303	VOC3.1	CsGy3G020140.2: Ribosome biogenesis ERB1	CsaV3_3G020350: Unknown protein	13.94	15.41	20.5	21.1	21.97	17.91	33.38
304	VOC3.1	**CsGy3G020170.1: MYB transcription factor protein 1**	**CsaV3_3G020410: protein rough sheath 2 homolog**	47.32	21.26	9.95	33.15	38.05	12.82	2.15
305	VOC3.1	no match	CsaV3_3G020420: Unknown protein	3.41	8.48	1.13	3.27	0.77	0.77	0.58
306	VOC3.1	CsGy3G020180.2: Cycloidea-like protein group 1A	CsaV3_3G020440: transcription factor TCP1	9.72	3.68	2.2	5.18	2.54	0.06	0
307	VOC3.1	CsGy3G020190.2: Abhydrolase_3 domain-containing protein	CsaV3_3G020450: isoprenylcysteine alpha-carbonyl methylesterase ICME-like	15.61	10.74	11.94	25.77	18	15.75	21.16
308	VOC3.1	CsGy3G020200.2: GDSL esterase/lipase	CsaV3_3G020460: GDSL esterase/lipase	1	1.25	0.42	1.73	1.29	1.39	0.26
309	VOC3.1	CsGy3G020210.2: Centromere protein F	CsaV3_3G020470: Centromere protein F	24.4	26.55	18.64	32.58	34.67	14.04	12.23
310	VOC3.1	CsGy3G020220.2: PMR5N domain-containing protein	CsaV3_3G020480: Protein trichome birefringence-like 19	41.02	57	70.03	50.5	41.38	54.2	46.76
311	VOC3.1	CsGy3G020230.2: Eukaryotic translation initiation factor 3 subunit G	CsaV3_3G020490: Eukaryotic translation initiation factor 3 subunit G	101.42	90.41	82.62	66.87	90.69	99.75	51.41
312	VOC3.1	CsGy3G020240.2: RING-type E3 ubiquitin transferase BRCA1	CsaV3_3G020510: E3 ubiquitin-protein ligase rnf8-A isoform X1	4.03	3.89	4.21	7.74	4.39	4.08	4.99
313	VOC3.1	CsGy3G020250.1: Uridine kinase	CsaV3_3G020520: Uridine kinase	45.59	42.64	34.19	63.12	65.97	50.42	27.73
314	VOC3.1	CsGy3G020280.1: 30S ribosomal protein 3	CsaV3_3G020570: 30S ribosomal protein 3, chloroplastic-like	97.46	30.35	17.26	79.88	29.27	13.2	23.57
315	VOC3.1	CsGy3G020290.2: IST1-like protein	CsaV3_3G020580: Regulator of Vps4 activity in the MVB pathway protein, putative	1.05	1.64	0.97	0.25	0.94	0	0.45
316	VOC3.1	no match	CsaV3_3G020590: Unknown protein	3.49	4.63	2.32	3.1	0.81	0.43	4.48
317	VOC3.1	CsGy3G020300.2: Alpha-1,3-glucosyltransferase	CsaV3_3G020610: Alpha-1,3-glucosyltransferase	9.71	9.99	6.57	7.48	6.06	8.05	12.71
318	VOC3.1	no match	CsaV3_3G020620: Phosphatidylserine decarboxylase proenzyme 2	3.08	3.51	2.72	2.78	1.81	3.35	6.7
319	VOC3.1	no match	CsaV3_3G020630: Unknown protein	0.63	0.78	0	0.39	0	0.9	1.86
320	VOC3.1	CsGy3G020330.1: cytokinin hydroxylase	CsaV3_3G020640: Cytochrome P450, putative	0	0.64	0.45	0	0	0	0
321	VOC3.1	CsGy3G020340.2: LOB domain-containing protein	CsaV3_3G020650: LOB domain-containing protein	234.69	232.96	95.36	114.93	268.26	15.21	26.68
322	VOC3.1	no match	CsaV3_3G020680: Unknown protein	220.69	231.15	187.56	294.4	391.13	247.5	156.41
323	VOC3.1	CsGy3G020360.1: protein ABA DEFICIENT 4, chloroplastic	CsaV3_3G020700: protein ABA DEFICIENT 4, chloroplastic	84.12	57.05	58.3	85.31	56.95	40.45	49.92
324	VOC3.1	no match	CsaV3_3G020720: phenolic glucoside malonyltransferase 1-like	17.17	5.9	3.4	5.23	6.37	0.83	1.1
325	VOC3.1	no match	CsaV3_3G020730: Unknown protein	6.05	3.13	1.26	4.37	1.33	1.13	2.22
326	VOC3.1	CsGy3G020400.2: BHLH domain-containing protein	CsaV3_3G020750: transcription factor BEE 3-like	1.63	1.35	2.85	2.26	3.04	4.99	0.23
327	VOC3.1	CsGy3G020430.2: H15 domain-containing protein	CsaV3_3G020800: HMG-Y-related protein A-like	126.14	84.88	51.67	52.14	51.38	31.28	37.97
328	VOC3.1	CsGy3G020440.2: Senescence-associated family protein	CsaV3_3G020810: senescence-associated family protein	11.43	19.18	47.19	39.18	46.42	68.07	31.94
329	VOC3.1	CsGy3G020460.2: Valyl-tRNA synthetase	CsaV3_3G020830: Valyl-tRNA synthetase	11.14	11.79	8.04	14.52	14.86	14.62	17.09
330	VOC3.1	CsGy3G020480.2: glutathione S-transferase DHAR3, chloroplastic	CsaV3_3G020840: glutathione S-transferase DHAR3, chloroplastic	32.85	21.72	19.11	32.3	20.66	17.31	14.46
331	VOC3.1	CsGy3G020500.1: DELLA protein	CsaV3_3G020850: DELLA protein	125.04	67.74	47.52	49	81.19	19.54	5.66
332	VOC3.1	CsGy3G020520.2: Senescence-associated protein	CsaV3_3G020860: Unknown protein	0	0.66	0.66	0	0	1.27	1.13
333	VOC3.1	no match	CsaV3_3G020870: Retrovirus-related Pol polyprotein from transposon TNT 1-94	3.11	5.48	3.23	10.79	7.1	7.13	5.23
334	VOC3.1	no match	CsaV3_3G020880: Unknown protein	26.07	18.48	3.42	1.22	0.22	0	0
335	VOC3.1	no match	CsaV3_3G020920: Unknown protein	3.37	3.91	1.4	2.28	1.19	0.4	0
336	VOC3.1	CsGy3G020590.2: RRM domain-containing protein	CsaV3_3G020960: THO complex subunit 4D	45	54.23	56.48	66.72	79.59	84.93	85.77
337	VOC3.1	CsGy3G020610.1: GDSL esterase/lipase	CsaV3_3G020990: GDSL esterase/lipase	0.86	0.54	0.18	1.78	0	0	0
338	VOC3.1	CsGy3G020620.2: MYB transcription factor 20	CsaV3_3G021000: Myb transcription factor	0.76	0.38	0	0.86	0.54	0	0
339	VOC3.1	CsGy3G020640.2: Protein kinase domain-containing protein	CsaV3_3G021010: Protein kinase domain-containing protein	20.05	19.79	12.9	26.7	24.73	16.12	6.81
340	VOC3.1	CsGy3G020650.1: glutamine synthetase 1;4	CsaV3_3G021030: Glutamine synthetase	126.44	254.71	426.35	95.69	118.87	185.1	188.5
341	VOC3.1	CsGy3G020660.1: TATA-binding protein-associated factor BTAF1	CsaV3_3G021040: TATA-binding protein-associated factor BTAF1	2.2	3.11	1.97	8.43	7.85	5.71	11.87
342	VOC3.1	no match	CsaV3_3G021050: TATA-binding protein-associated factor BTAF1	2.65	2.2	1.65	8.61	10.49	8.91	10.6
343	VOC3.1	no match	CsaV3_3G021060: TATA-binding protein-associated factor BTAF1	4.34	4.28	3.26	9.3	10.62	8.62	7.87
344	VOC3.1	no match	CsaV3_3G021070: Unknown protein	0.92	2.02	0.76	0	0.36	0	0.94
345	VOC3.1	no match	CsaV3_3G021080: TATA-binding protein-associated factor BTAF1	22.21	14.98	14.28	10.79	11.08	12.82	10.33
346	VOC3.1	CsGy3G020670.2: SET domain-containing protein	CsaV3_3G021090: protein SET DOMAIN GROUP 40-like	15.05	20.56	19.92	28.85	30.65	40.03	32.04
347	VOC3.1	no match	CsaV3_3G021100: PC-Esterase	11.53	11.67	10.96	7.35	8.07	4.81	9.87
348	VOC3.1	CsGy3G020680.2: UTP--glucose-1-phosphate uridylyltransferase	CsaV3_3G021110: UTP--glucose-1-phosphate uridylyltransferase	224.82	175.16	173.29	273.86	189.81	108.18	114.75
349	VOC3.1	CsGy3G020690.1: RING-type E3 ubiquitin transferase	CsaV3_3G021120: RING-type E3 ubiquitin transferase	19.46	26.2	31.43	0.93	26.33	5.41	47.09
350	VOC3.1	CsGy3G020700.2: nijmegen breakage syndrome 1 protein	CsaV3_3G021130: nijmegen breakage syndrome 1 protein	8.87	8.37	7.62	16.68	10.84	9.26	15.54
351	VOC3.1	CsGy3G020710.2: Unknown protein	CsaV3_3G021140: Unknown protein	492.25	462.81	503.9	293.32	269.44	318.35	310.13
352	VOC3.1	CsGy3G020720.2: Importin N-terminal domain-containing protein	CsaV3_3G021150: importin-11	16.92	21.31	21.53	34.97	37.33	32.59	37.58

* Numbers are normalized gene expression value in FPKM (Fragments Per Kilobase of transcript per Million mapped reads) from NCBI accessions SRA071224 (Wei et al. 2016) and GSE116581 (Lu et al. 2018).

** FL = Cucumber fruit flesh; numbers are age of fruit (days after anthesis)
